# The treeness of the tree of historical trees of life

**DOI:** 10.1371/journal.pone.0226567

**Published:** 2020-01-15

**Authors:** Marie Fisler, Cédric Crémière, Pierre Darlu, Guillaume Lecointre

**Affiliations:** 1 UMR 7205 CNRS-MNHN-SU-EPHE « Institut de Systématique, Evolution et Biodiversité », département « Origines & Évolution », Muséum National d’Histoire Naturelle, Paris, France; 2 Musée d’Histoire Naturelle du Havre, Place du vieux marché, Le Havre, France; 3 UMR 7206 CNRS-MNHN-UPD « Eco-anthropologie et Ethnobiologie », département « Hommes, Nature et Sociétés », Muséum National d’Histoire Naturelle, Paris, France; Universite de Lausanne Faculte de biologie et medecine, SWITZERLAND

## Abstract

This paper compares and categorizes historical ideas about trees showing relationships among biological entities. The hierarchical structure of a tree is used to test the global consistency of similarities among these ideas; in other words we assess the “treeness” of the tree of historical trees. The collected data are figures and ideas about trees showing relationships among biological entities published or drawn by naturalists from 1555 to 2012. They are coded into a matrix of 235 historical trees and 141 descriptive attributes. From the most parsimonious “tree” of historical trees, treeness is measured by consistency index, retention index and homoplasy excess ratio. This tree is used to create sets or categories of trees, or to study the circulation of ideas. From an unrooted network of historical trees, treeness is measured by the delta-score. This unrooted network is used to measure and visualize treeness. The two approaches show a rather good treeness of the data, with respectively a retention idex of 0.83 and homoplasy excess ratio of 0.74, on one hand, and a delta-score of 0.26 on the other hand. It is interpreted as due to vertical transmission, i.e. an inheritance of shared ideas about biological trees among authors. This tree of trees is then used to test categories previously made. For instance, cladists and gradists are « paraphyletic ». The branches of this tree of trees suggest new categories of tree-thinkers that could have been overlooked by historians or systematists.

## Introduction

Treeness, the degree to which shared features among entities fit to a rooted hierarchical non-cyclic connected graph (a « tree »), in other words the degree to which such a connected graph can be described as a rooted tree, is a topical matter of investigation in comparative cultural data [1–5]. The question of transmission of scientific symbols, figures and ideas through times is today at the interface between history of science, philosophy of science and formalized comparative methods that were born in the realm of systematics [6]. These methods are the basis from which systematists create concepts (or categories in the wide sense), i.e. justified sets of objects with a name attached to them. In a previous article, we showed that these methods are suitable for history of science [7]. Indeed, historians create sets of authors and ideas, gathered into some categories (lamarckians, darwinians, cladists, pheneticists, linneans, etc.) in a non-formalized manner. Fisler and Lecointre [7] showed that using a hierarchical non-cyclic connected graph (usually called a rooted « tree ») obtained through parsimony methods, one can possibly obtain nested sets of concepts, test the consistency of previous categories of thinkers, and discover new ones. We will not come back on that point.

Primary data were extracted from the various trees showing relationships among biological entities published (most of them drawn) by the scientific community between 1766 and 1991 [8], [9], [10], [11]. Their conceptual and graphical similarities were coded into a matrix of 41 historical trees x 91 characters from which a parsimonious tree called « tree of trees » was obtained. Here we expand that matrix to 235 historical trees drawn from the period between 1555 and 2012, and each of them described by 141 characters.

The first question is how to choose the best representation of the data. A hierarchical non-cyclic connected graph (a « tree ») will draw complete categories even though the data are poorly hierarchical (weak treeness), in such a case the tree would be said poorly robust or non-robust. Remember that a category of objects is said complete when it does not leave outside an object that bears the properties that justify the category. Even if a tree representation is a suitable way to obtain the most consistent (poor or strong) combination of hierarchical nested sets of objects, it does not necessarily show along its branches all the relevant similarities that are coded in the data matrix, because of the presence of homoplasies. Networks have the advantage to exhibit links induced by similarities and make ambiguities highly visible. However complete categories cannot be deduced from an unrooted network [12], [13]: p 334] because there is no mean to know if a set of objects is complete because the root is outside it, or incomplete because the root is located inside it. Moreover, there is a huge collection of hierarchically incompatible sets on an unrooted network so that it is difficult to choose among them ([12]: p 572). Let’s take advantage of both rooted trees and networks: tree will be used to discuss categories and their robustness, and network to get a visual impression of the treeness of the data. Treeness measurements (RI and HER) will be calculated for tree representations, and delta-score for networks.

The second question is to test if the different categories of naturalists previously recognized by systematists (like cladists, pheneticists, gradists, etc.) or by historians (linneans, lamarckians, darwinians, etc.) are consistent with a tree structure representation, and to discover possibly new categories. Last but not the least, the improvement of the data matrix, compared to the primary ones [7], will lead to test previous published categories and to discuss the circulation of ideas about published biological trees through times.

## Materials and methods

Following the framework described in Fisler and Lecointre [7], we selected 235 historical trees published between 1555 and 2012 in the scientific field of natural history and in which the purpose to represent relationships among biological entities was expressed and/or illustrated by naturalists. The period was chosen because the metaphor or the picture of “tree” started to be usual in natural history in the middle of the XVIth century. Most of these publications were selected because they encompass well-known and/or fundamental literature in systematics, and because they all have a theoretical, empirical and/or educational content. They may talk about biology, paleontology, or even they can be philosophical essays. They all have in common to display and comment representations that could be named “trees of life”, even if sometimes only a part of living diversity is taken into account. We limited the sampling to the modern scientific era so that trees used to support metaphysical views beyond science before the XVII^th^ century [14] are out of the scope of the present analysis. In the same way, we chose to limit our sample to natural history, keeping only trees depicting the diversity of life, not trees organizing knowledge (e.g. [15]) or languages (e.g. [16–20]) or any other kind of classified objects. The languages of the selected publications were English, French, German and Latin. The list of trees chosen are described in the appendix (annex I). These “historical Trees” are treated as operational taxonomic units (OTUs). Characters describing each OTU are detailed in the appendix (annex II) and succinctly included below when describing the groups identified in the tree of trees. The final matrix contains 235 OTU (or published trees) and 141 characters (S1–S3 Files).

Standard parsimony approach was conducted using PAUP* 4.0b10 [21] to get the parsimonious tree(s). Characters of the matrix were treated as unordered and unweighted trees and they are all informative. Heuristic searches were performed with 1000 random addition sequences and TBR branch swapping. The results are shown under a 100%-majority-rule consensus tree of the parsimonious trees (strict consensus). Characters changes are optimized on that tree using the ACCTRAN option, favouring reversions over convergences when equi-parsimonious choice among these two possibilities is given. The reason is that, if exchange of ideas (possibly by transfer, or circulation of ideas) is to be tested and inferred, ACCTRAN is the most conservative condition of inferring convergences, as only convergences that do not depend on the choice among the two optimization possibilities are taken into account. ACCTRAN or DELTRAN options have no role on the estimation of treeness anyway. The tree was rooted using an all-zero hypothetical ancestor. This is justified by the fact that, in the coding of character states, a « zero » symbol was given to code for the absence of a given trait. Therefore, this rooting option implicitly considers that the OTUs with the lowest number of ideas is basal, meaning that evolution of ideas should proceed through gaining ideas rather than losing them.

We calculated Consistency Index (CI [22]), Retention Index (RI [23]), and Homoplasy Excess Ratio (HER, [23]) as homoplasy measurements, but only RI and HER as relevant criteria to discuss the structure of our data. CI = M/S, where M is the minimum number of character changes given the number of character states, and S the actual number of character changes (“steps”) required in the parsimonious tree(s). The CI may be overestimated because of autapomorphic changes (i.e. changes unique to a given OTU) and it is negatively correlated with the number of OTUs. The Retention Index and the HER correct this. RI = (G-S)/(G-M), where G is the maximum number of steps implied by the matrix (the number of steps of a tree with a single node, i.e. no resolution at all), M is the minimum of steps implied by the matrix (the sum of character amplitudes), and S is the number of necessary steps in the parsimonious tree(s) [24], [25]. RI measures the excess of homoplasy regardless of autapomorphic changes. HER has the same formula as RI, but with G replaced with A, A being the average length of the parsimonious trees calculated from randomly perturbed matrices (with conservation of the number of character states). RI and HER better reflect the hierarchical structure of the data matrix than CI does [4], [26]. The first one starts from the data structure itself, the second from a random process. Comparison allows to better appraise how close to randomness character states are distributes across taxa.

The network was constructed using SplitsTree v.4 [27]. SplitsTree only uses two-states characters. For the 19 characters that are multistate, we decomposed them into several binary characters. To measure treeness, the delta-score [28] was calculated. This score is based on the four point condition which supposes that a tree is totally resolved or “tree-like” if, for any set of four OTU (*x*,*y*,*u*,*v*), the distances between them satisfy the following condition:

$$d_{xy|uv}\leq d_{xu|yv}\leq d_{xv|yu}\;\mathrm{with}\;d_{xy|uv}=d_{xy}+d_{uv}$$

The delta-sore δ is the mean over all quartets of the ratios: $\delta_{q}=\frac{d_{xv|yu}-d_{xu|yv}}{d_{xv|yu}-d_{xy|uv}}$.

δ varies between 0 and 1, being 0 when all the four points conditions hold and converging to 1 as far as the tree structure moves away for a tree-like.

Support of the branches was calculated using a non-resampling method in order to keep the internal structure of the data, i.e. Bremer support [29]. The Bremer support for a node is simply the extra length (expressed in number of steps) needed to lose the node in the strict consensus of less parsimonious trees.

RI, HER and delta-score were used to estimate the treeness of the tree of trees. Other methods of data analysis than trees or networks might be relevant enough to be applied, such as multivariate methods, like PCA, MCA or MDS. However, although they are designed to highlight proximities between OTUs, they do not allow deducing a hierarchical structure integrating an explicit historical dimension which is our main concern. Similarly, VMS or machine learning methods are no more appropriate for our project, which is not to draw discriminant functions to distinguish OTUs sharing a limited number of features in common. Clearly, although all these methods might be applied to our data set, they pursue different objectives basically different from the present one.

## Results

### Treeness

**Fig 1:**
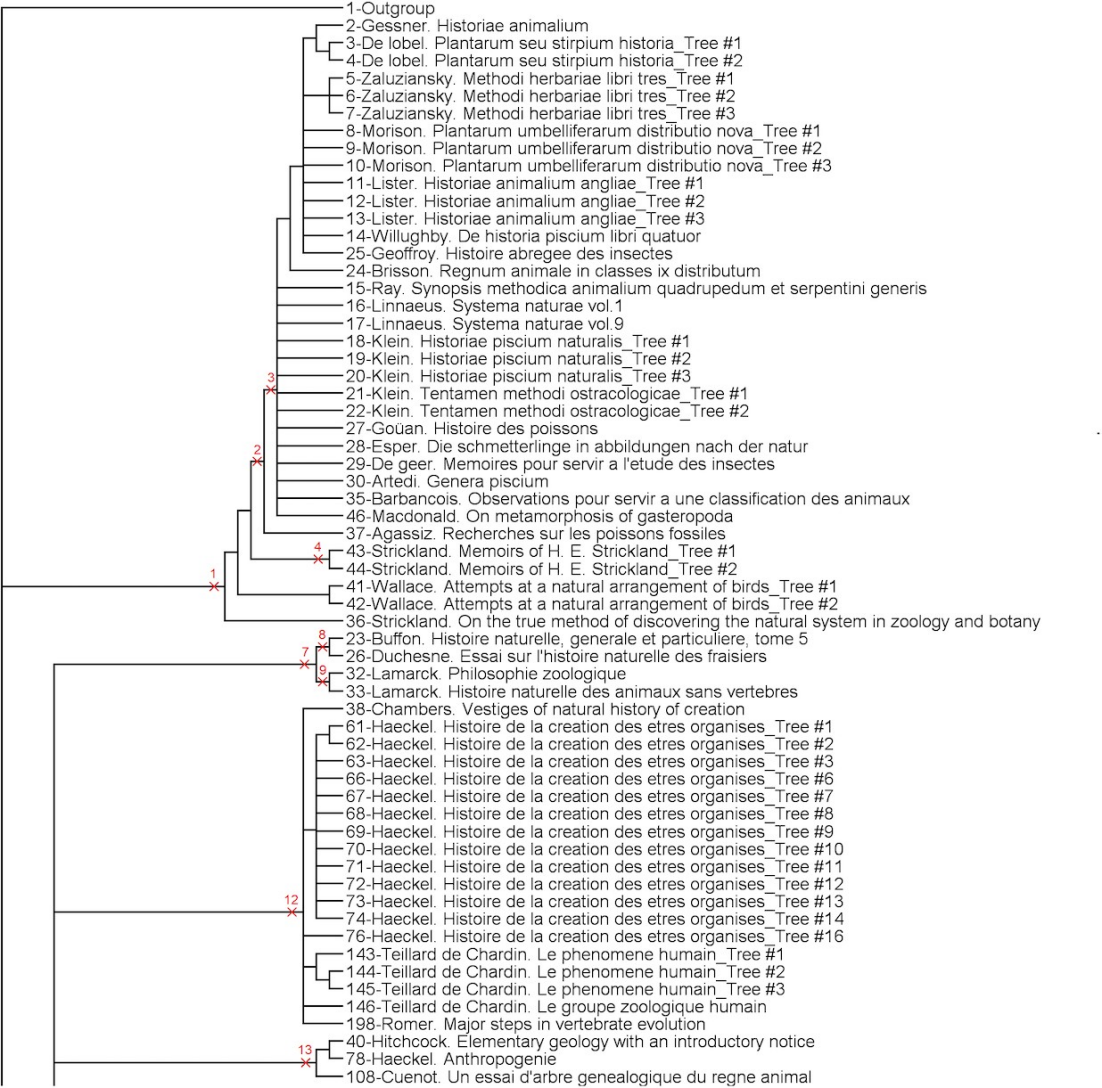
Upper part of the strict consensus tree from 3087 equi-parsimonious trees of 1217 steps (CI: 0.13, RI: 0.83, HER: 0.74). Numbers on branches refer to groups described below.

**Fig 2:**
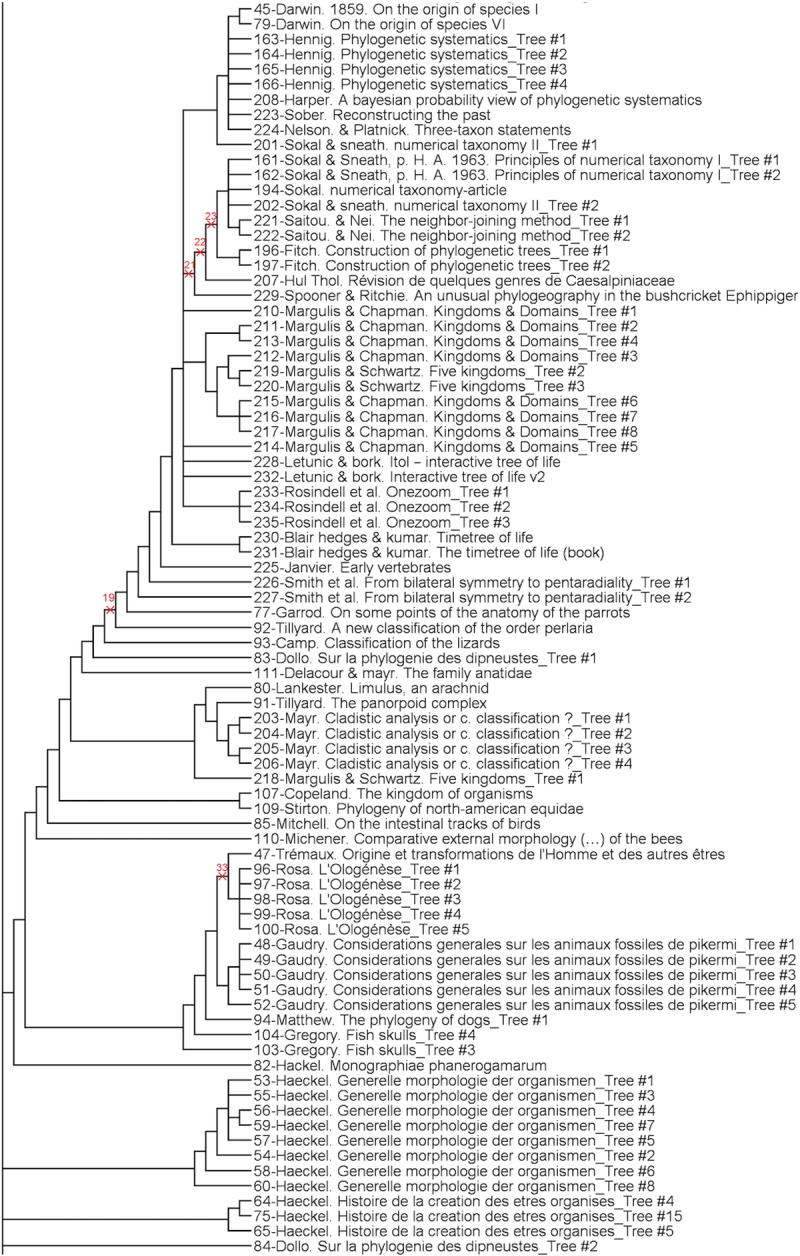
Middle part of the strict consensus tree.

**Fig 3:**
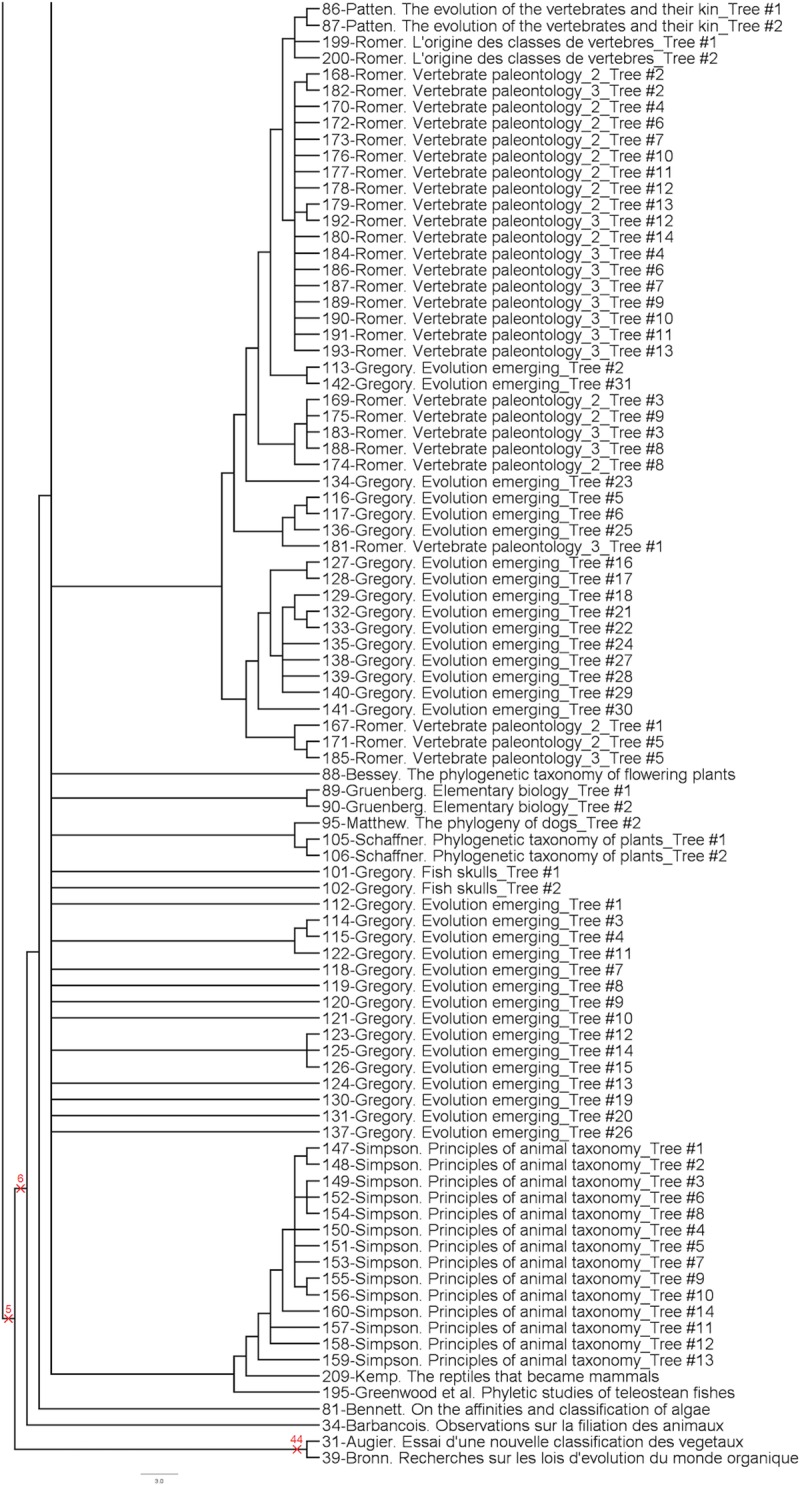
Lower part of the strict consensus tree.

**Fig 4:**
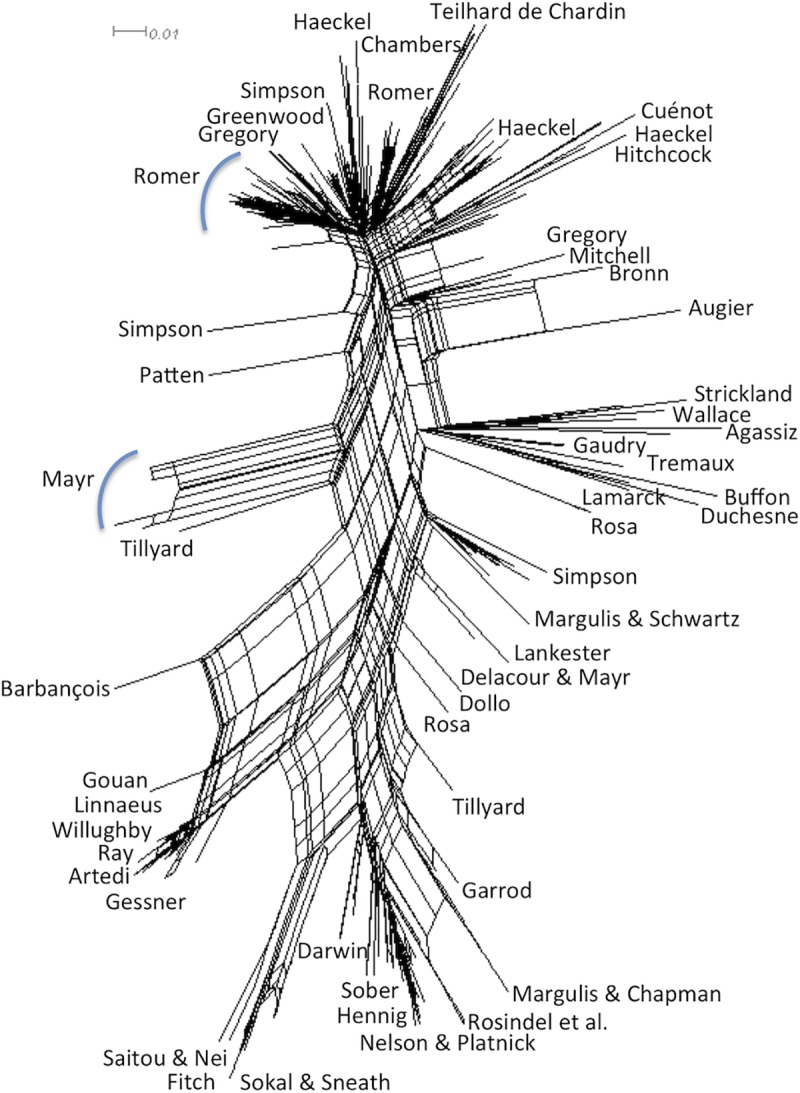
Split network obtained with SplitsTree (Delta Score: 0.26). For simplicity, the tips of the branches are not all labelled.

The analysis provided 3087 parsimonious trees of 1217 steps, with a CI of 0.13, a RI of 0.83 and a HER of 0.74 (strict consensus tree shown Figs 1–3). The network (Fig 4) has a delta-score of 0.26. The RI and HER values indicate a good level of hierarchical structure in the data matrix. The two indices are very close to each other, suggesting that homoplastic characters–those that do not perfectly fit to the tree- are not congruently structured among them but rather randomly distributed. The delta score shows a moderate but significant treeness, 0 corresponding to perfect treeness. The network, once generated, provides a visual impression of the treeness of the data albeit it cannot create categories (see [12]). This is the reason why we do not identify all OTUs at the tip of the branches in Fig 4.

### Categories

A complete category is justified with at least one property (a character state changing at the node, i.e. a synapomorphy) and all objects having this property are included in the category. To generate complete categories, one can virtually create a category per node in Figs 1–3. This will provide nested categories. In the tree, some previously recognized groups (or categories) are already identified:

#### Identification trees (group #3)

**Fig 5:**
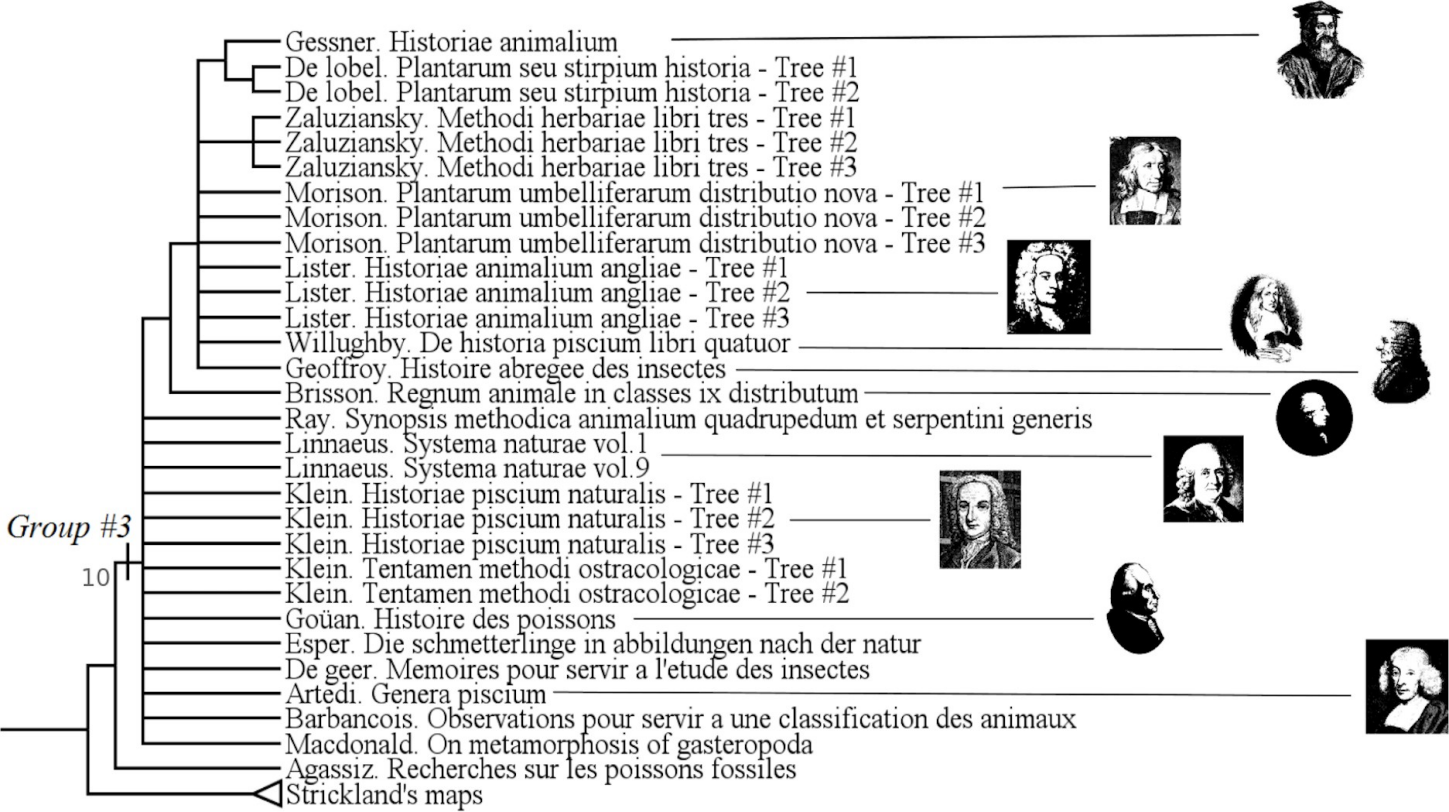
Subtree showing the group #3 (called “Identification Trees”).

The group #3 is the group of “Identification trees” [8], [30], [11]. It includes authors like Linnaeus, Klein or Gessner. (Fig 5)

It has a Bremer index of 10 and is supported by 22 characters:

Character #2 (The root refers to a named entity: No);

Character #5 (The root refers to discretized properties: Yes);

Character #22 (Branches show groups which follow one another: No);

Character #29 (Branches express discretized properties: Yes);

Character #33 (The branches can rotate around the nodes: Yes);

Character #42 (The leaves show discretised properties: Yes);

Character #45 (The leaves are thick: No);

Character #47 (The objects located at the leaves are the only classified objects: Yes);

Character #54 (The hierarchical axis shows an interlocking of the groups: Strict);

Character #55 (The hierarchical axis shows a succession of groups: No);

Character #58 (The hierarchical axis shows the diversity of the shapes: No);

Character #60 (The hierarchical axis shows discretised properties: Yes);

Character #75 (The ancestral morphotype still exists at the present time: Yes);

Character #80 (Entities are grouped because they share a typical form: No);

Character #85 (The groups are the basis of the classification and must be linked to one another: No);

Character #99 (Justification of how species are classified on the tree ?: Yes);

Character #102 (The tree is a simplification of reality: No);

Character #107 (Evolution rates are different among bloodlines: No);

Character #111 (Can a form be reached by several different bloodlines at the same time?: Yes);

Character #122 (The method used to draw the tree is general: No);

Character #125 (The tree has a Practical reach: Yes);

Character #131 (The tree must follow overall similarity: No).

An *Identification tree*, or *Identification Key*, is a tree-shaped pattern helping to identify a species.

This group #3 has a strong temporal coalescence: almost all the authors from the Renaissance to the classical age are grouped there, and there is almost no other author.

In those trees, the figure allows to identify a species as well as to classify it among others.

Pietsch [11] described them as “bracketed tables” (p.7) in his chapter “*Brackets and Tables*, *Circles and Maps”*. Here, only bracketed tables are grouped. Tassy [8] also recognized this group as “*Arbres parenthésés*” (bracketed trees).

#### « Trees of life » (group #5)

**Fig 6:**
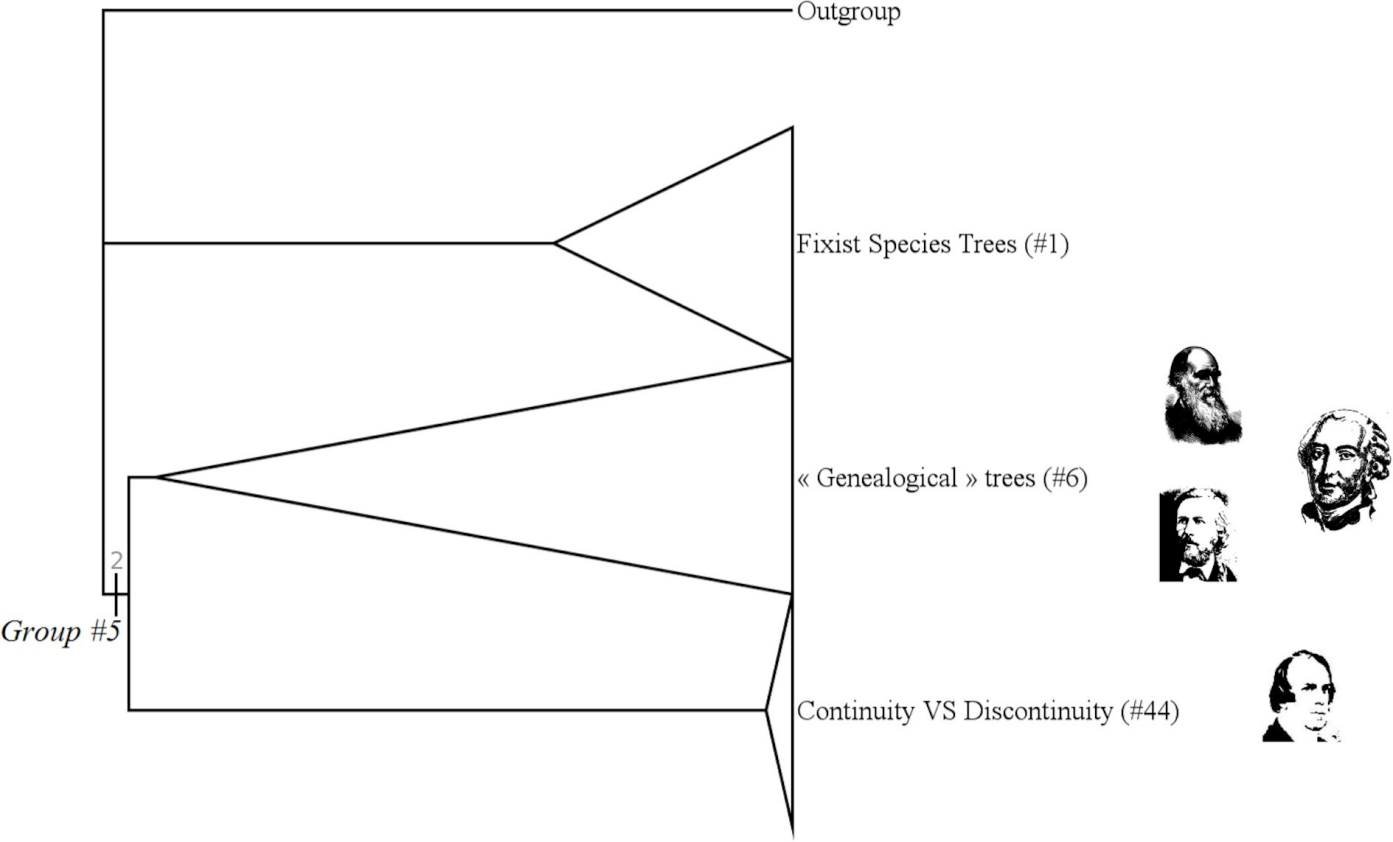
Subtree showing the group #5 (called “Trees of Life”).

The group #5 is the group of “Trees of Life” [10], [11]. It includes authors like Augier, Darwin or Lamarck. (Fig 6)

It has a Bremer index of 2 and is supported by 13 characters:

Character #1 (The root carries the idea of an appreciative amount of value: Yes, Negative);

Character #3 (The root refers to a supposed living being?: Yes, considered as a "stem-group");

Character #13 (The trunk shows an evolutionary direction: No);

Character #24 (Some branches show a change in shape: Yes);

Character #29 (Branches express discretized properties: No);

Character #32 (The branches show affinity links: No);

Character #40 (The entity located at the nodes is reconstructed: By analogy);

Character #46 (The leaves reconstruct the tree: Not only them);

Character #59 (The hierarchical axis shows quantifiable data: No);

Character #62 (The hierarchical axis shows a temporal order: Relative);

Character #65 (The diversification axis shows complexity levels: Yes);

Character #121 (The tree is worth for all life: No);

Character #129 (The tree topology is created: No).

*Trees of life* are figures aimed at classifying species. As the name suggests figures are more or less directly inspired by the shape of a tree. There is not necessarily an evolutionary theory underlying this classification: Bronn's and Augier's trees are not evolutionary trees and do not consider any idea of evolution. Trees of Life is what several authors, among them Pietsch, describe in their works. Pietsch names those figures “trees” or “diagrams”.

#### Genealogical trees (group #6)

**Fig 7:**
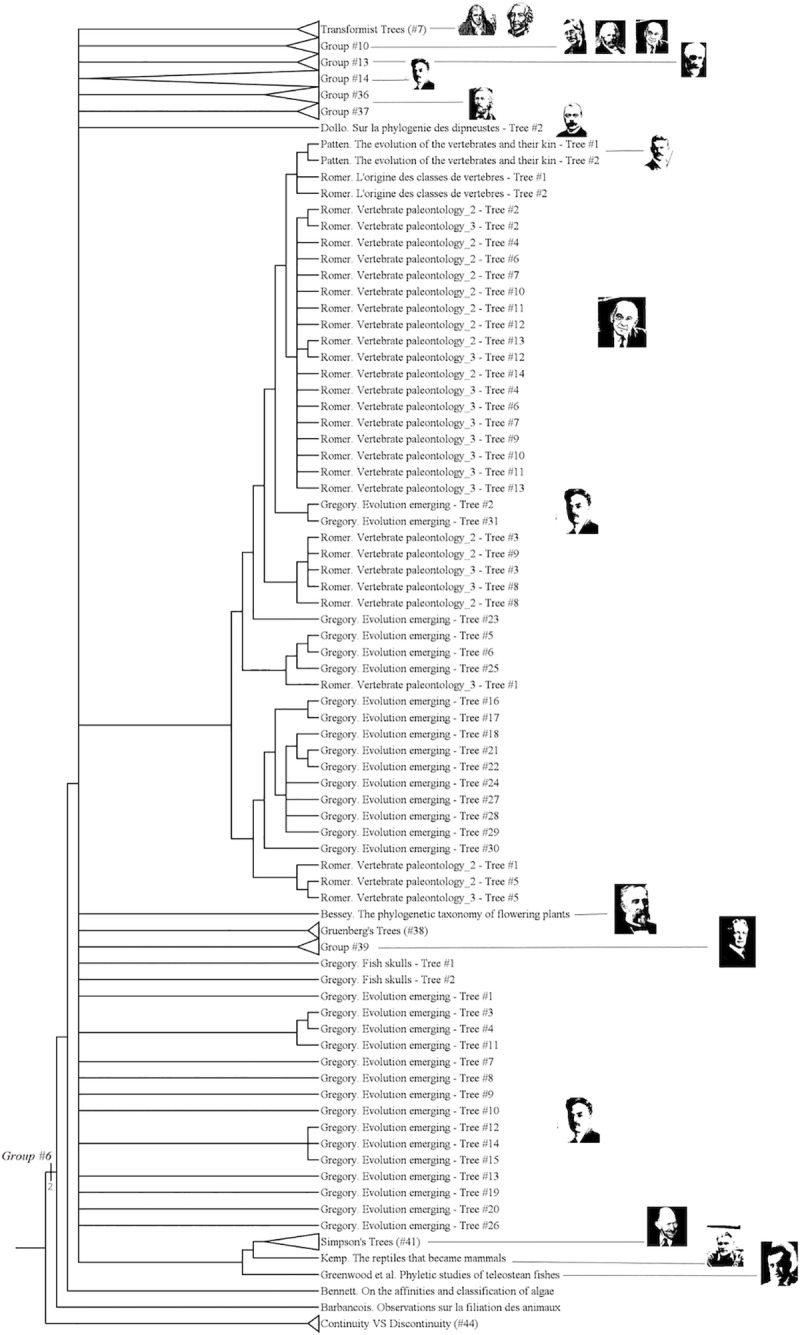
Subtree showing the group #6 (called “Genealogical Trees”).

The group #6 is the group of “« genealogical » trees” [31]. It includes authors like Darwin, Bronn or Haeckel. (Fig 7)

It has a Bremer index of 2 and is supported by 12 characters:

Character #17 (The trunk has an aesthetic intention: No);

Character #19 (The trunk is thick: No);

Character #37 (The nodes correspond to an ancestor: Yes);

Character #48 (Bubbles enable to visualize the quantity of species according to the time: Yes);

Character #49 (The bubbles enable to visualize an evolutionary gradation: Yes);

Character #52 (Bubbles are covering the branches: Yes);

Character #77 (Special place for mankind: For a group to which mankind belong);

Character #108 (Evolution rates are different among times ?: Yes);

Character #113 (Expressed Relations: Genealogy: Yes);

Character #124 (The tree has a Theoretical reach: No);

Character #130 (The tree must follow genealogical links: Yes);

Character #132 (How is the time taken into account in the tree?: The tree is genealogical), and this character has a CI of 0,4.

“*Genealogical*” trees include the idea of evolution or transformism in life. The tree reflects a genealogy, on a way or another. Those trees are classically what we think about trees of life. For example, they are what Grimoult [31] described in his *Histoire de l'évolutionnisme contemporain en France*.

#### Mutationist trees (group #7)

**Fig 8:**
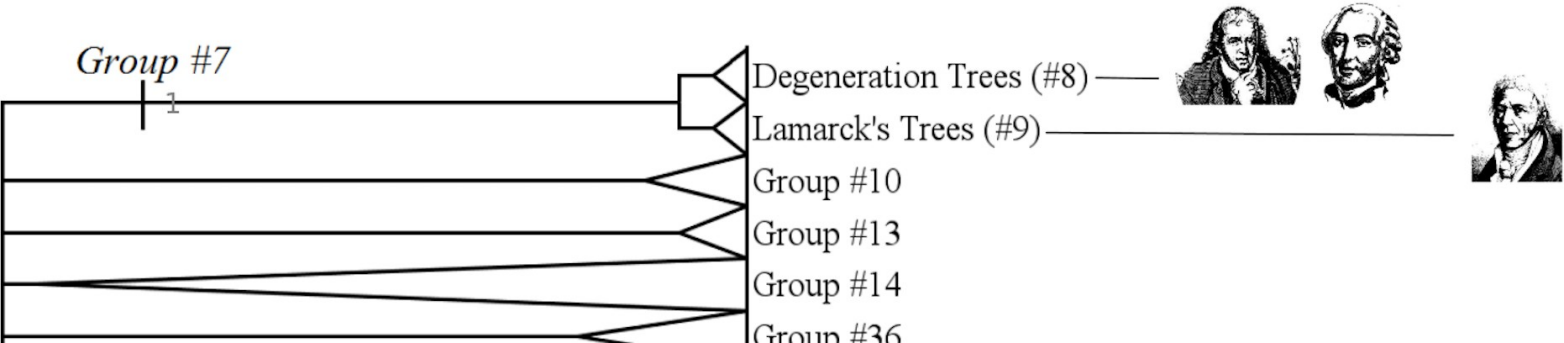
Subtree showing the group #7 (called “Mutationist Trees”).

The group #7 is the group of “mutationist Trees” [32], [33] or “Transformism” [34]. It includes Buffon, Duchesne and Lamarck. (Fig 8)

It has a Bremer index of 1 and is supported by 21 characters:

Character #2 (The root refers to a named entity?: Yes, and it is a species);

Character #9 (What is located at the root still exists: Yes);

Character #26 (What's on the branches still exists today: Yes);

Character #32 (The branches show affinity links: No);

Character #39 (The entity located at the nodes can be detected materially: Yes);

Character #40 (The entity located at the nodes is reconstructed: No);

Character #46 (The leaves reconstruct the tree: No);

Character #54 (The hierarchical axis shows an interlocking of the groups: No interlocking);

Character #73 (The ancestor is a less adapted state of a group: No);

Character #79 (Entities are grouped because they have points in common: No);

Character #82 (Entities are grouped because they share a level of complexity: No);

Character #84 (The groups are an arbitrary convention: Yes), and this character has a CI of 1;

Character #99 (Justification of how species are classified on the tree ?: Yes);

Character #101 (Justification of the tree representation ?: Yes);

Character #102 (The tree is a simplification of reality: Yes);

Character #107 (Evolution rates are different among bloodlines ?: No);

Character #108 (Evolution rates are different among times ?: No);

Character #122 (The method used to draw the tree is general: No);

Character #124 (The tree has a Theoretical reach: Yes);

Character #126 (The tree has a Didactical reach: No);

Character #128 (The tree topology is reconstructed: No).

Mutationism (in the sense of Grimoult [32]) is the idea of limited species transformations across generations, passing from one form to another form within a range limited by a morphological reference or standard. Three authors do present this kind of transformist ideas: Buffon ([35] but this is discussed, see [36]), Duchesne and Lamarck. Other transformists *sensu lato* are situated elsewhere in the tree of trees.

#### Degeneration trees (group #8)

**Fig 9:**
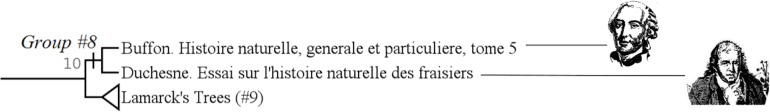
Subtree showing the group #8 (called “Limited-transformism trees”).

The group #8 is the group of “degeneration trees”, gathering authors belonging to a school also called “Limited-transformism” by Grimoult ([32]: pp 24–25), [37], Barsanti, ([9]:p 91), Gaudant and Gaudant ([35]: pp 196–197),or the “buffonian school” (node 44) of Fisler and Lecointre [7]. This group includes Buffon and Duchesne. It has a strong Bremer index of 10 (Fig 9)

It is supported by 11 characters:

Character #1 (The root carries the idea of an appreciative amount of value: Yes, Positive);

Character #38 (The nodes correspond to a type: No);

Character #57 (The hierarchical axis shows complexity levels: No);

Character #76 (The ancestor is: An individual);

Character #77 (Special place for mankind: No);

Character #87 (The groups can be modified if new data require it: Yes);

Character #90 (Reticulations?: Yes);

Character #109 (Increasing complexity ?: No);

Character #118 (Classified Objects: Species: Yes);

Character #119 (Classified Objects: objets with a rank above the species one: No);

Character #134 (Characters states used: Arbitrary choice).

The idea of “degeneration” appears firstly in Buffon's *Histoire Naturelle*, followed by his student, Duchesne. Degeneration is a kind of transformism. This group #8 is mainly based on the character #1 (the root bears something of positive value that degenerates along the tree) and had previously been recognized by Armand de Quatrefages in his *Darwin et ses précurseurs français*. *Etude sur le transformisme* ([38]: p 43).

#### Teilhard de Chardin's trees (group #12)

**Fig 10:**
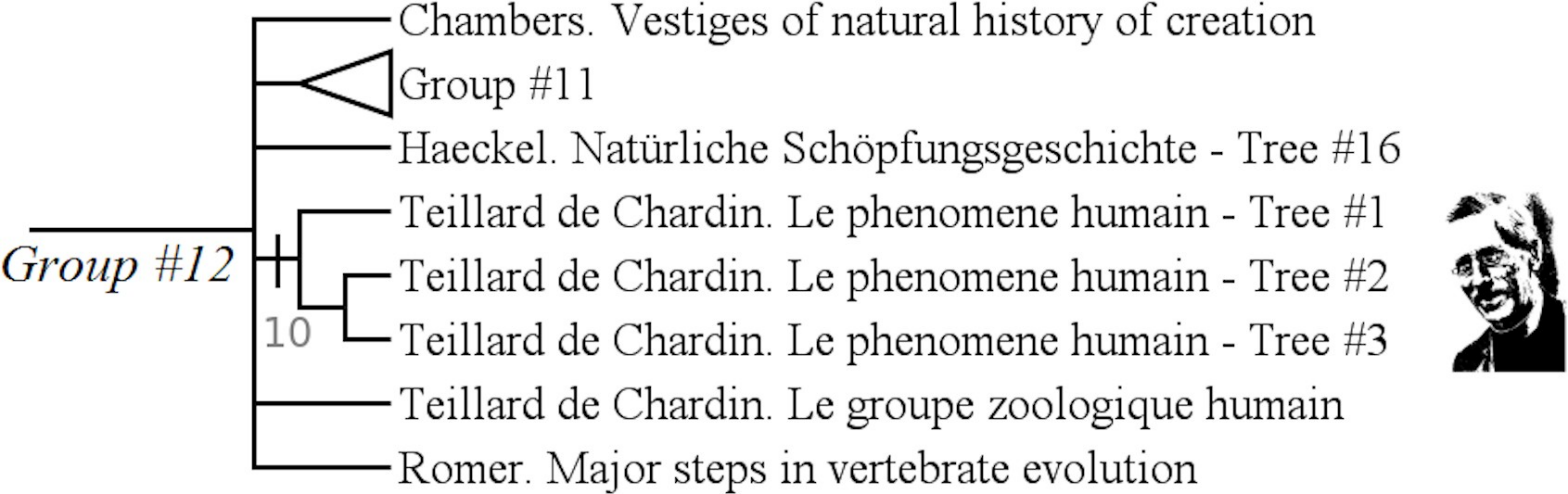
Subtree showing the group #12 (called “Teilhard de Chardin's trees”).

The group #12 is the group of “Teilhard de Chardin's trees”, recognized by Grimoult [31], Tassy [39] and de Bonis [40] (Fig 10).

It has a Bremer index of 10 and is supported by 20 characters:

Character #2 (The root refers to a named entity?: No);

Character #23 (Some branches show a purpose to which the evolution leads: Yes);

Character #62 (The hierarchical axis shows a temporal order: Strict);

Character #63 (The hierarchical axis shows the time as dates: Yes);

Character #65 (The diversification axis shows complexity levels: Yes);

Character #77 (Special place for mankind: Yes);

Character #100 (Justification of the classificatory system ?: Yes);

Character #103 (Classificatory purpose?: No);

Character #105 (Finalism, direction in changes?: Yes);

Character #123 (The tree has an Epistemological reach: No);

Character #124 (The tree has a Theoretical reach: Yes);

Character #134 (Characters states used: Arbitrary choice);

Character #135 (Characters used: Arbitrary choice).

This group illustrates the homogeneity of Teilhard de Chardin's trees. Grimoult in his *Histoire de l'évolutionnisme contemporain en France (*[31]: p 117) classified this author as belonging to a “*finalisme chrétien” (Christian finalism)*.

#### Pheneticists (group #22)

**Fig 11:**
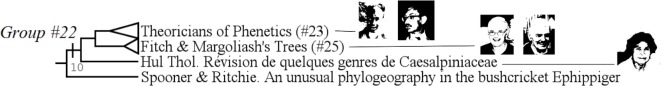
Subtree showing the group #22 (called “Pheneticists”).

The group #22 is the group of “Pheneticists” [8], [30], [41]: pp 79, 263), [42], [43], [44], [45], [46]. It includes authors like Hul Thol, Fitch & Margoliash and Sokal & Sneath (Fig 11).

It has a Bremer index of 10 and is supported by 10 characters:

Character #5 (The root refers to discretized properties: No);

Character #29 (Branches express discretized properties: No);

Character #35 (The nodes correspond to a set of discretised properties: No);

Character #62 (The hierarchical axis shows a temporal order: No);

Character #99 (Justification of how species are classified on the tree ?: Yes);

Character #100 (Justification of the classificatory system ?: Yes);

Character #101 (Justification of the tree representation ?: Yes);

Character #130 (The tree must follow genealogical links: No);

Character #136 (Outgroup ?: No);

Character #137 (Selection criterion of the tree: maximization of sharings: No).

Phenetics is a method of elaboration of trees based on overall similarity, defined by Sokal & Sneath in 1963, in their *Numerical Taxonomy*. This group had previously been recognized by numerous authors, among them Pascal Tassy in his *Arbre à remonter le Temps* [8]

#### Previous categories unrecovered

Some groups, though previously recognized by some historians of Biology, have not been recovered in this *Tree of Trees*: Gradists, Cladists and Affinity Trees.

The group of “Gradists” (also called “Systématique évolutionniste” by Mayr, [43] and Dupuis [47], also called “syncretists” [30], [44], [48], but see [46] is not recovered: it is splitted, as in Fisler and Lecointre [7]. In other words, it is an “uncomplete group”. This means that “non-gradist” authors, such as Hennig, Darwin or Sokal & Sneath are found among gradists. This group is broken by several nodes, among which the node bearing group #15 which includes authors like Stirton, Mayr, Hennig or Darwin, and has a Bremer index of 1 and is supported by 12 characters.

**Fig 12:**
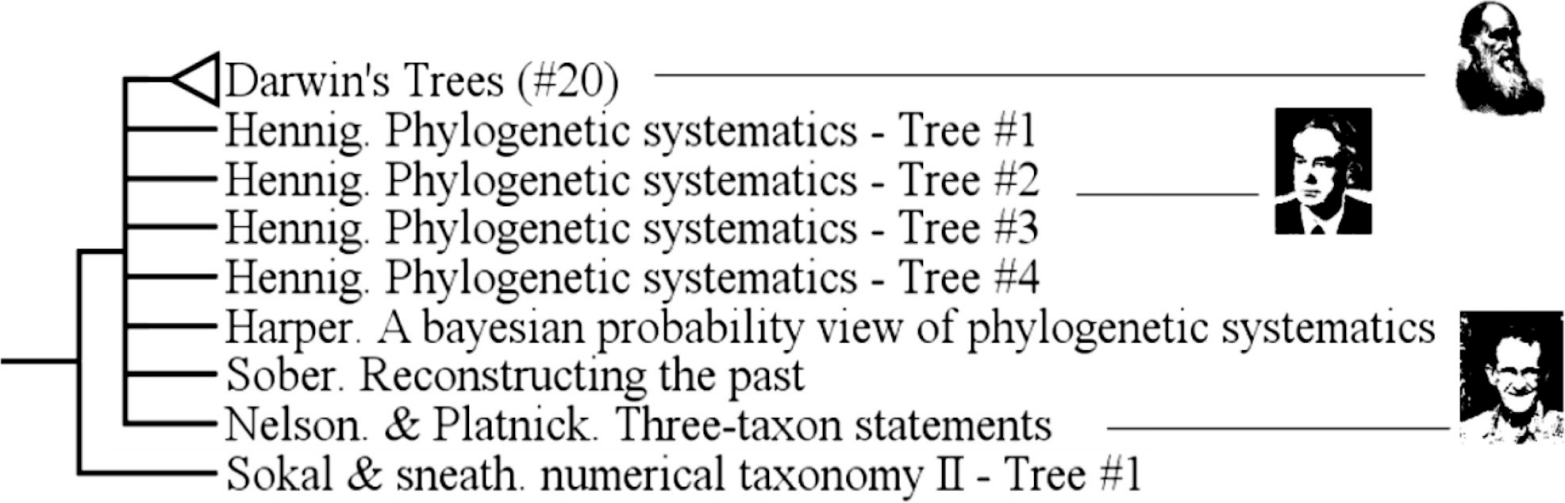
Subtree showing the lack of resolution among Darwin's trees and “cladist theoreticians” trees.

The monophyly of the group of “Cladist theoreticians” [30], [43], [44], [45], [46], [49], [50], -that should also gather Hennig, Sober, Nelson, Platnick, the latter being part of group #20- is not obtained. With them are branched in a polytomy other authors like Harper or Darwin: qualifying Darwin as a cladist would be anachronistic, given that he lived 100 years before the rise of cladistics. However, cladists are neither monophyletic nor paraphyletic because there is no resolution within the clade. (Fig 12)

The group of Affinity Trees [51], [52], [53] is another uncomplete group. It is broken by the insertion of the node-group of “identification trees” among “affinity trees”.

The whole group has a Bremer Index of 2 and is sustained by 7 characters. Other groups categorized by Fisler and Lecointre [7] are not recovered here because the increase of historical trees sampling either reveals paraphylies (cladists, node 63 of Fisler and Lecointre), or increases the number of poorly-supported nodes contradicting them (“initial trees users”, node 78, “tree makers”, node 79, “evolutionists”, node 72, “grade theoreticians”, 66, “metaphoricians”, 60, “strictly genealogical classifications”, 65, connected graphs users, 70, similarity classifiers, 69).

#### Shaping new categories

We could pinpoint each node of the tree (Figs 1–3) and create a category from it that contains everything downstream. But such an exercise is only worth for robust nodes. In other words, the reliability of the category depends on the level of support or the robustness of the node from which it has been created. Here we chose to detail the nodes with a Bremer support of 2 or above, and/or a number of supporting characters above 7.

#### Fixist species trees (group #1)

**Fig 13:**
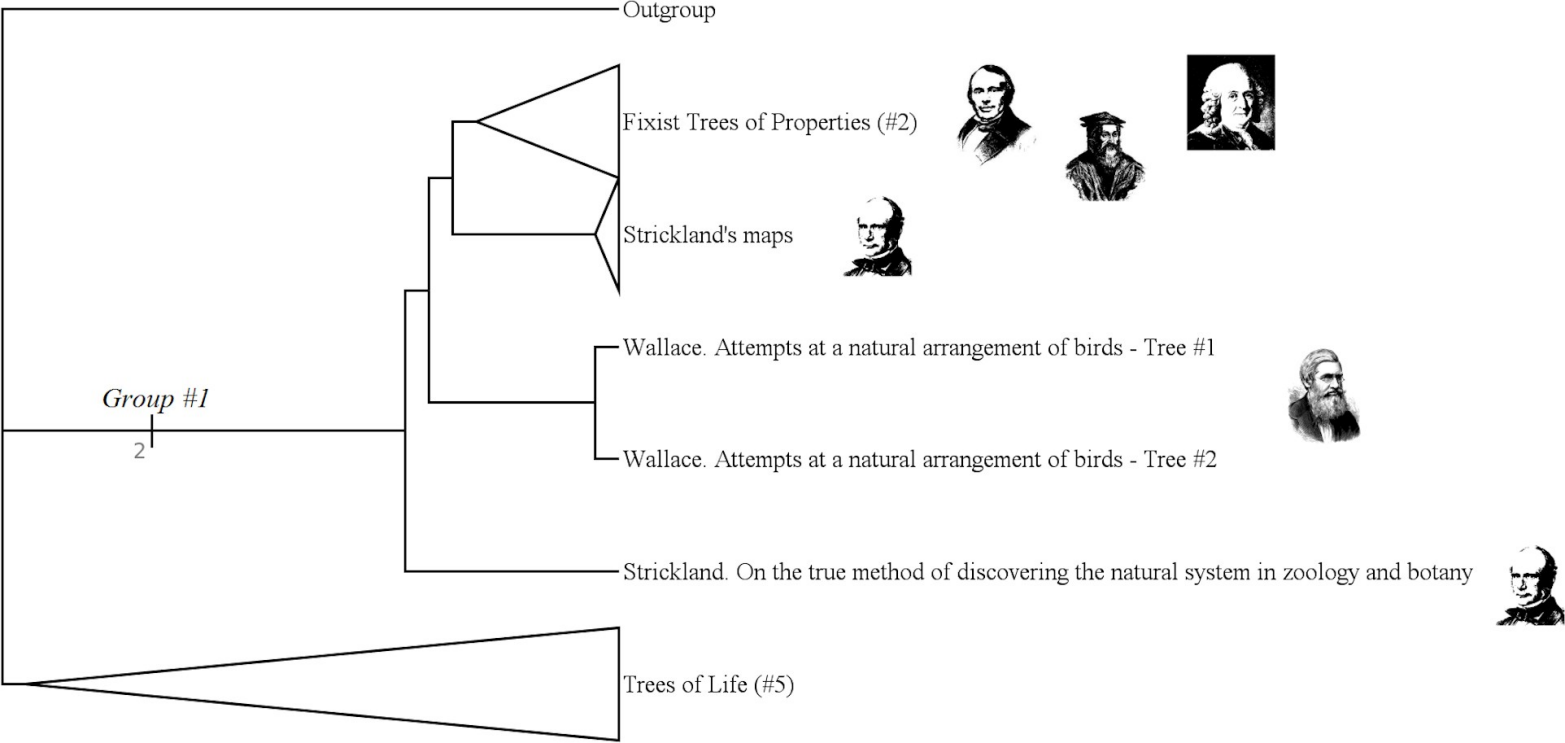
Subtree showing the group #1 (called “Fixist species trees”).

The group #1 is the group of “Fixist Species Trees”. It includes authors like Wallace, Strickland or Linnaeus. (Fig 13)

It has a Bremer index of 2 and is supported by 7 characters:

Character #26 (What's on the branches still exists today: No);

Character #38 (The nodes correspond to a type: No);

Character #53 (The hierarchical axis shows a gradation of the values: No);

Character #57 (The hierarchical axis shows complexity levels: No);

Character #61 (The hierarchical axis shows a similarity rate: Yes);

Character #82 (Entities are grouped because they share a level of complexity: No);

Character #112 (Does the tree express a gradation in value between beings?: No).

It is not surprising that Wallace belongs to “fixist” authors. Indeed, when Wallace wrote about the evolution of species, he did not draw any tree. Conversely, when he drew trees, they are inspired by Strickland's fixist methods: those trees group species according to their affinities. Linnaeus also belongs to this group: in his *Systema Naturae*, two trees are drawn. The first one is found in the fourth part of the first edition of this work: it is a key to the insects. The second one is found in the tenth edition of *Systema Naturae*: it is a classification of plants.

#### Fixist trees of properties (group #2)

**Fig 14:**
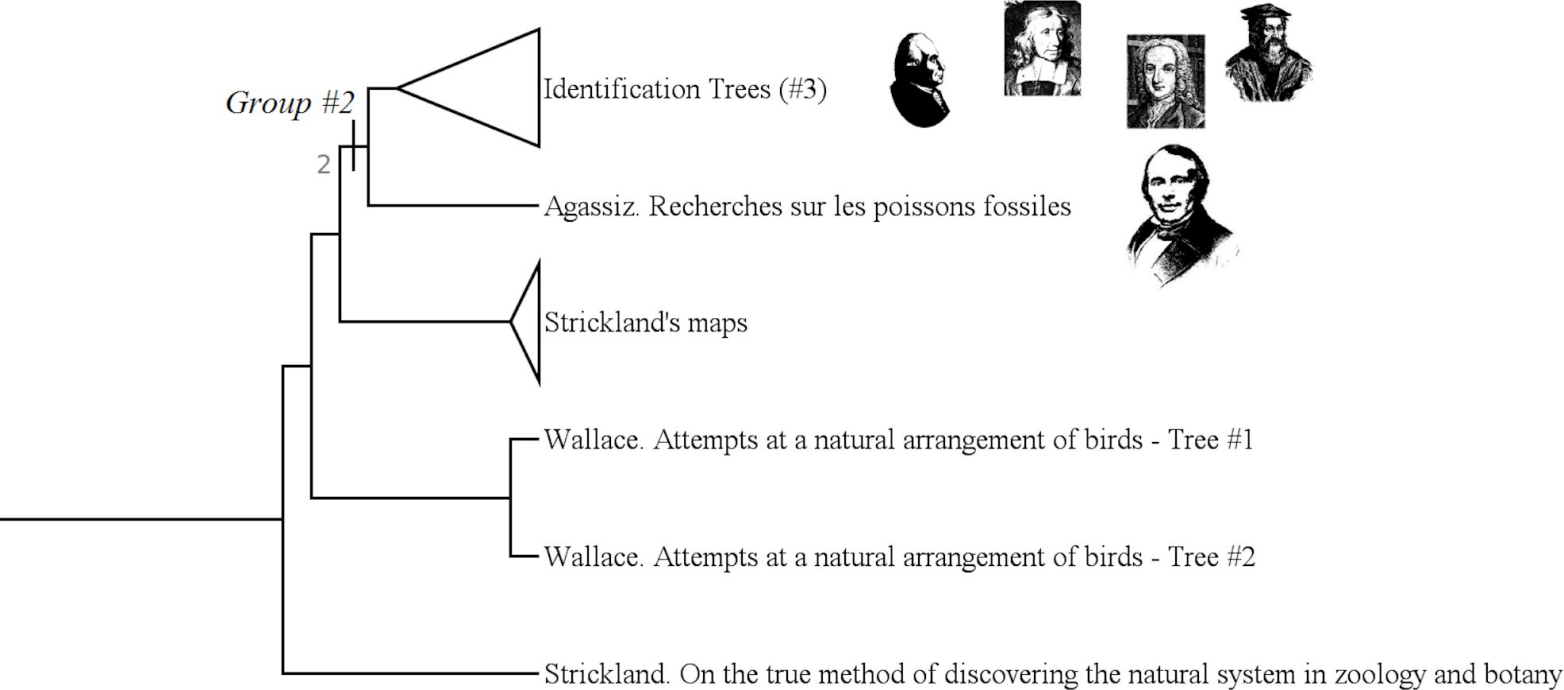
Subtree showing the group #2 (called “Fixist trees of properties”).

The group #2 is the group of “Fixist Trees of Properties”. It includes authors like Agassiz, Linnaeus or Klein. (Fig 14)

It has a Bremer index of 2 and is supported by 7 characters:

Character #32 (The branches show affinity links: No);

Character #35 (The nodes correspond to a set of discretised properties: Yes);

Character #48 (Bubbles enable to visualize the quantity of species according to the time: Yes);

Character #61 (The hierarchical axis shows a similarity rate: No);

Character #93 (Elaboration of "complete" groups?: Yes, as a result of a classification program);

Character #134 (Characters states used: Arbitrary choice);

Character #135 (Characters used: Arbitrary choice).

Those *Trees of Properties* gather what we classically call “brackets trees” with Agassiz' tree. These authors group species according to a set of properties: scales, wings, reproductive organs, etc. There are no affinity links between those species, but only a sharing of characteristics.

“Brackets trees” do not have “bubbles”, but Agassiz' one does so. Those “bubbles” enable to visualize the quantity of species according to time.

#### Group #4

**Fig 15:**
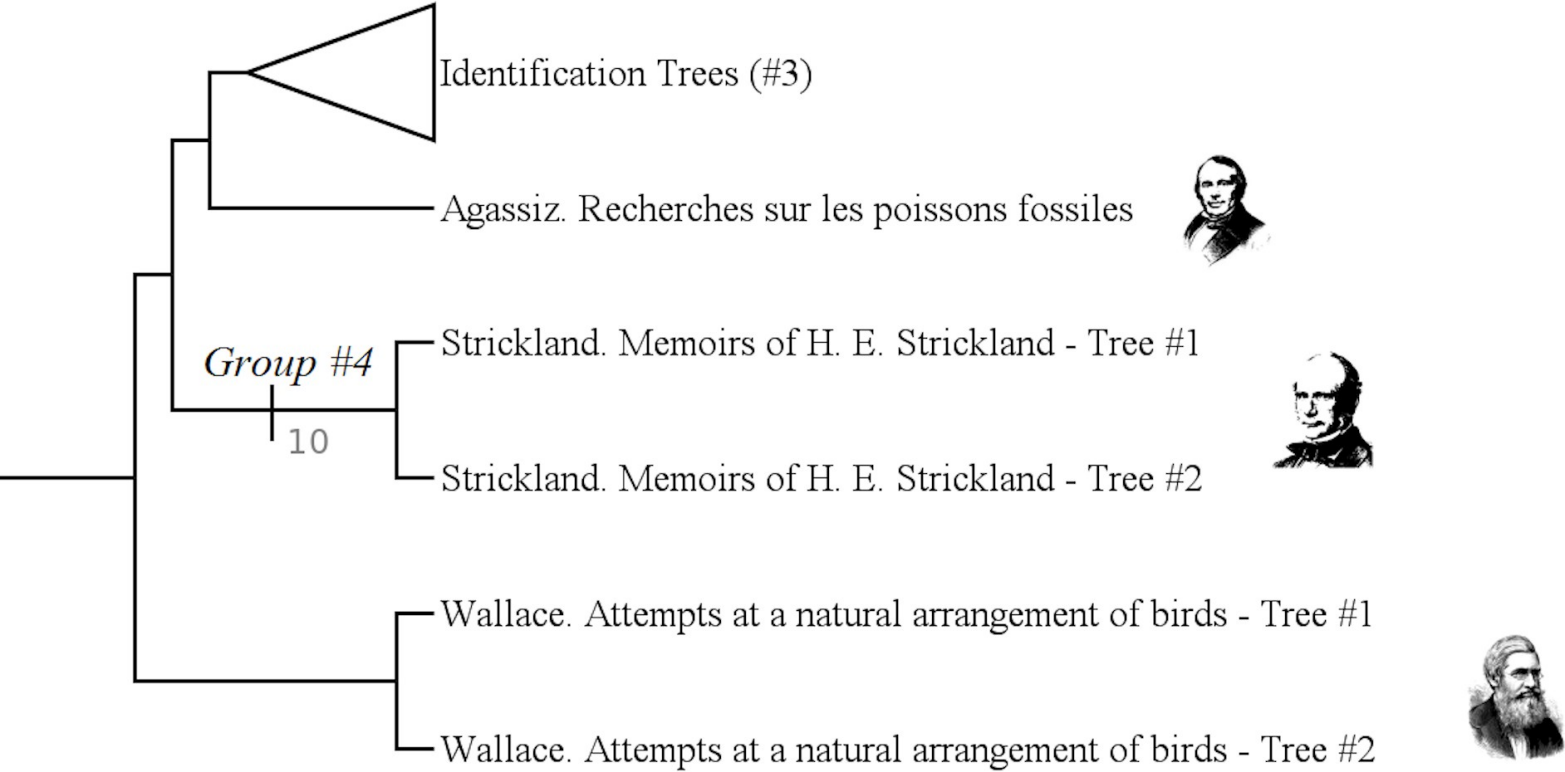
Subtree showing the group #4 (called “Strickland's “maps”).

The group #4 is the group of “Strickland's « maps ». It only includes Strickland (Fig 15).

It has a Bremer index of 10 and is supported by 10 characters:

Character #24 (Some branches show a change in shape: Yes);

Character #25 (What's on the branches is actually detectable: No);

Character #30 (Branches express a degree of similarity: Yes);

Character #46 (The leaves reconstruct the tree: Not only them);

Character #52 (Bubbles are covering the branches: Yes);

Character #66 (The diversification axis shows a similarity rate: Yes);

Character #86 (The ensembles have a practical vocation: possibly);

Character #88 (The tree gives the groups: Yes);

Character #90 (Reticulations ?: Yes);

Character #110 (Spontaneous creations ?: No).

In 1841, Strickland proposed a method aimed at classifying species according to their “affinity links”. This tree in not an evolutionist one: it does not consider any transformation in life.

He drew two trees: one, strictly theoretical, in his *“On the true method of discovering the natural system in zoology and botany”*, one classifying species in his “*Part of the Chart of the Natural Affinities of the Class of Birds”*. This last one is much more complex than the first one. “real” species are classified. But those two trees are here grouped.

#### Lamarck's trees (group #9)

The group #9 is the group of “Lamarck's trees”. It only includes Lamarck.

It has a Bremer index of 1 and is supported by 7 characters:

Character #31 (The branches show an evolutionary path: Yes);

Character #62 (The hierarchical axis shows a temporal order: Strict);

Character #81 (Entities are grouped because they have a common ancestor: No);

Character #89 (Scalist reading: Yes);

Character #97 (Classificatory ranks follow the tree graph ?: No), and this character has a CI of 0,5;

Character #106 (« Jumps » in the transformation?: No);

Character #110 (Spontaneous creations ?: Yes).

We have studied two of Lamarck's trees: one from his “*Histoire Naturelle des animaux sans vertèbres”*, one from his “*Philosophie Zoologique”*.

The first one is a “brackets tree”. While very different, they share a same manner to elaborate a classification.

#### Aesthetic trees (group #13)

**Fig 16:**
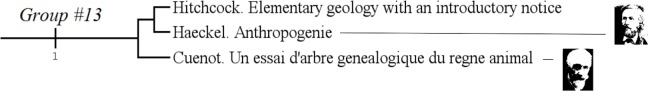
Subtree showing the group #13 (called “Aesthetic trees”).

The group #13 includes Cuénot's and Hitchcock's trees, and Haeckel's tree of mankind in *Anthropogenie*. (Fig 16)

It has a Bremer index of 1 and is supported by 20 characters:

Character #11 (The root is drawn like a real tree root: Yes);

Character #13 (The trunk shows an evolutionary direction: Yes);

Character #17 (The trunk has an aesthetic intention: Yes);

Character #19 (The trunk is thick: Yes);

Character #27 (Branches have an aesthetic significance: Yes);

Character #28 (Branches are drawn as real tree branches: Yes);

Character #32 (The branches show affinity links: No);

Character #44 (The leaves have an aesthetic impact: Yes);

Character #45 (The leaves are thick: Yes);

Character #51 (Bubbles enable to visualize similar ecosystems: Yes);

Character #74 (The ancestor can be detected effectively: No);

Character #77 (Special place for mankind: Yes);

Character #89 (Scalist reading: Yes);

Character #105 (Finalism, direction in changes?: Yes);

Character #108 (Evolution rates are different among times ?: No);

Character #111 (Can a form be reached by several different bloodlines at the same time?: Yes);

Character #115 (Expressed Relations: Living environment: Yes);

Character #117 (Considered character: Intelligence: Yes);

Character #121 (The tree worths for all life: Yes);

Character #127 (The tree has an asthetic reach: Yes).

Three trees are here grouped. They both have a strong aesthetic goal: intended for a general public, they are either drawn as “real” trees, or richly coloured. Branches are thick, and a main direction in evolution is given: a finalism that leads to mankind.

#### Unnamed (group #19)

**Fig 17:**
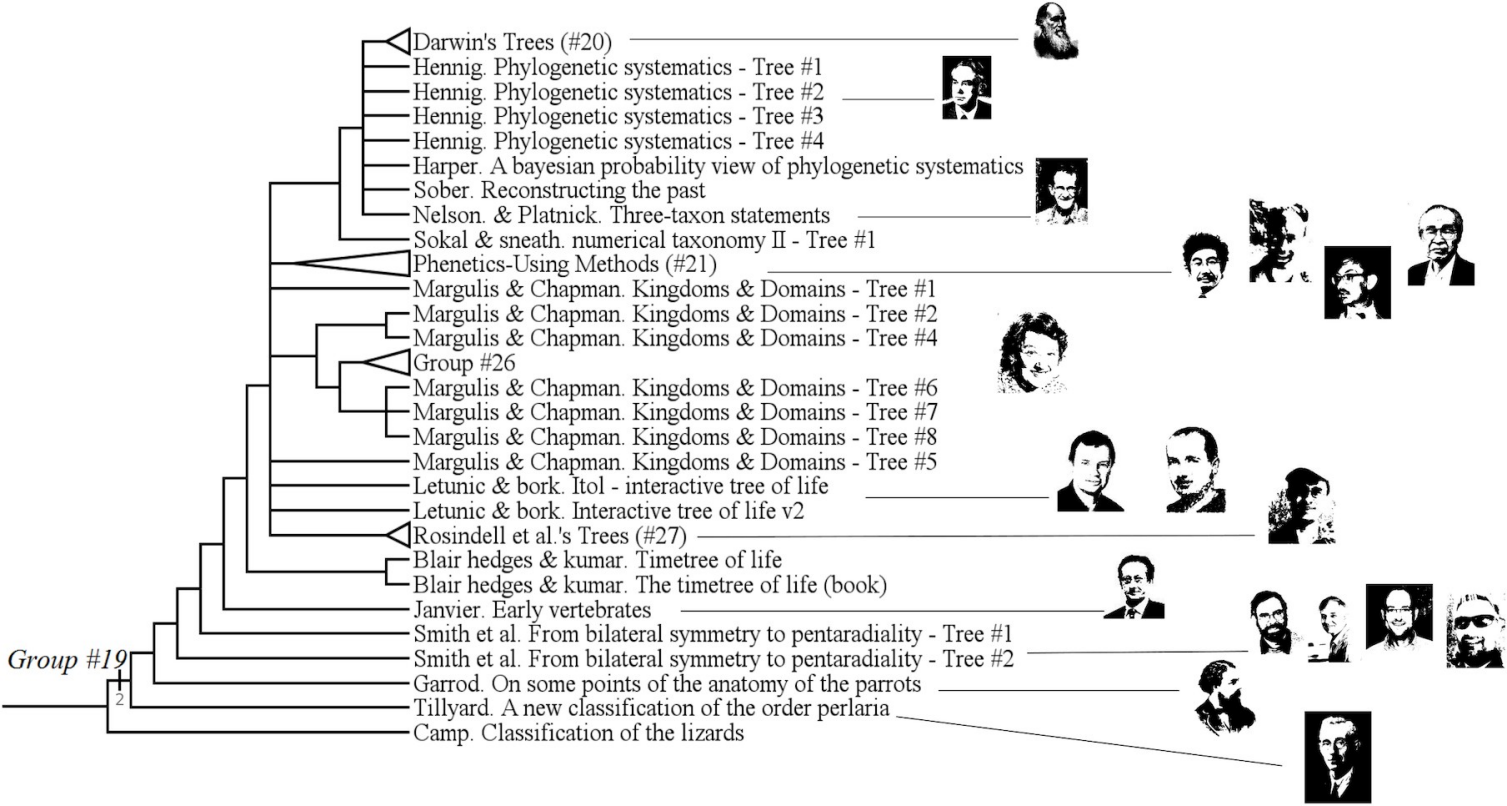
Subtree showing the unnamed group #19.

The unnamed group #19 includes authors like Garrod, Tillyard or Darwin. (Fig 17)

It has a Bremer index of 2 and is supported by 9 characters:

Character #10 (The root is a reconstruction, a hypothesis: Yes);

Character #80 (Entities are grouped because they share a typical form: No);

Character #83 (We test the groups: Yes);

Character #101 (Justification of the tree representation?: Yes);

Character #114 (Expressed Relations: Result of a Calculus: Yes);

Character #135 (Characters used: Arbitrary choice);

Character #136 (Outgroup?: Yes);

Character #140 (Use of algorithms: Yes);

Character #141 (Formalization of characters?: Yes).

Those trees are grouped by several characters detailed above, but also by a strong temporal consistency: from 1859 to nowadays. We notice the appearance of mathematical methods for the elaboration of the tree, whatever those methods can be. Those authors do “nominalist” classifications: they do not classify idealistic typical forms, but species. Moreover, groups are tested.

There is here a reversion with Darwin for characters #136, #140 and #141. Indeed, Darwin did not use any outgroup, nor any algorithms, nor any formalization of characters. But his trees share other characteristics with the others authors of this group.

#### Distances-methods users (group #21)

The group #21 is the group of “Distances-methods users”. It includes authors like Spooner & Ritchie, Sokal & Sneath and Hul Thol.

It has a Bremer index of 10 and is supported by 20 characters:

Character #3 (The root refers to a supposed living being: No);

Character #30 (Branches express a degree of similarity: Yes);

Character #32 (The branches show affinity links: Yes);

Character #36 (The nodes correspond to a percentage of similarity based on the characters: Yes), and this character has a CI of 1;

Character #37 (The nodes correspond to an ancestor: No);

Character #40 (The entity located at the nodes is reconstructed: No);

Character #41 (The leaves contain individuals: No);

Character #46 (The leaves reconstruct the tree: No);

Character #60 (The hierarchical axis shows discretised properties: No);

Character #61 (The hierarchical axis shows a similarity rate: Yes);

Character #79 (Entities are grouped because they have points in common: No);

Character #81 (Entities are grouped because they have a common ancestor: No);

Character #113 (Expressed Relations: Genealogy: No);

Character #121 (The tree worths for all life: No);

Character #124 (The tree has a Theoretical reach: Yes);

Character #128 (The tree topology is reconstructed: No);

Character #131 (The tree must follow overall similarity: Yes);

Character #132 (How is the time taken into account in the tree?: No time), and this character has a CI of 0,4;

Character #134 (Characters states used: All characters states);

Character #138 (Selection criterion of the tree:overall likelihood: Yes).

Among *Distances-methods users*, we find classical pheneticists: Sokal and Sneath (Fisler and Lecointre, [7]: node 68), Saitou & Nei … The *Minimum Evolution* method chooses the tree with the smallest sum of branch lengths. As *minimum evolution* uses distances methods, it also belongs to the *Distances-methods users* group.

#### Theoreticians of Phenetics (group #23)

**Fig 18:**
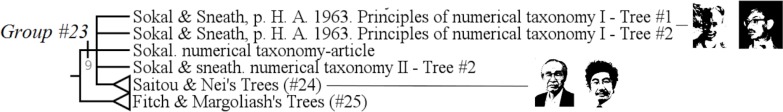
Subtree showing the group #23 (called “Theoricians of Phenetics”).

The group #23 is the group of “Theoreticians of phenetics”. It includes Sokal & Sneath (“pheneticists” of Fisler and Lecointre [7]: node 68) or Saitou & Nei. (Fig 18).

It has a Bremer index of 9 and is supported by 4 characters:

Character #98 (The tree aims at presenting the order of Nature: No), and this character has a CI of 0,5;

Character #102 (The tree is a simplification of reality: Yes);

Character #118 (Classified Objects: Species: No);

Character #123 (The tree has an Epistemological reach: No).

Among pheneticians, some authors have specifically worked on the theoretical foundations of this method. We have studied three of them: Sokal & Sneath, Saitou & Nei, and Fitch & Margoliash. They are here grouped. The characters #118 and #123 insist on the fact that those authors realize theoretical trees.

#### Theoretical genealogies (group #33)

**Fig 19:**
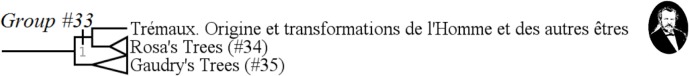
Subtree showing the group #33 (called “Theoretical genealogies”).

The group #33 is the group of “Theoretical genealogies”. It includes Trémaux and Rosa. (Fig 19)

It has a Bremer index of 1 and is sustained by 8 characters:

Character #58 (The hierarchical axis shows the diversity of the shapes: No);

Character #72 (The ancestor shows an order of appearance: No);

Character #93 (Elaboration of "complete" groups: Yes, as a result of a classification program);

Character #101 (Justification of the tree representation ?: Yes);

Character #103 (Classificatory purpose: No);

Character #121 (The tree is worth for all life: Yes);

Character #123 (The tree has an Epistemological reach: No);

Character #124 (The tree has a Theoretical reach: Yes).

We find here two authors: Trémaux and Rosa. Like Darwin's (1859) one, their figures are strictly theoretical ones: they present the way that a classification should be if the theory of the author is true. But their theories of evolution and classification of species are different from Darwin's one. Trémaux develops a theory of the evolution of species based on the type of soil on which they live. On the other hand, Rosa's theory of evolution is based on the idea of an asymmetry of the evolutionary rates. This is why Darwin is not grouped here.

#### Unnamed (group #44)

**Fig 20:**
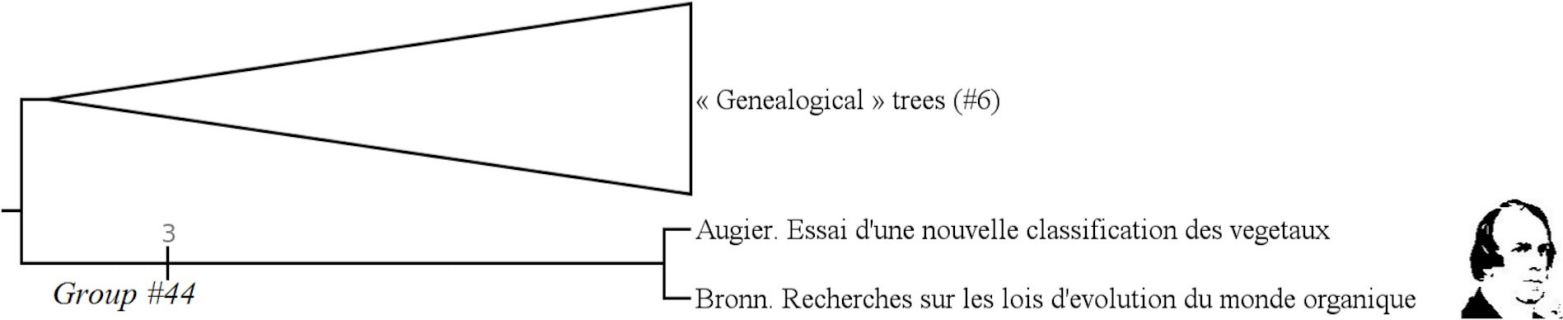
Subtree showing the unnamed group #44.

The group #44 is the group of “trees for showing continuities and discontinuities in Nature in a fixist framework”. It only includes Augier and Bronn. (Fig 20)

It has a Bremer index of 3 and is supported by 9 characters:

Character #11 (The root is drawn like a real tree root: Yes);

Character #15 (Trunk shows the succession of "evolutionary grades": No);

Character #26 (What's on the branches still exists today: No);

Character #27 (Branches have an aesthetic significance: Yes);

Character #28 (Branches are drawn as real tree branches: Yes);

Character #54 (The hierarchical axis shows an interlocking of the groups: Amongst other things);

Character #100 (Justification of the classificatory system ?: Yes);

Character #101 (Justification of the tree representation ?: Yes);

Character #127 (The tree has an aesthetic purpose: Yes);

The question of continuity or discontinuity in Nature is a main question in the 18th–early 19th centuries. The trees grouped here have been drawn not to show an evolution–or a transformation–in life, but to illustrate both continuities and discontinuities in Nature.

## Discussion

The rooted non-cyclic connected graph (the «rooted tree ») provides a hierarchy and creates nested categories of ideas about the published trees. However, to interpret such a graph in a historical manner, one must keep in mind that ideas do circulate in various ways. They can be « inherited » vertically when an author integrates the idea of direct predecessors. They can be « horizontally » transmitted when an author discusses with (or reads) another contemporary author. Finally, they can also appear convergently and independently at the same times and even at different times. Therefore two author’s trees can be sister-groups for all these different reasons while it has to be noted that the tree is optimized in a way where convergences that do occur cannot be subject to other optimization options.

### Significance of treeness

The high values of CI and RI mean that a majority of characters change consistently along the tree of trees, imposing a relatively strong hierarchical structure. In other words, the contradicting character changes (homoplasies) being minor, we get a rather high level of tree resolution, and relative high degree of hierarchy in character states distribution (RI of 0.83) which cannot be explained by other theoretical model than vertical transmission. The difference between RI and HER is low (0.09), indicating that part of characters that appear as homoplastic do not change jointly, but rather in a random manner. Low CI and RI are well explained by a mix of horizontal diffusion and vertical transmission and/or high rates of change while the high values of CI and RI reflect dominant vertical transmission. In an interesting study, Nunn et al. [4] simulated horizontal and vertical transmission and showed that a RI above about 0.60 is indicative of a high degree of vertical transmission and a low degree of horizontal transmission. This result is consistent with a previous study by Collard et al. [54] who compared the RI of biological data with the one of cultural data. They showed that the average RI of 21 sets of biological data was 0.61, while the average RI of cultural data was 0.59. This is also consistent with other studies analysing cultural data with the same methods [55]. Therefore, the hierarchical structure of our present data as measured through RI supports the hypothesis that the sharing ideas about biological trees among naturalists could have been vertically inherited. Since the RI does not equal to 1, there must also be a certain amount of horizontal diffusion, convergence or borrowings. The rather good treeness (0.26) of our tree of trees suggests that most of the similarities in ideas about biological trees must have been globally vertically inherited,

### Circulation of ideas about trees

Although most of the characters we describe appear to be “inherited” (vertically transferred), some of them appear to be horizontally circulating (RI < 1). With a hand-analysis, it is possible to shed light on exchanges between authors onto a rooted tree. To establish them, the date of publication of a given tree is necessary information. When members of a same group or clade are of the same period, it suggests that the authors shared at least some ideas of their times or might have read the same sources. When two OTUs (two biological trees) are sister-groups but published at different centuries, it suggests that the recent author has borrowed at least some ideas from the more ancient sister-author. The dates of publication offer to the historian some indications on the tracks to follow by looking back and read authors of the past to check for possible borrowings. Moreover, when characters changes occur more than once in the tree by convergences or reversions (homoplastic events) the published dates of trees can indicate the direction of changes and hence the direction of the circulation of ideas, as authors cannot read ideas not yet published. Let’s take the example of characters 41 and 55.

**Fig 21:**
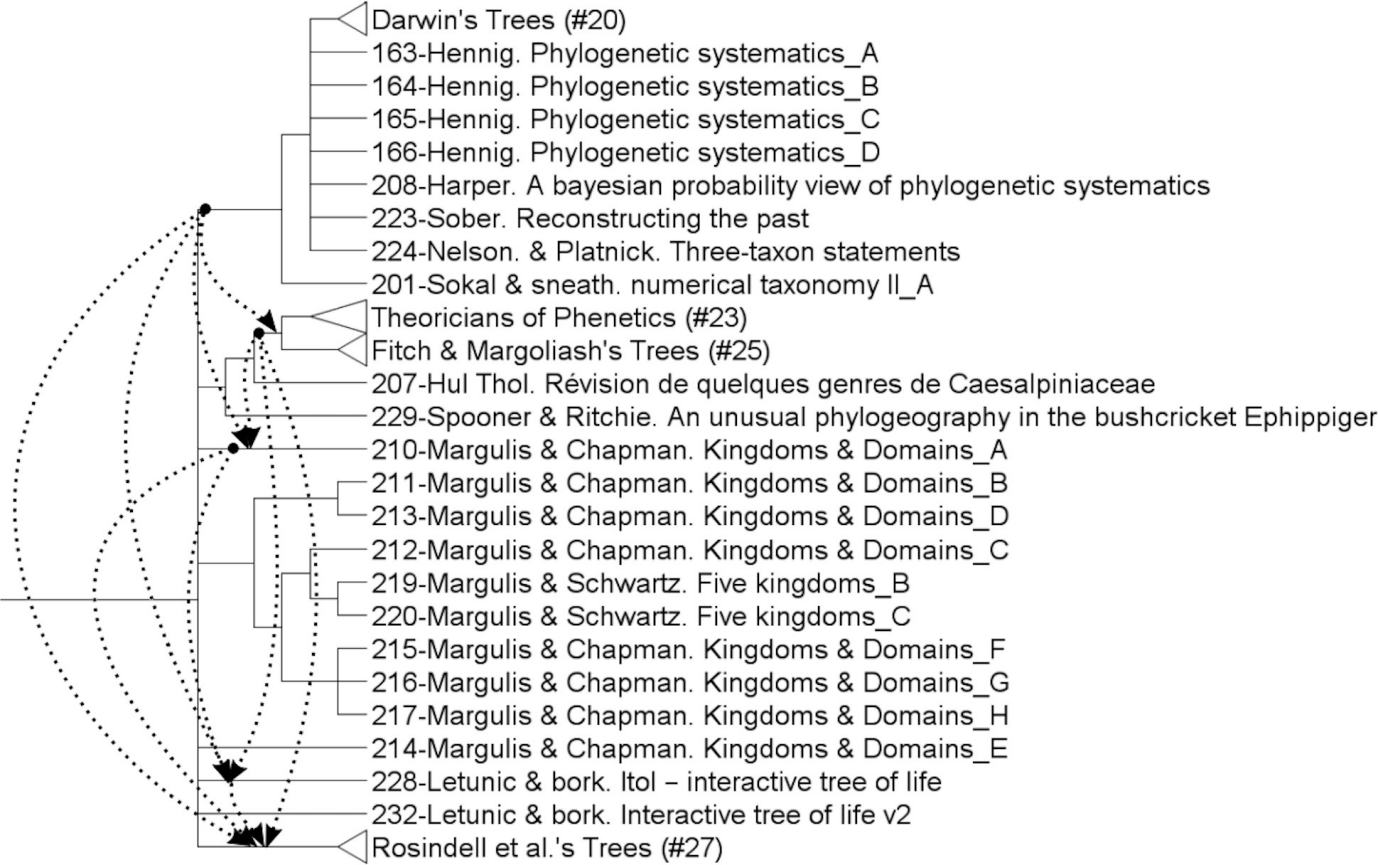
Circulation of the character #41: *“The leaves contain individuals”* (see text).

The character #41 (The leaves contain individuals, Fig 21) is generally associated with a formalized classification, as are cladist ones (since 1966). However, non-cladist authors (pheneticians or Margulis & Schwartz, tree #210) have proposed to classify individuals as leaves slightly before (1963). As a result, arrows start from the branch of, or the subtree containing, the oldest tree -here, the group containing Darwin's tree—to other ones containing this idea. This is repeated for each convergent occurrence of the character, from the oldest branches to the youngest ones. This is the reason why arrows also start from the node containing groups #23 & 25 and from the tree #210 as well. These arrows are hypotheses of transfers to be tested by further historical analyses.

**Fig 22:**
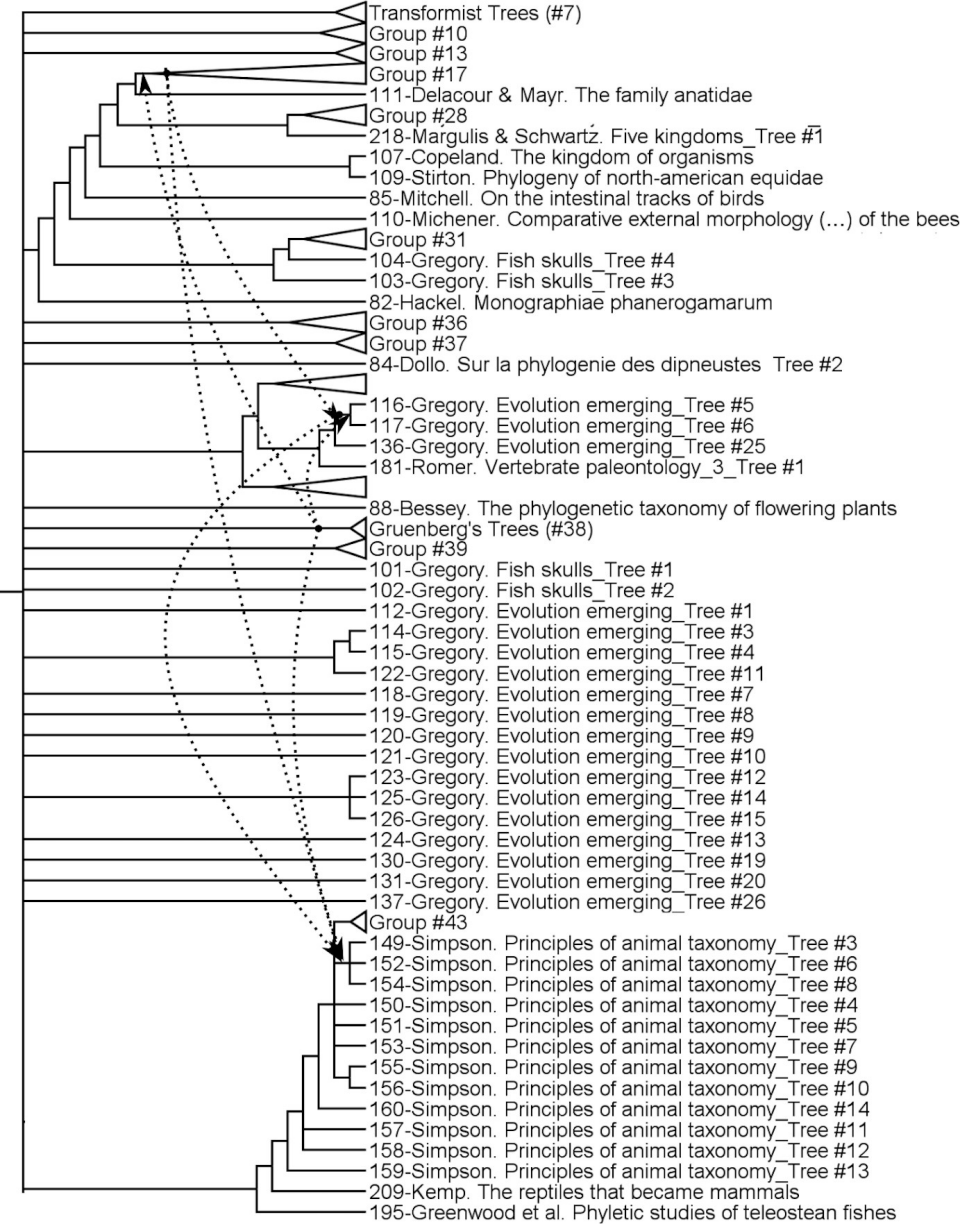
Circulation of the character #55: *“The hierarchical axis shows a succession of groups”* (see text).

The second character (#55: The hierarchical axis shows a succession of groups, Fig 22) is found in both evolutionary trees (e.g. Romer, Haeckel, Simpson) and non-evolutionary trees (Wallace's trees of birds). It is a succession of groups along the hierarchical axis. It starts from the group containing the oldest tree (Group #38) and goes to non-related trees (Gregory, 116–117; then to Simpson (149, 152, 154), then to group #17).

Each of these non-related trees, in turn, transfers to each other the same idea from the oldest to the youngest. For instance, this idea goes from Group #17 (which contains Darwin) to Simpson and Gregory.

### Is there any phylogeny in all this?

It is not required to interpret our rooted hierarchical non-cyclic connected graph as a phylogenetic tree *a priori*, but it can be interpreted as such *a posteriori*. As the treeness is rather good, it is not forbidden to interpret such a “tree” as a phylogenetic tree. There are nevertheless differences between a biological phylogenetic tree and a phylogenetic tree of ideas. In biology, the time frame of character transmission is embedded within the time span of organism’s life. An organism cannot inherit directly from its grand-grand-grandparents. Passing characters from one generation to the next one, sometimes with changes, is one of the facts founding the theoretical tree of life (Darwin’s « *descent with modification* »). In a phylogenetic tree of ideas, the time frame of character transmission is fully open, given the restriction that an author of the past cannot read an author of the future. Theoretically it is possible that an author discovers another unknown one dating back from centuries ago and uses his ideas. This « jump in time » makes possible–and even normal- that the topology of our tree does not necessarily follow the time of the published trees used as OTUs. In other words, it is possible that two authors of very different times are closely related to one another. So, if the present tree can be considered as phylogenetic because its good treeness, such possibilities of “jumps in time” disrupts the time frame of the classical phylogenetic model. Such jumps are not so frequent here, but it could be different with another matrix of other ideas. They seem to disrupt the time frame of the phylogenetic model as applied in biology, but do not affect the legitimacy of the method. The main point is transmission with modification (whatever the time frame), and this is what ideas are subject to.

Whatever the legitimacy of interpreting a tree of ideas as a phylogenetic tree, the tree can be best used to create nested categories from the most robust clades. The circulation of ideas can be better studied in the split network (Fig 4), which carries no idea of phylogeny (as a matter of fact it is unrooted and ancestors cannot be reconstructed). In that kind of representation the strength of the global resemblance between ideas about two different trees is represented by the length that connects them. The shorter the length, the more similar the ideas are, the uncertainties being represented by the multiple possible paths joining them. A given character is mapped onto the network. Then the circulation is indicated using the shortest paths from one character state to its other occurrence(s) with the direction indicated by the dates of publication attached to the tip of the branches (OTUs). However, such a network representation is no longer a phylogeny since a network is unrooted and the ancestors not inferred. Hence, only trees allow to build several hierarchical categories in the different representations of the history of life made over time, helping to formulate new hypotheses and to open new perspectives in the field of the history of science. In building some categories through these comparative and quantitative approaches, we hope to improve testability of categories used to group thinkers in this field. Since the data processing methods used here for analyzing our data have proven to be rather successful, they deserve to be tested on other data as well, although it is never guaranteed that the results will be as good.

## Appendix

### Annex 1: List of historical trees coded

Conrad Gesner

1516 (Zurich, Switzerland) - 1565 (Zurich, Switzerland)

After first theology studies, Gesner went to France to study medicine and ancient languages. There, he learned botany, zoology and wrote many works about it.

We study the third volume of his book *Historiae Animalium* (or "History of Animals"), published in 1555. This is a large four-volume work written in Latin. A tree is drawn page 582. It is a "determination key", which allows both to identify a specimen and to classify species.

Matthias De L'Obel

1538 (Lille, France) - 1616 (London, England)

Fascinated since childhood by botany, De l'Obel studied this discipline. Rondelet is one of his teachers. As an eminent botanist, De l'Obel made several trips to study plants.

We studied his main book, *Plantarum seu Stirpium Historia*, published in 1576. Trees are drawn on pages 69 and 94–95. Those are still "determination keys" with thin leaves, branches, roots and nodes.

Adam Zalužanský ze Zalužan

1558 (Mnichovo Hradiště, Bohemia) - 1613 (Prague, Bohemia)

As a doctor of the University of Prague, Zaluziansky taught Ancient Greek. He was also a doctor in medicine and knew poetry, pharmacy, medicine, religion.

We studied the second of the three volumes of his book *Methodi herbariae Libri Tres*. Zaluziansky classifies plants according to their "natural" links. There are many trees in the book. These are, again, "determination keys" that classify plants to facilitate their identification. The three trees we selected appear on pages 109, 136 and 202 of the book.

Robert Morison

1620 (Aberdeen, Scotland) - 1683 (London, England)

After escaping from Scotland during the 1644 civil war, Morison studied medicine in France, where he discovered botany. He managed the botanical gardens in Blois, then taught botanics in Oxford. There, he continued his research and proposed a new classification of plants inspired from Cesalpino's ideas and based on the attributes of flowers and seeds

We studied his book *Plantarum Umbelliferarum distributio nova*, published in 1672. Three trees were selected: they are located pages 15, 28 and 218.

Martin Lister

1639 (Radclive, England) - 1712 (Epsom, England)

After studying medicine in France, Lister practiced in England. There, he began to work on invertebrate zoology. He described, classified and discussed their development. His rich collections were exhibited in his cabinet of curiosities.

We studied his *Historiae animalium angliae*, published in 1678. There Lister draws three trees. The first of these charts, p. 19, is a classification of spiders. Lister organized this classification following the shape of their eyes. The second shaft, p. 110, classes gastropods following their environment and shell type. The third, p. 154, is a finer classification of marine shells according to their shell type.

Francis Willughby

1635 (Middleton Hall, England) - 1672 (Middleton Hall, England)

It is during his studies at Trinity College, Cambridge that Willughby met John Ray. The two friends travelled through Europe in order to study animals and plants. But Willughby's precocious death prevented the publication of his own works. It is thanks to Ray that they were published.

We studied his *De historia piscium libri quatuor*, published in 1686, particularly the tree drawn page 63. It is a classification of fishes based on the properties of their bodies.

John Ray

1627 (Black Notley, England) - 1705 (Black Notley, England)

John Ray discovered and started to study botany as a child. Later, he taught botany at the Trinity College of Cambridge. But he opposed in 1662 to the Act of Uniformity and was then dismissed. He accompanied his friend Willughby during his expeditions: the latter studied zoology, and Ray botany.

We studied here Ray's *Synopsis methodica* (1673), a description and classification of animals. On page 53, a tree is drawn. This is a “keys” tree that classes animals.

Carolus von Linnaeus

1707 (Stenbrohult, Sweden) - 1778 (Uppsala, Sweden)

Swedish physician and botanist, Linnaeus revolutionized theories and classificatory practices. He also introduced the concept of "binomial nomenclature."

His major work is his *Systema Naturae*. We found there two trees. The first one is drawn in the fourth part of the first edition of his *Systema Naturae* (1735), p. 1520. It is a key to the insects, prior to their description in the following pages. The second tree is drawn in the tenth edition of *Systema Naturae* (1758), p. 837. It is a classification of plants according to their reproductive organs.

Jacob Theodor Klein

1685 (Königsberg, Germany) - 1759 (Gdansk, Poland)

After law studies, Klein studied Natural History. After a travel to Europe, he made his career in politics and created his own botanical garden and cabinet of curiosities.

We studied the trees of two books of Klein.

The *Historiae piscium naturalis* (1742) is a classification of fishes. Three trees were selected: they are on pages 7 and 9 of the second part and 6 of the fourth part of his book.

In his *Tentamen Methodi ostracologiae* (1753), Klein draws two trees: one classifying different shells following their shape, and the other being an identification key of the different snakes and legless vertebrates.

Georges Louis Leclerc, Comte de Buffon

1707 (Montbard, France) - 1788 (Paris, France)

Intendant of the *Jardin du Roi* (King’s Garden, now called the *Jardin des Plantes* in Paris), Buffon accomplished there his major work: the "*Histoire Naturelle*, *Générale et Particulière*" (Natural History, General and Particular) in thirty six volumes (including seven supplements).

In the fifth volume of this work, Buffon presented "The dog with its varieties." Pp. 258–259, a figure is drawn. It reconstructs the history of the different dog varieties. It is both a tree and a map. It is a tree because it has a root—the sheepdog, in the centre of the figure—but also nodes, branches and leaves. The reading direction goes from the root to the nodes, and leaf nodes. Crosslinks are also present. But this figure is also a map. A compass is drawn: it shows the north. It is to be compared to the description given by Buffon in the text: this compass illustrates the directions of the migrations—north, east, south or west–that led to the present varieties of dogs.

Mathurin-Jacques Brisson

1723 (Fontenay-le-Comte, Vendée, France) - 1806 (Magny-les-Hamlets, France)

After theology studies, Brisson discovered Natural History. His meeting with Réaumur was decisive, and they started to work together. But the death of Réaumur deprived Brisson from his protection. He then turned away from Natural History to focus on physics.

We studied the tree located on pages 12–13 of his book *Le règne animal divisé en IX* classes (the animal kingdom divided in 9 classes). This tree is both a classification and key to the quadrupeds. To do this, Brisson uses highly visible characters: presence or absence of teeth, type of legs, tail or fingers shape, etc. It separates quadrupeds in orders, each corresponding to a part of his work.

Etienne Louis Geoffroy

1725 (Paris, France) - 1810 (Chartreuve, France)

Physician, Geoffroy after his graduation reoriented his studies to zoology. He wrote several books on animals: insects (1762), gastropods (1767) and vertebrates (1778) and medicine.

We studied the second volume of his book *Histoire abrégée des insectes*, *dans laquelle ces animaux sont rangés suivant un ordre méthodique* (Abbreviated History of Insects, in which those animals are placed in a methodical order, 1762). He drawn there a tree to classify insects, and proposed new classification criteria as wings (presence, number, texture, shape …) or tarsus.

Antoine-Nicolas Duchesne

1747 (Versailles, France) - 1827 (Paris, France)

As a Buffon's disciple, Duchesne was fascinated by Natural History. He grew strawberries near the Château de Versailles.

We studied his *Histoire Naturelle des Fraisiers* (Natural History of Strawberries), published in 1766. The book is in two parts. In the first one, Duchesne studies the plant, its varieties and hybrids. He himself grew some at Versailles. In the second part of the book, he describes the culture and maintenance of these plants. Duchesne drew a tree classifying strawberries after their genealogy. This classification follows Buffon's idea of degeneration.

Antoine Gouan

1733 (Montpellier, France) - 1821 (Montpellier, France)

Doctor in Medicine, Gouan discovered Natural History there and devoted his career to it. He first studied zoology and, since 1762, botany.

We studied his *Histoire des poissons* (history of fishes, 1770). This book is written in two languages: French (even pages) and Latin (odd pages). The author describes there the anatomy of fishes. At the middle of the book is drawn a "clé des classes" (Key to the classes). This is a tree diagram. Animals are organized into classes according to their gills and then to the arrangement of their fins.

Eugen Johann Christoph Esper

1742 (Wunsiedel, Germany) - 1810 (Erlangen, Germany)

After studying theology and philosophy, Esper discovered and taught Natural History at the University of Erlangen (Germany).

We studied *Die Schmetterlinge in nach der Natur mit Abbildungen Beschreibungen* (Butterflies after nature pictures with descriptions, 1777). It is a multivolume description of butterflies found in his country. This description follows the Linnaean method. In this first volume of the work, he drew a tree-like arrangement of butterflies.

Charles De Geer

1720 (Rinsinge, Sweden) - 1778 (Stockholm, Sweden)

Since childhood, De Geer was interested in entomology. In addition to his political and industrial activities, he conducted research in Natural History.

We studied the seventh volume of his *Mémoires pour servir à l'histoire des insectes* (Memoirs for the history of insects, 1778), his main work. There are described and drawn a large amount of insects so as to allow their identification. At the end of the seventh volume of this book, there is a classificatory tree that classifies insects according to their types of wings, and if there are no such, according to their metamorphosis.

Peter Artedi

1705 (Anundsjö v (Sweden)) - 1735 (Amsterdam, Netherlands)

Artedi was one of the closest friends of Linnaeus. They met at the University of Uppsala. But Artedi accidentally precociously died in Amsterdam. Linnaeus published his work posthumously.

We studied his *"Genera Piscium"* ("fishes types", in. Ichththyologiae pars. III, 1792). The tree immediately follows the preface. It is a "determination key". Artedi distinguished what we now call "cetacea" (*"plagiures"*) from the rest of the "fishes" by the position of their fins.

Augustin Augier (1725–1825)

We have no information about Augustin Augier. The only information that reached us about him is that he lived near Lyon (France). We only know one work from him: his *Essai d'une nouvelle classification des* végétaux (Essay of a new classification of Plants). It is a tree-shaped classification of plants. But the tree is definitely not a genealogical one: it combines the discontinuity and continuity between the various forms of plants.

Jean-Baptiste de Monet, (chevalier) de Lamarck

1744 (Bazentin-le-petit, France)– 1829 (Paris, France)

Major naturalist in the History of Sciences, Lamarck is the author of both the term of "biology" and the first theory of a widespread transformation of animals.

We studied his masterpiece, his *Philosophie zoologique* (Zoological Philosophy).

In the first part of his work, he describes the mutations of bloodlines among generations. Lamarck's theory is the first generalized tranformism. Evolution–a word that Lamarck never uses, employing the term of “chaîne animale” (animal chain)–is seen as a mutation with an increasing complexity of the organs of animals.

In the text, Lamarck describes his concept that the only possible classification of life is onto a scale of beings. But, in the addenda, he draws a tree illustrating the successive states of bloodlines.

Charles Hélion de Barbançois-Villegongis

1760 (Villegongis, France) - 1822 (Villegongis, France)

Barbançois is known as an experimenter breeder and an agronomist. He published numerous books and articles from his studies and experiments.

We studied two articles written by Barbançois. Both were published in 1816 and followed Lamarck's ideas. The first one is his *"Observation sur la filiation des animaux"* (Observation on the parentage of animals). In this short paper, he more thoroughly investigates the succession of beings that he organizes them onto his own tree. Barbançois makes some modifications in Lamarck's classification. The tree produced by Barbançois is much richer in details than Lamarck's one.

The second tree drawn by Barbançois is found in his article "*Observations pour servir à une classification des animaux*" (Observations for use in a classification of animals). He firstly redefines there Lamarck's own classification. Then, Barbançois defines different subclasses within each class previously defined. The tree then drawn is a succession of subdivisions in the group of animals: vertebrates and invertebrates; vertebrates with a visible or en invisible nervous system, etc.

Hugh Edwin Strickland

1811 (Reighton, England) - 1853 (Retford, England)

After studying geology, Strickland participated to a scientific exploration in Asia and Europe. He wrote several books to synthesize his discoveries and became a recognized geologist. But he died hit by a train in 1853.

We studied three trees by Strickland. The first one appears in his article "On the true method of discovering the natural system in zoology and botany" (1841). This tree illustrates his methodology to classify species according to their affinity relationships.

The second tree appears in his 1843 article: "Part of the Chart of the Natural Affinities of the Class of Birds." It is an implementation of the method presented in the article above.

The third and final tree appears in his article "Map of the family Alcedinidae" (1843). This is again an implementation of its classificatory system proposed in 1841 to classify kingfishers

Louis Agassiz

1807 (Motier en Vuly, Switzerland) - 1873 (Cambridge, Massachusetts)

Ichthyologist and glaciologist Agassiz taught and popularized Natural Sciences and realized several scientific expeditions.

We studied his *Researches on Fossil Fish* (1843). The tree (p.170) represents fossil fish from their scales. But Agassiz was not an evolutionary biologist, and imagined periods of appearance and disappearance of species by supernatural causes. This is translated by spaces between groups.

Robert Chambers

1802 (Peebles, Scotland) - 1871 (St Andrews, Scotland)

Self-taught, Chambers was the editor and author of several books on various subjects.

We studied Chamber's *Vestiges of the natural history of Creation* (3rd edition, 1845). This work is Chamber's best known. Published anonymously, this book, written for the general public, offers ideas about the history of the formation of the world and life. The tree is drawn p. 176 represents a progressive succession of forms: fish, reptile, bird and mammal).

Heinrich Georg Bronn

1800 (Ziegelhausen, Germany) - 1862 (Heidelberg, Germany)

Bronn taught Palaeontology at the University of Heidelberg and works during his career on this subject. His major work is his *Letkaea Geognostica*.

We studied his article "*Recherches sur les lois d'évolution du monde organique pendant la formation de la croûte terrestre*" (Researches on the laws of evolution of the organic world during the formation of the Earth's crust). It is an answer to a question put on competition in 1850 by the French Academy of Sciences.

Researchers had to elaborate a classificatory system suitable for all geological periods. Bronn won the prize in 1856. The system he elaborated is not an evolutionary one, but is based on times of extinctions and creations. Bronn drawn a classificatory tree to illustrate his conclusions (p. 900).

Edward Hitchcock

1793 (Deerfield, Massachusetts) - 1864 (Amherst, Massachusetts)

After theology studies, Hitchcock performed his first scientific career in astronomy. He then redirected to the Natural Sciences, teaching chemistry and theology and studying geology.

We studied the eighth edition of his *Elementary Geology*. This popular book presents the geological history of the Earth and the fossils found in different geological strata.

On the second page of the book is drawn a large colourful figure: two trees are facing. The left one shows the plant kingdom, the right of the animal kingdom. The graph itself is entitled "*Paleontological Chart"*. A crown is placed on a group of each tree. On the tree of animals, this is the man which is thus crowned. For plants, it is the palm.

Alfred Russel Wallace

1823 (Usk, Wales) - 1913 (Broadstone, England)

Naturalist explorer, Wallace is known to be Darwin's co-discoverer of the theory of natural selection. This author, in his evolutionary texts, also proposed to classify species on a tree, but this tree had never been drawn.

Yet Wallace drew two trees in his article "*attempts at a Natural Arrangement of Birds*". They are very different as affinity trees. Each aims at classifying a group of birds. The first is p. 205, the second p. 215.

Charles Darwin

1809 (Shrewsbury, England) - 1882 (Downe, England)

Key author in biology, Darwin is famous for his ideas about the origin of species, their classification and natural selection.

In his “*Origin of Species”*, Darwin draws a strictly theoretical tree: theoretical, because it is not a classification on tangible species, but a conjecture about the form to be taken by the classification if his theory is correct.

We studied the trees drawn by Darwin in the first (1859) and sixth (1876) editions of his *Origin of Species*.

John Denis Macdonald

1826 (Cork, Ireland) - 1908

Member of the Royal Navy and fond of Natural History, Macdonald embarked in a large scientific expedition to the Pacific. His descriptions and drawings brought him honours from his peers.

We studied his article *"Further Observations on the Metamorphosis of Gasteropoda*, *and the Affinities of some Genera"* (1862). It comes from his observations during his mission in the Pacific. Macdonald studied anatomy of molluscs that class then in a tree. But this tree is not an evolutionary one: it is composed of three independent figures which are located on the same page. These are series of dichotomies.

Pierre Trémaux

1818 (Charrecey, France) - 1895 (Tournus, France)

Former student of the Fine Arts School in Paris, Trémaux performs two journeys to the North and the East of Africa. There he made many photographs, drawings and maps.

We studied his scientific work *"Origine et transformations de l'homme et des autres êtres"* (Origin and transformation of mankind and other beings", 1865). Trémaux sees a link between the perfection of a species and the quality of the soil on which it lives. Thus, evolution is does not follow an increasing complexity but depends on geological causes.

Trémaux draws a single circular tree (p.272). It has four main roots: each one corresponds to one of the four *types* recognized by Blainville (amorphozoa, actinozoa, malacozoa and zygozoa). The classification is genealogical.

Albert Gaudry

1827 (Saint Germain en Laye, France) - 1908 (Paris, France)

French paleontologist, Gaudry is an evolutionist author. He directed several scientific missions, particularly in Greece.

We studied his book "*Considerations générales sur les animaux fossiles de la faune de Pikermi*" (General Considerations on the fossil animals of Pikermi, 1866), made from the data of one of his missions. Several trees are drawn: these are reconstructions of genealogies of species. Fossils found in Pikermi follow one another in ancestors to descent links.

We kept five trees from this work. The first one (p.36) is a classification of Hyenidae; the second (p.38) of Elephantidae; the third (p.41) Rhinocerotidae; the fourth (p.44) of the Equidae and the fifth (p.46) of various mammals.

Ernst Haeckel

1834 (Postdam, Prussia)- 1919 (Iena, Germany)

Biologist, teacher and popularizer of Natural Sciences, Haeckel was a convinced evolutionist. He proposed a theory of recapitulation–the idea that the development of individuals, from embryo to adult, recapitulates stages of evolution of its lineage–and a monistic view of the development of life.

We studied the trees drawn in the three major works of Haeckel: *Generelle Morphologie* (General Morphology, 1866); *Anthropogenie* (Anthropogeny, 1874) and *Natürliche Schöpfungsgeschichte* (Natural History of Creation, 1868).

*Generelle Morphologie* presents eight trees.

The first one (p.463) is a "monophyletic family tree of organisms (*"*Monophyletischer Stammbaum der organismen *"*)". It presents a monophyletic classification of the three kingdoms of life: animals, plants and protists. Its trunk is thick, branches are sinuous and its texture is that of the bark.

The second (p.465) is a tree of plants (*"Stammbaum der Pflanzenreichs")*; Very close to the previous one, it distinguishes from this one by the shape of its trunk.

The third tree (p.467) is a tree of coelenterates (*"Stammbaum oder der Coelenteraten Acalephen (Zoophyten)"*). Its trunk is short and the tree on itself full of incomplete groups.

The fourth tree (p.469) is a tree of echinoderms (*"Stammbaum der echinoderms"*). Very different from the previous ones, it is a succession of geological eras.

The fifth tree (*"Stammbaum der Articulaten"*, p.471) is a tree of "Articulata"(arthropods and worms). It is very similar to the first item of the three trees.

The sixth tree (p.473) is a tree of snails (*"Stammbaum der Mollusken"*). Its trunk is vertical.

The seventh tree (p.475) is a tree of vertebrates (*"Stammbaum der Wirbelthiere"*). It shows, as the fourth tree, paleontological data.

The eighth tree (p.477) is a tree of mammals (*"Stammbaum der Säugethiere"*). It shows the group of mammals, in which mankind is set. But its place is a privileged one: at the very top right of the tree.

*Natürliche Schöpfungsgeschichte* shows sixteen trees, each being very different.

The first tree (p.444) is a monophyletic tree of organized beings. It represents the pattern of the diversification of life if all species were derived from a single ancestral form. Life is split in three kingdoms: animals, plants and protists.

The second one (p.445) is a polyphyletic tree of organic beings. Very similar to the one above in its structure, it presents the pattern of the diversification of life if its origin is polyphyletic.

The third tree (P.453) is a monophyletic tree of plants. Plant groups follow one another.

The fourth tree (p.483) is a monophyletic tree of plants drawn from paleontological data. Very different from previous, it follows the succession of paleontological times.

The fifth tree (p.489) is an historical explanation of the six animal tribes. It is part of a strict chronology, illustrated by the sequence of geological eras in the vertical direction of the figure.

The sixth tree (P.501) is a monophyletic tree of the animal kingdom. It is very similar to the fourth one, but we chose to keep it because of the place given to the vertebrates, placed at the top of the tree.

The seventh tree (P.511) is a tree of zoophytes. Those organisms, once considered as intermediate beings between plants and animals are set here at the basis of the tree of animals.

The eighth tree (p.517) is a tree of worms. Animals follow one to another in an order of complexity similar to that of Lamarck's tree.

The ninth tree (p.527) is a tree of snails. Molluscs suceed to worms. Their arrangement on the tree follows again the idea of an increasing complexity.

The tenth tree (p.535) is a tree of echinoderms. It shows a succession of echinoderm groups.

The eleventh tree (p.551) is a tree of crustaceans. It is very similar to the previous one.

The twelfth tree (p.555) is a tree of Tracheata, that is to say of terrestrial arthropods.

The thirteenth tree (p.577) is a tree of vertebrates

The fourteenth tree (p.583) is a tree of anamniotic craniates. It classifies anamniotes, that is to say all vertebrates with a skull except those with an amnios

The fifteenth tree (p.593), based on paleontology is a monophyletic tree of vertebrates. It seems strictly genealogical, but is not based on a calculation. It only presents the ideas of Haeckel about evolution. Thus, animals are not classified by their genealogical order, but according to a Lamarckian principle of increasing complexity.

The sixteenth tree (p.685) is a hypothetical sketch of the monophyletic origin and distribution of the twelve kinds of humans on earth since the Lemurian strain. This tree is very different from others. Here, the support is a map.

His *Anthropogenie* contains only one tree: the "Family Tree of Mankind". This tree is certainly Haeckel's most famous. It represents a "concrete" tree, being both a tree of life and a scale of beings.

Alfred Henry Garrod

1846 (London, England) - 1879 (London, England)

As an apothecary, Garrod wad fascinated by zoology. He studied and became a teacher of this discipline at the St John's College, Cambridge. But he died of tuberculosis aged 33.

We studied his article *"On Some Points of the anatomy of the parrots; All which bear on the classification of the suborder"* (1874). This is a classification of Psittacidae. Retrospectively, we could consider that the method used is innovative and strikingly similar to what Hennig will propose 75 years later: Garrod defines specific characters for each family, and groups families together on the basis of *derived characters*.

On page 591, a tree is drawn, followed by a Venn diagram. The figure shows those sharings.

Edwin Ray Lankester

1847 (London, England) - 1929 (London, England)

Geologist and zoologist, Lankester was a convinced evolutionist. He proposed to differentiate organs into which is inherited from a common ancestor (now called *homology*) from what looks like having been acquired independently (now called *homoplasy*).

We studied his article *"Limulus*, *an arachnid"* (1881). He wondered there about the classification of the family of horseshoe crabs. Should they be placed, according to the general opinion among the crustaceans or near arachnids? Lankester moves in favour of the second proposal. More importantly, he provided morphological arguments to support this hypothesis.

Alfred William Bennett

1833 (Clapham, England) - 1902 (London, England)

Botanist, Bennett is famous for the research expedition that he has made in 1875 to study Swiss flora. He communicated with Darwin during the 1870’s.

We studied his article *"On the affinities and classification of algae"* (1887). Bennett distinguished three main types of plants: Protophyta, lacking chlorophyll; Cyanophyta, and Chlorophyllophytes. Each group has its own classificatory tree. Plants are grouped according to their "affinities" of shapes. These series of affinities show the complexity of classified forms: the more they are distant from the root, the more they are complex.

Eduard Hackel

1850 (Haida, Bohemia) - 1926 (Attersee, Austria)

Botanist, Hackel taught and conducted research on this discipline. He did in 1876 an exploration in Spain and Portugal and discovered the role of *lodicules* in the reproduction of flowering plants.

We studied his work *Monographiae Phanerogamarum* (1889). Written in Latin, it exposes (page 723) a tree. This tree does not show family links, but affinity ones, which *"can be partly explained by the genealogy*.*"*

Louis Antoine Marie Joseph Dollo

1857 (Lille, France) - 1931 (Brussels, Belgium)

French paleontologist working in Belgium, Dollo studied specifically fossil "reptiles".

We studied the trees of his article *"Sur la phylogénie des Dipneustes"* (On the phylogeny of lungfishes). This article proposes to reconstruct the genealogical ties that bind lungfish. Dollo draws two shafts (pp.89 and 113).

The first is a classification of fossil lungfish. The second classification of gnathostomes (jawed vertebrates).

Peter Chalmers Mitchell

1864 (Dunfermline, Scotland) - 1945

Scottish zoologist, Mitchell was the secretary of the Zoological Society from 1903 to 1935.

We studied his article *"On the intestinal tracts of birds; With remarks on the valuation and nomenclature of zoological characters"* (1901). This article comes from his research in anatomy on the birds of the Zoological Society. Mitchell classifies birds according to the changes they share in the shape of their intestine.

William Patten

1861 (Watertorn, USA) - 1932 (Hanover, USA)

American zoologist, Patten advocated for an arachnid origin of vertebrates. He also defended the idea of harmonious cooperation in evolution.

We studied his book *The Evolution of the Vertebrates and Their Kin* (1912). There, Patten exposes his ideas about the arachnid origin of vertebrates. He shows the many similarities between both to support his thesis.

Patten draws two trees. The first (p.382) is a phylogeny of different groups of vertebrates. The second (p.397) classifies *Acrania* and describes their transformation from their arthropod ancestor. The "+" and "-" marks are drawn on the shaft of the bubbles. They correspond to the different character states: presence or absence.

Charles Edwin Bessey

1845 (Milton Township, Ohio) - 1915 (Lincoln, Nebraska)

American botanist, Bessey works on the classification of angiosperms.

We studied his article *"The phylogenetic taxonomy of flowering plants"* (1915). Bessey draw a tree (p.118) made of series of *"blobs"*. This image evokes a cactus. The *"blobs"* show the succession of groups in their evolution.

Benjamin Charles Gruenberg

1875 (Novo Sielitz, Romania) - 1965 (New York, USA)

Professor of Biology, commercial chemist and writer Gruenberg did not devote his life to research. He was particularly involved in the dissemination of knowledge.

We studied his book *Elementary Biology*: *an introduction to the science of life* (1919). Written for students, it is a synthesis of knowledge in biology. The last section is a classification of species containing two trees: the first one (p.479) classifies plants, the other one (p.483) classifies animals. These shafts are designed in the manner of "real" trees.

Robert ("Robin") John Tillyard

1881 (England) - 1937 (Goulburn, Australia)

First working as a math teacher, Tillyard discovered zoology and entomology and devoted to these. His works about the physiology and classification of insects gave him several awards.

We studied two articles from him. The first one, "*The panorpoid complex"* (1919), is a classification of Panorpoids, a group of insects, (including butterflies, flies and fleas) following the veins of their wings. Tillyard draws there a tree (p.708). This is a "Spindle Diagram": its branches are thick. But this tree is very different from those drawn, for example, by Romer or Agassiz: bubbles are indeed grouped into complete groups. More precisely, the bubble is a hypothesis of phylogeny.

We also studied the article *"A New Classification of the Order Perlaria"* (1921). The tree (p.38) drawn here has no "blobs": its branches are thin. Its data come from a table of characters, whose states are distinguished between "Archaic" and "Specialized".

Charles Lewis Camp

1893 (Jamestown, USA) - 1975 (San Jose, USA)

Zoologist and palaeontologist, one of his teachers was W.K. Gregory. Camp specialized his research interests to amphibians and reptiles.

We studied his article *"Classification of the lizards"* (1923). This long article is a main one in herpetology. Camp elaborated a phylogeny of lizards. Thus, he described each character used for this study, Then, he groups the different species by their sharing of derived characters. But the tree he drawn (p.333) created incomplete groups.

William Diller Matthew

1871 (St John, Canada) - 1930 (United States)

Paleontologist, specialized in the study of vertebrates, Matthew also studied zoology, geology and mineralogy.

We studied his article *"The Phylogeny of Dogs"* (1930). This phylogeny was reconstructed by fossil data. Each fossil represents one of the "types" which follows each other.

Matthew drew two trees. The first one (p.119) is a "spindle diagram". It shows the diversification of the Carnivora).

The second one (p.132) results of his studies on the skulls of fossil dogs. Branches are linear.

Daniele Rosa

1857 (Susa, Italy) - 1944 (Novi Ligure, Italy)

Teacher in biology and comparative anatomy, Rosa spent his career at the Museum of Turin.

We studied his book *“L'Ologenèse*. *Nouvelle théorie de l'évolution et de la distribution géographique des êtres vivants”* (The Ologenesis, New theory of evolution and geographical distribution of living beings). This book proposes a theory about the diversification of species. The causes of the evolution of species are internal and should not be sought in their environment. Rosa based his theory on the notion of *idioplasma*, support of the heredity, and offered a theory of transmutation of species, closely related to a theory of classification.

Rosa drawn five trees. The first one, p.175, illustrates the procedure that Rosa proposed to reconstruct a genealogy.

The second one, p.191, shows a comparison between Rosa's classification and a more traditional one.

The third tree, p.250 illustrates the duplication of the *idioplasma* during evolution.

The fourth tree, p.260, shows the principle of the diversification of species.

The fifth and last tree illustrates the idea that when they separate, the two bloodlines do not have the same future. One is "early" but is rather complex, while the other is "late", but more complex.

William King Gregory

1876 ​​(New York, USA) - 1970 (New York, USA)

Gregory became a research assistant of Pr. Osborn at the American Museum of Natural History. The first group of animals he studied was fishes, but he soon moved his interest to land vertebrates. Gregory had a particular passion for comparative anatomy. Far from performing mere descriptions or comparisons of organs, Gregory proposed theoretical principles such as "habit" or "legacy". Beings are composed of a mosaic of characters: some are primitive, others are specialized. From this, Gregory proposed in 1947 his " palimpsest theory". A palimpsest is a document written on an already used parchment, from which the first text has been scraped or washed off. Following this idea, ancestral characters tend to be replaced by the modified ones, which complicates the determination of kinship.

Fish skulls

*Fish Skulls* is a comprehensive study of fish skeletons. Gregory initially defines the anatomical elements that will be studied in this book. He then focuses on their transformations among the different groups. Finally, he describes each of these groups. In this last section, the largest of the book, comes several "phylogenies" of fishes.

Tree 1: *"Inferred phyletic connections on the main branches of the scombriform fishes"*, p.318.

This tree is a phylogeny of Scombriformes (family of tunas or mackerels). It consists of five main branches, which join at the base but not connect each other. Fishes are classified in thick branches. The shape of this tree has been inspired by marine plants, a form that we have not seen in any other figure.

Tree 2: *"Divergent Evolution in Ceratioids"*, p.405

This tree is a classification of Ceratoids, an order of abyssal fishes. On each node, leaves and on the root is set an identified species of fishes.

Tree 3: *"Tentative Phylogeny of the Major Groups of Fishes*. *Mostly based on Skull Structure"*, p.412

The fine branches of this tree are structured around a trunk, symbolizing the increasing complexity of the classified fishes.

Tree 4: *"Evolution of the Fishes"*, p.455

This tree is very different from the three previous ones. Here, the height of the branches does not correspond to a degree of complexity but a strict temporal order. Indeed, the tree follows the geological times. Its branches are not homogeneous: most of them are fine, but a "bubble" represents the central group of teleosts. Groups are derived from one another.

Trees in *Evolution Emerging* (1951)

Tree 1: *"Family tree of Common Sea Shells"*. In. Volume 2, p. Fig 40. 2.38.

The first tree is formed of thin arrows. They interconnect the various classified shells. At the top of the tree are the names of certain groups. These groups are incomplete, and follow one another.

Tree 2: *"Comparative History of Invertebrates & Vertebrates"*. In. Volume 2, p. 86, fig. 6.1.

Very different from the previous one, this tree is a classification of animals. Gregory splits them into two groups: vertebrates at right, other invertebrates on the rest of the tree. The group of invertebrates is, unsurprisingly, an incomplete group. Gregory primarily uses sets of fossils to reconstruct the tree. When he develops in this way: the branches of the tree are represented by solid black lines.

Tree 3: *"Family Tree of the Placoderms"*. In. Volume 2, p. 120 fig.7.28.

Very similar in its structure to the first tree, this one classifies the fossils of an extinct group: placoderms.

Tree 4: *"Winged Sharks*. *Adaptive Branching of the Skates and Rays"*. In. Volume 2, p. 170, fig.8.60.

Although very similar to the trees 1 and 3, this tree is specific in that some animals are ranked on some of its nodes.

Tree 5: *"Family Tree of the Ganoids and Teleost Fishes (1)"*. In. Volume 2, p. 174 fig.9.1.a. and Tree 6: *"Family Tree of the Ganoids and Teleost Fishes (2)"*. In. Volume 2, p. 175 fig.9.1.b.

Those two trees are very similar. They are succession of thick "bubbles". Some of those are related to each other, other are separated.

Tree 7: *"Selective Branching of the Alepocephaloidea"*. In. Volume 2, p. 205 fig.9.37.

This tree, which classifies a group of teleost fishes, is also composed of arrows. But here, each taxon is set on leaves, and not any on the branches, nodes or root.

Tree 8: *"Branching of the Stomiatoids"*. In. Volume 2, p. 207 fig.9.39.

Very similar to the previous one, this tree is a classification of a teleosts group. Here, however, one of those animals is set at the root, while others are placed on branches.

Tree 9: *"Adaptive Branching of Carnivorous Characins"*. In. Volume 2, p. 213 fig.9.47.

Very similar to the previous two trees, this one presents group names around the tree.

Tree 10: *"Provisional phylogeny of the Herbivorous Characins"*. In. Volume 2, p. 214 fig.9.49.

This tree, describing the same group as the previous tree, classifies however fewer taxa. Tritomies (separation of a three-branch) are more frequent.

Tree 11: *"Provisional Phylogeny of the Iniomi"*. In. Volume 2, p. 227 fig.9.63.

This tree, made of arrows, classifies a deepwater teleost group (Iniomi). Some of these animals are set at the branches of the tree, other at the nodes. One of these animals is the set on the root.

Tree 12: *" Range of Body Forms in Fossils and Recent Carangoids"*. In. Volume 2, p. 272 fig.N.

This other classification of teleosts is made of arrows. The root is formed by three arrows.

Tree 13: *" Outline of Fish History"*. In. Volume 2, p. 321 fig.9.166.

This tree is very different from the previous ones. Included in the series of paleontological eras, it is formed of two bubbles and linear branches. Some of those objects are not connected to the others.

Tree 14: *"Suggested Family Tree of the Air Breathers"*. In. Volume 2, p. 324 fig.10.1.

Gregory outlines in this chapter the evolution of "air-breathers" (lungfishes and coelacanths, close relatives of tetrapods). This tree is composed of arrows, but its temporal hierarchy is strict. Some of the classified animals are set in the branches of the tree, others at its nodes. None, however, is set on the root.

Tree 15: *"Diverse Body and Skull forms Earlier in Amphibia"*. In. Volume 2, p. 369, fig.11.28.

Made of arrows, this tree classifies the two traditional groups of fossil amphibians: labyrinthodonts and lepospondyles. Two geological eras are shown in the figure: Perm-Triassic and Carboniferous.

Tree 16: *"Branching Evolution in Reptiles*, *Birds and Mammals"*. In. Volume 2, p. 378 fig.12.1.

This tree is very different from the previous ones. Composed of dotted lines, it is set into geological eras. No taxon is drawn, but the names are written at the nodes of the tree. Crosses figure extinct groups, arrows still-existing ones.

Tree 17: *"Evolution of the Pineal Eye-Hole in Geologic Time"*. In. Volume 2, p. 442–443, fig.12.53.

This tree is based on the transformation of a character: the pineal eye pit. The tree has a semi-circular shape. Each group is represented by its skulls.

Tree 18: "*Phylogeny*, *Life-zones and Locomotion*". In. Volume 2, p. 468 fig.14.1.

This tree, of complex aspect, illustrates two data at the same time: the phylogeny of sarcopterygians (tetrapods, lungfishes and coelacanths) and their mode of locomotion. The arrows illustrate the transition from one mode of locomotion to another.

Tree 19: *"Branching Evolution and Palatal Types in Birds"*. In. Volume 2, p. 547, fig.15.25.

This tree, with linear branches, presents the evolution of birds. Some branches present a strict chronology; other a relative one.

Tree 20: *"Tentative Family Tree of North American Pelycosaurs"*. In. Volume 2, p. 549 fig.16.2.

This tree is a classification of Pelycosaurs, an extinct amniotes group close to mammals. Two geological eras therein. Animals are represented by a drawing of their skull. Two taxa are not classified on nodes, but on branches.

Tree 21: *"Adaptive Branching of the Marsupials"*. In. Volume 2, p. 663 fig.18.21.

This tree is a classification of living and fossil marsupials. Branches are thicker and have an arrow shape. Most groups are located in the leaves.

Tree 22: *"Adaptive Branching of the Kangaroos in relation to habitat"*. In. Volume 2, p. 681 fig.18.I.

This large tree has thick trunk and branches. It is a phylogeny of kangaroos. Existing and fossil species are presented. All are located on the leaves of the tree.

Tree 23: *"Phylogeny and Distribution of edentates and Supposed edentates"*. From Simpson. In. Volume 2, p. 705 fig.19.25.

This tree is a "bubble tree." It fits into the succession of geological ages and offers a history of the evolution of a class of mammals: edentates. This tree was produced by Simpson, but reinterpreted by Gregory in this book. That is why we have chosen to include it.

Tree 24: *"Simplified Family Tree of the Rodents"*. In. Volume 2, p. 718 fig.19.40.

This tree is a phylogeny of rodents. A fossil squirrel, *Paramys* is situated at its root. Numerous branches are arranged circularly.

Tree 25: *"Independent derivation of ricochetal kinds of rodents in different families"*. In. Volume 2, p. 723 fig.19.46.

This tree, originally produced by Hatt and reinterpreted by Gregory has a very different form from the previous ones. Angles are straight and branches thick. But above all, the height of the branches does not indicate an idea of temporality, but a way of locomotion.

Tree 27:*" Phylogeny and Classification of the cat-like (aeluroid)"*. In. Volume 2, p. 740 fig.20.16.

made with Hellman, this tree is a phylogenetic classification of Felidae.

Tree 28: *"Main Branches of the Oreodonts"*. In. Volume 2, p. 868 fig.21.98.

This tree, made of arrows, does not follow a strict temporality. It rather shows the diversification in the forms of a group of mammals: ungulates. Five forms are classified.

Tree 29: *"Main Branches of Antelopes"*. In. Volume 2, p. 886–887, fig.21.116.

This tree is large. Its thin branches are made of arrows. Its chronology is neither strict nor relative: this tree shows the diversification of antelope forms.

Tree 30:*" Skulls of cebids"*. In. Volume 2, p. 942–943, fig.23.34.

This tree, composed of thin arrows, shows a classification of primate skulls.

Tree 31: *"Grandfather Fish and Its descendants"*. In. Volume 2, p. 992 fig. 24.I.

This tree, reproduced from a booklet of science popularization, is made of bubbles. It presents, humoristically, the succession of vertebrates: the "fish" ancestral form has the role of the "grandfather", from which the other forms descent: jawless fishes, placoderms, cartilaginous fishes and bony fishes.

Tree 32: In. Volume 1, p. coverage.

The tree shown here is drawn on the cover of the first volume of Gregory's *Evolution Emerging*.

It represents the development of vertebrates through geological times. This tree is made of "bubbles". Groups follow one another: mammals and birds to reptiles, tetrapods to fishes. Far right of the tree, mankind is set apart from other mammals.

John Henry Schaffner

1866 (Marion County, USA) - 1939

Schaffner discovered sciences while he was in College. He taught these disciplines and dedicated to research in life sciences.

"Phylogenetic taxonomy of Plants." In. Quarterly review of biology.

This article proposes a new type of classification of plants. Taxonomy, proposed Shaffner, should be based on the species phylogeny. He thus delineated ten major stages in the evolution of plants. These stages are "grades", that is to say levels of complexity.

Tree 1: p.142

This tree is a three-dimensional graph. It shows both the evolutionary history of plants, their classification and their current diversity. A vertical trunk displays the succession of groups from the least to the most complex. The root of the tree shows archaeophytes, which Schaffner recognized as the first stage of the evolution of plants.

Tree 2: p.150

The second tree, although similar in appearance, is quite different. It is a two-dimensional graph and does not have any trunk. Its shape is semi-circular and parallel lines run from the centre to the edge. They represent a set of characters carried by the different groups. The most a group is given as "evolved" the most its branch is near the edge of the circle.

Herbert Faulkner Copeland

1902–1968

Little is known about Copeland's life. His major work is a new classification of microorganisms. He formed the group Primogenium corresponding to Eukaryota (filled core cells), with the exception of plants and animals.

*"The kingdom of organisms"*. In. Quarterly review of biology.

This article proposes a new organization of the kingdoms of life. At the end of the article, Copeland draws a tree showing the phylogenetic relationships and the development of those kingdoms. The most a group has changed, the most its branch recedes from its strain-group. For example, the Monera, few derivated are drawn in a substantially vertical bubble.

Lucien Cuénot

1866 (Paris, France) - 1951 (Nancy, France)

Preparer of compared Anatomy and Physiology at the Faculty of Paris, Cuénot settled in 1890 at the Faculty of Sciences of Nancy, where he remained for the rest of his life.

In 1840 Cuénot wrote his *"Essai d'un arbre généalogique du règne animal"* (Essay of a family tree of the animal kingdom). The tree drawn there is pictured as a "real" tree: it has a thick trunk, branches, thick leaves and even roots that plunge into the ground. Although only the animals were detailed, plants are only discussed. Groups follow each other along the trunk. According to Cuénot, evolution is “complete”: since the last 500 million years, no new group appeared. Life is going to gradually extinct.

Ruben Arthur Stirton

1901 (Muscotah, USA) - 1966

Zoologist, Stirton studied specially of mammals and birds, both living and fossil.

*Phylogeny of north-american equidae"*. In. Bulletin of the Department of Geological Sciences.

Sirton proposed, in this article, a phylogeny of *equidae* based on their fossils. At the end of the article is drawn a large tree. It is a "bubble tree". Its branches are thick and groups follow each other among geological eras. Groups are defined according to an overall shape and the sharing of attributes, both ancestral or derived.

Charles Duncan Michener

1918 (Pasadena, USA)– 2015 (Lawrence, Kansas, USA)

As a son of naturalists, Michener discovered Natural Sciences as a child. He specialized in the study of Lepidoptera (butterflies). He’s known for having published with Robert Sokal in 1958 the most commonly used hierarchical clustering method using distance averages called UPGMA.

*Comparative external morphology*, *phylogeny*, *and a classification of the bees (Hymenoptera)*. In. Bulletin of the American Museum of Natural History.

This article is a study of the external morphology of bees. Michener firstly performed the accurate study of a bee, *Anthophora edwardsii*. Then he performed an analysis of comparative anatomy of bees. Finally, Michener established the phylogeny of this group. There, he draws a tree. The groups follow one another according to their degree of specialization.

Jean Delacour

1890 (Paris, France) - 1985 (Los Angeles)

During his childhood, Delacour discovered ornithology, especially bird breeding. He studied natural sciences, particularly horticulture and bird watching. He earned from the University of Lille a PhD in biology. Ornithologist and bird breeder, his life was closely linked to the history of his castle at Clères in Normandy.

*"The Family Anatidae*.*"* Jean Delacour & Ernst Mayr. In. The Wilson Bulletin

This article is a classification of ducks based on their characters. All the groups developed on this tree are complete ones. Yet they do not correspond to what we would today call "monophyletic" groups: they indeed gather species on the basis of a set of defined characters, but those ones may be ancestral. Thus, the method used for the elaboration of this tree is closer to the phenetic methods. Yet, paradoxically, this tree is called "phylogenetic" (p.7).

Pierre Teilhard de Chardin

1881 (Orcines, France) - 1955 (New York, USA)

Jesuit priest and paleontologist, Teilhard de Chardin travelled throughout his scientific career and conducted researches on the ancestors of mankind.

Published posthumously, Teilhard de Chardin's *Phénomène Humain* (The Human Phenomenon) shows the vision that the author has about the nature of evolution. Three trees are drawn in this book.

Tree 1, p131

The first of those trees looks like a classificatory tree of fossils. At the left of the figure is drawn a time scale. The figure itself is a sequence of branches. This tree shows the increasing complexity of groups and the appearance of new "types". Thus, the curves drawn on the top of each "type" show the greater or lesser degree of perfection of the given group.

Tree 2, p.172

This tree shows the development of primates. Anthropoids, among which is mankind, are drawn therein.

Tree 3, p.212

The third tree of the book shows the development of mankind from primates, and the convergence of humans in the "Omega Point."

*Le groupe zoologique humain*, *ou la place de l'Homme dans la nature* (The human zoological group, or the place of mankind in nature) follows Teilhard's *Phénomène Humain*. It has been published anonymously in 1962 and extended the ideas presented in his previous book.

On the page 47 of the book is represented a tree. It shows the evolution—understood in the sense of increasing complexity–of he major lineages of beings: chordates, coelenterates, arthropods and plants.

George Gaylord Simpson

1902 (Chicago, USA) - 1984 (Tucson, USA)

Professor of paleontology (and palaeontologist), Simpson was also a curator at the American Museum of Natural History.

*Principles of Animal Taxonomy* is a methodological work. Simpson proposes there a set of methods to name and classify species, since their description to the elaboration of a tree, then the publication of a scientific article. Many trees appear in this book.

Tree 1, p.104

This tree illustrates the phenomenon of adaptive convergence and parallelism. Here, Simpson established groups according to the degree of similarity between species.

Tree 2, p.119

The second tree shows more clearly how to form group, following the degree of divergence between species.

Tree 3, p.138 (a)

On page are drawn two trees, each one being a way to group species in a theoretical phylogeny.

Tree 4, p.138 (b)

This second tree focuses more on the overall shape of the animal.

Tree 5, p.143

This tree shows the formation of groups over times, and relates it to the assignment of taxonomic ranks. The dotted lines show genera and crosses delimit families.

Tree 6, p.195

This tree shows two hypotheses of phylogeny: one drawn in solid lines, the other in dotted lines. The differences between both are based on the characters of the species listed.

Tree 7, p.197 (A)

The following two charts are shown on a same page. They propose two possibilities of reconstructions. This first tree shows a divisive method. It splits each group into two, on the basis of the date of occurrence of a given node. Groups are incomplete.

Tree 8, p.197 (B)

On the second tree groups are based on the sharing of a common ancestor. They are, in the modern sense of the term, monophyletic. However, Simpson blames them as they greatly complicate the elaboration of the tree.

Tree 9, p.202

This ninth tree shows the different configurations that can take the evolution of species. It illustrates the phenomenon of "evolutionary explosion", that is to say the massive diversification of a group.

Tree 11, p.211

If all previous trees were purely theoretical, Simpson proposes here a concrete illustration of this elaboration with the determination of different groups of the order Carnivora.

Tree 12, p.213

This tree is a second concrete example of classifications. It presents a classification of primates.

Tree 13, p.215

This second tree of primates is even more explicit. Decomposed into "grades", it shows the increasing progression of intellectual and social abilities of the different primate groups.

Tree 14, p.217

The last tree of this book is a phylogeny of the families of rodents established according to the fossil record and arranged in a succession of forms.

Robert R. Sokal

1926 (Vienna, Austria) - 2012 (New York, USA)

Entomologist and expert of biogeography, it is for his seminal work on numerical taxonomy and phenetics, conducted with Peter HA Sneath, that Sokal is the most famous.

Peter H. A. Sneath

1923 (Galle, Sri Lanka) - 2011 (Leicestershire, England)

Researcher in medicine, Sneath is famous for his works on the phenetic method.

We have studied three of their works. Two of them were jointly written by Sokal and Sneath; the third is an article written by Sokal.

Principles of Numerical Taxonomy

Released in 1963, the Numerical Taxonomy of Sokal and Sneath is the founder work of the phenetic method. It offers both the epistemological and methodological foundations of this approach. Several trees are drawn. We kept two of them : those made ​​by Sokal and Sneath themselves.

Tree 1: Figs 7–10, p.201

This tree is the result of a phenetic analysis of two sub-types of bees. The horizontal lines indicate the degree of similarity between two nodes.

Tree 2: Figs 8–1, p.219

The second tree has been elaborated ​​using the phenetic method. But its structure is different: it is three-dimensional in order to show the different rates of evolution.

Sokal, R. R. *"Numerical Taxonomy"*. In Scientific American

In this article, Sokal popularizes the phenetic method. Twenty-nine Caminalcules, small imaginary animals invented by Professor Joseph Camin, are there classified.

Numerical Taxonomy

Published in 1973, this second work is different from the first one: the scientific community has lived the arrival of cladistics, the development of new algorithms, the spread of computers and the implementation of phenetic methods in the scientific field.

Tree 1: Figs 6–9, p.334

This first tree is called a "cladogram", that is to say a tree showing interrelationships established following the cladistic method, a name given to Hennig’s phylogenetic systematics. It classifies seven caminalcules.

Tree 2: Figs 6–13, p.345

The second tree is a classification of beings according to several phenetic methods. Paradoxically, this tree is called a "phylogeny". Yet it is produced according to distance methods.

Willi Hennig

1913 (Dürrhennersdorf, Germany) - 1976 (Ludwigsburg, Germany)

German entomologist, Hennig is a central figure in the history of systematics He is indeed the leader theoretician of phylogenetic systematics, later called the “cladistic method”.

We studied his major work: *Phylogenetic Systematics*. But this works contains dozens of trees and we could not treat them all. We thus had to remove those whose message was similar.

Tree 1: Fig 21, p.89

This tree illustrates the phenomenon of speciation and transformation of characters. Characters states A and B are the initial states—apomorph ones. The states A', B' and B'' are derived (or plesiomorph) characters.

Tree 2: Fig 32, p.110

The second tree illustrates the correspondence between a tree and a Venn diagrams. Here, a phenomenon of horizontal transfer occurs: the white dots correspond to parasitic species of those represented by black dots.

Tree 3: Fig 47, p.150

The third tree shows the relationships between different groups of brachiopods (a group of marine animals). Thick lines cover the tree. They correspond to the derived characters of the different characters studied.

Tree 4: Fig 65, p.215

The fourth tree is included in a discussion about the evolutionary radiations and polytomies.

Alfred Sherwood Romer

1894 (White plains, USA) - 1973 (Cambridge, USA)

Paleontologist, Romer specialized in the study of fossil vertebrates. He is also the author of numerous paleontological publications, for both the general public and specialists.

Trees Romer

Vertebrate Paleontology

Tree 1: Volume 2, page 20

The first tree is a family tree of invertebrates and lower chordates. It presents the classification of all animals, except vertebrates. The tree is made of linear branches.

Tree 2: Volume 2, page 22

This tree is a comprehensive vertebrate phylogeny. It separates them into eight grades, seven reaching until modern times.

Tree 3: Volume 2, page 39

This tree is again a "bubble" tree. It presents the transformations of the first vertebrates, classified into three groups: jawless fishes, placoderms and cartilaginous fishes.

Tree 4: Volume 2, page 76

This tree is a classification of bony fishes and amphibians. It is again a "bubble tree" and shows the succession of geological eras. Its base is formed by two branches, which are not interconnected by a root

Tree 5: Volume 2, page 105

This fifth tree is very different from the previous ones. It is strictly linear: no "bubbles" in it. But it also has a vertical stem, in which groups follow one another.

Tree 6: Volume 2, page 171

This tree is a classification of "reptiles". It is a "bubble"tree, which root forms a polytomy and bubbles fan out.

Tree 7: Volume 2, page 210

This tree is a classification of archosaurs, a group of "reptiles" including crocodiles, dinosaurs (ornithischians and saurischians) and pterosaurs but excluding birds.

Tree 8: Volume 2, page 310

This "bubble" tree shows the development of the "types" of lower mammals, monotremes and marsupials.

Tree 9: Volume 2, page 325

This tree is a phylogeny of placental mammals. "Bubble" tree, its root forms a polytomy and uncertain kinship links are shown by dashed lines.

Tree 10: Volume 2, page 363

This tree is a phylogeny of the Carnivora group, a group of mammals.

Tree 11: Volume 2, page 421

This tree shows a phylogeny of Perissodactyla, a group of mammals.

Tree 12: Volume 2, page 443

This tree is a provisional phylogeny of artiodactyls groups.

Tree 13: Volume 2, page 492

Drawn in the chapter on cetaceans, this tree is a phylogeny of this group. Cetaceans are divided into three groups: archaeocetes, odontocetes (dolphins, killer whales …) and whales.

Tree 14: Volume 2, page 495

This tree is a phylogeny of rodents. Data are based on fossil records.

Tree 15: Volume 3, page 12

This tree is a classification of invertebrates. These are seen as groups succeeding each other.

Tree 16: Volume 3, page 13

Similar to the second tree of our study, this figure is also a vertebrate phylogeny.

Tree 17: Volume 3, page 25

This tree is also a "romerogram”. it is a classification of early vertebrates in "grades". Romer distinguishes jawless fishes, placoderms and cartilaginous fishes.

Tree 18: Volume 3, page 47

This tree is a classification of bony fishes and amphibians.

Tree 19: Volume 3, page 62

This tree is very different from the previous one: its branches are fine and no bubbles cover them. It classifies the different teleosts.

Tree 20: Volume 3, page 108

This tree is a phylogeny of reptiles.

Tree 21: Volume 3, page 137

Phylogeny of archosaurs, this tree is a "bubble tree".

Tree 22: Volume 3, page 208

Similar to the ninth tree, this one is a temporal distribution of placental mammals.

Tree 23: Volume 3, page 232

Similar to the tenth tree, this figure is a classification of Carnivora.

Tree 24: Volume 3, page 263

The following tree is a phylogeny of Perissodactyla.

Tree 25: Volume 3, page 274

This tree, similar in structure to the twelfth tree, classifies artiodactyls.

Tree 26: Volume 3, page 300

Similar to the thirteenth tree, this one is a phylogenetic classification of cetaceans.

Tree 27: Volume 3, page 303

This tree, the largest of the book, is a phylogeny of rodents.

1967 "Major Steps in Vertebrate Evolution." In. Science, vol. 158 (3809), p. 1629–1637

This article relates the history of vertebrate evolution. The tree we studied is not a "romerogram" but a series of fine arrows showing the succession of forms that led to extant vertebrates. This tree is highly finalist. Indeed, Romer saw mankind as "the end of the story" of vertebrates.

1973 *L'oigine des classes de Vertébrés* (The origin of vertebrate class, in. La Recherche)

Published in the French journal La Recherche, this article discusses the "monophyly" of vertebrates. Did they appear from a single common ancestor, or more?

Tree 1: page 350

The first tree is a "bubble" tree. It shows a classification of "fishes" in their traditional definition: all vertebrates except tetrapods.

Tree 2: Page 354–359

This tree is a semi-circular phylogeny of tetrapods.

Peter Humphry Greenwood

1927 (Redruth, England) - 1995 (London, England)

Zoologist, Greenwood specialized in the study of fish.

*Phyletic studies of Teleostean fishes with a provisional classification of living forms*. In. Bulletin of the American Museum of Natural History.

This article, lavishly illustrated, is a vast anatomical study and classification of teleosts (most of "fishes"). The tree appears at the beginning of the article. It presents the phylogeny of this group. The branches of the tree are fine, but "bubbles" are superimposed.

Walter Monroe Fitch

1929 (San Diego, USA) - 2011

Professor of Biological Sciences, Fitch is mostly famous for his works in the field of molecular systematics. He developed methods to reconstruct interrelationships among species from protein and DNA sequences.

Emanuel Margoliash

1920 (Cairo, Egypt) - 2008 (United States)

Research Biochemist, Margoliash is best known for his work with Fitch.

1967 "Construction of phylogenetic trees" in. Science, vol. 155 pp.279-284

This six-page article lays the foundations of a tree reconstruction method using pairwise distances calculated from aligned protein sequences. The idea of reconstructing phylogenies using molecular data was developed two years ago by Emile Zuckerkandl and Linus Pauling (1965).

Four trees are pictured in this article. We studied those drawn p.282 and p.283 of the article.

Tree 1: Fig 3, p.282

This first tree is a molecular phylogeny of several globin (a protein present in the blood of animals and which is part of hemoglobin).

Tree 2: Fig 4, p.283

This method allows much finer analyses than the mere morphological reconstructions. Thus, this tree shows the differences between different populations of India.

Ernst Mayr

1904 (Kepten, Germany) - 2005 (Bedford, USA)

Son of amateur naturalists, Mayr was particularly interested in birds and became an ornithologist. He is famous for his general views about evolution, his books and his acerbic writings. But he drew very few trees.

1974 *"Cladistic analysis or cladistic classification*? *"*. In. Zeitschrift für Zoologische Systematik und Evolutionforschung

In this famous article, Mayr opposed strongly to Hennig's cladists ideas. According to him, cladistics should only be the first step of an analysis. Later on, classification should include phenetic methods, global similarity, ecology, i.e. various parameters to classify species into grades. From the seven trees drawn here, we only kept four: those made ​​by Mayr himself.

Tree 1: 1, p.103

This first tree shows both kinship and rates of divergence between species. The tree is genealogical, and nodes show common ancestors. But the rates and the angles of the branches show the divergence rate.

Tree 2: 2, p.103

This second shaft defines the different types of groups. Their development depends on parallelism and differences between species during evolution.

Tree 3: 4, p.112

The third shaft is a "bubble" tree. It covers a structure of branches and, unlike the two previous trees, it is not a strictly theoretical tree. It shows the sequence of "mammal" and "bird" grades since their "reptilian" ancestor.

Tree 4: 8, p.119

This last tree is again a "bubble" tree. It outlines the differences between cladistic and gradist classifications by the example of fossil flies.

Sovanmoly Hul

1946 (Phnom Penh, Cambodia)

Researcher and lecturer in plant biology, Hul is working since 1987 at the French National Museum of Natural History.

*Contribution à la révision de quelques genres de Caesalpiniaceae*, *représentées en Asie* (Contribution to the revision of some kinds of *Caesalpiniaceae*, represented in Asia).

The PhD thesis of Hul is a phenetic analysis of an Asian plants group.

On page 34 of this thesis, Hul drew ​​three trees. Each one is the result of a same classificatory method on different characters. Thus, we treated them as a single tree.

Charles W. Harper Jr.

Harper works on palaeontology techniques and methods, quantitative approaches and paleoecology.

1979 *"A Bayesian Probability View of Phylogenetic Systematics"*. In. Systematic Biology.

In this article, Harper proposes an application of bayesian methods to phylogenetic reconstruction. At page 549 of the article draw two trees.

The first tree shows how to reconstruct a phylogeny using the Bayesian approaches.

The second one is an illustration of the elaboration of the tree.

Tom Kemp

Research professor in zoology and paleobiology since 1972, Tom Kemp was also Curator of Zoology at the University of Oxford.

1982 *"The reptiles That Became mammals"*. In. New Scientist.

Written for a non-scientific audience, this article, based on paleontological data, traces the evolutionary history of mammals.

On the second page of his article, Kemp drew a tree. Forms follow one another on the branches: sphenacodonts, cynodonts, mammals, dinocephalans, etc. On the leaves of the tree, genera are represented by fossil skulls.

Lynn Margulis

1938 (Chicago, USA) - 2011 (Amherst, USA)

After studies in zoology and genetics, Margulis published in 1967 an article about the origin of eukaryotes, one of three major radiations of life. She promoted the idea that eukaryotes would derive from a symbiosis with bacteria, an idea that was already circulating before, and now validated.

Karlene Vila Schwartz

Born in 1936, Karlene V. Schwartz is an American biologist.

Here we studied two books written by Margulis and Schwartz: *Five Kingdoms*, *An Illustrated Guide to the Phyla of Life on Earth*, published in 1982, and *Kingdoms and Domains*, published in 2009.

Five Kingdoms

Written for the general public, this book provides a description and a classification of the five major kingdoms of life: Monera, "protoctists" plants, animals and fungi. The authors draw there three trees.

Tree 1: Cover

The first of these trees is a general classification of the five kingdoms. It consists of five successive "bubbles". Branches are represented in these bubbles. Numerous illustrations complement the tree.

Tree 2: p.24

Very different from the previous one, this tree contains no bubble: its branches are fine. It is a classification of Monera. This tree illustrates both the phylogeny of this group and their living environment (air or anaerobic, microaerophilic or facultative anaerobic). But these groups are paraphyletic.

Tree 3: p.160

The third tree is a phylogeny of animals. Its branches are linear, but it has a central thicker trunk. It shows the sequence of major animal forms.

Kingdoms and Domains

This book, published in 2009, is a revised edition of *Five Kingdoms*. We studied eight trees.

Tree 1: Fig I-1, p.11

This first tree shows the genealogical links between the three major kingdoms of life: Eubacteria, Eukaryotes and Archaea. It was performed according to the molecular data.

Tree 2: Non-numbered figure, p.36

This second tree opens the chapter on the super kingdom of prokaryotes, the name given to the paraphyletic group gathering bacteria and archaea.

Tree 3: Fig Prokaryotae-II-1, p.54

This tree is a justification for the separation of living in two super-kingdoms: prokaryotes and eukaryotes, the seconds being a symbiosis of the first. Drawn in color, this tree has four roots and three leaves. It shows the symbiosis that occurred in the history of these groups.

Tree 4: non-numbered figure, p.118

This tree opens the second chapter of the book, about Protoctistes. This paraphyletic group groups all unicellular eukaryotes.

Tree 5: Fig A, p.169

Very different from the previous one, this tree comes from a maximum likelihood analysis of a set of molecular data. It is a classification Stramenopiles, a group close to eukaryotic plants.

Tree 6: non-numbered figure, p.232

This tree opens the chapter about animals. It has a trunk and some of its branches are vertical.

Tree 7: Non-numbered figure, P.380

This tree opens the fourth chapter, about fungi. But the tree is much more complex than a simple phylogeny of the group: it shows the symbiosis that might have occurred.

Tree 8: non-numbered figure, p.412

This last tree is set at the beginning of the fifth and last chapter of the book. It is a phylogenetic classification of plants.

Naruya Saitou

Saitou is Professor of Anthropology and Genetics at Mishima, Japan.

Masatoshi Nei

1931 (Miyazaki, Japan)

Nei is a professor of genetics and biology. He received the 2013 Kyoto Prize in Basic Sciences.

*"The Neighbor-joining Method*: *A New Method for Reconstructing Phylogenetic Trees"*.

The Neighbor-joining method is a method for constructing trees from pairwise distances among taxa. It is not the first distance-based tree reconstruction method, but it is the first to minimize patristic distances. We analyzed two of their trees.

Tree 1: 5, p.414

The first tree is a classification of frogs of the genus *Rana*, compared to the one established by Fitch in 1981. On branches are shown distances between taxa. Due to the calculation principles, some of these distances are negative.

Tree 2: 7, p.419

This second tree has branches of different lengths. They correspond to changes in the rate of nucleotide substitution.

Elliott Sober

1948 (Baltimore, USA)

Initially philosopher, Sober also specializes in the 1980s, in population genetics. He also participates in several groups of philosophy of science.

*Reconstructing the Past*: *Parsimony*, *Evolution*, *and Inference*.

This methodological work analyzes phylogenetic reconstruction methods, especially the parsimony principle.

We studied the tree drawn on page 30 of the book. It illustrates Sober's ideas about synapomorphies and the parsimony principle.

Gareth Nelson

1937 (Chicago, USA)

Curator, ichthyologist and herpetologist at the American Museum of Natural History, Nelson is a member of several scientific societies and has received numerous awards for his work, notably theoretical ones.

Norman I. Platnick

1951 (Bluefield, United States)

Arachnologist, Platnick was the entomology curator of the American Museum of Natural History, biology professor at the City College of the City University of New York and researcher at the University of Columbia.

*"Three-Taxon Statements*: *A More Precise Use of Parsimony*? *"*. In Cladistics.

This founder article presents a new method for reconstructing phylogenetic trees. This method is called Three-Taxon Statements. Many trees are drawn ​​in this short article: they illustrate the different steps for obtaining a tree with this method. We have chosen to study the latest of these trees. This tree is the result of the analysis with the three-taxon statement of a matrix of characters. The letters correspond to taxa, and X to the extra-group.

Philippe Janvier

1948 (Chinon, France)

French paleontologist, specialized in the study of vertebrates, Philippe Janvier is Research Director at the CNRS.

1996 *Early Vertebrates*. Fig 4.18, p. 114.

Between 470 and 250 million years ago lived the first vertebrate animals. This book describes and analyses them.

Several trees are drawn in this book. We studied, p.114, the classification of osteostracans, an extinct group of jawless vertebrates.

Andrew B. Smith

1954

Research professor in geology and palaeontology, Smith is particularly interested in three research areas: sea urchins, marine biodiversity of the Phanerozoic era and relationships among the first echinoderms.

Kevin J. Peterson

Professor of biology, ecology, evolutionary science and molecular biology, Kevin Peterson works at the Dartmouth College.

Gregory A. Wray

After first studies in molecular and developmental biology, Wray studies gene regulation and evolutionary sciences.

Tim J. Littlewood

Researcher at the Natural History Museum of London, Tim Littlewood is particularly interested in the taxonomy of Platyhelminthes ("flat worms"), molecular biology and animal phylogeny.

2004 *"From Bilateral Symmetry to Pentaradiality*: *The Phylogeny of hemichordates and Echinoderms"*, in. *Assembling the Tree of Life*

Collaborative work, *Assembling the Tree of Life* is a broad classification of many groups of living organisms. This article proposes a classification of echinoderms, strange animals: where all deuterostomes and protostomes (insects, molluscs, worms …) shows bilateral symmetry, echinoderms have five-fold (or pentaradial) symmetry.

Two trees are drawn:

Tree 1: Fig 22.8, p.376

This tree is a classification of crinoids based on their morphology.

Tree 2: Fig 22.6, p.374, Fig A

This second proposes a classification tree for echinoderms based on molecular data and established from Bayesian approaches.

Ivica Letunic

Letunic develops computer programs, including SMART (Simple Modular Architecture Research Tool), iPath (interactive Pathways Explorer) or iTOL (interactive Tree Of Life).

Peer Bork

1965 (Berlin, Germany)

After a PhD in biochemistry, Bork leads a team of research in bioinformatics at the European Molecular Biology Laboratory.

The trees studied here are quite different from all the previous ones. They do not offer new classificatory theory and do not classify species, but indeed offer a visualization of trees from the researchers' calculations. We studied this author's trees drawn in his articles.

Tree 1: p.276, in. *"Interactive Tree Of Life (iTOL)*: *an online tool for phylogenetic tree display and annotation"*. In. Bioinformatics

This first tree comes from the original version of iTOL. Branches can rotate around nodes, and the software can highlight chosen groups if they are complete ones.

Tree 2: p. 2 (W46), in. *"Interactive Tree Of Life v2*: *online annotation and display of phylogenetic trees made ​​easy"*. In. Nucleic Acids Research.

This second tree is presented with the second version of the iTOL software. Updates have been made, but the main features remain the same: leaves can rotate around nodes and groups must be monophyletic for being manipulated.

Laura Spooner

PhD student in Ritchie's laboratory at the University of St Andrews (Scotland), Spooner performed between 2002 and 2005 a PhD on the geographical distribution of Ephippigers' songs and their use for reproduction.

Michael Gordon Ritchie

Professor at the University of St Andrews, Ritchie particularly works on the links between genetics, evolution and behavior, especially in link to sexual isolation and sympatric speciation.

2006 *"An unusual phylogeography in the bushcricket Ephippiger ephippiger from Southern France*.*"* In. Nature

This article is a phylogeographic study of Ephippigers. A tree from molecular data is elaborated following the Minimum Evolution method. This method seems close both to the phenetic approach and to the parsimony one. It consists in choosing, in a set of trees established by distance method, the shortest of all.

Stephen Blair Hedges

Biologist and expert on amphibians, Hedges focuses his research on genetic and genomic mechanisms responsible of the current biodiversity. He is particularly interested in the events of speciation, diversification, extinction and biogeography of species.

Sudhir Kumar

Doctor in Genetics, Kumar teaches biology, evolutionary science and genetics. He is also the director of the Center for Evolutionary Medicine and Informatics at the School of Life Sciences at the University of Arizona.

Hedges and Kumar have developed a knowledge base for the general public: the TimeTree of Life. It can be used to merely estimate the divergence time between the two groups (man and cat, for example); but also for the visualization of a huge tree of life, concatenation of all small partial trees used for the elaboration of this base. Finally, a book describing this tree has also been produced.

Tree 1: p.7, in. Chapter "Discovering the timetree of Life", in. the Timetree of Life book

This tree is the one shown on the site TimeTree. It is a concatenation of several tens of trees. The nodes of the tree are dated and included in geological times.

Tree 2: p.2024, in. "TimeTree2: species divergence times on the iPhone." In. Bioinformatics

This second article presents a new approach this tree: the iPhone application. It is now from his mobile phone that the user can approach the TimeTree tree.

We preferred this article to the one published in 2006 because there was presented the most recent version of this tree.

James Rosindell

After mathematics studies, Rosindell is, since 2011, researcher at Imperial College London.

Luke Harmon

After a PhD in evolution, ecology and biology of populations, Harmon teaches biology at the University of British Columbia and the University of Washington. He is also postdoctoral researcher at the University of British Columbia.

*"OneZoom*: *A Fractal Explorer for the Tree of Life"*. In. Plos Biology

The OneZoom software is, as iTOL, a tree-visualization software. But the shape of the trees generated by iTOL and OneZoom are not similar: the OneZoom trees have a trunk and a fractal display.

Here we analyze three OneZoom trees: each corresponds to one of the three types of trees proposed OneZoom: spiral, feather or "natural".

Tree 1: 3 (Spiral), p.3

This first tree is built around a core spiral wound. The OneZoom trees are fractal ones: it is possible to enlarge them indefinitely to see the inner clades. There is a thick inner trunk.

Tree 2: 3 (Feather), p.3

This second tree shape takes the form of a "feather". The trunk is both thick and strictly vertical.

Tree 3: Fig 3 (Natural), p.3

The third tree, qualified as "natural", has a thick trunk and a vertical development.

### Annex 2: List of characters

Characters presentation

Axis # 1: The objects of the tree

Theme: The root

Character #1: The root carries the idea of an appreciative amount of value

No: 0;

Yes, Positive: 1;

Yes, Negative: 2;

The root of some trees does not take any consideration of values. This is for example the case of the roots of Darwin's or Hennig's trees.

Rather, the root takes a positive value when what it carries is considered as more complex or more perfect than the others objects of the tree. This is for example the case of the roots Buffon's or Duchesne's trees.

Finally, the root carries a negative value when what it carries is considered as less complex, more "primitive" than the others objects of the tree. This is for example the case in Simpson's or Lamarck's trees.

Character #2: The root refers to a named entity?

Yes, with a taxonomic rank higher than the species level: 0;

Yes, and it is a species: 1;

No: 2;

The root of the tree can refer to a living being. This entity must here be named. As an example, Buffon, Lamarck, Haeckel or Gaudry proceed as such.

For Lamarck or Haeckel in his *Generelle Morpho*logie, the living being set at the root is a type. Its taxonomic level therefore has a higher rank than the species one. What we call here "type" is not the holotype or paratype of our inventories. Holotype and paratype designate the specimen of a species that has been used for its description. But here, the term of type is taken in the sense that it had in the nineteenth century: a "global form".

But for Buffon or Gaudry, the living being set at the root of the tree is a species: in their trees these authors draw genealogies of concrete species.

Finally, in Darwin's tree, no living being appears at the root: this tree is a strictly one and does not classify specific species.

Character #3: The root refers to a supposed living being?

No: 0;

Yes, considered as an ancestor: 1;

Yes, considered as a "stem-group": 2;

The root can refer to a living being although this one is not named. The author knows that this entity has existed–or already exists–but does not know its exact identity.

This case is not a generality: in fact, the root of some tree does not refer to a living being. This is for example the case of trees drawn by Gessner, Klein or Brisson, or those developed by a phenetic method as those of Sokal & Sneath.

This living being can be considered as the ancestor of a group. This is for example the case of Hennig's, Gaudry's or Darwin's trees.

Other authors, like Lamarck, Buffon or Haeckel, do not consider this entity as the ancestor of a group, but rather as an "ancestral group" of the tree, the "type" from which descend all the others beings.

Character #4: The root refers to an inorganic object

No: 0;

Yes: 1;

An organic object is an object whose properties are specific to living beings. In contrast, an object is inorganic when it does not have these properties. Objects like rocks, water or metals are inorganic ones.

Many authors consider that the root of their trees are organic entities: Gaudry, Simpson or Lamarck, for example. However, some authors set inorganic objects in the root of their trees. Such is the case of Hitchcock's or Teilhard de Chardin's trees.

Character #5: The root refers to a set of properties

No: 0;

Yes: 1;

A "Property" is a rigorously defined attribute. This concept is similar to what we today call a "character".

The root of many trees does not carry such discretized properties: those of Lamarck, Buffon or Simpson, for example.

However, several authors, heterogeneous from a temporal point of view, set "properties" at the root of their trees. This is for example the case of the "identification keys" drawn by Gessner, Klein or Zaluziansky. But it is also found in much more contemporary trees, as those of Hennig, developed using a cladistic method, or Sokal & Sneath's ones, made according to a phenetic method setting numerized similarity on every branch, root included.

Character #6: The tree has several roots

No: 0;

Yes: 1;

A tree may have several roots.

This is for example the case of Darwin's or Lamarck's trees.

Character #7: Several trees are shown on a same picture

No: 0;

Yes: 1;

Some authors will draw several trees on a same picture. Some trees are drawn on a same page; follow one another within a same set of pictures. Each tree has its own root, its own branches and, if appropriate, its own trunk. This can illustrate the idea that certain groups of living things–plants and animals, for example–do not have the same origin. They can then not be classified in a same tree. However, the mechanisms of the evolution (or of the classification) of those groups are similar. They are then illustrated similarly.

This is for example the case of Hitchcock's trees, facing in a same picture, or of Geoffroy's ones, which follow each other in a same series.

Character #8: The living being located at the root can be practically found.

Yes: 0;

No: 1;

The roots of certain trees refer to a concrete living being. It may then be possible to find it in practice, as a living or fossil form. When trees are non-evolutionist ones, there will be species that still exist. Evolutionist authors can also set current species at the root of trees: they are then seen as "living fossils".

Finally, set on the root of their trees species concretely found in the fossil record. This is particularly true in Gaudry's trees.

Character #9: What is located at the root still exists

Yes: 0;

No: 1;

If the object at the root of the tree is a concrete entity, it exists today.

When the tree is a transformist one, this entity can be seen as the ancestor of all the others: this is particularly the case of Buffon's or Lamarck's trees. It is also found in non-evolutionary figures, such as Strickland's or Wallace's classifications by affinities.

Character #10: The root is an abstraction.

No: 0;

Yes: 1;

On some trees, the object set at the root can be reconstructed, inferred from the current data. In such a case, it is an "identikit" of this living being that is built. This is for example the case of trees of Sober. In other cases, this identity is not a hypothesis about how the ancestor would have looked like, but an abstraction that does not allow reconctruction of an identikit. This is especially the case of the phenetic trees of Sokal and Sneath, owing to the fact that an outgroup has a distance to members of the ingroup that results from a calculus.

Character #11: The root is drawn like a real tree root

No: 0;

No, but it is thick: 1;

Yes: 2;

Some authors draw the stem of their trees as a concrete one: it is thick. Roots can get out to dive into a figurative "soil". This is, for example, the case of Hitchcock's or of Haeckel's (in Anthropogenie) trees. Note that this Character is not redundant with the Character #28: a tree may have its root drawn as a real one, but not its branches. This is particularly the case of Hitchcock's trees. Conversely, a tree can have its branches drawn as real ones, but not its root.

In some other trees, the root is not drawn as a "real" one, but is nevertheless thick, drawn in two dimensions. It is as an example the case of Cuénot's or Romer's trees.

Theme: The trunk

Character #12: The trunk forbids the visualization of links between groups

Yes: 0;

No: 1;

Some trees have a trunk, that is, a central axis of the tree. It is sometimes vertical, sometimes thicker than the branches of this tree, sometimes both.

Frequently, the succession of branches on the trunk forbids to visualize properly the links between them. This is for example the case of Haeckel's trees in his *Generelle morphologie*.

However, the trunks of other trees do not hide the links between the branches. This is for example the case of the trees generated with the One Zoom software.

Character #13: The trunk shows an evolutionary direction

Yes: 0;

No: 1;

Sometimes, the trunk of the tree illustrates the idea that the succession of groups follows an evolutionary direction.

This is particularly the case of Barbançois' or Haeckel's (in. *Anthropogenie*) trees.

Character #14: The trunk shows the increase of complexity

Yes: 0;

No: 1;

The trunk of some trees illustrates the idea of an increasing complexity. In this case, the nearer a branch is to the top of the tree, the greater its complexity is.

This idea is as an example found in Haeckel's or Bronn's trees.

Character #15: Trunk shows the succession of "evolutionary grades"

Yes: 0;

No: 1;

An "evolutionary grade" is a type, a global appearance.

The trunks of some trees can show the succession of these grades. This is for example the case of most of Haeckel's trees (as those drawn in *Generelle Morphologie* or in *Anthropogenie*).

Character #16: The trunk emphasizes on the kinship links of the studied group

No: 0;

Yes: 1;

Sometimes, the trunk can be drawn to focus on a particular group: the one that is studied. In this case, despite its thickness or its verticality, the trunk is not necessarily a privileged axis.

Thus, the trees drawn by Haeckel in his *Generelle morphology* focus on the studied group.

This Character is not redundant nor incompatible with an idea of increasing complexity or succession of "grades" along the trunk.

Character #17: The trunk has an aesthetic intention.

Yes: 0;

No: 1;

The trunk of some trees has an aesthetic significance, and the way that it is drawn not only depends on a scientific idea.

Thus, the software OneZoom has this aesthetic purpose, and the trees drawn with it are colourful and thick.

Character #18: The trunk is strictly vertical

Yes: 0;

No: 1;

The trunk of a tree can be strictly vertical. This is as an example the case of the tree of mankind drawn by Haeckel in *Anthropogenie*, or of the "feather" and "natural" shapes of the trees drawn with the OneZoom software.

Character #19: The trunk is thick

Yes: 0;

No: 1;

The trunk of the tree may be thick–that is to say, to have more than one dimension. This is as an example the case of trees generated by the OneZoom software or of those drawn by Haeckel in his *Anthropogenie*.

This character, though close to the #16 one, is not redundant. Indeed, the trunk of certain trees may be thick but do not hide the links between branches. This is for example the case of the tree drawn by Trémaux. The reverse is also true: the trunk of a tree can mask the links between taxa without being thick. This is found for example on the second tree drawn by Gregory in his Fish Skulls book.

Character #20: The trunk is a branch like any other

Yes: 0;

No: 1;

The trunk of some trees may have no more value or information than its branches.

This is for example the case of the trees drawn by Haeckel in his *Generelle Morphologie* or by Augier in his *Essai d'un Arbre Botanique*.

This Character is independent of the characters 13, 14 and 15. Indeed, on Bronn's or Haeckel's (in. *Anthropogenie*) trees, only the trunk shows the idea of an increasing complexity: this one is not carried on the branches of the picture. Then, the trunk is not a branch like any other.

On the opposite, Augier's or Haeckel's (in. *Generelle Morphologie*) trees show the same kind of information on their trunk and on their branches. The trunk, despite its form, has the same properties as the branches.

Character #21: what is carried by the trunk is concretely detectable

Yes: 0;

No: 1;

The objects set on the trunk can sometimes be concretely found: in the fossil record or in living groups or species.

This is as an example the case of the tree drawn by Haeckel in *Anthropogenie*. There are, on the trunk of the tree, still existing groups as "Dipneusta" or "Vermes". Present worms or lungfishes aren't seen by Haeckel as the ancestors of other present species, but the group by itself is considered as a "type", a typical form that had the ancestor of another present group. Thus, lungfishes are seen as the ancestral form of tetrapods.

Theme: Branches

Character #22: Branches show groups which follow one another

Yes: 0;

No: 1;

The branches of the tree may show successions of groups. We consider that groups follow one another when the following is not included in the previous one: it is then possible to get out of a group. For example, when considering that the birds "descend" from reptiles. We then say that the groups are arranged hierarchically under constraint of order.

Groups in trees of Haeckel, Lamarck or Romer follow one another on the branches.

This is however not the case with those drawn up by Darwin or by Hennig.

Character #23: Some branches show a purpose to which the evolution leads

No: 0;

Yes: 1;

The branches of some trees show an evolutionary purpose. This is particularly the case of Teilhard de Chardin's trees.

This Character is not redundant with the Character #14. This purpose can indeed be carried by the trunk but not by the branches. This is for example the case of Haeckel's tree (in *Anthropogenie*). But it can also be carried by the branches and not by the trunk. This is particularly the case of Teilhard de Chardin's trees in *Le Phénomène Humain* or of Romer's ones in his "Major steps in vertebrate evolution '.

Character # 24: Some branches show a change in shape

No: 0;

Yes: 1;

Some branches may illustrate the shape variation in a group. When one branch passes to another one, it means that the shape of an individual or of a group, his "type" has changed.

This is particularly the case of Romer's, Gregory's, Haeckel's or Lamarck's trees.

Character #n° 25: What's on the branches is actually detectable

Yes: 0;

No: 1;

Some authors consider that the objects set on the branches can be concretely detected: in the fossil record, for example, or among living species.

The branches of Haeckel's tree (in Anthropogenie) or of Lamarck's one have such properties.

This Character is not redundant with the eventual presence of detectable objects at the root of the tree: in Romer's trees, for example, objects in the root are not actually detectable, but those at the branches are.

Character # 26: What's on the branches still exists today

Yes: 0;

No: 1;

Some items set on branches may still exist today.

This is particularly the case of Lamarck's tree.

Character #27: Branches have an aesthetic significance

No: 0;

Yes: 1;

The branches of some trees may have an aesthetic significance. This means that their shape, orientation or even colour have an aesthetic function. But these branches are however not necessarily drawn as real tree branches.

This property is found in the Haeckel's trees, but also on those of the OneZoom software.

Character #28: Branches are drawn as real tree branches

No: 0;

No, but they are thick: 1;

Yes 2;

The branches of a tree can be represented as real ones.

This is for example the case of the branches or Gruenberg's or Haeckel's (in *Anthropogenie*) trees.

The branches of other trees can be just thick without being drawn as real tree branches. This is particularly the case of those developed through OneZoom software.

This Character is not redundant with the previous Character #27: only trees with branches drawn as real tree branches have *a priori* aesthetic purposes. The branches of Agassiz' tree, for example, are thick without having an aesthetic purpose. On the opposite, trees drawn using the OneZoom software have a strong aesthetic purposes but are not drawn like real trees.

Character #29: Branches express discretized properties

Yes: 0;

No: 1;

Tree branches can express discretized properties (the definition of this term is identical to the one given on the Character #5).

This is for example the case of the "key" trees made ​​by Gesner, Klein, or Zaluziansky but also of Hennig's or Darwin's trees.

Character #30: Branches express a degree of similarity

No: 0;

Yes: 1;

Some methodologies for the elaboration of the tree classify the objects according to their degree of similarity. This is particularly the case of phenetic methods.

Thus, the trees of these authors, including Sokal & Sneath or Fitch, express relative degrees of global similarity between groups or individuals.

This Character is not redundant with respect to the preceding Character (29). The four possible combinations implied by these characters are in fact represented in the corpus, as shown below:

Discretized properties and degrees of similarity in some author's methodologies:

Degrees of similarity and discretized properties: Mayr 1974, Tillyard 1921;

Degrees of similarity and no discretized properties: Sokal & Sneath, Fitch;

No degrees of similarity and discretized properties: Hennig, Zaluziansky;

No degrees of similarity and no discretized properties: Haeckel, Bronn.

Character #31: The branches show an evolutionary path

No: 0;

Yes: 1;

Sometimes, tree branches show an evolutionary path. This notion doesn’t necessarily involve a teleological finality that would direct evolution: an evolutionary path can be induced by natural laws whereas finality only possesses supernatural causes.

For example, Lamarck’s tree shows such an evolutionary path. The species follow indeed the same overall progress in the successive rises of their properties. However, these rises are definitely not teleological but rather linked to natural principles, the product of a historical process.

Character #32: The branches show affinity links

Yes: 0;

No: 1;

“Affinity links” are links showing the resemblance between various objects. For example, a bat and a bird can share an “affinity link” because they both possess wings. A pigeon and a tit have many other “affinity links”. Besides, those links are not necessarily defined.

Numerous classificatory figures of the 19th century use this notion of affinity links between species. Among them are Strickland or Wallace. Those trees are clearly defined as such. However, this notion can also be extended to today’s phenetic trees which use distance methods. This is the case of the trees created by Sokal & Sneath or Fitch, even of non-rooted networks.

Character #33: The branches can rotate around the nodes

No: 0;

Yes: 1;

The branches of some trees can freely rotate around the nodes without modifying the reading of the tree.

This is especially the case of the trees of “identification key” created by Gessner, Klein or Geoffroy. But it is also found in Gaudry or Darwin’s trees, in Hennig’s cladograms or in Sokal & Sneath’s phenograms.

However, it should be noted that it is not the case in trees found in OneZoom software: if the content can potentially be carried by the trunk or by the branches, the form of the tree itself prevents the branches from rotating with one another.

Theme: The nodes

Character #34: The layout of the nodes shows a finality to which evolution is tending

No: 0;

Yes: 1;

Within some trees, the layout of the nodes itself shows a finality to which evolution will tend. This is reflected in the verticality or the height of the series of nodes. This is especially the case of Barbançois and Chambers’ trees. But this can also be an unfortunate interpretation of the trees created using the OneZoom software: they are also considered as possessing this character.

Character #35: The nodes correspond to a set of discretised properties

No: 0;

Yes: 1;

The nodes of some trees can correspond to a set of discretised properties. The definition of this term is identical to the one given for Character #5.

However, this Character is neither redundant with Character #5 nor with Character #29. Indeed, Wallace and Strickland create affinity trees and place definite and familiar species on their nodes. As a result, they carry no discretised properties. Nonetheless, the later are carried by the branches of their trees. The nodes of trees created by other authors don’t correspond to a set of discretised properties but to a degree of global similarity between various objects. This is especially the case of trees created following a phenetic method.

On the other hand, several authors locate those discretised properties in the nodes of their trees. Some even define them a priori. This is especially the case of Gessner or Zaluziansky’s trees. Others such as Hennig or Garrod algorithmically maximize their distribution.

Character #36: The nodes correspond to a percentage of similarity based on the characters

No: 0;

Yes: 1;

The nodes of some trees can correspond to degrees of global similarity based on characters. In this way, if the construction of the tree is truly based on the study of the characters, the trees are in this case only analysed as a whole.

We can especially find this particular instance in trees reconstructed using distance methods. This is the case of Saitou & Nei or Sokal & Sneath’s trees.

Character #37: The nodes correspond to an ancestor

No: 0;

Yes: 1;

Some authors match the nodes of their trees with an ancestor.

The latter can be considered as definite and searched for in the fossil record. Some authors such as Gaudry even assign the ancestor status to this or that fossil. On the other hand, other authors give the ancestor status to the nodes of their trees without searching for it in the fossil record. This is especially the case of the cladistics method.

Character #38: The nodes correspond to a type

Yes: 0;

No: 1;

A “type” is an overall shape, an overall morphology, a Bauplan. Some authors classify those “types” on the nodes of their trees. This is the case, for example, of Haeckel’s trees in *Generelle morphology*, and of Romer’s trees.

Character #39: The entity located at the nodes can be detected materially

Yes: 0;

No: 1;

When the nodes of a tree correspond to an entity (species, individual, group…), the latter can be detected materially: in the fossil record, for example, or as a present individual.

This is the case, for example, of Gaudry who establishes the genealogies of fossils in his trees. This is also the case of Duchesne or Buffon who placed species or current shapes at the nodes. This is ultimately what some authors of more recent trees, such as Romer or Stirton, are doing by classifying the fossils in relation to each other in their trees.

Character #40: The entity located at the nodes is reconstructed

No: 0;

By analogy: 1;

By the characters 2;

Sometimes, the entities located at the nodes of the trees are not–or cannot be–found materially. In this case, they can possibly be reconstructed. This reconstruction can be done either by analogy or by the characters.

When the reconstruction is carried out “by analogy”, the shape located at the nodes is reconstructed according to an idea of the overall shape. A series of types are imagined and, by analogy with the current types; we deduce the type of the group’s ancestor. This method is the one used by Haeckel, Romer or Lamarck.

On the other hand, when the shape at the node is reconstructed by characters, shared features had previously been detected among the species compared. If the node is in the tree, this is because this node is supported by a number of shared features located on it. This is for example the method used by Darwin, Hennig or Delacour & Mayr.

Theme: The leaves

Character #41: The leaves contain individuals

No: 0;

Yes: 1;

The trees can sometimes classify species, larger groups or smaller groups such as subspecies or individuals. As a result, some authors classify individuals on their trees. The reason is that we only sample a set of individuals belonging to the species that we are studying.

Some authors such as Sokal & Sneath, Hennig or Sober draw leaves as such.

Character #42: The leaves show discretized properties

No: 0;

Yes: 1;

The objects classified on leaves can be seen as sets of discretized properties, as defined at Character #5.

This Character is not redundant with the fact that the leaves contain or not individuals. Indeed, some authors such as Garrod classify groups with a rank higher than the rank of the species, but split them up in series of properties that will be classified.

It is also not redundant with characters 5, 29 and 35. Indeed, some authors who, such as Strickland or Wallace, create affinity trees between definite species, don’t locate discretized properties at the leaves or at the nodes of their trees but only locate them at the branches. Other more contemporary authors group leaves that are defined as sets of discretized properties, avoiding that the nodes themselves are a set of those properties. Those authors create sophisticated trees following the phenetic method. Finally, some authors such as Dollo (p.89) locate definite species at the leaves of their trees without linking them to a set of properties and locate instead sets of discretized properties at the nodes of their figures. Variations among authors can be described as:

Discretized properties on leaves but not on nodes: Sokal & Sneath;

Discretized properties on nodes but not on leaves: Dollo;

Discretized properties on leaves and nodes: Hennig;

Discretized properties nowhere: Strickland, Wallace.

Discretized properties are especially located in “identification” trees such as the ones created by Gessner or Klein. They are also found on very contemporary trees such as Margulis, Sokal & Sneath, Darwin or Hennig’s trees.

Character #43: The leaves express an evolutionary fate

No: 0;

Yes: 1;

The leaves of some trees show an evolutionary fate. In general, this is correlated to the text describing the tree. Such leaves are generally organized vertically so as to show this fate. The more a leave is located far from the root, the more the species that it carries is considered as advanced in its evolution.

This is especially the case of Teilhard de Chardin, Barbançois or Chambers.

Character #44: The leaves have an aesthetic impact

No: 0;

Yes: 1;

Some authors draw the leaves of their trees as real leaves. Others give them a configuration, a shape or a colour that only aims at the aesthetic of the figure. These leaves are considered as having an aesthetic impact.

This is especially the case of Augier’s tree, where the leaves are drawn to look like real tree leaves. This can also be found in trees created using the OneZoom software.

Character #45: The leaves are thick

No: 0;

Yes: 1;

The leaves of some trees are thick: they are not single dots but are drawn in several dimensions.

This is for example what we can find in Romer or Agassiz’s trees, or in Haeckel’s book *Anthropogenie*.

Character #46: The tree is reconstructed from the leaves only

No: 0;

Not only them: 1;

Yes: 2;

Nothing apart from the leaves is known nor classified in the tree. The remainder of its objects–nodes, branches, root, and topology–is reconstructed from the study of the leaves only.

This is how cladists or pheneticians create their trees: they only use the leaves to do it (coded 2).

Others authors, such as Haeckel or Romer, while using the leaves to reconstruct their trees, do not limit themselves to the leaves but place other objects at the branches or at the nodes (coded 1). Gaudry construct his fossils genealogies without depending on what he places at the leaves of its trees (coded 0).

Character #47: The objects located at the leaves are the only classified objects

No: 0;

Yes: 1;

Some authors only classify objects that they locate at the leaves. No classified object are located elsewhere (root, branches).

This is especially what is done by authors such as Gesner, Klein, Delacour & Mayr or Hennig, or by the OneZoom software.

Theme: The bubbles

Character #48: Bubbles enable to visualize the quantity of species according to the time

No: 0;

Yes: 1;

A bubble is a two-dimensional object which replaces the branches of a tree or is placed on top of the branches of a tree. The thickness of the bubble carries information about the quantity of species known from a group at a given time. Doing so, bubbles show the group’s diversification or decline along periods of time.

This is for example what can be found in Agassiz, Romer or Gregory’s trees.

Character #49: Bubbles enable to visualize an evolutionary gradation

No: 0;

Yes: 1;

An evolutionary gradation is both a growing complexity and an increasing improvement of shapes that succeed one another. This can be found in the succession of bubbles.

This is especially the case of Cuénot or Romer’s trees. This is however not the case of Copeland or Agassiz’s trees.

Character #50: Bubbles enable to visualize the types, the typical shapes

Yes: 0;

No: 1;

The bubbles of a tree can correspond to types. The succession of bubbles is then the succession of types according to the classification. This notion is not necessarily linked to an idea of growing complexity.

This is the case of almost all trees with bubbles in the study: Hitchcock, Agassiz, Romer or Simpson. However, this is not the case of Copeland’s tree.

Character #51: Bubbles enable to visualize similar ecosystems

No: 0;

Yes: 1;

The bubbles of some trees can represent the fact of living in the same ecosystem: aquatic, terrestrial, etc.

This is for example the case of Patten’s tree.

Character #52: Bubbles are covering the branches

No: 0;

Not all of them: 1;

Yes: 2;

The bubbles of some trees are an indivisible element of the figure. In that case, they don’t cover implied branches. This is for example the case of Augier and Copeland’s trees, where the bubble is an element as such, without implicit underlying tree structure.

Other authors draw bubbles to cover a tree simplification, drawn or implied. Bubbles can then only cover some branches of the tree, such as the tree drawn in the inside front cover of Margulis and Schwartz’s book, or cover all the branches of the tree.

Axis #2: Spatial inscription of the tree

Theme: The hierarchical and diversification axes

Character #53: The hierarchical axis shows a gradation of values

Yes: 0;

No: 1;

The hierarchical axis of a tree shows the orientation according to which the hierarchy of the tree’s groups is organized. It spreads from the root to the leaves of the tree.

This hierarchical axis can bear an idea of gradation of values: this is the case of Haeckel, Buffon or Romer. For those authors, there is a variation in the level of complexity and/or perfection of the living beings according to the distance existing from the root of the tree.

Character #54: The hierarchical axis shows nested groups

No nesting: 0;

Amongst other things: 1;

Strict: 2;

By definition, the hierarchical axis shows nested groups. However, this is not always the case.

For example, with Duchesne, Lamarck or Buffon, there is no nesting of the groups according to the hierarchical axis.

Character #55: The hierarchical axis shows a succession of groups

Yes: 0;

No: 1;

Within a tree, the hierarchical axis can also show a succession of groups. This is not redundant with the previous character. Indeed, the succession of groups doesn’t prevent a potential nesting. Quite the opposite, the two cases can superimpose on each other. This is for example the case of Mayr’s trees: the groups are nested and follow each other on it. Reptiles and birds are both vertebrates but the birds follow the group of the reptiles.

With Lamarck, Gaudry or Buffon, the groups follow each other on the hierarchical axis without nesting each other. Mammals follow reptiles but they are not part of them. With Darwin or Hennig, the groups are nested but are not following each other. Ultimately, for authors such as Agassiz, the groups are absolutely independent from one another and are neither following each other nor nesting.

Distribution of the authors according to the potential nestings and successions of groups can be described as follows:

Succession of nested groups: Mayr;

Succession of groups, not nested: Lamarck, Gaudry, Buffon;

No succession, groups nested: Darwin, Hennig;

No succession, not nested groups: Agassiz.

Character #56: The hierarchical axis shows a living environment

No: 0;

Yes: 1;

With some authors, the hierarchical axis can show a living environment. In consequence, the species are located farther or closer from the root according to the place where they live.

This is for example the case of Cuénot’s tree: a barrier divides the aquatic living environment (bottom, close to the root) from the terrestrial environment (top, close to the tips). The return to the aquatic environment made by the cetaceans is symbolized by a branch going down.

Character #57: The hierarchical axis shows complexity levels

Yes: 0;

No: 1;

A complexity level refers to the complexity of the organism under study. The latter can be interpreted from the number of organs, for example, or from the capacities of the nervous and cognitive system.

The hierarchical axes of Haeckel or Stirton’s trees, for example, show an increase in the complexity level.

Even though they are often associated with one another, the complexity levels and the gradation of values are not the same notions, as shown here:

Gradation of values and complexity levels: Haeckel;

Gradation of values and no complexity levels: Buffon;

No gradation of values and complexity levels: Stirton;

No gradation of values and no complexity levels: Darwin.

The “evolutionary gradation” implies an idea of growing improvement. A species can be considered as more complex than another without necessarily being seen as more perfect. And, on the contrary, a species can be seen as more perfect than another without necessarily being more complex. As a result, the hierarchical axes of Darwin or Hennig’s trees show neither a complexity level nor a growing complexity. However, for Buffon or Duchesne, the hierarchical axis shows a gradation of values but no change in the complexity level: the degenerated species is not less complex than the one which gave birth to her, it is only less perfect. On Stirton’s tree, on the contrary, the hierarchical axis shows complexity levels but no gradation of the values. Ultimately, Haeckel’s trees show both complexity levels and gradations of values.

Character #58: The hierarchical axis shows the diversity of shapes

Yes: 0;

No: 1;

The hierarchical axis of the tree can show a diversity of shapes: in such cases, it illustrates either the appearance of the big “types” of living beings or their distribution in the idealistic continuity along the chain of living beings.

Haeckel or Teilhard de Chardin’s trees carry on their hierarchical axes this idea of appearance of the types. The diversity of shapes within non-evolutionary trees is in addition illustrated on Augier or Wallace’s figures.

Character #59: The hierarchical axis shows quantifiable data

Yes: 0;

No: 1;

The data are said to be “quantifiable” when it is possible to measure them and to represent them in a formalised manner. This consists in formalising the lack or presence of a character, the measure of a length, a number (of legs, of cardiac chambers …) or even a similarity rate. The hierarchical axis shows quantifiable data when it displays a succession of such information.

In this way, the hierarchical axis of Gesner or Klein’s trees shows quantifiable data: the different points of characters’ hierarchy. But this is also the case of Hennig, Sokal & Sneath or Delacour & Mayr’s trees.

Character #60: The hierarchical axis shows discretized properties

Yes: 0;

No: 1;

The hierarchical axis of the tree can refer to discretized properties.

This is especially the case of the trees of identification keys, but also of Hennig, Garrod or Tillyard’s trees.

However, trees created following phenetic methods, such as Sokal & Sneath’s trees, don’t have discretized properties on their hierarchical axes. Indeed, there are no characters but degrees of similarity that are carried by this axis.

Character #61: The hierarchical axis shows a degree of global similarity

No: 0;

Yes: 1;

The hierarchical axis of the tree can show degrees of global similarity, that is to say a quantity of similarities between entities.

This is the case of the trees created using distance methods, such as Sokal & Sneath or Fitch’s trees, but also such as Wallace and Strickland’s trees.

Character #62: The hierarchical axis shows a temporal order

No: 0;

Strict: 1;

Relative: 2;

The hierarchical axis of the tree can carry a constraint of temporal order. The latter can be strict or relative. A strict temporal order respects the sequence of events. If the present is located at the top of the tree, a node occurring after another node cannot be located under the latter. In the same way, a present group cannot be located higher than another present group.

Darwin, Lamarck or Hennig’s trees carry strictly respected constraints of order.

However, with other authors, the constraint of order is relaxed: temporal order is relative. The position of the various objects on the hierarchical axis is not directly linked to the temporal sequence and a present group can succeed another present group. This is for example the case of Cuénot, Buffon or Haeckel’s trees.

Character #63: The hierarchical axis shows the time as dates

No: 0;

As horizontal undated lines: 1;

Yes: 2;

The hierarchical axis can carry absolute time. Some authors represent it under the form of dates which are determined thanks to fossils or to important geological events for example. In consequence, the graph of the tree can directly superimpose the dates to the links between species.

This is for example what Agassiz, Gregory or Copeland do.

However, other authors, such as Hennig or Lamarck, don’t represent directly the dates of the events on their trees.

Ultimately, some authors such as Darwin don’t show time as dates. However, the tree carries undated temporal lines. These enable to synchronise specific events.

Character #64: The diversification axis shows reversible changes

No: 0;

Yes: 1;

The diversification axis of a tree is the axis representing the diversification of the groups. Contrary to the hierarchical axis, its extent doesn’t depend on the level of resolution of the tree. An fully unresolved tree has the same level of diversification than a fully resolved tree of the same number of taxa. By contrast, the hierarchical axis of an unresolved rooted n-species-tree has an extent of zero while the hierarchical axis of a fully resolved rooted n-species-tree has an extent of n-2: the value of the hierarchical axis depends on the level of resolution of the tree.

The diversification axis of some trees can show reversible changes. They are individual or accidental variations of a global type which can be reversible. The animal which undergoes a variation can come back to its initial type if the conditions of the environment change.

This is especially the case of Lamarck’s tree.

Character #65: The diversification axis shows complexity levels

No: 0;

Yes: 1;

The diversification axis of a tree can sometimes be oriented. In that case, it can mark a variation of the complexity levels.

In this way, the tree itself shows a classification of the species but the diversification axis arranges species according to their complexity. Branching points of the tree are arranged according to a comb (“Peigne” is the literal translation in French but “arbre-échelle” (“scale-tree”) is a more suitable translation).

The diversification axes of several trees show such a variation in complexity degrees. This is especially the case of Augier’s tree, but also of Tillyard or Romer’s trees and of Haeckel’s tree in *Natürliche Schöpfungsgeschichte*.

Character #66: The diversification axis shows a similarity rate

No: 0;

Yes: 1;

The diversification axis of the tree can show a similarity rate between several objects. The more two leaves or two nodes are distant from one another on this axis, the less they are similar.

The trees with bubbles created by Romer in his *Vertebrate Paleontology* or the trees drawn by Simpson in his Principles of animal taxonomy show such similarity rates according to the diversification axis.

Character #67: The diversification axis shows a biogeographical distribution

No: 0;

Yes: 1;

Sometimes, the diversification axis of a tree shows the environment of a species: country, geographical zone or environment.

This is the case of Buffon’s “*La table des chiens et de leurs variétiés*” in the *Histoire Naturelle*.

Character #68: Discontinuities on the hierarchical axis

No: 0;

Yes: 1;

The diversification axis of the tree is never continuous except in the case of cross-links. The groups are separated from one another.

The hierarchical axis of the tree, on the other hand, is generally continuous. But with some authors, it is not the case, especially in Agassiz’s trees. Indeed, this author doesn’t see any genealogy of the species that he classifies but only cycles of supernatural creations and extinctions. In consequence, there can be no hierarchical continuity in his temporal tree.

Character #69: Vertical “cuts”

No: 0;

Yes: 1;

Some trees are cut by vertical lines. These lines separate arbitrarily two parts of the tree. They are found in several of Haeckel’s trees, among which the first tree of his *Generelle Morphologie*, but also the fourteenth tree of his *Natürliche Schöpfungsgeschichte*.

Theme: The dimensions (in the mathematical sense of the term)

The term of “dimension” is to be considered in the sense of the “mathematical dimensions” in which the tree is contained. A dot, for example, is an object without dimension. A line only has one dimension. A leaf or a plant are two-dimensional objects.

Character #70: Number of dimensions of the tree

Two: 0;

Three: 1;

A scale of the living beings is a strictly linear object and in consequence, it only has one dimension. Most trees under study are two-dimensional. However, some authors use, by perspective effects, a three-dimensional view.

Among the later are Haeckel’s tree in Anthropogenie and Copeland’s tree.

Character #71: The tree needs more dimensions

No: 0;

Yes: 1;

In order to be read, some trees need to possess more than three dimensions. This is depicted, on the figure, by using different lines: dotted lines, colours, etc. The figures still possess a root, leaves, branches or nodes. They still are trees.

This is especially the case of Buffon’s tree of dogs.

Axis #3: The tree as a science-based approach of the world

Theme: The ancestor

Character #72: The ancestor shows an order of appearance

No: 0;

Yes: 1;

On some trees, the ancestor can show a history: the history of the successive transitional forms until the present time. In this case, the ancestor shows an order of appearance: the ancestor of mankind, for example, is the “ape” type, then the “mammal-like reptiles” type, etc. Warning: when the ancestor of a group shows an order of appearance, this doesn’t involve a consideration of values.

Haeckel or Gregory’s trees show such orders of appearance.

Character #73: The ancestor is a less adapted state of a group

No: 0;

Yes: 1;

Some authors see in the ancestor of a group a less adapted state, less perfect than all or parts of its present representatives.

This ancestor doesn’t necessarily show an order of appearance between typical forms. In this case, for Buffon, the ancestor only gives the order of appearance and doesn’t show a less adapted state. For Haeckel however, the ancestor is both the mark of an order of appearance and a less adapted state of characters. Ultimately, for Gaudry or for Delacour & Mayr, the ancestor only shows an order of appearance and isn’t a less adapted state of the characters.

Character #74: The ancestor can be detected effectively

No: 0;

Yes: 1;

Trees depict interrelationships among entities from he real world. For some authors, some of these entities can be considered as ancestors.

This is especially the case of Buffon, Duchesne, Lamarck or Gaudry’s trees.

This Character is not redundant with Character #39: indeed, all the nodes don’t necessarily carry an ancestor. In this way, with Augier, Strickland or Wallace, who create non-evolutionary trees, what is carried by the nodes can be detected effectively (they are entities of the real world) but the ancestor cannot. Indeed, there is no ancestor at the nodes. On the contrary, in Tillyard’s article from 1919 (“*The panorpoïd complex*”), entities that can be detected are seen as ancestors but are not located at the nodes which are considered as reconstructions of characters states. Links between nodes, ancestors and entities that can be described as follows:

Objects effectively detectable can be ancestors, placed on nodes: Gaudry;

Objects effectively detectable placed on nodes (not as ancestors): Augier;

Objects effectively detectable can be ancestors, but not placed on nodes: Tillyard 1919;

Objects effectively detectable are not ancestors, and not placed on nodes: Hennig.

Character #75: The ancestral morphotype still exists at the present time

No: 0;

Yes: 1;

The ancestral morphotype is the overall form, the Bauplan that is supposed to represent the ancestor of a group. Some authors consider that this morphotype can be found in some present species.

This is especially the case of Buffon and Duchesne who even consider that representatives of present species are the ancestors of other species–the horse and the donkey for example. Romer or Haeckel’s trees also display a persistence of the ancestral morphotype in present species.

Character #76: The ancestor is:

A group of superior rank: 0;

A species: 1;

An individual: 2;

The taxonomic rank that is given to the ancestor of a group can vary.

Some authors consider that the ancestor is a group with a rank superior to the rank of the species understood in the sense of interbreed individuals. This is especially the case of Romer, Haeckel or Teilhard de Chardin. Other authors consider that the ancestor of a particular group is one or some individuals: Haeckel, Darwin or Hennig for example. Ultimately, several authors consider that the ancestor of a group is a species as a whole, understood in the sense of interbreed individuals: this is especially what Buffon or Duchesne do.

Theme: Place of mankind

Character #77: Special place for mankind

Yes: 0;

For a group to which mankind belongs: 1;

No: 2;

The authors can sometimes give a special place to human beings who then have a privileged classification in the tree.

This is especially the case of Haeckel’s tree in *Anthropogenie* or of his eighth tree in *Generelle morphologie*.

Other authors can give a special place not to mankind but to a group which carries it: primates, mammals, vertebrates. This is for example the case of Lamarck’s trees or of Haeckel’s seventh tree in *Generelle morphologie*.

Ultimately, several trees don’t give any special place to mankind: Darwin or Hennig’s trees for example. This Character is coded as “irrelevant” if the tree doesn’t carry any human beings.

Character #78: Hierarchy of human groups?

No: 0;

Yes: 1;

Some authors distinguish, within their trees, groups of human beings that they will later classify hierarchically.

This is for example what Haeckel does with his tree of human migrations or what Barbançois does with his *Observations pour servir à une classification des animaux*. This Character is not redundant with the previous Character because a tree can classify human beings while placing mankind at a special place in the tree, whereas other trees cannot locate men at a special place while classifying them in relation to each other.

Theme: Consistency of the groups

Character #79: Members of a group have features in common

Yes: 0;

No: 1;

When making groups, authors can use several means. Some authors justify their groups by the fact that members share features: feathers, wings, scales, swimming, etc. These features are not necessarily derived characters or morphological traits. Here the term “features” is to be taken in its widest meaning.

In that way, the groups created by Haeckel or Hennig are based on a shared features.

Character #80: Members of a group share a typical form

Yes: 0;

No: 1;

The groups of a tree can be justified by the fact that members share a typical form, a Bauplan.

This is for example the case of Haeckel, Romer or Delacour & Mayr’s trees.

Character #81: Members of a group have a common ancestor

No: 0;

Yes: 1;

The groups of a tree can be justified by the fact that members have a common ancestors. Several species are then grouped if we believe that they have a kinship.

This is the case of the groups created on Darwin, Romer, Buffon or Haeckel’s trees.

Character #82: Members of a group share a level of complexity

Yes: 0;

No: 1;

The groups of a tree can correspond to an idea of complexity level. This notion, as it has been defined in Character #57, is different from the notion of “grade”. Haeckel, Romer or Teilhard de Chardin create their groups according to their level of complexity.

Character #83: Groups are tested

No: 0;

Yes: 1;

No matter how the groups were created, they can sometimes be tested. The purpose here is to check if all the organisms that should belong to the group are actually present in it, and if no unnecessary organism has been added to it.

Several authors test their groups: Patten, Hennig, Sokal & Sneath or Garrod.

Character #84: The groups are an arbitrary convention

No: 0;

Yes: 1;

For some authors, the groups are only a human construction and do not necessarily need to reflect a natural hierarchy. If they create them, it is by convention and for language simplicity.

We are here talking of Duchesne, Buffon and Lamarck. With other authors such as Darwin, on the contrary, the groups are not, even implicitly, arbitrary but must reflect a natural hierarchy, i.e. they must meet a set of accurate rules related to genealogy.

Character #85: The groups are the basis of the classification and must be linked to one another

Yes: 0;

No: 1;

Some authors define groups *a priori* and then classify them in relation to each other.

This is for example the method used by Teilhard de Chardin, Romer or Haeckel.

Character # 86: The groups have a practical vocation

Yes: 0;

Possibly: 1;

Groups can permit the identification of species or, alternatively, of the group to which it belongs, even if the tree that support them was not constructed for that purpose. In other cases, trees are explicitely dedicated to this purpose: determination keys, for example. In such cases they have a practical vocation (coded 0).

Trees developed by Klein, Zaluziansky or Gesner are such ones.

But in other trees, such as Hennig's or Sokal & Sneath's, identification is a possible consequence of the classification and have no practical purpose.

Character # 87: The groups can be modified if new data require it

No: 0;

Yes: 1;

When groups are elaborated, they can be used to support the assignment of a new form. For some authors, all forms should even be assigned to an existing and irremovable group of life.

This is particularly the case for Haeckel's or Romer's trees.

In contrast, the "identification keys" drawn ​​by authors such as Zaluziansky or Gessner separate groups of beings into sets of properties that can be modified if needed. Although the process is different, it is the same with authors such as Hennig, Sober or Sokal & Sneath. Here, groups are directly read from the tree and are likely to change if new information is provided.

Character # 88: The tree gives the groups

No: 0;

Yes: 1;

For some authors, groups of life do not exist *a priori*. Instead, they are given by the tree.

These authors are, for example Darwin, Hennig, Sokal & Sneath or Lankester.

This character is different from the character 84, as an author can consider that groups are not arbitrary, without expecting the tree to give those ones. This is for example the case of the trees developed by Haeckel. Details of possible situations are:

The tree gives the groups which are an arbitrary convention: Buffon;

The tree gives the groups which follow a natural hierarchy: Darwin, Hennig;

The tree does not give the groups; groups are an arbitrary convention: Lamarck;

The tree does not give the groups; groups follow a natural hierarchy: Gesner, Haeckel.

Theme: Continuity and discontinuity on the tree

Character # 89: Scalist reading?

Yes: 0;

No: 1;

Several trees can, despite their structure, be read like as scales of beings.

This scale can be found at several levels: on the trunk of the tree, as Haeckel's one in *Anthropogeny*; in the nesting of the branches, as found in the first tree drawn by Haeckel in his *Generelle Morphology*, or even on the diversification axis (see definition above) of the tree. This is particularly the case of Barbançois' tree in his "*Observations pour servir à une classification des animaux"*.

Character # 90: Reticulations?

No: 0;

Yes: 1;

A reticulation is a branch drawn horizontally, connecting together two branches of a tree. It makes the tree graph become a cyclic connected graph: then there are several possible "paths" to go from one node to another.

Reticulations are found in Buffon's, Duchesne's or Schrank von Paula's trees.

This character is different from the character #71, which identifies trees represented in more than two dimensions. Indeed, it is possible for a tree to be represented in three dimensions without being reticulated.

Theme: Elaboration of the groups

Character # 91: Groups are:

Interlinked: 0;

Independent: 1;

Groups can be linked together by relations of inclusion (nesting) or succession. This is the case of most of the authors we studied: Romer, Darwin, Haeckel, Buffon.

Groups can also be completely independent the ones from the others: it is for example the case of Agassiz' tree. For this author, groups can neither be included, nor succeed the ones to the others since each of them is an independent divine creation.

Character # 92: Delimitation of groups

Vague: 0;

Well defined: 1;

Within trees, groups can be clearly defined, or remain vague in their definition.

Thus, established by Darwin, Hennig, Sokal & Sneath, Klein and Gessner groups are well defined. It is easy to distinguish what belongs to a group from what does not.

However, in Romer, Haeckel or Gregory, group boundaries are blurred. It's hard to say where the limits the group are.

Character # 93: Elaboration of "complete" groups?

Yes default program by chance: 0;

Yes, as a result of a classification program: 1;

A group is said "complete" when it groups to a node all the nodes and branches derived from it. In an evolutionary thinking, this corresponds to a "monophyletic" group. Mathematically speaking, no tree can be drawn without at least one complete group. Thus, some authors can create "complete" groups only by chance, default program. This is for example the case of Romer's , and Haeckel's trees.

Other authors, however, create such complete groups as a result of their classifying program. This is for example what do Hennig, Darwin or Delacour & Mayr.

Character # 94: Formation of incomplete groups.

Yes, due to methodological imprecision: 0;

Yes, because of a classification program: 1;

No: 2;

A group is said "incomplete" when it does not include all the branches derived from its node.

Some authors develop incomplete groups as a consequence of the vagueness of their methodology. This is particularly the case of the tree published by Delacour & Mayr. The basis of the tree is formed by an "incomplete" group, and the rest of the tree—the majority of it—is made of complete groups.

Other authors however clearly elaborate their classifications as a chain of groups succeeding each other, and therefore each of them being incomplete—except the latest. This is for example the case of Haeckel's, Mayr’s or Romer's trees.

Finally, several authors such as Darwin or Hennig, refuse the formation of incomplete groups.

This character is not redundant with the previous character (#93). Indeed, several authors can produce complete and incomplete groups as a result of their classificatory program. This is for example the case of Mayr in his article "*Cladistic analysis or cladistic classification*?". The reverse case is found with the tree drawn by Haeckel p.477 its *Generelle Morphologie der organismen*: all groups are complete and there are no incomplete group. However, the reading of the text and the review of other trees shows that the formation of such complete groups is here due to chance.

Character # 95: Elaboration of 'heterogeneous' groups

No: 0;

Yes default program by chance: 1;

Yes, as a result of a classification program: 2;

A "heterogeneous" group is formed of various species non-directly connected one to another. In evolutionary terms, it is called "polyphyletic group."

Some writers, such as Teilhard de Chardin in his *Phénomène Humain* (p.77) create such groups by chance.

Other authors, like Mayr, in his *"Cladistic analysis or cladistic classification*? *"* (p.103) article, form heterogeneous groups as a result of a classification program to focus on adaptive convergence.

Finally, some trees, as Darwin's ones will have no heterogeneous group.

Character #96: The groups are elaborated following a specific method

No: 0;

Yes: 1;

Are groups formed according to a particular methodology, detailed in the book?

Neither Lamarck nor Wallace nor Romer, for example, described in the text or in the figure the way they elaborated their groups.

Conversely, Klein's, Gesner's or Haeckel's trees detailed the characters which support their groups. Authors like Sokal & Sneath or Hennig developed explicit methodologies to create groups.

Theme: Ranks and trees

Character #97: Do classificatory ranks follow the tree shape?

Yes: 0;

No: 1;

Trees and classifications are often linked. It can be the same with classificatory ranks that can follow the classification given by the tree.

Such is the case with authors like Darwin, Haeckel and Romer. Regardless of how the tree and the groups are elaborated, classificatory ranks follow this figure.

However, this is not the case of Lamarck, an author according to whom classificatory ranks are arbitrary.

Theme: Why a tree?

Character #98: The tree aims at depicting the order of nature

Yes: 0;

No: 1;

By establishing a classification of life, many authors seek to find an "order of nature", whatever it could be. Linnaeus, for example, will see a "divine order". For Darwin and Haeckel, it will be the past genealogy of species. For Lamarck, their order of evolution.

However, some authors openly renounce to find such order of nature. Such are the authors belonging to the phenetic school, such as Sokal & Sneath.

Character #99: Explicitation of how the tree is constructed

Yes: 0;

No: 1;

Some authors explicit the way species are related to each other in a tree: sharing of characters, similarity rates, overall shape … Such do, for example, authors of "keys" like Klein or Gessner. They base their classifications on commonalities among species. It is also the case for authors like Darwin, Hennig or Sokal & Sneath.

In contrast, Romer, Haeckel or Teilhard de Chardin do not explicit how their tree is elaborated.

Character #100: Justification of the classificatory system

No: 0;

Yes: 1;

Some authors will justify the criteria by which they classify their species. If the previous character (#99) sought to identify which authors detailed the elaboration of their classification, this one asks why a particular classification criterion is preferred over another. Teilhard de Chardin, Darwin, Sokal & Sneath, Augier or Nelson justify their classificatory system over others. They made their choice explicit.

However, trees synthetizing knowledge, such as Timetree of life, simple visualization of trees like OneZoom or simple classification structures, such as Delacour & Mayr's or Lankester's trees, do not justify this classificatory system.

Character #101: Justification of using a tree as a representation

No: 0;

Yes: 1;

Classifying is a thing. Using tree to illustrate this classification is another thing. Some authors justify this use of a tree for classifying beings. Augier or Darwin are some of them.

However, this is not the case for other authors like Haeckel, Teilhard de Chardin or Barbançois.

Character #102: The tree is a simplification of reality.

No: 0;

Yes: 1;

The tree drawn by some authors is assumed as being only a mere illustration, a simplification of reality. It does not aim at representing the phenomenon described in the book, but only at illustrating, giving a simple approach.

This is for example the case of Teilhard de Chardin's or of Lamarck's trees.

However, this is not the case of Darwin's tree, which seeks to accurately represent the phenomenon described and the principle of genealogical classification. This is neither, for another reason, the case of authors using the phenetic method as Sokal & Sneath. Indeed, for them, the tree cannot be a simplification of reality as it does not represent it.

Character #103: Is there any classificatory aim?

Yes: 0;

No: 1;

Some trees do not intend to propose any classification of life.

This is particularly the case of Teilhard trees in his book *Le Phénomène Humain*. Here, trees are used only to illustrate the ideas of the author on teleology.

Character #104: Is classification elaborated before the tree?

Yes: 0;

No: 1;

There are two possible ways to classify beings: upstream or downstream of the tree.

In the works of authors like Romer or Haeckel, but also in the case of the "keys" elaborated by Gesner or Klein, the classification is established before the tree, which merely illustrates it.

However, for other authors like Hennig or Sokal & Sneath, the tree is made first, and then gives the classification.

Theme: Tree and evolution

Character #105: Is there teleology, any direction of evolution?

Yes: 0;

Implicitly: 1;

No: 2;

Teleology is a direction to which evolution is guided.

This can be explicitly assumed by some authors, like Teilhard de Chardin.

Other ones, like Darwin, Romer or Hennig, do not see any teleology in evolution.

Finally, other authors, like Haeckel, have an implicit teleology emerging from their writings. Indeed, because evolution goes from the simplest to the most complex, evolution of bloodlines is guided toward maximum complexity, and not due to mere chance (for example the “genealogical tree of mankind” in *Anthropogenie*).

Character #106: Are there any evolutionary leaps, or saltations?

Yes: 0;

No: 1;

An "evolutionary leap" is a large and sudden change in the shape or capacity (organs, nervous system, etc.) of a species. It is an evolutionary jump.

Many authors see the existence of such evolutionary leaps: Romer, Haeckel or Buffon for example.

For other ones, as Lamarck, Darwin or Hennig, the change is progressive.

Character #107: Do evolutionary rates vary among bloodlines?

Yes: 0;

No: 1;

The evolutionary rates of species may vary. Some authors consider that those rates differ across lineages. Thus, some bloodlines can evolve faster than other ones, due to morphological constraints or place in the food chain. Some of them could even remain morphologically the same across generations: it is what some authors call a "living fossil".

Many of our authors, as Haeckel, Romer or Gregory, consider such variations of rates of changes among bloodlines.

Character #108: Do evolutionary rates vary over time?

No: 0;

Yes: 1;

Evolutionary rates may also differ, for a same bloodline, over time. Environmental conditions can be responsible for these variations. Then, they do not depend on the “abilities” of the bloodline itself, but a same bloodline may experience acceleration or deceleration of its evolutionary rate over times.

Darwin, Hennig or Delacour & Mayr see variation of evolutionary rates over times.

Darwin, Hennig or Delacour & Mayr see different rates of evolution along time.

Character #109: Does complexity grow?

Yes: 0;

No: 1;

Some authors see an increasing complexity in the evolution of beings.

This is not necessarily related to saltation or evolutionary jumps. Lamarck sees increasing complexity through a gradual progress. Other authors, like Romer or Haeckel, consider increasing complexity through saltation. Several saltationist authors do not see any increasing complexity in evolution.

Character #110: Are there any spontaneous generations?

Yes: 0;

No: 1;

Spontaneous generation is the appearance of living forms where there were none before. Beings appeared by spontaneous generation have therefore not been generated by other beings. Lamarck or Haeckel, in his *Natürliche Schöpfungsgeschichte*, consider the existence of such spontaneous generation.

Character No. 111: Can a same form be reached by different bloodlines?

Yes: 0;

No: 1;

According to some authors, a same form can be independently reached by different bloodlines. The concept of “form” is not here a mere “convergence”, but more a *bauplan*, a global organisation level.

For example, it is the case for Romer, Haeckel or Teilhard de Chardin.

Character No. 112: Does the tree show a gradation in terms of value among beings?

Yes: 0;

No: 1;

A gradation in terms of “value” implies both an increasing complexity and an improvement of the lineage, an increase in its perfection. Species are then classified according to their value.

For instante, this is the case for Romer or Haeckel’s trees.

This character is not redundant with characters #1 and #65 because, in some trees, including Trémaux' one, where there is an increasing complexity, but the gradation of value is not shown by the tree.

Axis 4: Development of the tree

Theme: relationships shown by the tree

Character No. 113: The expressed relationships are genealogical.

No: 0;

Yes: 1;

Some trees aim at presenting the genealogy of classified entities. This is the case of Darwin's, Lamarck's or Haeckel's trees. On the opposite, Gessner's “keys” or Sokal & Sneath's trees do not express such genealogies.

Character No. 114: The relations shown are the result of a calculation

No: 0;

Yes: 1;

Some authors use mathematical tools to establish relationships among objects of their trees. This is for example the case of Delacour & Mayr's tree, but also of Garrod's, Tillyard's, Sokal & Sneath's or Hennig's ones. The method used is not necessarily performed by an algorithm: for example, Garrod’s method may sound like parsimony, but it is not really one, mathematically speaking.

Character No. 115: The relations expressed are environmental.

No: 0;

Yes: 1;

Some trees express the sharing of a same environment. This may be biogeography, as in Buffon's tree, or a physical environment (water, land, etc.), as in Lamarck’s, Dollo’s or Lister’s trees.

Character No. 116: Among characters considered is the place in the food chain

No: 0;

Yes: 1;

Some authors also consider the place of species in the food chain to elaborate their classification. These authors are very few. Among them, Hitchcock and Gaudry.

Character No. 117: Among characters considered is intelligence, degree of nerve irrigation etc.

No: 0;

Yes: 1;

A criterion considered by several authors to elaborate their classifications is the “degree of intelligence”. This is particularly the case for Teilhard de Chardin, Haeckel or Barbançois.

Theme: classified objects

Character No. 118: Classified objects are species

No: 0;

Yes: 1;

Some authors classify species in their trees: species are at the tip of the branches. A "species" refer to a group of interbreeding individuals, or is declared as such by the author.

Gaudry, Buffon or Duchesne classify species in their trees.

Character No. 119: Classified objects are groups of higher rank than that of the species

Yes: 0;

No: 1;

Several authors classify in their trees groups of higher rank than the species rank. High-ranked groups are at the tip of the branches: families, classes, kingdoms, etc. These groups (“mammals”, “reptiles” are taken as real.

This is for example the case for Romer’s, Haeckel’s or Lamarck’s trees.

Character 120: Does the rank of the classified object matter?

No: 0;

Yes: 1;

Some authors indistinctly classify species, individuals or groups of higher rank, without further epistemological reflexion about the status of these ranks (which is not the priority). This is for example the case for Romer's or Lamarck's trees.

Conversely, Gaudry openly classifies species in his tree. This rank matters and corresponds to his vision of evolution and genealogical classification. This is also the case of Hennig, Sokal & Sneath or Nelson.

Character No. 121: The tree worths for all life

Yes: 0;

No: 1;

Some trees aim to classify or categorize all living forms. This is for example the case for Darwin's or Trémaux' trees. Other trees classify only a part of life: Romer's, Gaudry's or Lamarck's trees, for example, only worth for animals.

Character No. 122: The method used to develop the tree worths for all life: it is a general one.

Yes: 0;

No: 1;

Some authors consider that any method of classification should be adapted to the group under scrutiny, and should be reconsidered if a different group is studied.

This is for example the case of Lamarck's tree, which clearly distinguishes methods for plants and methods for animals.

Theme: aim of the tree

Character No. 123: Is the tree epistemological?

Yes: 0;

No: 1;

A tree is called "epistemological" when it offers a classification of real beings, when it exhibits relationships among concrete living things, given a classificatory theory. It is the outcome of a real research investigation.

Haeckel's, Garrod's, Lankester's, Gaudry's or Lamarck's trees are epistemological ones.

Character No. 124: Is the tree theoretical?

Yes: 0;

No: 1;

A tree is called "theoretical" when it represents a theory of relationships and/or classification rather than relationships among concrete organisms.

Darwin's or Hennig's trees are examples of strictly theoretical trees. Names at the tip at the branches are letters. Lamarck's one is also a theoretical tree: it is both theoretical and epistemological, in that it offers both a model of classification of animals and an effective classification of them.

Character 125: Does the tree have a practical purpose?

No: 0;

Yes: 1;

The tree has a “practical” purpose when it aims at helping the reader in a specific task, such as identifying a specimen. Thus, determination keys, Klein's or Gessner's trees have such a practical purpose.

Character 126: Does the tree have a didactic purpose?

No: 0;

Yes: 1;

A tree has a "didactic" purpose when it aims at synthesizing knowledge. Haeckel's, Teilhard de Chardin's or Gregory's trees have such a didactic purpose. However, Darwin's or Lamarck's trees, published as strict scientific works, do not have such a purpose.

Character 127: Does the tree have an aesthetic purpose?

No: 0;

Yes: 1;

A tree has an "aesthetic" purpose when its design is openly intended to be aesthetic, pleasant to read. Such are the trees drawn as "real" trees (Haeckel's, Gruenberg's or Cuénot's ones), but also the trees drawn with the *OneZoom* software.

A tree can have its leaves, its trunk or its branches designed aesthetically without having on itself an aesthetic vocation. This is for example the case of the leaves of the tree made by Gregory at the p.992 of his *Evolution Emerging*, or the trunk of Trémaux's tree.

Theme: What does the tree express?

Character No. 128: The tree topology is reconstructing the tree of life

No: 0;

Yes: 1;

Topology is here understood in its sense used in Systematics: the internal organization of branches, nodes and leaves. Some authors reconstruct the topology of their trees and then consider that this topology reflects a historical phenomenon. The theoretical « tree of life » is reconstructed by the topology. Haeckel or Hennig are reconstructing this way the « tree of life » as an underlying historical phenomenon. Other authors like Sokal & Sneath consider that there is no « tree of life » to be reconstructed by their topology.

Character 129: The tree topology is created as a result of an investigation

Yes: 0;

No: 1;

Some authors can draw trees in which nodes are considered as the classifier's creation. Sometimes these nodes can show neither ancestors nor genealogy. This is for example the case of phenetic trees made by Sokal & Sneath or Fitch. Other authors will be aware that the nodes of their trees are a creation resulting from their investigation, study and calculations, but will later interpret this topology as a genealogical hypothesis probably reflecting the « tree of life ». These authors are, for example, Hennig and Nelson. Indeed, onto a cladogram, the node is a reconstruction of character states, which can be given afterwards the status of an ancestor. The tree drawn is the result of human investigation.

The topology of the tree can be or reconstituted or created, or both:

Both: the tree is created by the investigator, reconstructing the tree of life: Hennig, Nelson;

Created, not reconstituted (the tree is created by the investigator, not reconstructing the tree of life): Sokal & Sneath, Fitch;

Not created, reconstituted (the tree is the direct reconstruction of the tree of life): Haeckel.

If the topology is considered as created as the result of an investigation, but probably reconstructing the « tree of life », we are in the cladistic method. If it is only created as the result of an investigation, without reconstructing a « tree of life », we are for example in Sokal & Sneath’s way of thinking. If the topology of the « tree of life » is directly accessible, then the reconstruction is reified. This is particularly the case of Haeckel's trees.

Character No. 130: The tree must follow genealogy

No: 0;

Yes: 1;

Trees can follow the genealogy of the species. This is for example the case for Lamarck's, Darwin's and Haeckel's trees. On the opposite, Klein's, Linnaeus', Agassiz's or Augier's trees are not genealogical ones : their authors do not think in terms of evolution. Some trees do not to follow genealogy while their authors accept the idea of ​​evolution. This is the case for trees developed following a phenetic method, suth as those of Sokal & Sneath.

Character 131: The tree must follow overall similarity

Yes: 0;

No: 1;

Trees can sometimes be non-genealogical ones, like those of Wallace, Augier or Sokal & Sneath. Others may be genealogical but with no idea of overall similarity, such as Darwin's or Hennig's ones. Some other trees follow both genealogy and overall similarity, such as Haeckel's or Romer's ones. Finally, some trees will follow neither genealogy, nor overall physical similarity: Gessner's, Klein's or Brisson's “keys”. Combinations of considerations about genealogy and overall similarity among authors can be listed as follows:

Trees reflect overall similarity and genealogy: Haeckel, Romer;

Trees reflect genealogy but not overall similarity: Darwin, Hennig;

Trees reflect overall similarity but not genealogy: Augier, Sokal & Sneath;

Trees reflect neither genealogy nor overall similarity: Klein, Brisson.

Theme: Trees and time

Character 132: How is time considered in the tree?

No time: 0;

The tree is temporal, but not genealogical: 1;

The tree is genealogical: 2;

Some trees do not take time into consideration. This can be explained by an absence of consideration of evolution as a phenomenon: so are, for example, Augier's or Gessner's trees. But this phenomenon can also be found in clearly evolutionist authors' trees: phenetician ones, like Sokal & Sneath. Here, the tree does not bear any notion of time because its authors are aware that the genealogy of the species can not be found with their method. Other trees are only temporal (coded 1): Agassiz's one, for example. Those trees are based on the fossil record and see a succession of species over time, but without any idea of evolution. Agassiz indeed sees a succession of creations and extinctions of species due to supernatural causes. Finally, most of our trees take into consideration the genealogy of species: Darwin's, Lamarck's or Haeckel's ones, for example.

Character 133: Can fossils be classified?

No: 0;

Yes: 1;

Some authors cannot classify fossils on their trees. This may be because they do not believe in the possibility of disappearance of species, such as, for example, Lamarck. This can also be explained by the fact that the method used is not compatible with fossil data. This is notably the case of trees derived from molecular data only.

Theme: Characters and their states

Character 134: States of characters used

Arbitrary choice: 0;

All of them: 1;

Only derived ones: 2;

Some authors will use discrete characters in order to justify their groupings. Many authors will base them on several arbitrary character states. This is particularly what do authors like Gessner or Klein. Others will instead use all character states to establish their classifications (being either primitive or derived). This justifies some groups such as "reptiles" (an amnios but neither hair nor feathers) or "fishes" (vertebrae, but no four legs). This is for example what Haeckel and Romer do. Finally, some authors, such as Hennig will classify beings only on the basis of derived character states: what is original and unique to several species will justify their grouping. This character is not redundant with the character #93: indeed, an “incomplete” group set on a tree can be based on the sharing of derived characters. Such are for example, the traditional groups of "reptiles" (the amnios appears at the root of reptiles and it is a derived feature in the context of vertebrates) or "fishes" (the cranium appears at the root of fishes and it is a derived feature in the context of deuterostomians).

Character 135: Characters used

Arbitrary choice: 0;

Several sources: 1;

Only one given kind of characters: 2;

A group is justified on the basis of sharings. These can be chosen arbitrarily, and their kind (either the shape of seeds, number of petals, etc.) possibly change depending on the group studied: such are, for example Gessner's, Klein's and Linnaeus' ones. Other authors will classify species according to multiple and heterogeneous information sources: living environment, alimentation, organs. Such are, for example, Romer's and Haeckel's classifications. Other authors will finally take into account only some character type. In cladistics, for example, species are only classified on the basis of what they have, and not depending on where they live or what they do. Such authors are, for example, include Sokal & Sneath or Hennig.

Character 136: Use of an outgroup?

No: 0;

Yes: 1;

An outgroup is a set of entities thought to be close to the studied group, but outside it, not belonging to it. This outgroup is used to determine what is proper to the group under study, or proper to a part of it (then called “derived” or « apomorphic » character states) and what is not (then called « primitive » or « plesiomorphic » character states). In other words, it is made to identify what is new among the set of species to classify, and what is not. Although it is commonly used today (with authors like Nelson or Sober), the use of an outgroup is not so recent: Garrod also used it for his classification of parrots. This character is not redundant with the character #134 because of the classificatory system proposed by Buffon or Darwin. Indeed, if it seems impossible to identify derived states of characters without using ant outgroup, this has not always been the case. Some authors, like Buffon, or Darwin, do not use an outgroup to do so.

Theme: tree and algorithmics

Character 137: Criterion for the choice of the tree: maximization of sharings

No: 0;

Yes: 1;

The tree that is chosen maximizes consistency of sharings across all the charachers of the matrix. The maximization of sharings among objects of a classification aims at minimizing the assumptions of independent appearance of characters–that is to say, finding the shortest possible tree. This is now formalized as the "parsimony principle" and the shortest tree of all is found with the help of computers and algorithms: this is for example what do authors such as Nelson & Platnick or Sober. It is noticeable that several authors have, long before the development of the computers, tried to find by themselves the shortest tree of all. Tillyard, Lankester and Garrod are those authors. However, this is not the case for trees developed using phenetic methods: such trees do not take into consideration the sharing of explicit characters.

Character 138: Criteria for the choice of the tree based on overall similarity

No: 0;

Yes: 1;

Algorithms may aim at selecting the tree that maximizes or minimizes a metric dealing with numerized overall similarity (for instance in phenetics, the choice of a tree that best reflects the pairwide distances of the initial matrix using a least-square method). As a result, the most globally similar are the species classified, the closer they are in the tree. Saitou or Sokal & Sneath use such methods. Note that this character is not duplicating character #131. Overall similarity can be considered for the elaboration of the tree, but without algorithms nor explicit criterion for retaining one of the possible trees (like in Augier, Romer or Haeckel, for instance; especially in Haeckel there is no explicit choice among possible trees).

Character 139: Criteria for the choice of the tree: probabilistic approaches

Yes: 0;

No: 1;

The optimization of sharings according to the likelihood method consists in selecting, among all trees, the tree that maximizes the likelihood of the data at hand according to the tree and the model. The model is about probabilities of molecular evolution. Another probabilistic approach is calculating the posterior probability of a branch according to the data and the evolutionary model at hand. This is called the Bayesian approach. Hedges and Kumar use, as an example, probabilistic approaches to develop their Timetree of life. Note that this method retains characters and can infer a node reconstruction, unlike, for example distance methods.

Character 140: Use of algorithms

No: 0;

Yes: 1;

An algorithm is a sequence of operations. They may be used to construct trees. Sober, Sokal & Sneath or Nelson especially use algorithms for their trees.

Character 141: Characters formalization

No: 0;

Yes: 1;

Formalizing characters means precisely describing their states for each studied object and coding those states into a character matrix. A character matrix and an alignment of DNA sequences are two forms of formalization of characters, but other ones exist: tables, lists, etc. Garrod, Sokal & Sneath or Hennig formalize the characters of the objects they classify. Haeckel, however, does not do this.

## Supporting information

S1 File Matrix-copy-raw-data. Matrix in Word format.
(DOCX)

S2 File Matrice-Fisler-Lecointre.nex. Matrix in Nexus format.
(NEX)

S3 File Matrix of 235 trees and 141 characters. “?” means “unknown” ; “-” means “irrelevant”. See Annex I for description of trees that are coded and Annex II for description of each of the 141 characters.
(PNG)

## References

[1] Currie TE, Greenhill SJ, Mace R. Is horizontal transmission really a problem for phylogenetic comparative methods? A simulation study using continuous cultural traits. Phil trans Roy Soc Lond. B (Biol. Sci.). 2010; 365: 3903–3912. 21041214 10.1098/rstb.2010.0014 PMC2981909

[2] Gray RD, Bryant D, Greenhill SJ. On the shape and fabric of human history. Phil trans Roy Soc Lond. B (Biol. Sci.). 2010; 365: 3923–3933. 21041216 10.1098/rstb.2010.0162 PMC2981918

[3] Heggarty P, Maguire W, McMahon A. Splits or waves? Trees or webs? How divergence measures and network analysis can unravel language histories. Phil trans Roy Soc Lond. B (Biol. Sci.). 2010; 365: 3829–3843. 21041208 10.1098/rstb.2010.0099 PMC2981917

[4] Nunn CL, Arnold C, Matthews L, Borgerhoff Mulder M. Simulating trait evolution for cross-cultural comparison. Phil trans Roy Soc Lond. B (Biol. Sci.). 2010; 365: 3807–3819. 21041206 10.1098/rstb.2010.0009 PMC2981906

[5] Brown S, Savage PE, Ko AMS, Stoneking M, Ko YC, Loo JH, Trejaut JA. Correlations in the population structure of music, genes and langage. Proc Roy Soc Lond B. 2014; 281: 2013–2072. 10.1098/rspb.2013.2072 PMC3843827 24225453

[6] Charbonnat P, Ben Hamed M, Lecointre G, editors. Apparenter la pensée ? Vers une phylogénie des concepts savants. Paris: Éditions Matériologiques; 2014.

[7] Fisler M, Lecointre G. Categorizing Ideas about Trees: A Tree of Trees. PLoS ONE, 2013; 8(8): e68814 10.1371/journal.pone.0068814 23950877 PMC3737276

[8] Tassy PE. L'arbre à remonter le temps. Paris: Christian Bourgois Éditeur; 1991.

[9] Barsanti G. La scala, la mappa, l'albero Immagini e classificazioni della natura fra sei e ottocento. Firenze: Sansoni; 1992.

[10] Ragan MA. Trees and Networks before and after Darwin. Biology Direct. 2009; 4(43), Available from: 10.1186/1745-6150-4-43 PMC2793248 19917100

[11] Pietsch T. Trees of Life A visual History of Evolution. Baltimore: John Hopkins; 2012.

[12] Fisler M, Crémière C, Lecointre G. Qu’est-ce qu’un arbre des idées? Explicitation des notions d’arbre et de phylogénie et histoire des représentations de l’arbre In: Charbonnat P, Ben Hamed M, Lecointre G, editors. *Apparenter la pensée*. Paris: Éditions Matériologiques; 2014 pp. 103–186.

[13] Brower A, De Pinna M. About nothing. Cladistics 2014; 30: 330–336. 10.1111/cla.12050 34788975

[14] Gontier N. Depicting the Tree of life: the Philosophical and historical Roots of Evolutionary Tree Diagrams. Evo Edu Outreach. 2011; 4: 515–538.

[15] Diderot D, D'Alembert J. Encyclopédie ou Dictionnaire raisonné des sciences, des arts et des métiers. Paris: Briasson; 1751.

[16] Schleicher A. Die ersten Spaltungen des indogermanischen Urvolkes. Allgemeine Monatsschrift für Wissenschaft und Literatur 1853; 3: 786–787.

[17] Holden CJ. Bantu language trees reflect the spread of farming across sub-Saharan Africa: a maximum-parsimony analysis. Proc. Roy. Soc. Lond. B. 2001; 269: 793–799. 10.1098/rspb.2002.1955 PMC1690959 11958710

[18] Gray RD, Atkinson QD. Language-tree divergence times support the Anatolian theory of Indo-European origin. Nature. 2003; 426: 435–439. 10.1038/nature02029 14647380

[19] Hurles ME, Matisoo-Smith E, Gray RD, Penny D. Untangling Polynesian origins: the edge of the knowable. Trends Ecol Evol. 2003; 18(10): 531–540.

[20] Nakhleh L, Warnow T, Ringe D, Evans SN. A comparison of phylogenetic reconstruction methods on an Indo-European Dataset. Trans Phil Soc. 2005; 103(2): 171–192.

[21] Swofford DL (2002) PAUP*. Phylogenetic Analysis Using Parsimony (*and Other Methods). Version 4. Sunderland, Massachusetts: Sinauer Associates; 2002.

[22] Kluge AG, Farris JS. Quantitative phyletics and the evolution of anurans. Syst Zool. 1969; 18(1): 1–32.

[23] Archie JW. A randomization test for phylogenetic information in systematic data. Syst Zool. 1989; 38: 239–252.

[24] Darlu P, Tassy PE. Reconstruction phylogénétique. Concepts et méthodes. Paris: Masson; 1993.

[25] Darlu P, Tassy PE. Reconstruction phylogénétique. Concepts et méthodes. 2d ed Paris: Masson; 2019.

[26] Archie JW. Homoplasy excess statistics and retention indices: a reply to Farris. Syst Zool. 1990; 39: 169–174.

[27] Huson DH, Bryant D. Application of Phylogenetic Networks in Evolutionary Studies. Mol Biol Evol. 2006; 23(2): 254–267. 10.1093/molbev/msj030 16221896

[28] Holland BR, Huber KT, Dress A, Moulton V. D-Plots: a tool for analyzing phylogenetic distance data. Mol Biol Evol. 2002; 19: 2051–2059. 10.1093/oxfordjournals.molbev.a004030 12446797

[29] Bremer K. Branch support and tree stability. Cladistics 1994; 10: 295–304.

[30] Christoffersen ML. Cladistic taxonomy, phylogenetic systematics, and evolutionary ranking. Syst Biol. 1995; 44(3): 440–454.

[31] Grimoult C. Histoire de l'évolutionnisme contemporain en France (1945–1995). Genève: Droz; 2000.

[32] Grimoult C. L'évolution biologique en France: une révolution scientifique, politique et culturelle. Genève: Droz; 2001.

[33] Grimoult C. Évolutionnisme et fixisme en France: histoire d'un combat (1800–1882). Paris: CNRS Éditions; 1998.

[34] Buican D. L'évolution et les théories évolutionnistes. Paris: Masson; 1997.

[35] Gaudant M, Gaudant J. Les théories classiques de l'évolution. Paris: Dunod; 1971.

[36] Roger J. Buffon: un philosophe au Jardin du Roi. Paris: Fayard; 1989.

[37] Grimoult C. Histoire de l'histoire des sciences Historiographie de l'évolutionnisme dans le monde francophone. Genève: Droz; 2003.

[38] De Quatrefages A. Lamarck In: De Quatrefages A, editor. Darwin et ses précurseurs français. Étude sur le transformisme. Paris: G. Baillière; 1870 pp. 42–59.

[39] Tassy PE. Teilhard de Chardin, l'arbre phylogénétique et l'orthogenèse In: Athané F, Guinet E, Silberstein M, editors. Émergence et réductions, Matière première, Tome 2. Paris: Syllepse; 2007 pp. 289–309.

[40] De Bonis L. Les carnassiers des phosphorites du Quercy: évolution et phylogénie d'après P. Teilhard de Chardin. Annales de paléontologie 2006; 92: 205–215.

[41] Wiley EO. Phylogenetics: Theory and Practice of Phylogenetic Systematics. New York: Wiley; 1981.

[42] Dupuis C. Willi Hennig's impact on taxonomic thought. Ann. Rev. Ecol. Syst. 1984; 15: 1–24.

[43] Mayr E. La systémique évolutionniste et les quatre étapes du processus de classification In: Tassy PE, editor. L'Ordre et la diversité du vivant. Quel statut scientifique pour les classifications biologiques? Paris: Fayard; 1986 pp. 143–160.

[44] Carpenter JM. Cladistics of cladists. Cladistics 1987; 3: 363–375. 10.1111/j.1096-0031.1987.tb00899.x 34949058

[45] Hull DL. Science as a Process: An Evolutionary Account of the Social and Conceptual Development of Science. Chicago: University of Chicago Press; 1988.

[46] Ebach MC, Morrone JJ, Williams DM. A new cladistics of cladists. Biology and Philosophy. 2008; 23(1): 153–156.

[47] Dupuis C. Darwin et les taxonomies d'aujourd'hui In Tassy PE, editor. L'Ordre et la diversité du vivant. Quel statut scientifique pour les classifications biologiques? Paris: Fayard; 1986 pp. 215–240.

[48] Schuh RT. Biological Systematics: Principles and Applications. Ithaca: Cornell University Press; 2000.

[49] Dupuis C. Permanence et actualité de la systématique : la “systématique phylogénétique” de W. Hennig. Historique, discussion, choix de références. Cahier des naturalistes, Bull. N. P. n. s. 1978; 341: 1–69.

[50] Dupuis C. Regards épistémologiques sur la taxinomie cladiste. Adresse à la onzième session de la Willi Hennig Society Paris, 1992. Cahier des naturalistes, Bull. N. P. n. s. 1992; 482: 29–56.

[51] Browne J. Strickland on Natural Systems In: Brooks JL editor. Just Before the Origin: Alfred Russel Wallace's Theory of Evolution. New York: Columbia University Press; 1985 pp. 96–99.

[52] O'Hara RJ. Strickland and Wallace and the systematic argument for evolution. Amer Zool. 1987; 27(4): 107A.

[53] O'Hara RJ. Representations of the Natural System in the Nineteenth century. Biology and Philosophy 1991; 6(2): 255–274.

[54] Collard M, Shennan SJ, Tehrani JJ. Branching, blending, and the evolution of cultural similarities and differences among human populations. Evol Hum Behav. 2006; 27: 169–184.

[55] Le Bomin S, Lecointre G, Heyer H. The Evolution of Musical Diversity: The Key Role of Vertical Transmission. PLoS ONE. 2016; 11(3): e0151570 10.1371/journal.pone.0151570 27027305 PMC4814106

