# Supplementary material for: The treeness of the tree of historical trees of life
Source: PLoS One. 2020 Jan 15;15(1):e0226567. doi: 10.1371/journal.pone.0226567 (PMC6961905; doi:10.1371/journal.pone.0226567)
Supplement: S1 File — Matrix in Word format. (DOCX) [file pone.0226567.s001.docx]

1-Outgroup 0 0 0 0 0 0 0 0 0 0 0 0 0 0 0 0 0 0 0 0 0 0 0 0 0 0 0 0 0 0 0 0 0 0 0 0 0 0 0 0 0 0 0 0 0 0 0 0 0 0 0 0 0 0 0 0 0 0 0 0 0 0 0 0 0 0 0 0 0 0 0 0 0 - 0 0 0 0 0 0 0 0 0 0 0 0 0 0 0 0 0 0 0 0 0 0 0 0 0 0 0 0 0 0 0 0 0 0 0 0 0 0 0 0 0 0 0 0 0 0 0 0 0 0 0 0 0 0 0 0 0 0 0 0 0 0 0 - - 0 0

2-Gessner. Historiae animalium 0 0 0 0 1 0 0 - - - 0 - - - - - - - - - - 1 0 0 - - 0 0 0 0 0 1 1 0 1 0 0 1 - 0 0 1 0 0 0 0 1 - - - - - 1 2 1 0 1 1 0 0 0 0 0 0 0 0 0 0 0 0 0 - - - - - - - 0 1 0 1 0 0 1 0 1 0 1 0 0 1 1 2 0 1 0 ? 0 0 0 ? 0 0 - - - - - - - 1 0 0 0 0 0 0 1 0 1 0 0 1 1 0 0 1 0 0 1 0 0 0 0 0 0 - - 0 0

3-De lobel. Plantarum seu stirpium historia, Tree #1 0 0 0 0 1 0 0 - - - 0 - - - - - - - - - - 1 0 0 - - 0 0 0 0 0 1 1 0 1 0 0 1 - 0 0 1 0 0 0 0 1 - - - - - 1 2 1 0 1 1 0 0 0 0 0 0 0 0 0 0 0 0 0 - - - - - - - 0 1 0 1 0 0 1 0 0 0 1 0 0 1 1 2 0 1 ? ? 0 0 0 ? 0 0 - - - - - - - 1 0 0 0 0 0 1 1 0 1 0 0 1 1 0 0 1 0 0 1 0 0 0 0 0 0 - - 0 0

4-De lobel. Plantarum seu stirpium historia, Tree #2 0 0 0 0 1 0 0 - - - 0 - - - - - - - - - - 1 0 0 - - 0 0 0 0 0 1 1 0 1 0 0 1 - 0 0 1 0 0 0 0 1 - - - - - 1 2 1 0 1 1 0 0 0 0 0 0 0 0 0 0 0 0 0 - - - - - - - 0 1 0 1 0 0 1 0 0 0 1 0 0 1 1 2 0 1 ? ? 0 0 0 ? 0 0 - - - - - - - 1 0 0 0 0 0 1 1 0 1 0 0 1 1 0 0 1 0 0 1 0 0 0 0 0 0 - - 0 0

5-Zaluziansky. Methodi herbariae libri tres, Tree #1 0 2 0 0 1 0 0 - - - 0 - - - - - - - - - - 1 0 0 - - 0 0 0 0 0 1 1 0 1 0 0 1 - 0 0 0 0 0 0 0 1 - - - - - 1 2 1 0 1 1 0 0 0 0 0 0 0 0 0 0 0 0 0 - - - - - - - 0 1 0 1 0 0 1 0 1 0 1 0 0 1 1 2 0 1 0 ? 0 0 0 ? 0 0 - - - - - - - 1 0 0 0 0 0 1 0 0 1 0 0 1 1 0 0 1 0 0 1 0 0 0 0 0 0 - - 0 0

6-Zaluziansky. Methodi herbariae libri tres, Tree #2 0 2 0 0 1 0 0 - - - 0 - - - - - - - - - - 1 0 0 - - 0 0 0 0 0 1 1 0 1 0 0 1 - 0 0 0 0 0 0 0 1 - - - - - 1 2 1 0 1 1 0 0 0 0 0 0 0 0 0 0 0 0 0 - - - - - - - 0 1 0 1 0 0 1 0 1 0 1 0 0 1 1 2 0 1 0 ? 0 0 0 ? 0 0 - - - - - - - 1 0 0 0 0 0 1 0 0 1 0 0 1 1 0 0 1 0 0 1 0 0 0 0 0 0 - - 0 0

7-Zaluziansky. Methodi herbariae libri tres, Tree #3 0 2 0 0 1 0 0 - - - 0 - - - - - - - - - - 1 0 0 - - 0 0 0 0 0 1 1 0 1 0 0 1 - 0 0 0 0 0 0 0 1 - - - - - 1 2 1 0 1 1 0 0 0 0 0 0 0 0 0 0 0 0 0 - - - - - - - 0 1 0 1 0 0 1 0 1 0 1 0 0 1 1 2 0 1 0 ? 0 0 0 ? 0 0 - - - - - - - 1 0 0 0 0 0 1 0 0 1 0 0 1 1 0 0 1 0 0 1 0 0 0 0 0 0 - - 0 0

8-Morison. Plantarum umbelliferarum distributio nova, Tree #1 0 2 0 0 1 0 0 - - - 0 - - - - - - - - - - 1 0 0 - - 0 0 0 0 0 1 1 0 1 0 0 1 - 0 0 1 0 0 0 0 1 - - - - - 1 2 1 0 1 1 0 0 0 0 0 0 0 0 0 0 0 0 0 - - - - - - - 0 1 0 1 0 0 1 0 1 0 1 0 0 1 1 2 0 1 0 ? 0 0 0 ? 0 0 - - - - - - - 1 0 0 1 0 0 1 1 0 1 0 0 1 1 0 0 1 0 0 1 0 0 0 0 0 0 - - 0 0

9-Morison. Plantarum umbelliferarum distributio nova, Tree #2 0 2 0 0 1 0 0 - - - 0 - - - - - - - - - - 1 0 0 - - 0 0 0 0 0 1 1 0 1 0 0 1 - 0 0 1 0 0 0 0 1 - - - - - 1 2 1 0 1 1 0 0 0 0 0 0 0 0 0 0 0 0 0 - - - - - - - 0 1 0 1 0 0 1 0 1 0 1 0 0 1 1 2 0 1 0 ? 0 0 0 ? 0 0 - - - - - - - 1 0 0 1 0 0 1 1 0 1 0 0 1 1 0 0 1 0 0 1 0 0 0 0 0 0 - - 0 0

10-Morison. Plantarum umbelliferarum distributio nova, Tree # 0 2 0 0 1 0 1 - - - 0 - - - - - - - - - - 1 0 0 - - 0 0 0 0 0 1 1 0 1 0 0 1 - 0 0 1 0 0 0 0 1 - - - - - 1 2 1 0 1 1 0 0 0 0 0 0 0 0 0 0 0 0 0 - - - - - - - 0 1 0 1 0 0 1 0 1 0 1 0 0 1 1 2 0 1 0 ? 0 0 0 ? 0 0 - - - - - - - 1 0 0 1 0 0 1 1 0 1 0 0 1 1 0 0 1 0 0 1 0 0 0 0 0 0 - - 0 0

11-Lister. Historiae animalium angliae, Tree #1 0 2 0 0 0 0 0 - - - 0 - - - - - - - - - - 1 0 0 - - 0 0 0 0 0 1 1 0 1 0 0 1 - 0 0 1 0 0 0 0 1 - - - - - 1 2 1 0 1 1 0 0 0 0 0 0 0 0 0 0 0 0 0 - - - - - - - 0 1 0 1 0 0 1 0 1 0 1 0 0 1 1 2 0 1 0 ? 0 0 0 ? 0 0 - - - - - - - 1 0 0 0 0 0 1 1 0 1 0 0 1 1 0 0 1 0 0 1 0 0 0 0 0 0 - - 0 0

12-Lister. Historiae animalium angliae, Tree #2 0 2 0 0 0 0 0 - - - 0 - - - - - - - - - - 1 0 0 - - 0 0 0 0 0 1 1 0 1 0 0 1 - 0 0 1 0 0 0 0 1 - - - - - 1 2 1 1 1 1 0 0 0 0 0 0 0 0 0 0 0 0 0 - - - - - - - 0 1 0 1 0 0 1 0 1 0 1 0 0 1 1 2 0 1 0 ? 0 0 0 ? 0 0 - - - - - - - 1 0 0 1 0 0 1 1 0 1 0 0 1 1 0 0 1 0 0 1 0 0 0 0 0 0 - - 0 0

13-Lister. Historiae animalium angliae, Tree #3 0 2 0 0 0 0 0 - - - 0 - - - - - - - - - - 1 0 0 - - 0 0 0 0 0 1 1 0 1 0 0 1 - 0 0 1 0 0 0 0 1 - - - - - 1 2 1 0 1 1 0 0 0 0 0 0 0 0 0 0 0 0 0 - - - - - - - 0 1 0 1 0 0 1 0 1 0 1 0 0 1 1 2 0 1 0 ? 0 0 0 ? 0 0 - - - - - - - 1 0 0 1 0 0 1 1 0 1 0 0 1 1 0 0 1 0 0 1 0 0 0 0 0 0 - - 0 0

14-Willughby. De historia piscium libri quatuor 0 2 0 0 0 0 0 - - - 0 - - - - - - - - - - 1 0 0 - - 0 0 0 0 0 1 1 0 1 0 0 1 - 0 0 1 0 0 0 0 1 - - - - - 1 2 1 0 1 1 0 0 0 0 0 0 0 0 0 0 0 0 0 - - - - - - - 0 1 0 1 0 0 1 0 1 0 1 0 0 1 1 2 0 1 0 ? 0 0 0 ? 0 0 - - - - - - - 1 0 0 0 0 0 1 0 0 1 0 0 1 1 0 0 1 0 0 1 0 0 0 0 0 0 - - 0 0

15-Ray. Synopsis methodica animalium quadrupedum et serp 0 2 0 0 0 0 0 - - - 0 - - - - - - - - - - 1 0 0 - - 0 0 0 0 0 1 1 0 1 0 0 1 - 0 0 1 0 0 0 0 1 - - - - - 1 2 1 0 1 1 0 0 0 0 0 0 0 0 0 0 0 0 0 - - - - - 0 0 0 1 0 1 0 0 1 0 1 0 1 0 0 1 1 2 0 1 ? 0 0 0 0 ? 0 0 - - - - - - - 1 0 0 0 0 0 0 0 0 1 1 0 1 1 0 0 1 0 0 1 0 0 0 0 0 0 - - 0 0

16-Linnaeus. Systema naturae vol.1 0 2 0 0 1 0 0 - - - 0 - - - - - - - - - - 1 0 0 - - 0 0 0 0 0 1 1 0 1 0 0 1 - 0 0 1 0 0 0 0 1 - - - - - 1 2 1 0 1 1 0 0 0 0 0 0 0 0 0 0 0 0 0 - - - - - - - 0 1 0 1 0 0 1 0 1 0 1 0 0 1 1 2 0 1 0 0 0 0 0 0 0 0 - - - - - - - 1 0 0 0 0 0 0 0 0 1 0 0 1 0 0 0 1 0 0 1 0 0 0 0 0 0 - - 0 0

17-Linnaeus. Systema naturae vol.9 0 2 0 0 1 0 0 - - - 0 - - - - - - - - - - 1 0 0 - - 0 0 0 0 0 1 1 0 1 0 0 1 - 0 0 1 0 0 0 0 1 - - - - - 1 2 1 0 1 1 0 0 0 0 0 0 0 0 0 0 0 0 0 - - - - - - - 0 1 0 1 0 0 1 0 1 0 1 0 0 1 1 2 0 1 0 0 0 0 0 0 0 0 - - - - - - - 1 0 0 0 0 0 0 0 0 1 0 0 1 0 0 0 1 0 0 1 0 0 0 0 0 0 - - 0 0

18-Klein. Historiae piscium naturalis, Tree #1 0 2 0 0 1 0 0 - - - 0 - - - - - - - - - - 1 0 0 - - 0 0 0 0 0 1 1 0 1 0 0 1 - 0 0 1 0 0 0 0 1 - - - - - 1 2 1 0 1 1 0 0 0 0 0 0 0 0 0 0 0 0 0 - - - - - - - 0 1 0 1 0 0 1 0 1 0 1 0 0 1 1 2 0 1 0 0 0 0 0 0 0 0 - - - - - - - 1 0 0 0 0 0 0 0 0 1 ? 0 1 1 0 0 1 0 0 1 0 0 0 0 0 0 - - 0 0

19-Klein. Historiae piscium naturalis, Tree #2 0 2 0 0 1 0 0 - - - 0 - - - - - - - - - - 1 0 0 - - 0 0 0 0 0 1 1 0 1 0 0 1 - 0 0 1 0 0 0 0 1 - - - - - 1 2 1 0 1 1 0 0 0 0 0 0 0 0 0 0 0 0 0 - - - - - - - 0 1 0 1 0 0 1 0 1 0 1 0 0 1 1 2 0 1 0 0 0 0 0 0 0 0 - - - - - - - 1 0 0 0 0 0 0 0 0 1 ? 0 1 1 0 0 1 0 0 1 0 0 0 0 0 0 - - 0 0

20-Klein. Historiae piscium naturalis, Tree #3 0 2 0 0 1 0 0 - - - 0 - - - - - - - - - - 1 0 0 - - 0 0 0 0 0 1 1 0 1 0 0 1 - 0 0 1 0 0 0 0 1 - - - - - 1 2 1 0 1 1 0 0 0 0 0 0 0 0 0 0 0 0 0 - - - - - - - 0 1 0 1 0 0 1 0 1 0 1 0 0 1 1 2 0 1 0 0 0 0 0 0 0 0 - - - - - - - 1 0 0 0 0 0 0 0 0 1 ? 0 1 1 0 0 1 0 0 1 0 0 0 0 0 0 - - 0 0

21-Klein. Tentamen methodi ostracologicae, Tree #1 0 2 0 0 1 0 0 - - - 0 - - - - - - - - - - 1 0 0 - - 0 0 0 0 0 1 1 0 1 0 0 1 - 0 0 1 0 0 0 0 1 - - - - - 1 2 1 0 1 1 0 0 0 0 0 0 0 0 0 0 0 0 0 - - - - - - - 0 1 0 1 0 0 1 0 1 0 1 0 0 1 1 2 0 1 0 0 0 0 0 0 0 0 - - - - - - - 1 0 0 0 0 0 0 0 0 1 1 0 1 1 0 0 1 0 0 1 0 0 0 0 0 0 - - 0 0

22-Klein. Tentamen methodi ostracologicae, Tree #2 0 2 0 0 1 0 0 - - - 0 - - - - - - - - - - 1 0 0 - - 0 0 0 0 0 1 1 0 1 0 0 1 - 0 0 1 0 0 0 0 1 - - - - - 1 2 1 0 1 1 0 0 0 0 0 0 0 0 0 0 0 0 0 - - - - - - - 0 1 0 1 0 0 1 0 1 0 1 0 0 1 1 2 0 1 0 0 0 0 0 0 0 0 - - - - - - - 1 0 0 0 0 0 0 0 0 1 1 0 1 1 0 0 1 0 0 1 0 0 0 0 0 0 - - 0 0

23-Buffon. Histoire naturelle, generale et particuliere, tome 5 1 1 2 0 0 0 0 0 0 0 0 - - - - - - - - - - 0 0 1 0 - 0 0 1 0 0 1 0 0 0 0 1 1 0 0 0 0 0 0 0 0 0 - - - - - 0 0 0 1 1 0 1 1 0 2 0 1 0 0 1 0 0 0 1 1 0 1 1 2 2 0 1 0 1 1 0 1 0 0 1 1 1 1 0 0 0 1 0 1 ? 0 0 1 1 ? 0 0 2 0 1 0 1 1 1 0 1 0 1 0 0 1 1 0 1 1 0 1 0 0 1 0 1 1 1 2 1 2 1 0 0 - - 0 0

24-Brisson. Regnum animale in classes ix distributum 0 2 0 0 1 0 0 - - - 0 - - - - - - - - - - 1 0 0 - - 0 0 0 0 0 1 1 0 1 0 0 0 - 0 0 1 0 0 0 0 1 - - - - - 1 2 1 0 1 1 0 0 0 0 0 0 0 0 0 0 0 0 0 - - - - - 1 0 0 1 0 1 0 0 1 0 1 0 1 0 0 1 1 2 0 1 0 0 0 0 0 ? 0 0 - - - - - - - 1 0 0 0 0 0 1 0 0 1 1 0 1 1 0 0 1 0 0 1 0 0 0 0 0 0 - - 0 0

25-Geoffroy. Histoire abregee des insectes 0 2 0 0 0 0 1 - - - 0 - - - - - - - - - - 1 0 0 - - 0 0 0 0 0 1 1 0 1 0 0 1 - 0 0 1 0 0 0 0 1 - - - - - 1 2 1 0 1 1 0 0 0 0 0 0 0 0 0 0 0 0 0 - - - - - 2 0 0 1 0 1 0 0 1 0 1 0 1 0 1 1 1 2 0 1 0 ? 0 0 0 1 0 0 - - - - - - - 1 0 0 0 0 0 1 0 0 1 0 0 1 1 0 0 1 0 0 1 0 0 0 0 0 0 - - 0 0

26-Duchesne. Essai sur l-histoire naturelle des fraisiers 1 1 2 0 0 0 0 0 0 0 0 - - - - - - - - - - 0 0 1 0 - 0 0 1 0 0 1 0 0 0 0 1 1 0 0 0 0 0 0 0 0 0 - - - - - 0 0 0 0 1 0 1 1 0 2 0 0 0 0 0 0 0 0 0 1 0 1 1 2 2 0 1 0 1 1 0 ? 0 0 1 0 1 1 0 0 0 1 0 0 0 0 0 0 0 1 0 0 2 0 0 1 1 1 1 0 1 0 0 0 0 1 1 0 1 1 0 0 0 0 0 0 1 1 0 2 0 0 1 0 0 - - 0 0

27-Goüan. Histoire des poissons 0 2 0 0 0 0 0 - - - 0 - - - - - - - - - - 1 0 0 - - 0 0 0 0 0 1 1 0 1 0 0 1 - 0 0 0 0 0 0 0 0 - - - - - 1 2 1 0 1 1 0 0 0 0 0 0 0 0 0 0 0 0 0 - - - - - - - 0 0 0 1 0 0 1 0 1 0 1 0 0 1 1 2 0 1 0 ? 0 0 0 1 0 0 - - - - - - - 1 0 0 0 0 ? 0 0 0 1 1 0 1 1 0 0 1 0 0 1 0 0 0 0 0 0 - - 0 0

28-Esper. Die schmetterlinge in abbildungen nach der natur 0 2 0 0 0 0 0 - - - 0 - - - - - - - - - - 1 0 0 - - 0 0 0 0 0 1 1 0 1 0 0 1 - 0 0 1 0 0 0 0 1 - - - - - 1 2 1 0 1 1 0 0 0 0 0 0 0 0 0 0 0 0 ? - - - - - - - 0 0 0 1 0 0 1 0 1 0 1 0 0 1 1 2 0 1 0 0 0 0 0 ? 0 0 - - - - - - - 1 0 0 0 0 0 0 0 0 1 1 0 1 1 0 0 1 0 0 1 0 0 0 0 0 0 - - 0 0

29-De geer. Memoires pour servir a l-etude des insectes 0 2 0 0 1 0 0 - - - 0 - - - - - - - - - - 1 0 0 - - 0 0 0 0 0 1 1 0 1 0 0 1 - 0 0 1 0 0 0 0 1 - - - - - 1 2 1 0 1 1 0 0 0 0 0 0 0 0 0 0 0 0 ? - - - - - - - 0 0 0 1 0 0 1 0 1 0 1 0 0 1 1 2 0 1 0 ? 0 0 0 ? 0 0 - - - - - - - 1 0 0 0 0 0 1 0 0 1 ? 0 1 0 0 0 1 0 0 1 0 0 0 0 0 0 - - 0 0

30-Artedi. Genera piscium 0 2 0 0 0 0 0 - - - 0 - - - - - - - - - - 1 0 0 - - 0 0 0 0 0 1 1 0 1 0 0 1 - 0 0 1 0 0 0 0 1 - - - - - 1 2 1 0 1 1 0 0 0 0 0 0 0 0 0 0 0 0 0 - - - - - - - 0 1 0 1 0 0 1 0 0 0 1 0 0 1 1 2 0 1 0 0 0 0 0 ? 0 0 - - - - - - - 1 0 0 0 0 0 0 0 0 1 1 0 1 1 0 0 1 0 0 1 0 0 0 0 0 0 - - 0 0

31-Augier. Essai d-une nouvelle classification des vegetaux 2 2 0 0 0 0 0 0 0 0 2 0 1 0 1 0 0 0 0 0 1 0 0 1 0 - 1 2 0 0 0 1 0 0 0 0 0 0 0 1 0 0 0 1 1 1 0 0 0 0 0 0 0 1 0 0 0 0 1 1 0 0 0 0 1 0 0 0 0 0 ? - - - - - - - 0 0 0 0 0 0 0 0 0 0 1 0 0 1 0 1 0 1 0 0 0 1 1 0 0 1 - - - - - - - 0 0 0 0 0 0 1 0 0 1 0 0 0 0 0 1 1 0 0 0 0 0 1 1 0 0 - - 0 0

32-Lamarck. Philosophie zoologique 2 1 2 0 0 1 0 0 0 0 0 - - - - - - - - - - 0 0 1 0 0 0 0 1 0 1 1 0 0 0 0 1 0 0 0 0 0 0 0 0 0 0 - - - - - 0 0 0 1 0 0 1 1 0 1 0 1 0 0 0 0 0 0 0 1 0 1 1 0 1 0 1 0 0 1 0 1 0 0 0 0 0 0 0 0 0 1 0 0 1 0 1 1 1 1 1 0 2 1 1 0 0 0 0 0 1 0 1 0 0 0 0 0 1 1 0 0 0 0 0 0 1 1 0 2 1 1 1 0 0 - - 0 0

33-Lamarck. Histoire naturelle des animaux sans vertebres 2 0 1 0 0 0 1 0 0 0 0 - - - - - - - - - - - 0 1 0 0 0 0 1 0 1 1 1 0 0 0 1 0 0 0 0 0 0 0 0 0 0 - - - - - 0 0 0 0 0 0 1 1 0 1 0 0 0 0 0 0 0 0 0 1 0 1 1 0 1 0 0 0 0 1 0 ? 1 1 0 0 0 0 0 0 0 1 0 0 ? 0 0 0 1 1 0 0 2 1 1 0 0 0 1 0 1 0 0 0 1 0 0 0 1 0 0 0 0 0 0 1 1 1 0 2 ? 1 1 0 0 - - 0 0

34-Barbancois. Observations sur la filiation des animaux 2 0 2 0 0 0 0 0 0 0 0 - - - - - - - - - - 0 0 1 0 0 0 0 1 0 1 1 0 0 0 0 1 0 0 1 0 0 0 0 0 1 0 - - - - - 0 0 0 1 0 0 1 1 0 1 0 0 1 0 0 0 0 0 0 1 1 1 0 0 1 0 0 0 0 0 0 0 0 0 0 0 1 0 0 0 0 1 0 1 0 0 1 0 0 0 0 0 2 0 0 ? 0 0 0 0 1 0 0 0 1 0 0 0 1 0 0 1 0 0 0 1 1 1 0 2 0 1 1 0 0 - - 0 0

35-Barbancois. Observations pour servir a une classification 0 2 0 0 1 0 0 - - - 0 - - - - - - - - - - 1 0 0 - - 0 0 0 0 0 1 0 0 1 0 0 1 - 0 0 1 0 0 0 2 1 - - - - - 1 2 1 0 1 1 0 0 0 0 0 0 1 0 0 0 0 0 0 1 1 1 1 0 0 1 0 0 0 0 0 0 0 0 1 0 0 0 0 0 1 2 0 1 0 0 0 1 0 0 0 0 2 0 1 0 0 0 0 0 1 0 0 0 1 1 0 0 1 1 0 1 0 0 0 1 0 0 0 0 0 0 0 0 0 - - 0 0

36-Strickland. On the true method of discovering the natural s 0 2 0 0 0 0 0 0 0 0 0 - - - - - - - - - - 0 0 0 0 - 0 0 0 0 0 0 ? 0 0 0 0 1 0 1 0 0 0 0 0 1 0 - - - - - 1 0 0 0 1 0 0 1 1 0 0 0 0 0 0 0 0 0 0 - - - - - - - 0 0 0 1 0 0 1 0 0 1 1 0 0 0 0 1 0 0 0 0 1 1 1 0 0 0 - - - - - - - 1 0 0 0 0 0 1 1 0 0 0 1 0 0 0 0 1 0 0 0 0 0 1 1 0 0 - - 0 0

37-Agassiz. Recherches sur les poissons fossiles 2 0 0 0 0 0 0 0 1 0 1 - - - - - - - - - - 0 0 0 0 1 0 1 1 0 0 1 0 - - - - - - - 0 0 0 0 1 0 0 1 0 0 0 0 1 0 0 0 1 0 1 1 0 1 2 0 0 0 0 1 0 0 0 - 1 1 0 - - - 0 0 0 1 0 0 0 0 0 0 1 0 0 1 1 2 0 1 0 0 1 0 0 1 0 0 0 0 0 0 0 0 1 - 0 0 0 0 0 1 0 0 1 0 0 0 0 0 0 1 0 0 0 1 1 0 0 0 0 - - 0 0

38-Chambers. Vestiges of natural history of creation 2 2 0 0 0 0 0 1 1 0 0 0 0 0 0 0 1 0 1 1 1 0 0 1 - - 0 0 1 0 0 1 0 1 0 0 1 0 0 1 0 0 1 0 0 1 0 - - - - - 0 0 0 0 0 0 1 1 0 2 0 0 0 0 0 0 0 0 0 1 1 0 1 0 1 0 0 0 0 0 0 0 0 0 0 ? 0 0 0 0 0 1 0 0 0 0 1 0 0 0 0 0 1 1 0 1 0 1 0 0 1 0 0 0 1 0 0 0 1 0 0 0 0 1 0 1 1 1 0 2 0 0 0 0 0 - - 0 0

39-Bronn. Recherches sur les lois d-evolution du monde organ 2 0 2 0 0 0 0 0 1 0 2 0 1 0 1 1 0 0 0 1 0 0 0 1 0 1 1 2 1 0 0 1 0 0 0 0 0 0 1 1 0 0 0 0 0 1 0 - - - - - 0 1 0 0 0 0 1 1 0 2 0 0 1 0 0 0 0 0 0 - - - - - 0 0 0 0 0 0 0 0 0 0 0 0 0 0 0 0 0 1 0 0 0 0 1 1 1 0 0 0 2 0 0 0 0 0 1 0 0 0 0 0 0 0 0 0 1 0 0 0 0 0 1 0 1 0 0 1 1 1 1 0 0 - - 0 0

40-Hitchcock. Elementary geology with an introductory notice 2 2 2 1 0 0 1 0 1 0 2 - - - - - - - - - - 0 0 1 0 1 1 1 1 0 0 1 0 0 0 0 1 0 0 1 0 0 0 1 1 0 0 1 1 0 1 2 0 1 0 0 0 0 1 1 0 1 2 0 1 0 0 0 0 0 0 1 1 0 0 0 0 0 0 0 0 0 0 0 0 0 0 0 1 0 0 0 0 1 0 1 0 0 1 0 0 0 0 0 0 0 0 0 0 0 ? 0 1 0 1 1 1 0 0 0 0 0 0 0 0 1 1 0 1 1 0 2 1 1 1 0 0 - - 0 0

41-Wallace. Attempts at a natural arrangement of birds, Tree 0 0 0 0 0 0 0 0 0 0 0 - - - - - - - - - - 0 0 0 0 - 0 0 0 0 0 0 ? 0 0 0 0 1 0 0 0 0 0 0 0 0 0 - - - - - 1 0 0 0 1 0 0 1 1 0 0 0 0 0 0 0 0 0 ? - - - - - - - 0 0 ? 1 0 0 0 0 0 0 1 0 0 1 0 1 0 0 0 0 1 0 0 1 0 0 - - - - - - - 1 0 0 1 0 0 0 0 0 1 0 0 1 0 0 0 0 1 ? 0 0 0 1 1 0 0 - - 0 0

42-Wallace. Attempts at a natural arrangement of birds, Tree 0 0 0 0 0 0 0 0 0 0 0 - - - - - - - - - - 0 0 0 0 - 0 0 0 0 0 0 ? 0 0 0 0 1 0 0 0 0 0 0 0 0 0 - - - - - 1 0 0 0 1 0 0 1 1 0 0 0 0 0 0 0 0 0 ? - - - - - - - 0 0 ? 1 0 0 0 0 0 0 1 0 0 1 0 1 0 0 0 0 1 0 0 1 0 0 - - - - - - - 1 0 0 1 0 0 0 0 0 1 0 0 1 0 0 0 0 1 ? 0 0 0 1 1 0 0 - - 0 0

43-Strickland. Memoirs of H. E. Strickland, Tree #1 - - - - - - 0 - - - - - - - - - - - - - - 0 0 1 1 - 0 0 1 1 0 0 0 0 0 0 0 0 0 0 0 0 0 0 1 1 0 0 0 0 0 2 1 1 0 0 1 0 1 1 1 0 0 0 0 1 0 0 0 0 ? - - - - - - - 0 0 0 1 0 0 0 1 1 1 1 1 0 1 0 0 0 1 0 0 1 0 0 1 0 0 - - - - - 1 1 1 0 0 0 0 0 0 0 0 1 0 0 1 0 0 0 1 ? 0 0 0 ? 1 1 0 0 - - 0 0

44-Strickland. Memoirs of H. E. Strickland, Tree #2 - - - - - - 0 - - - - - - - - - - - - - - 1 1 1 1 - 0 0 ? 1 0 0 0 0 0 0 0 1 0 0 0 0 0 0 1 1 0 0 0 0 0 2 1 0 0 0 1 0 0 1 1 0 0 0 0 1 0 0 0 0 ? - - - - - - - 0 0 0 1 ? 0 0 1 1 1 1 1 0 1 ? 2 0 1 0 0 ? ? ? ? 0 0 2 - - - - - - 1 0 1 0 0 0 1 1 0 1 0 0 1 0 0 0 1 ? 0 0 0 ? 1 1 0 0 - - 0 0

45-Darwin. 1859. On the origin of species I 0 2 1 0 1 1 0 1 1 0 0 - - - - - - - - - - 1 0 0 1 1 0 0 0 0 0 1 1 0 1 0 1 1 1 2 1 1 0 0 0 2 1 - - - - - 1 2 1 0 1 1 0 0 0 1 1 0 0 0 0 0 0 0 0 0 0 ? 0 2 2 0 0 1 1 1 ? 0 1 0 1 1 1 0 0 1 1 2 0 1 ? 0 0 1 1 0 0 1 2 1 0 1 1 1 1 1 1 0 0 0 0 1 1 0 0 0 1 0 0 0 0 1 1 1 1 2 1 2 1 0 1 - - 0 0

46-Macdonald. On metamorphosis of gasteropoda 0 2 0 0 1 0 1 - - - 0 - - - - - - - - - - 1 0 0 - - 0 0 1 0 0 1 1 0 1 0 0 1 - 0 0 1 0 0 0 0 1 - - - - - 1 2 1 0 1 1 0 0 0 0 0 0 0 0 0 1 0 0 0 - - - - - - - 0 1 0 1 0 0 1 0 0 0 1 0 0 1 1 2 0 1 0 0 1 0 0 0 0 0 - - - - - - - 1 0 0 0 0 0 0 0 0 1 0 0 1 0 0 0 0 0 0 1 0 0 0 0 0 0 - - 0 0

47-Trémaux. Origine et transformations de l-Homme et des au0 2 1 0 0 0 0 1 1 0 0 1 1 1 1 0 0 1 0 0 1 0 0 1 0 1 0 0 1 0 0 1 1 0 0 0 1 0 0 0 0 0 0 0 0 0 0 - - - - - 1 1 0 0 1 1 1 1 0 1 2 0 0 0 0 0 0 0 0 0 0 1 0 2 2 0 0 0 1 1 0 0 1 0 0 0 1 0 0 1 ? ? 0 ? ? 0 0 1 1 0 1 0 2 0 1 1 0 1 1 0 1 0 0 0 0 1 1 1 0 0 1 0 0 1 0 0 1 1 1 2 1 1 1 0 0 - - 0 0

48-Gaudry. Considerations generales sur les animaux fossiles 0 1 1 0 0 0 0 0 1 0 0 - - - - - - - - - - 0 0 0 0 1 0 0 1 0 0 1 1 0 0 0 1 0 0 0 0 0 0 0 0 1 0 - - - - - 1 0 0 0 1 0 1 1 0 1 2 0 0 0 0 0 0 0 0 1 0 1 0 2 - - 0 0 1 1 0 0 0 0 0 0 1 0 0 1 0 1 0 0 0 0 1 0 0 0 0 0 2 1 ? ? 1 1 1 1 1 0 0 1 0 1 1 1 1 0 0 1 0 0 0 1 1 1 1 2 1 1 1 0 0 - - 0 0

49-Gaudry. Considerations generales sur les animaux fossiles 0 1 1 0 0 0 0 0 1 0 0 - - - - - - - - - - 0 0 0 0 1 0 0 1 0 0 1 1 0 0 0 1 0 0 0 0 0 0 0 0 1 0 - - - - - 1 0 0 0 1 0 1 1 0 1 2 0 0 0 0 0 0 0 0 1 0 1 0 2 - - 0 0 1 1 0 0 0 0 0 0 1 0 0 1 0 1 0 0 0 0 1 0 0 0 0 0 2 1 ? ? 1 1 1 1 1 0 0 1 0 1 1 1 1 0 0 1 0 0 0 1 1 1 1 2 1 1 1 0 0 - - 0 0

50-Gaudry. Considerations generales sur les animaux fossiles 0 1 1 0 0 0 0 0 1 0 0 - - - - - - - - - - 0 0 0 0 1 0 0 1 0 0 1 1 0 0 0 1 0 0 0 0 0 0 0 0 1 0 - - - - - 1 0 0 0 1 0 1 1 0 1 2 0 0 0 0 0 0 0 0 1 0 1 0 2 - - 0 0 1 1 0 0 0 0 0 0 1 0 0 1 0 1 0 0 0 0 1 0 0 0 0 0 2 1 ? ? 1 1 1 1 1 0 0 1 0 1 1 1 1 0 0 1 0 0 0 1 1 1 1 2 1 1 1 0 0 - - 0 0

51-Gaudry. Considerations generales sur les animaux fossiles 0 1 1 0 0 0 0 0 1 0 0 - - - - - - - - - - 0 0 0 0 1 0 0 1 0 0 1 1 0 0 0 1 0 0 0 0 0 0 0 0 1 0 - - - - - 1 0 0 0 1 0 1 1 0 1 2 0 0 0 0 0 0 0 0 1 0 1 0 2 - - 0 0 1 1 0 0 0 0 0 0 1 0 0 1 0 1 0 0 0 0 1 0 0 0 0 0 2 1 ? ? 1 1 1 1 1 0 0 1 0 1 1 1 1 0 0 1 0 0 0 1 1 1 1 2 1 1 1 0 0 - - 0 0

52-Gaudry. Considerations generales sur les animaux fossiles 0 1 1 0 0 0 0 0 1 0 0 - - - - - - - - - - 0 0 1 0 1 0 0 1 0 0 1 1 0 0 0 1 0 0 0 0 0 0 0 0 1 0 - - - - - 1 0 0 0 1 0 1 1 0 1 2 0 0 0 0 0 0 0 0 1 0 1 0 2 - - 0 0 1 1 0 0 0 0 0 0 1 0 0 1 0 1 0 0 0 0 1 0 0 0 0 0 2 1 ? ? 1 1 1 1 1 0 0 1 0 1 1 1 1 0 0 1 0 0 0 1 1 1 1 2 1 1 1 0 0 - - 0 0

53-Haeckel. Generelle morphologie der organismen, Tree #1 2 0 2 0 0 0 0 0 1 0 2 0 1 0 0 1 0 0 0 0 1 0 0 1 1 ? 1 2 1 0 0 1 0 0 0 0 1 0 1 1 0 0 0 1 0 1 0 - - - - - 0 1 0 0 0 0 1 1 0 2 0 0 0 0 0 0 1 0 0 ? ? 0 1 0 1 0 0 0 1 ? 0 0 0 0 0 0 1 0 0 ? 0 0 0 1 ? 0 1 0 0 0 0 0 2 0 ? ? 0 1 ? 0 1 0 0 0 0 0 0 0 0 0 0 1 0 1 1 1 1 1 0 2 1 1 1 0 0 - - 0 0

54-Haeckel. Generelle morphologie der organismen, Tree #2 2 0 2 0 0 0 0 0 1 0 2 0 1 0 0 1 0 0 0 0 1 0 0 1 1 1 1 2 1 0 0 1 0 0 0 0 1 0 1 1 0 0 0 1 0 1 0 - - - - - 0 1 0 0 ? 0 1 1 0 2 0 0 0 0 0 0 0 0 0 ? ? 0 1 0 - - 0 0 1 ? 0 0 0 0 0 0 0 0 0 ? 0 0 0 1 0 0 1 0 0 0 0 0 2 0 ? ? 0 1 ? 0 1 0 0 0 0 0 0 0 1 0 0 1 0 1 1 1 1 1 0 2 1 1 1 0 0 - - 0 0

55-Haeckel. Generelle morphologie der organismen, Tree #3 2 0 2 0 0 0 0 0 1 0 2 0 1 0 0 1 0 0 0 0 1 0 0 1 1 1 1 2 1 0 0 1 0 0 0 0 1 0 1 1 0 0 0 1 0 1 0 - - - - - 0 1 0 0 ? 0 1 1 0 2 0 0 0 0 0 0 0 0 0 ? ? 0 1 0 - - 0 0 1 ? 0 0 0 0 0 0 1 0 0 ? 0 0 0 1 ? 0 1 0 0 0 0 0 2 0 ? ? ? 1 ? ? 1 0 0 0 0 0 0 0 1 0 0 1 0 1 1 1 1 1 0 2 1 1 1 0 0 - - 0 0

56-Haeckel. Generelle morphologie der organismen, Tree #4 2 0 2 0 0 0 0 0 1 0 2 0 1 0 0 1 0 1 0 0 1 0 0 1 1 0 1 2 1 0 0 1 0 0 0 0 1 0 1 1 0 0 0 1 0 1 0 - - - - - 1 1 0 0 ? 0 1 1 0 1 2 0 0 0 0 0 0 0 0 ? ? 0 1 0 - - 0 0 1 ? 0 0 0 0 0 0 1 0 0 ? 0 0 0 1 ? 0 1 0 0 0 0 0 2 0 ? ? 0 1 ? 0 1 0 0 0 0 0 0 0 1 0 0 1 0 1 1 1 1 1 0 2 1 1 1 0 0 - - 0 0

57-Haeckel. Generelle morphologie der organismen, Tree #5 2 0 2 0 0 0 0 0 1 0 2 0 1 0 0 1 0 0 0 0 1 0 0 1 1 1 1 2 1 0 0 1 0 0 0 0 1 0 1 1 0 0 0 1 0 1 0 - - - - - 0 1 0 0 ? 0 1 1 0 2 0 0 0 0 0 0 0 0 0 ? ? 0 1 0 - - 0 0 1 ? 0 0 0 0 0 0 1 0 0 ? 0 0 0 1 ? 0 1 0 0 0 0 0 2 0 ? ? ? 1 ? ? 1 0 0 0 0 0 0 0 1 0 0 1 0 1 1 1 1 1 0 2 1 1 1 0 0 - - 0 0

58-Haeckel. Generelle morphologie der organismen, Tree #6 2 0 2 0 0 0 0 0 1 0 2 0 1 0 0 1 0 0 0 0 1 0 0 1 1 1 1 2 1 0 0 1 0 0 0 0 1 0 1 1 0 0 0 1 0 1 0 - - - - - 0 1 0 0 ? 0 1 1 0 2 0 0 0 0 0 0 0 0 0 ? ? 0 1 0 - - 0 0 1 ? 0 0 0 0 0 0 0 0 0 ? 0 2 0 1 ? 0 1 0 0 0 0 0 2 0 ? ? ? 1 ? ? 1 0 0 0 0 0 0 0 1 0 0 1 0 1 1 1 1 1 0 2 1 1 1 0 0 - - 0 0

59-Haeckel. Generelle morphologie der organismen, Tree #7 2 0 2 0 0 0 0 0 1 0 2 0 1 0 0 1 0 1 0 0 1 0 0 1 0 1 1 2 1 0 1 1 0 0 0 0 1 0 1 1 0 0 0 1 0 1 0 - - - - - 0 1 0 0 0 0 1 1 0 1 2 0 1 0 0 0 0 0 0 ? ? 0 1 0 1 0 0 0 1 ? 0 0 0 0 0 0 1 0 0 ? 0 0 0 1 ? 0 1 0 0 0 0 0 2 0 ? ? 0 1 ? 0 1 0 0 0 0 0 0 0 1 0 0 1 0 1 1 1 1 1 0 2 1 1 1 0 0 - - 0 0

60-Haeckel. Generelle morphologie der organismen, Tree #8 2 0 2 0 0 0 0 0 1 0 2 0 1 0 0 1 0 0 0 0 1 0 0 1 0 1 1 2 1 0 1 1 0 0 0 0 1 0 1 1 0 0 0 1 0 1 0 - - - - - 0 1 0 0 0 0 1 1 0 2 0 0 1 0 0 0 0 0 0 ? ? 0 1 0 1 0 0 0 1 ? 0 0 0 0 0 0 0 0 0 ? 0 2 0 1 ? 0 1 0 0 0 0 0 2 0 ? ? 0 1 ? 0 1 0 0 0 0 0 0 0 1 0 0 1 0 1 1 1 1 1 0 2 1 1 1 0 0 - - 0 0

61-Haeckel. Histoire de la creation des etres organises, Tree 2 0 2 1 0 0 0 0 1 0 0 - - - - - - - - - - 0 0 1 0 0 0 0 1 0 0 1 0 0 0 0 1 0 1 1 0 0 0 0 0 1 0 - - - - - 0 1 0 0 0 0 1 1 0 2 0 0 1 0 0 0 0 0 0 1 1 0 1 0 1 0 0 0 1 0 0 0 0 0 0 0 0 0 0 0 0 1 0 1 0 0 1 0 1 0 0 0 2 0 0 0 0 0 ? 0 1 0 0 0 ? 0 0 0 0 0 0 1 0 1 0 1 1 1 0 2 1 1 1 0 0 - - 0 0

62-Haeckel. Histoire de la creation des etres organises, Tree 2 0 2 1 0 1 0 0 1 0 0 - - - - - - - - - - 0 0 1 0 1 0 0 1 0 0 1 0 0 0 0 1 0 1 1 0 0 0 0 0 1 0 - - - - - 0 1 0 0 0 0 1 1 0 2 0 0 1 0 0 0 0 0 0 1 1 0 1 0 2 0 0 0 1 0 0 0 0 0 0 0 1 0 0 0 0 1 0 1 0 0 1 0 1 0 0 0 2 0 0 0 0 0 ? 0 1 0 0 0 ? 0 0 0 0 0 0 1 0 1 0 1 1 1 0 2 1 1 1 0 0 - - 0 0

63-Haeckel. Histoire de la creation des etres organises, Tree 2 0 2 1 0 0 0 0 1 0 0 - - - - - - - - - - 0 0 1 0 0 0 0 1 0 1 1 0 0 0 0 1 0 1 1 0 0 0 0 0 1 0 - - - - - 0 1 0 0 0 0 1 1 0 2 0 0 1 0 0 0 0 0 0 1 1 0 1 0 - - 0 0 1 0 0 0 0 0 0 0 0 0 0 0 0 1 0 1 0 0 1 0 0 0 0 0 2 0 0 0 0 0 ? 0 1 0 0 0 ? 0 0 0 1 0 0 1 0 1 0 1 1 1 0 2 1 1 1 0 0 - - 0 0

64-Haeckel. Histoire de la creation des etres organises, Tree 2 0 2 1 0 0 0 0 1 0 0 1 1 0 0 0 0 1 1 0 1 0 0 1 0 1 1 2 1 0 0 1 0 0 0 0 1 0 1 1 0 0 0 1 0 1 0 - - - - - 0 1 0 0 0 0 1 1 0 1 2 0 1 0 0 0 1 0 0 1 1 0 1 0 - - 0 0 1 0 0 0 0 0 0 0 0 0 0 0 0 1 0 1 0 0 1 0 0 0 0 0 2 0 0 0 0 0 ? 0 1 0 0 0 ? 0 0 0 1 0 0 1 0 1 1 1 1 1 0 2 1 1 1 0 0 - - 0 0

65-Haeckel. Histoire de la creation des etres organises, Tree 2 0 2 0 0 0 0 0 1 0 0 1 1 0 0 0 0 0 1 0 1 0 0 1 0 0 1 2 1 0 0 1 0 0 0 0 1 0 1 1 0 0 0 1 0 1 0 - - - - - 0 1 0 0 0 0 1 1 0 1 2 0 0 0 0 0 0 0 0 1 1 0 1 0 2 0 0 0 1 0 0 0 0 0 0 0 0 0 0 0 0 1 0 1 0 0 1 0 0 0 0 0 2 0 0 0 0 0 ? 0 1 0 0 0 ? 0 0 0 1 0 0 1 0 1 1 1 1 1 0 2 1 1 1 0 0 - - 0 0

66-Haeckel. Histoire de la creation des etres organises, Tree 2 0 2 1 0 0 0 0 1 0 0 - - - - - - - - - - 0 0 1 0 0 0 0 1 0 1 1 0 0 0 0 1 0 1 1 0 0 0 0 0 1 0 - - - - - 0 1 0 0 0 0 1 1 0 2 0 0 1 0 0 0 0 0 0 1 1 0 1 0 1 0 0 0 1 0 0 0 0 0 0 0 0 0 0 0 0 1 0 1 0 0 1 0 0 0 0 0 2 0 0 0 0 0 ? 0 1 0 0 0 ? 0 0 0 1 0 0 1 0 1 0 1 1 1 0 2 1 1 1 0 0 - - 0 0

67-Haeckel. Histoire de la creation des etres organises, Tree 2 0 2 0 0 0 0 0 1 0 0 - - - - - - - - - - 0 0 1 0 0 0 0 1 0 1 1 0 0 0 0 1 0 1 1 0 0 0 0 0 1 0 - - - - - 0 1 0 0 0 0 1 1 0 2 0 0 1 0 0 0 0 0 0 1 1 0 1 0 - - 0 0 1 0 0 0 0 0 0 0 0 0 0 0 0 1 0 1 0 0 1 0 0 0 0 0 2 0 0 0 0 0 ? 0 1 0 0 0 ? 0 0 0 1 0 0 1 0 1 0 1 1 1 0 2 1 1 1 0 0 - - 0 0

68-Haeckel. Histoire de la creation des etres organises, Tree 2 0 2 0 0 0 0 0 1 0 0 - - - - - - - - - - 0 0 1 0 0 0 0 1 0 1 1 0 0 0 0 1 0 1 1 0 0 0 0 0 1 0 - - - - - 0 1 0 0 0 0 1 1 0 2 0 0 1 0 0 0 0 0 0 1 1 0 1 0 - - 0 0 1 0 0 0 0 0 0 0 0 0 0 0 0 1 0 1 0 0 1 0 0 0 0 0 2 0 0 0 0 0 ? 0 1 0 0 0 ? 0 0 0 1 0 0 1 0 1 0 1 1 1 0 2 1 1 1 0 0 - - 0 0

69-Haeckel. Histoire de la creation des etres organises, Tree 2 0 2 0 0 0 0 0 1 0 0 - - - - - - - - - - 0 0 1 0 0 0 0 1 0 1 1 0 0 0 0 1 0 1 1 0 0 0 0 0 1 0 - - - - - 0 1 0 0 0 0 1 1 0 2 0 0 1 0 0 0 0 0 0 1 1 0 1 0 - - 0 0 1 0 0 0 0 0 0 0 0 0 0 0 0 1 0 1 0 0 1 0 0 0 0 0 2 0 0 0 0 0 ? 0 1 0 0 0 ? 0 0 0 1 0 0 1 0 1 0 1 1 1 0 2 1 1 1 0 0 - - 0 0

70-Haeckel. Histoire de la creation des etres organises, Tree 2 0 2 0 0 0 0 0 1 0 0 - - - - - - - - - - 0 0 1 0 0 0 0 1 0 1 1 0 0 0 0 1 0 1 1 0 0 0 0 0 1 0 - - - - - 0 1 0 0 0 0 1 1 0 2 0 0 1 0 0 0 0 0 0 1 1 0 1 0 - - 0 0 1 0 0 0 0 0 0 0 0 0 0 0 0 1 0 1 0 0 1 0 0 0 0 0 2 0 0 0 0 0 ? 0 1 0 0 0 ? 0 0 0 1 0 0 1 0 1 0 1 1 1 0 2 1 1 1 0 0 - - 0 0

71-Haeckel. Histoire de la creation des etres organises, Tree 2 0 2 0 0 0 0 0 1 0 0 - - - - - - - - - - 0 0 1 0 0 0 0 1 0 1 1 0 0 0 0 1 0 1 1 0 0 0 0 0 1 0 - - - - - 0 1 0 0 0 0 1 1 0 2 0 0 1 0 0 0 0 0 0 1 1 0 1 0 - - 0 0 1 0 0 0 0 0 0 0 0 0 0 0 0 1 0 1 0 0 1 0 0 0 0 0 2 0 0 0 0 0 ? 0 1 0 0 0 ? 0 0 0 1 0 0 1 0 1 0 1 1 1 0 2 1 1 1 0 0 - - 0 0

72-Haeckel. Histoire de la creation des etres organises, Tree 2 0 2 0 0 0 0 0 1 0 0 - - - - - - - - - - 0 0 1 0 0 0 0 1 0 1 1 0 0 0 0 1 0 1 1 0 0 0 0 0 1 0 - - - - - 0 1 0 0 0 0 1 1 0 2 0 0 1 0 0 0 0 0 0 1 1 0 1 0 - - 0 0 1 0 0 0 0 0 0 0 1 0 0 0 0 1 0 1 0 0 1 0 0 0 0 0 2 0 0 0 0 0 ? 0 1 0 0 0 ? 0 0 0 1 0 0 1 0 1 0 1 1 1 0 2 1 1 1 0 0 - - 0 0

73-Haeckel. Histoire de la creation des etres organises, Tree 2 0 2 0 0 0 0 0 1 0 0 - - - - - - - - - - 0 0 1 0 0 0 0 1 0 1 1 0 0 0 0 1 0 1 1 0 0 0 0 0 1 0 - - - - - 0 1 0 0 0 0 1 1 0 2 0 0 1 0 0 0 0 0 0 1 1 0 1 0 1 0 0 0 1 0 0 0 0 0 0 0 0 0 0 0 0 1 0 1 0 0 1 0 0 0 0 0 2 0 0 0 0 0 ? 0 1 0 0 0 ? 0 0 0 1 0 0 1 0 1 0 1 1 1 0 2 1 1 1 0 0 - - 0 0

74-Haeckel. Histoire de la creation des etres organises, Tree 2 0 2 0 0 0 0 0 1 0 0 - - - - - - - - - - 0 0 1 0 0 0 0 1 0 1 1 0 0 0 0 1 0 1 1 0 0 0 0 0 1 0 - - - - - 0 1 0 0 0 0 1 1 0 2 0 0 1 0 0 0 0 0 0 1 1 0 1 0 1 0 0 0 1 0 0 0 0 0 0 0 0 0 0 0 0 1 0 1 0 0 1 0 0 0 0 0 2 0 0 0 0 0 ? 0 1 0 0 0 ? 0 0 0 1 0 0 1 0 1 0 1 1 1 0 2 1 1 1 0 0 - - 0 0

75-Haeckel. Histoire de la creation des etres organises, Tree 2 0 2 0 0 0 0 0 1 0 0 0 1 0 0 0 0 1 1 0 1 0 0 1 0 1 1 0 1 0 0 1 0 0 0 0 1 0 1 1 0 0 0 1 0 1 0 - - - - - 0 1 0 0 0 0 1 1 0 1 2 0 1 0 0 0 1 0 0 1 1 0 1 0 1 0 0 0 1 0 0 0 0 0 0 0 0 0 0 0 0 1 0 1 0 0 1 0 0 0 0 0 2 0 0 0 0 0 ? 0 1 0 0 0 ? 0 0 0 1 0 0 1 0 1 1 1 1 1 0 2 1 1 1 0 0 - - 0 0

76-Haeckel. Histoire de la creation des etres organises, Tree 2 2 1 0 0 0 0 1 1 0 0 - - - - - - - - - - 0 0 1 0 0 0 0 1 0 0 1 0 0 0 0 1 0 1 1 0 0 0 0 0 1 0 - - - - - 0 1 0 0 0 0 1 1 0 2 0 1 1 0 1 0 0 0 0 1 1 0 1 0 0 1 0 0 0 0 0 0 0 0 0 0 0 0 0 0 0 1 0 1 0 0 0 1 0 1 0 0 2 0 0 0 0 1 0 0 1 0 1 0 1 1 0 0 1 1 0 1 0 1 1 1 1 1 0 2 0 1 1 0 0 - - 0 0

77-Garrod. On some points of the anatomy of the parrots 0 2 1 0 1 0 0 1 1 1 0 - - - - - - - - - - 1 0 0 1 1 0 0 0 0 0 1 1 0 1 0 1 1 1 2 0 1 0 0 0 2 1 - - - - - 1 2 1 0 1 1 0 0 0 1 0 0 0 0 0 0 0 0 0 0 0 0 0 ? - - 0 1 1 1 1 0 1 0 1 1 1 0 0 1 1 2 0 1 0 0 0 1 1 0 0 0 2 1 0 ? 1 1 1 1 1 1 0 0 0 0 0 0 1 0 0 1 0 0 0 1 1 1 1 2 1 2 0 1 0 - - 1 1

78-Haeckel. Anthropogenie 2 0 2 1 0 0 0 0 1 0 2 0 0 0 0 1 0 0 0 1 0 0 0 1 0 1 1 2 1 0 0 1 0 1 0 0 1 0 1 1 0 0 1 1 1 1 0 - - - - - 0 1 0 0 0 0 1 1 0 2 0 0 1 0 0 0 0 0 0 1 1 0 1 0 0 0 0 0 1 0 0 0 0 0 0 0 0 0 0 0 0 1 0 1 0 0 1 0 0 0 0 0 1 1 0 0 0 0 ? 0 1 0 0 0 1 0 0 0 1 0 0 1 0 1 1 1 1 1 0 2 1 1 1 0 0 - - 0 0

79-Darwin. On the origin of species VI 0 2 1 0 1 1 0 1 1 0 0 - - - - - - - - - - 1 0 0 1 1 0 0 ? 0 0 1 1 0 1 0 1 1 1 2 1 1 0 0 0 2 1 - - - - - 1 2 1 0 1 1 0 ? 0 1 1 0 0 0 0 0 0 0 0 0 0 ? 0 2 2 0 0 1 1 1 ? 0 1 0 1 1 1 0 0 1 ? 2 0 1 ? 0 0 1 1 0 0 1 2 1 0 1 1 1 1 1 1 0 0 0 0 1 1 0 0 0 1 0 0 0 0 1 1 1 1 2 1 2 1 0 1 - - 0 0

80-Lankester. Limulus, an arachnid 0 0 2 0 1 0 1 1 1 0 0 1 1 0 0 0 1 0 1 0 1 0 0 1 1 1 0 0 0 0 0 1 1 0 1 0 1 1 1 2 0 1 0 0 0 2 1 - - - - - 0 1 0 0 0 0 0 0 0 2 0 0 0 0 0 0 0 0 0 1 0 0 0 0 - - 0 0 1 1 1 0 0 0 0 1 1 0 0 0 1 1 0 1 0 0 0 0 1 0 0 0 2 0 0 1 1 1 1 0 1 1 0 0 0 0 0 0 1 0 0 1 0 0 0 1 1 1 1 2 1 1 1 0 0 - - 0 1

81-Bennett. On the affinities and classification of algae 2 0 2 0 0 0 1 ? 0 0 0 1 1 0 0 0 1 0 1 0 0 0 0 1 0 0 0 0 1 0 0 0 0 0 0 0 1 0 1 1 0 0 0 0 0 1 0 - - - - - 0 0 0 0 0 0 1 1 0 2 0 0 1 0 0 0 0 0 0 1 0 1 1 0 - - ? 0 1 0 0 0 0 ? ? 0 1 0 0 0 0 1 0 ? 0 0 1 0 0 ? 0 0 2 ? 0 ? 0 1 ? 0 1 0 0 0 - 0 0 0 1 0 0 1 0 0 0 1 1 1 0 2 0 1 1 0 0 - - 0 0

82-Hackel. Monographiae phanerogamarum 2 0 2 0 0 0 0 0 1 0 0 0 1 0 0 1 1 0 1 0 1 0 0 1 0 1 0 0 1 0 0 0 0 0 0 0 1 0 1 1 0 0 0 0 0 1 0 - - - - - 0 1 0 0 0 0 1 1 0 2 0 0 0 0 0 0 1 0 0 1 ? ? 0 0 - - 0 0 1 1 0 0 0 0 0 0 1 0 0 0 0 1 0 1 0 0 1 0 0 0 0 0 2 1 0 0 0 1 ? 0 1 0 0 0 0 0 0 0 1 0 0 1 0 0 0 1 1 1 0 2 0 1 1 0 0 - - 0 0

83-Dollo. Sur la phylogenie des dipneustes, Tree #1 0 2 1 0 0 0 0 1 1 0 0 1 1 1 1 0 1 0 1 1 1 1 0 0 1 1 0 0 1 0 0 1 0 0 1 0 1 1 1 2 0 0 0 0 0 2 1 - - - - - 1 2 1 0 0 0 0 1 0 2 0 0 0 0 0 0 0 0 0 1 0 0 0 0 - - 0 0 1 1 ? 0 1 0 0 0 1 0 0 1 1 2 0 1 0 0 0 0 0 0 0 0 2 1 ? ? 0 1 1 1 1 0 0 0 0 1 0 0 1 0 0 1 0 0 0 1 1 1 1 2 1 2 1 0 0 - - 0 0

84-Dollo. Sur la phylogenie des dipneustes, Tree #2 2 0 2 0 0 0 0 0 1 0 0 - - - - - - - - - - 0 0 1 0 0 0 0 0 0 0 1 1 0 0 0 1 0 0 1 0 0 0 0 0 2 0 - - - - - 0 1 0 1 0 0 1 1 0 2 0 0 0 0 0 0 0 0 0 1 1 0 0 0 1 0 0 0 1 0 0 0 1 0 0 0 0 0 0 0 0 1 0 0 0 0 0 0 0 0 0 0 2 0 0 ? 0 1 1 0 1 0 1 0 0 0 0 0 1 0 0 1 0 0 0 1 1 1 0 2 1 1 1 0 0 - - 0 0

85-Mitchell. On the intestinal tracks of birds 2 0 2 0 0 0 0 0 1 0 0 - - - - - - - - - - 0 0 1 - - 0 0 1 0 0 1 0 0 0 0 1 1 1 2 0 1 0 0 0 0 0 - - - - - 1 1 0 0 0 0 1 1 0 2 0 0 0 0 0 0 0 0 0 1 0 0 0 0 - - 0 0 1 1 1 0 0 0 0 0 1 0 0 0 0 1 0 1 0 0 1 0 0 0 0 0 2 1 ? ? 0 1 1 1 1 0 0 0 0 0 0 0 1 0 0 1 0 0 0 0 1 1 0 2 0 1 1 1 0 - - 0 1

86-Patten. The evolution of the vertebrates and their kin, Tree 2 0 2 0 0 0 0 0 1 0 1 - - - - - - - - - - 0 0 1 0 1 0 1 1 0 0 1 0 0 0 0 1 0 1 1 0 0 0 0 1 1 0 1 1 0 0 0 0 1 0 0 0 0 0 1 0 1 2 0 1 0 0 0 0 0 0 1 1 1 1 0 2 0 0 0 1 0 0 0 0 0 0 0 0 0 0 0 0 1 0 1 0 0 1 0 0 0 0 0 2 0 0 1 0 1 0 0 1 0 0 0 0 0 0 0 1 0 0 1 0 1 0 1 1 1 0 2 1 1 1 0 0 - - 0 1

87-Patten. The evolution of the vertebrates and their kin, Tree 2 0 2 0 0 0 0 1 1 0 1 - - - - - - - - - - 1 0 1 0 1 0 1 0 0 0 1 1 0 1 0 1 ? 1 2 0 0 0 0 1 1 0 1 0 0 0 0 1 1 0 0 0 0 0 0 0 1 0 0 1 1 0 0 0 0 0 1 ? 0 1 0 0 0 0 0 1 0 0 0 0 0 0 0 1 0 0 0 0 1 0 1 0 0 0 0 0 0 0 0 2 0 0 1 0 1 0 0 1 1 0 0 0 0 0 0 1 0 0 1 0 1 0 1 0 1 0 2 1 1 1 0 0 - - 0 1

88-Bessey. The phylogenetic taxonomy of flowering plants 2 0 2 0 0 0 0 0 1 0 1 - - - - - - - - - - 0 0 1 0 0 1 1 0 0 0 0 0 0 0 0 1 0 1 1 0 0 0 1 1 0 0 1 1 0 0 2 0 1 0 0 0 0 1 1 0 2 0 0 0 0 0 0 0 0 0 1 1 1 1 0 - - 0 0 1 1 0 0 0 0 0 0 1 0 0 0 0 1 0 1 0 0 1 0 0 0 0 0 2 0 0 ? 0 1 ? 0 1 0 0 0 0 0 0 0 1 0 0 1 0 0 1 0 1 1 0 2 0 1 1 0 0 - - 0 0

89-Gruenberg. Elementary biology, Tree #1 2 0 1 0 0 0 0 1 1 1 2 0 1 0 0 0 0 0 0 1 1 1 0 1 1 1 1 2 0 1 0 1 0 0 0 0 1 0 1 1 0 0 0 1 1 2 0 - - - - - 1 2 1 0 0 1 1 1 1 2 0 0 0 0 0 0 0 1 0 0 1 0 0 0 - - 0 0 1 0 0 0 0 0 0 0 1 1 0 0 1 2 0 1 0 0 1 0 0 0 0 0 2 1 0 ? 0 1 ? 0 1 0 0 0 - 0 0 0 1 0 0 1 0 1 1 1 1 1 0 2 0 1 1 0 0 - - 0 0

90-Gruenberg. Elementary biology, Tree #2 2 0 1 0 0 0 0 1 1 1 2 0 1 0 0 0 0 0 0 1 1 1 0 1 1 1 1 2 0 1 0 1 0 0 0 0 1 0 1 1 0 0 0 1 1 2 0 - - - - - 1 2 1 0 0 1 1 1 1 2 0 0 0 0 0 0 0 1 0 0 1 0 0 0 0 0 0 0 1 0 0 0 0 0 0 0 1 1 0 0 1 2 0 1 0 0 1 0 0 0 0 0 2 1 0 ? 0 1 ? 0 1 0 0 0 0 0 0 0 1 0 0 1 0 1 1 1 1 1 0 2 0 1 1 0 0 - - 0 0

91-Tillyard. The panorpoid complex 2 2 2 0 1 0 0 1 1 1 1 - - - - - - - - - - 0 0 1 0 1 0 1 0 0 0 1 0 0 1 0 1 0 1 2 0 1 0 0 1 2 0 1 1 0 0 2 1 1 0 0 1 0 0 0 0 1 2 0 0 0 0 0 0 0 0 0 1 1 0 0 - - 0 0 1 1 1 0 ? 0 0 1 1 0 0 1 1 1 0 1 0 0 0 0 0 0 0 0 2 0 0 ? 0 1 1 0 1 1 0 0 0 0 0 0 1 0 0 1 0 0 0 1 1 1 0 2 1 1 0 0 1 - - ? 1

92-Tillyard. A new classification of the order perlaria 0 2 1 0 1 0 0 1 1 1 0 - - - - - - - - - - 1 0 0 1 1 0 0 0 1 0 1 1 0 1 0 1 1 1 2 0 1 0 0 0 2 1 - - - - - 1 2 1 0 1 1 0 0 0 1 0 0 1 0 0 0 0 0 0 0 0 0 0 ? - - 0 1 1 1 1 0 1 0 0 0 1 0 0 1 1 2 0 1 0 0 0 0 1 0 0 0 2 1 0 1 1 1 1 1 1 1 0 0 0 0 0 0 1 0 0 1 0 0 0 1 1 1 1 2 ? 2 0 1 1 - - 1 1

93-Camp. Classification of the lizards 0 2 1 0 1 0 0 1 1 0 0 - - - - - - - - - - 1 0 0 0 1 0 0 0 0 0 1 ? 0 1 0 1 1 1 2 0 1 0 0 0 0 0 - - - - - 1 2 1 0 1 1 0 0 0 1 1 0 0 0 0 0 0 0 0 0 1 0 0 ? - - 0 0 1 1 0 0 0 0 0 0 1 0 0 1 1 2 0 1 0 0 1 0 0 0 0 0 2 1 0 1 1 1 ? 1 1 0 0 0 0 0 0 0 1 0 0 1 0 0 0 0 1 1 1 2 1 1 1 0 0 - - 0 0

94-Matthew. The phylogeny of dogs, Tree #1 2 2 2 0 0 0 0 0 1 0 0 - - - - - - - - - - 0 0 1 0 1 0 0 1 0 0 1 0 0 0 0 1 0 1 1 0 0 0 0 0 0 0 - - - - - 0 1 0 0 0 0 1 1 0 1 2 0 0 0 0 0 0 0 0 1 1 1 0 0 - - 0 0 1 ? 0 0 0 0 0 0 1 0 0 0 0 2 0 1 0 0 1 0 0 0 0 0 2 0 0 ? 0 1 ? 0 1 0 0 0 0 1 1 0 1 0 0 1 0 1 0 0 1 1 0 2 1 1 1 0 0 - - 0 0

95-Matthew. The phylogeny of dogs, Tree #2 2 0 2 0 0 0 0 0 1 0 1 - - - - - - - - - - 0 0 1 0 1 1 1 1 0 0 1 0 0 0 0 1 0 1 1 0 0 0 0 1 0 0 - - - - - 0 1 0 0 0 0 1 1 0 1 2 0 0 0 0 0 0 0 0 1 1 1 0 0 - - 0 0 1 0 0 0 0 0 0 0 1 0 0 0 0 1 0 1 0 0 1 0 0 0 0 0 2 0 0 ? 0 1 ? 0 1 0 0 0 0 0 0 0 1 0 0 1 0 1 0 0 1 1 0 2 1 1 1 0 0 - - 0 0

96-Rosa. L-Ologénèse, Tree #1 2 2 2 0 0 0 0 ? 1 0 0 - - - - - - - - - - 0 0 0 0 1 0 0 1 0 0 0 1 0 0 0 1 0 ? 0 ? 0 0 0 0 2 ? - - - - - 1 1 0 0 0 1 1 1 0 1 0 0 1 0 0 0 0 0 0 0 1 ? 1 1 - - 0 1 1 1 0 0 0 1 0 0 1 0 0 1 1 2 0 0 0 0 1 0 1 0 1 0 2 0 1 1 1 1 0 1 1 0 0 0 0 1 0 0 0 0 1 0 0 1 0 1 1 1 1 2 1 1 1 0 0 0 1 0 0

97-Rosa. L-Ologénèse, Tree #2 2 2 2 0 0 0 0 ? 1 0 0 - - - - - - - - - - 0 0 0 0 1 0 0 1 0 0 0 1 0 0 0 1 0 ? 0 ? 0 0 0 0 2 ? - - - - - 1 1 0 0 0 1 1 1 0 1 0 0 1 1 0 0 0 0 0 0 1 ? 1 1 - - 0 1 1 1 0 0 0 1 0 0 1 0 0 1 1 2 0 0 0 0 1 0 1 0 1 0 2 0 1 1 1 1 0 1 1 0 0 0 0 1 0 0 0 0 1 0 0 1 0 1 1 1 1 2 1 1 1 0 0 0 1 0 0

98-Rosa. L-Ologénèse, Tree #3 2 2 2 0 0 0 0 ? 1 0 0 - - - - - - - - - - 0 0 0 0 1 0 0 1 0 0 0 1 0 0 0 1 0 ? 0 ? 0 0 0 0 2 ? - - - - - 1 1 0 0 0 1 1 1 0 1 0 0 1 0 0 0 0 1 0 0 1 ? 1 1 - - 0 1 1 1 0 0 0 1 0 0 1 0 0 1 1 2 0 0 0 0 1 0 1 0 1 0 2 0 1 1 1 1 0 1 1 0 0 0 0 1 0 0 0 0 1 0 0 1 0 1 1 1 1 2 1 1 1 0 0 0 1 0 0

99-Rosa. L-Ologénèse, Tree #4 2 2 2 0 1 0 0 0 1 0 0 - - - - - - - - - - 0 0 0 0 1 0 0 1 0 0 0 1 0 1 0 1 0 ? 0 ? 0 0 0 0 2 ? - - - - - 1 1 0 0 0 1 1 1 0 1 0 0 1 0 0 0 0 0 0 0 1 ? 1 1 - - 0 1 1 1 0 0 0 1 0 0 1 0 0 1 1 2 0 0 0 0 1 0 1 0 1 0 2 0 1 1 1 1 0 1 1 0 0 0 0 1 0 0 0 0 1 0 0 1 0 1 1 1 1 2 1 1 1 0 0 0 1 0 0

100-Rosa. L-Ologénèse, Tree #5 2 2 2 0 0 0 0 ? 1 0 0 - - - - - - - - - - 0 0 0 0 1 0 0 1 0 0 0 1 0 0 0 1 0 ? 0 ? 0 0 0 0 2 ? - - - - - 1 1 0 0 0 1 1 1 0 1 1 0 1 0 0 0 0 0 0 0 1 ? 1 1 - - 0 1 1 1 0 0 0 1 0 0 1 0 0 1 1 2 0 0 0 0 1 0 1 0 1 0 2 0 1 1 1 1 0 1 1 0 0 0 0 1 0 0 0 0 1 0 0 1 0 1 1 1 1 2 1 1 1 0 0 0 1 0 0

101-Gregory. Fish skulls, Tree #1 2 1 2 0 0 1 0 0 1 0 1 - - - - - - - - - - 0 0 1 0 0 1 1 1 0 0 0 0 0 0 0 1 0 1 1 0 0 0 1 1 1 0 - - - - - 1 0 0 0 0 0 1 1 0 2 0 0 0 0 0 0 0 0 0 1 1 1 1 1 - - 0 0 1 1 0 0 ? 0 0 0 1 0 0 0 0 1 0 1 ? 0 1 0 0 0 0 0 2 0 0 ? 0 1 ? 0 1 0 0 0 0 1 1 0 1 0 0 1 0 1 1 1 1 1 0 2 1 1 1 0 0 - - 0 0

102-Gregory. Fish skulls, Tree #2 2 1 2 0 0 0 0 0 0 0 0 - - - - - - - - - - 0 0 1 0 0 0 0 1 0 0 0 1 0 0 0 1 0 0 1 0 0 0 0 0 1 0 - - - - - 1 0 0 0 0 0 1 1 0 1 0 0 0 0 0 0 0 0 0 1 1 1 1 1 - - 0 0 1 1 0 0 0 0 0 0 1 0 0 0 0 1 0 1 0 0 0 0 0 0 0 0 2 0 ? ? 0 1 ? 0 1 0 0 0 0 1 1 0 1 0 0 1 0 1 0 1 1 1 0 2 1 1 1 0 0 - - 0 0

103-Gregory. Fish skulls, Tree #3 2 0 2 0 0 0 0 0 1 0 0 0 0 0 0 0 1 1 1 1 1 0 0 1 0 1 0 0 1 0 0 1 0 0 0 0 1 0 1 1 0 0 0 0 0 0 1 - - - - - 0 1 0 0 0 0 1 1 0 2 0 0 0 0 0 0 0 0 0 1 1 1 1 0 2 0 0 0 1 1 0 0 0 0 0 0 1 0 0 1 0 1 0 1 0 0 1 0 0 0 0 0 2 0 0 ? 0 1 ? 0 1 0 0 0 0 0 0 0 1 0 0 1 0 1 0 0 1 1 0 2 1 1 1 0 0 - - 0 0

104-Gregory. Fish skulls, Tree #4 2 ? 2 0 0 0 0 0 1 0 0 - - - - - - - - - - 0 0 1 0 1 0 0 1 0 0 1 0 0 0 0 1 0 1 1 0 0 0 0 0 0 0 1 0 0 0 2 0 1 0 0 0 0 1 1 0 1 2 0 1 0 0 0 0 0 0 1 1 ? 1 0 2 0 0 0 1 1 0 0 0 0 0 0 1 0 0 1 0 1 0 1 0 0 1 0 0 0 0 0 2 0 0 ? 0 1 ? 0 1 0 0 0 0 0 0 0 1 0 0 1 0 1 0 0 1 1 0 2 1 1 1 0 0 - - 0 0

105-Schaffner. Phylogenetic taxonomy of plants, Tree #1 2 0 2 0 0 0 0 0 1 0 1 0 1 0 0 0 0 0 0 1 1 0 0 1 0 ? 0 1 0 0 0 1 0 0 0 0 1 0 1 1 0 0 0 0 0 0 0 - - - - - 0 1 0 0 0 0 1 1 0 2 0 0 0 0 0 0 0 0 0 1 1 ? 0 0 - - 0 0 1 0 0 0 0 0 0 0 0 0 0 0 0 1 0 1 0 0 1 0 0 0 0 0 1 0 0 ? 0 1 0 0 1 0 0 0 - 0 0 0 0 0 0 1 0 0 0 0 1 1 0 2 ? 1 1 0 0 - - 0 0

106-Schaffner. Phylogenetic taxonomy of plants, Tree #2 0 0 2 0 0 0 0 0 1 0 1 - - - - - - - - - - 1 0 1 1 1 0 1 0 0 0 1 0 0 0 0 1 0 1 1 0 0 0 0 1 0 0 0 1 0 0 2 0 1 0 0 0 0 0 0 0 2 0 0 1 0 0 0 0 0 0 1 1 ? 0 0 - - 0 0 1 0 0 0 0 0 0 0 1 0 0 0 0 1 0 1 0 0 1 0 0 0 0 0 2 0 0 ? 0 1 0 0 1 0 0 0 - 0 0 0 1 0 0 1 0 0 0 0 1 1 0 2 ? 1 1 0 0 - - 0 0

107-Copeland. The kingdom of organisms 2 0 2 0 0 0 0 0 1 0 1 - - - - - - - - - - 0 0 1 0 1 1 1 1 0 0 1 0 0 0 0 1 1 1 1 0 1 0 1 1 2 1 1 0 1 0 0 1 1 0 0 0 0 1 1 0 1 2 0 0 0 0 0 0 1 0 1 0 0 0 0 2 0 0 0 1 1 ? 0 0 0 0 1 1 0 0 1 0 1 0 1 0 0 1 0 0 0 0 0 2 ? 0 1 0 1 1 1 1 0 0 0 0 0 0 0 0 0 0 1 0 0 1 ? ? 1 1 2 1 1 1 0 0 - - 0 0

108-Cuenot. Un essai d-arbre genealogique du regne animal 2 0 2 0 0 0 0 0 0 0 2 0 0 0 0 0 0 0 0 0 1 0 0 1 1 1 1 2 1 0 0 1 0 0 0 0 1 0 1 1 0 0 0 1 1 1 0 - - - - - 0 1 0 1 0 0 1 1 0 2 0 0 0 1 0 0 0 1 0 1 1 0 1 0 0 0 0 0 1 0 0 0 0 0 0 0 0 0 0 0 0 1 2 0 0 0 0 0 1 0 0 0 0 0 0 0 0 1 0 0 1 0 1 0 1 0 0 0 0 0 0 1 0 1 1 1 1 1 0 2 1 1 1 0 0 - - 0 0

109-Stirton. Phylogeny of north-american equidae 2 0 2 0 0 0 0 0 1 0 1 - - - - - - - - - - 0 0 1 0 1 0 1 1 0 0 1 0 0 0 0 1 0 1 1 0 0 0 0 1 1 0 1 1 0 0 2 1 1 0 0 0 0 1 1 0 1 2 0 0 0 0 0 0 0 0 1 0 0 0 0 - - 0 0 1 1 0 0 0 0 0 0 1 0 0 0 0 1 0 1 0 0 1 0 0 0 0 0 2 0 0 1 1 1 0 1 1 0 0 0 0 1 0 0 1 0 0 1 0 0 0 1 1 1 0 2 1 1 1 0 0 - - 0 0

110-Michener. Comparative external morphology (…) of the b 2 0 2 0 0 0 0 0 1 0 0 1 1 0 0 0 1 0 1 1 1 0 0 1 0 1 0 0 1 0 0 1 0 0 0 0 1 1 1 2 0 0 0 0 0 0 ? - - - - - 0 1 0 0 0 0 1 1 0 1 0 0 ? 0 0 0 0 0 0 1 1 0 0 0 - - 0 0 1 0 0 0 0 0 0 0 1 0 0 0 0 1 0 1 0 0 1 0 0 0 0 0 2 0 0 ? 0 1 ? 0 1 0 0 0 0 1 0 0 1 0 0 1 0 0 0 0 1 1 0 2 ? 1 1 0 0 - - 0 1

111-Delacour & mayr. The family anatidae 0 2 1 0 1 0 0 1 1 0 0 1 1 1 0 0 1 0 1 0 1 1 0 1 1 1 0 0 0 0 0 1 0 0 1 0 1 1 1 2 0 1 0 0 0 2 1 - - - - - 1 1 0 0 1 0 0 0 0 2 0 0 0 0 0 0 0 0 0 1 ? 0 0 0 - - ? 0 1 1 0 0 0 0 0 ? 1 0 0 0 1 0 0 1 0 0 1 0 0 0 0 0 2 0 0 ? 0 1 ? 1 1 1 0 0 0 0 0 0 1 0 0 1 0 0 0 1 1 1 1 2 ? 1 1 0 0 - - 0 1

112-Gregory. Evolution emerging, Tree #1 2 0 2 0 0 0 0 1 1 0 0 0 1 0 0 0 1 1 0 0 0 1 0 1 0 1 0 1 1 0 0 1 0 0 0 0 1 0 1 1 0 0 0 0 0 1 0 - - - - - 0 0 0 0 0 0 1 1 0 2 0 0 0 0 0 0 0 0 0 1 ? 1 1 0 - - 0 0 1 ? 0 0 0 0 0 0 1 0 0 0 0 1 0 0 0 0 1 0 0 0 0 0 2 0 0 - 0 1 1 ? 1 0 0 0 0 0 0 0 1 0 0 1 0 1 1 1 1 1 0 2 1 1 1 0 0 - - 0 0

113-Gregory. Evolution emerging, Tree #2 2 0 2 0 0 0 0 0 0 0 0 - - - - - - - - - - 0 0 1 0 1 0 0 1 0 0 1 0 0 0 0 1 0 ? 1 0 0 0 0 0 1 0 - - - - - 1 1 0 0 0 0 1 1 0 1 2 0 1 0 0 0 0 0 0 1 0 1 1 0 0 0 0 0 1 0 0 0 0 ? 0 0 0 0 0 0 0 1 0 0 0 0 1 0 0 0 0 0 2 0 0 0 0 1 1 0 1 0 0 0 0 0 0 0 1 0 0 1 0 1 0 1 1 1 0 2 1 1 1 0 0 - - 0 0

114-Gregory. Evolution emerging, Tree #3 2 2 2 0 0 0 0 0 1 0 1 0 1 0 0 0 1 0 1 0 0 0 0 1 0 1 0 0 1 0 0 0 1 0 0 0 1 0 ? 1 0 0 0 0 0 1 0 - - - - - 0 1 0 0 0 0 1 1 0 2 0 0 0 0 0 0 0 0 0 1 1 1 1 0 - - 0 0 1 ? 0 0 0 0 0 0 1 0 0 0 0 1 0 0 0 0 0 0 0 0 0 0 2 0 0 1 0 1 1 0 1 0 0 0 0 0 0 0 1 0 0 1 0 1 0 1 1 1 0 2 1 1 1 0 0 - - 0 0

115-Gregory. Evolution emerging, Tree #4 2 0 2 0 0 0 0 0 1 0 1 - - - - - - - - - - 0 0 1 0 1 0 0 1 0 0 0 1 0 0 0 1 0 ? 1 0 0 0 0 0 1 0 - - - - - 0 1 0 0 0 0 1 1 0 2 0 0 0 0 0 0 0 0 0 1 1 1 1 0 - - 0 0 1 ? 0 0 0 0 0 0 1 0 0 0 0 1 0 0 0 0 1 0 0 0 0 0 2 0 0 1 0 1 1 0 1 0 0 0 0 0 0 0 1 0 0 1 0 1 0 1 1 1 0 2 1 1 1 0 0 - - 0 0

116-Gregory. Evolution emerging, Tree #5 2 2 2 0 0 1 0 0 1 0 1 - - - - - - - - - - 0 0 1 0 1 0 1 1 0 0 0 0 0 0 0 1 0 ? 1 0 0 0 0 1 1 0 - - - - - 0 1 1 0 0 0 1 1 0 2 0 0 0 1 0 1 0 0 0 1 1 ? ? 0 - - 0 0 1 0 0 0 0 ? 0 0 1 0 0 0 0 1 0 0 0 0 1 0 0 0 0 0 2 0 0 0 0 1 1 0 1 0 0 0 0 0 0 0 1 0 0 1 0 1 0 0 0 1 0 2 1 1 1 0 0 - - 0 0

117-Gregory. Evolution emerging, Tree #6 2 2 2 0 0 1 0 0 1 0 1 - - - - - - - - - - 0 0 1 0 1 0 1 1 0 0 0 0 0 0 0 1 0 ? 1 0 0 0 0 1 1 0 - - - - - 0 1 1 0 0 0 1 1 0 2 0 0 0 1 0 1 0 0 0 1 1 ? ? 0 - - 0 0 1 0 0 0 0 ? 0 0 1 0 0 0 0 1 0 0 0 0 1 0 0 0 0 0 2 0 0 0 0 1 1 0 1 0 0 0 0 0 0 0 1 0 0 1 0 1 0 0 0 1 0 2 1 1 1 0 0 - - 0 0

118-Gregory. Evolution emerging, Tree #7 2 0 2 0 0 0 0 0 1 0 0 - - - - - - - - - - 0 0 1 0 1 0 0 1 0 0 0 0 0 0 0 1 0 ? 1 0 0 0 0 0 1 0 - - - - - 0 1 0 0 0 0 1 1 0 2 0 0 0 0 0 1 0 0 0 1 1 1 1 0 - - 0 0 1 ? 0 0 0 0 0 0 1 0 0 0 0 1 0 0 0 0 1 0 0 0 0 0 2 0 0 1 0 1 1 0 1 0 0 0 0 0 0 0 1 0 0 1 0 1 0 1 1 1 0 2 1 1 1 0 0 - - 0 0

119-Gregory. Evolution emerging, Tree #8 2 0 2 0 0 0 0 0 1 0 0 - - - - - - - - - - 0 0 1 0 1 0 0 1 0 0 0 0 0 0 0 1 0 ? 1 0 0 0 0 0 1 0 - - - - - 0 1 0 0 0 0 1 1 0 2 0 0 0 0 0 1 0 0 0 1 1 1 1 0 - - 0 0 1 ? 0 0 0 0 0 0 1 0 0 0 0 1 0 0 0 0 1 0 0 0 0 0 2 0 0 1 0 1 1 0 1 0 0 0 0 0 0 0 1 0 0 1 0 1 0 1 1 1 0 2 1 1 1 0 0 - - 0 0

120-Gregory. Evolution emerging, Tree #9 2 0 2 0 0 0 0 0 1 0 0 - - - - - - - - - - 0 0 1 0 1 0 0 1 0 0 0 0 0 0 0 1 0 0 1 0 0 0 0 0 1 0 - - - - - 0 1 0 0 0 0 1 1 0 2 0 0 0 0 0 0 0 0 0 1 1 1 1 0 - - 0 0 1 ? 0 0 0 0 0 0 1 0 0 0 0 1 0 0 0 0 1 0 0 0 0 0 2 0 0 1 0 1 1 0 1 0 0 0 0 0 0 0 1 0 0 1 0 1 0 1 1 1 0 2 1 1 1 0 0 - - 0 0

121-Gregory. Evolution emerging, Tree #10 2 0 2 0 0 0 0 0 1 0 0 - - - - - - - - - - 0 0 1 0 1 0 0 1 0 0 0 0 0 0 0 1 0 ? 1 0 0 0 0 0 1 0 - - - - - 0 1 0 0 0 0 1 1 0 2 0 0 0 0 0 0 0 0 0 1 1 1 1 0 - - 0 0 1 ? 0 0 0 0 0 0 1 0 0 0 0 1 0 0 0 0 1 0 0 0 0 0 2 0 0 1 0 1 1 0 1 0 0 0 0 0 0 0 1 0 0 1 0 1 0 1 1 1 0 2 1 1 1 0 0 - - 0 0

122-Gregory. Evolution emerging, Tree #11 2 0 2 0 0 0 0 0 1 0 0 - - - - - - - - - - 0 0 1 0 1 0 0 1 0 0 0 1 0 0 0 1 0 0 1 0 0 0 0 0 1 0 - - - - - 0 1 0 0 0 0 1 1 0 2 0 0 0 0 0 0 0 0 0 1 1 1 1 0 - - 0 0 1 ? 0 0 0 0 0 0 1 0 0 0 0 1 0 0 0 0 1 0 0 0 0 0 2 0 0 1 0 1 1 0 1 0 0 0 0 0 0 0 1 0 0 1 0 1 0 1 1 1 0 2 1 1 1 0 0 - - 0 0

123-Gregory. Evolution emerging, Tree #12 2 0 2 0 0 1 0 0 1 0 0 - - - - - - - - - - 0 0 1 0 1 0 0 1 0 0 0 0 0 0 0 1 0 ? 1 0 0 0 0 0 1 0 - - - - - 0 1 0 0 0 0 1 1 0 2 0 0 0 0 0 0 0 0 0 1 1 1 1 0 - - 0 0 1 ? 0 0 0 0 0 0 1 0 0 0 0 1 0 0 0 0 1 0 0 0 0 0 2 0 0 1 0 1 1 0 1 0 0 0 0 0 0 0 1 0 0 1 0 1 0 1 1 1 0 2 1 1 1 0 0 - - 0 0

124-Gregory. Evolution emerging, Tree #13 0 0 2 0 0 0 0 0 1 0 0 - - - - - - - - - - 0 0 1 0 1 0 0 1 0 0 0 0 0 0 0 1 0 ? 1 0 0 0 0 0 1 0 1 0 0 0 2 ? 1 0 0 0 1 1 1 0 1 2 0 1 0 0 1 0 0 0 1 1 1 1 0 - - 0 0 1 ? 0 0 0 0 0 0 1 0 0 0 0 1 0 0 0 0 1 0 0 0 0 0 2 0 0 1 0 1 1 0 1 0 0 0 0 0 0 0 1 0 0 1 0 1 0 1 1 1 0 2 1 1 1 0 0 - - 0 0

125-Gregory. Evolution emerging, Tree #14 2 0 2 0 0 1 0 0 1 0 0 - - - - - - - - - - 0 0 1 0 1 0 0 1 0 0 0 0 0 0 0 1 0 0 1 0 0 0 0 0 1 0 - - - - - 0 1 0 0 0 0 1 1 0 2 0 0 0 0 0 0 0 0 0 1 1 1 1 0 - - 0 0 1 ? 0 0 0 0 0 0 1 0 0 0 0 1 0 0 0 0 1 0 0 0 0 0 2 0 0 1 0 1 0 0 1 0 0 0 0 0 0 0 1 0 0 1 0 1 0 1 1 1 0 2 1 1 1 0 0 - - 0 0

126-Gregory. Evolution emerging, Tree #15 2 0 2 0 0 1 0 0 1 0 0 0 1 0 0 0 1 0 1 0 0 0 0 1 0 1 0 0 1 0 0 0 0 0 0 0 1 0 ? 1 0 0 0 0 0 1 0 - - - - - 0 1 0 0 0 0 1 1 0 2 0 0 0 0 0 0 0 0 0 1 1 1 1 0 - - 0 0 1 ? 0 0 0 0 0 0 1 0 0 0 0 1 0 0 0 0 1 0 0 0 0 0 2 0 0 1 0 1 1 0 1 0 0 0 0 0 0 0 1 0 0 1 0 1 0 1 1 1 0 2 1 1 1 0 0 - - 0 0

127-Gregory. Evolution emerging, Tree #16 2 0 2 0 0 0 0 0 1 0 0 - - - - - - - - - - 0 0 1 0 1 0 0 1 0 0 0 0 0 0 0 1 0 0 1 0 0 0 0 0 1 0 - - - - - 0 1 0 0 0 0 1 1 0 1 2 0 1 0 0 0 0 0 0 1 1 1 1 0 - - 0 0 1 ? 0 0 0 0 0 0 1 0 0 0 0 1 0 0 0 0 1 0 0 0 0 0 2 0 0 1 0 1 0 0 1 0 0 0 0 1 0 0 1 0 0 1 0 1 0 1 1 1 0 2 1 1 1 0 0 - - 0 0

128-Gregory. Evolution emerging, Tree #17 2 0 2 0 0 0 0 0 1 0 0 - - - - - - - - - - 0 0 1 0 1 0 0 0 0 0 0 0 0 0 0 1 0 ? 1 0 0 0 0 0 1 0 - - - - - 0 1 0 0 0 0 1 1 0 1 2 0 1 1 0 0 0 0 0 1 1 1 1 0 0 0 0 0 1 0 0 0 0 0 0 0 0 0 0 0 0 1 0 0 0 0 1 0 0 0 0 0 2 0 0 1 0 1 0 0 1 0 0 0 0 1 0 0 1 0 0 1 0 1 0 1 1 1 0 2 1 1 1 0 0 - - 0 0

129-Gregory. Evolution emerging, Tree #18 2 0 2 0 0 0 0 ? 1 0 0 - - - - - - - - - - 0 0 1 0 1 0 0 0 1 0 0 0 0 0 0 1 0 ? 1 0 0 0 0 0 1 0 - - - - - 1 1 0 0 0 0 1 1 0 1 2 1 1 1 1 0 0 0 1 1 1 1 1 0 - - 0 0 1 0 0 0 0 0 0 0 0 0 0 0 0 1 2 0 0 0 0 0 0 0 0 0 2 0 0 1 0 1 0 0 1 0 1 0 0 1 0 0 1 0 0 1 0 1 0 1 1 1 0 2 1 1 1 0 0 - - 0 0

130-Gregory. Evolution emerging, Tree #19 2 2 2 0 0 0 0 0 1 0 0 0 1 0 0 0 1 1 1 0 0 ? 0 1 ? 1 0 0 1 0 0 0 0 0 0 0 1 0 ? 1 0 0 0 0 0 1 0 - - - - - 0 1 0 0 0 0 1 1 0 2 0 0 0 1 0 0 0 0 0 1 1 1 1 0 - - 0 0 1 ? 0 0 0 0 0 0 1 0 0 0 0 1 0 0 0 0 1 0 0 0 0 0 2 0 0 1 0 1 1 0 1 0 0 0 0 0 0 0 1 0 0 1 0 1 0 1 1 1 0 2 1 1 1 0 0 - - 0 0

131-Gregory. Evolution emerging, Tree #20 2 2 2 0 0 1 0 0 1 0 0 0 1 0 0 0 1 1 1 0 0 ? 0 1 ? 1 0 0 1 0 0 0 0 0 0 0 1 0 ? 1 0 0 0 0 0 1 0 - - - - - 0 1 0 0 0 0 1 1 0 2 0 0 0 0 0 1 0 0 0 1 1 1 1 0 - - 0 0 1 ? 0 0 0 0 0 0 1 0 0 0 0 1 0 0 0 0 1 0 0 0 0 0 2 0 0 1 0 1 1 0 1 0 0 0 0 0 0 0 1 0 0 1 0 1 0 1 1 1 0 2 1 1 1 0 0 - - 0 0

132-Gregory. Evolution emerging, Tree #21 2 2 2 0 0 0 0 ? 1 0 0 - - - - - - - - - - 0 0 1 0 0 0 0 0 1 0 0 0 0 0 0 1 0 0 1 0 0 0 0 0 1 0 - - - - - 1 1 0 0 0 0 1 1 0 2 0 1 1 0 1 0 0 0 0 1 1 1 1 0 - - 0 0 1 0 0 0 0 0 0 0 1 0 0 0 0 1 2 0 0 0 0 0 0 0 0 0 2 0 0 1 0 1 0 0 1 0 1 0 0 1 0 0 1 0 0 1 0 1 0 1 1 1 0 2 1 1 1 0 0 - - 0 0

133-Gregory. Evolution emerging, Tree #22 2 0 2 0 0 0 0 ? 1 0 0 0 1 0 0 0 1 0 0 0 0 ? 0 1 0 0 0 1 0 1 0 0 0 0 0 0 1 0 ? 1 0 0 0 0 1 1 0 - - - - - 1 1 0 0 0 0 1 1 0 2 0 1 1 0 1 0 0 0 0 1 1 1 1 0 - - 0 0 1 0 0 0 0 0 0 0 1 0 0 0 0 1 2 0 0 0 0 0 0 0 0 0 2 0 0 1 0 1 0 0 1 0 1 0 0 1 0 0 1 0 0 1 0 1 0 1 1 1 0 2 1 1 1 0 0 - - 0 0

134-Gregory. Evolution emerging, Tree #23 2 0 2 0 0 0 1 0 1 0 0 - - - - - - - - - - 0 0 1 0 1 0 1 1 0 0 0 0 0 0 0 1 0 ? 1 0 0 0 0 1 1 0 1 1 0 0 0 0 1 0 0 0 0 1 1 0 1 2 0 0 1 1 0 0 0 0 1 1 1 1 0 - - 0 0 1 0 0 0 0 0 0 0 1 0 0 0 0 1 0 0 0 0 1 0 0 0 0 0 2 0 0 0 0 1 ? 0 1 0 1 0 0 0 0 0 1 0 0 1 0 1 0 1 1 1 0 2 1 1 1 0 0 - - 0 0

135-Gregory. Evolution emerging, Tree #24 2 0 2 0 0 0 0 0 1 0 0 - - - - - - - - - - 0 0 1 0 1 0 0 0 0 0 0 0 0 0 0 1 0 ? 1 0 0 0 0 0 1 0 - - - - - 1 1 0 0 0 0 1 1 0 2 0 0 1 0 0 0 0 0 0 1 1 1 1 0 - - 0 0 1 0 0 0 0 0 0 0 1 0 0 0 0 1 0 0 0 0 1 0 0 0 0 0 2 0 0 1 0 1 0 0 1 0 0 0 0 1 0 0 1 0 0 1 0 1 0 1 1 1 0 2 1 1 1 0 0 - - 0 0

136-Gregory. Evolution emerging, Tree #25 ? 0 2 0 0 0 0 ? 1 0 1 - - - - - - - - - - 0 0 1 ? 0 0 2 1 0 0 1 0 0 0 0 1 0 ? 1 0 0 0 0 1 1 0 - - - - - 1 1 0 0 1 1 1 1 0 1 0 0 0 1 0 1 0 0 0 1 1 ? 1 0 - - 0 0 1 ? 0 0 0 0 0 0 1 0 0 0 0 1 0 0 0 0 1 0 0 0 0 0 2 0 0 0 ? 1 ? ? 1 0 0 0 0 0 0 0 1 0 0 1 0 1 0 1 1 1 0 2 1 1 1 0 0 - - 0 0

137-Gregory. Evolution emerging, Tree #26 2 0 2 0 0 0 0 0 1 0 0 - - - - - - - - - - 0 0 1 0 1 0 0 1 0 0 0 0 0 0 0 1 0 ? 1 0 0 0 0 0 1 0 - - - - - 0 1 0 0 0 0 1 1 0 2 0 0 0 0 0 0 0 0 0 1 1 1 1 0 - - 0 0 1 ? 0 0 0 0 0 0 1 0 0 0 0 1 0 0 0 0 1 0 0 0 0 0 2 0 0 1 0 1 1 0 1 0 0 0 0 0 0 0 1 0 0 1 0 1 0 1 1 1 0 2 1 1 1 0 0 - - 0 0

138-Gregory. Evolution emerging, Tree #27 2 2 2 0 0 1 0 0 1 0 0 - - - - - - - - - - 0 0 1 ? 1 0 0 0 0 0 0 0 0 0 0 1 0 ? 1 0 0 0 0 0 1 0 - - - - - 1 1 0 0 0 0 1 1 0 2 0 0 1 0 0 0 0 0 0 1 1 1 1 0 - - 0 0 1 0 0 0 0 0 0 0 1 0 0 0 0 1 0 0 0 0 1 0 0 0 0 0 2 0 0 1 0 1 0 0 1 0 0 0 0 1 0 0 1 0 0 1 0 1 0 1 1 1 0 2 1 1 1 0 0 - - 0 0

139-Gregory. Evolution emerging, Tree #28 2 2 2 0 0 0 0 0 1 0 0 - - - - - - - - - - 0 0 1 0 1 0 0 0 0 0 0 0 0 0 0 1 0 ? 1 0 0 0 0 0 1 0 - - - - - 1 1 0 0 0 0 1 1 0 2 0 0 1 1 0 0 0 0 0 1 1 1 1 0 - - 0 0 1 0 0 0 0 0 0 0 1 0 0 0 0 1 0 0 0 0 1 0 0 0 0 0 2 0 0 1 0 1 0 0 1 0 0 0 0 1 0 0 1 0 0 1 0 1 0 1 1 1 0 2 1 1 1 0 0 - - 0 0

140-Gregory. Evolution emerging, Tree #29 2 0 2 0 0 1 0 0 1 0 0 - - - - - - - - - - 0 0 1 0 1 0 0 0 0 0 0 0 0 0 0 1 0 ? 1 0 0 0 0 0 1 0 - - - - - 1 1 0 0 0 0 1 1 0 2 0 0 1 1 0 0 0 0 0 1 1 1 1 0 - - 0 0 1 0 0 0 0 0 0 0 1 0 0 0 0 1 0 0 0 0 1 0 0 0 0 0 2 0 0 1 0 1 0 0 1 0 0 0 0 1 0 0 1 0 0 1 0 1 0 1 1 1 0 2 1 1 1 0 0 - - 0 0

141-Gregory. Evolution emerging, Tree #30 2 0 2 0 0 0 0 0 1 0 0 - - - - - - - - - - 0 0 1 0 1 0 0 1 0 1 0 0 0 0 0 1 0 0 1 0 0 0 1 0 C 0 0 1 0 1 2 0 1 0 1 0 0 1 1 0 2 0 0 1 1 0 0 0 0 0 1 1 1 1 0 0 0 0 0 1 0 0 0 0 0 0 0 1 0 0 0 0 1 0 0 0 0 1 0 0 0 0 0 2 0 0 0 0 1 ? 0 1 0 1 0 0 1 0 0 1 0 0 1 0 1 0 1 1 1 0 2 1 1 1 0 0 - - 0 0

142-Gregory. Evolution emerging, Cover 2 2 2 0 0 1 0 ? 1 0 0 - - - - - - - - - - 0 0 1 0 1 0 0 1 0 0 1 0 0 0 0 1 0 1 1 0 0 0 0 1 1 0 1 1 0 0 0 0 1 0 0 0 0 1 1 0 1 2 0 1 0 0 0 0 0 0 1 1 ? 1 0 0 0 0 0 1 0 0 0 0 0 0 0 0 0 0 0 0 1 0 0 0 0 1 0 0 0 0 0 2 0 0 ? 0 1 1 0 1 0 1 0 0 0 0 0 1 0 0 1 0 1 1 1 1 1 0 2 1 1 1 0 0 - - 0 0

143-Teillard de Chardin. Le phenomene humain, Tree #1 2 2 2 0 0 0 0 0 1 0 0 - - - - - - - - - - 0 1 1 0 1 0 0 1 0 1 1 0 1 0 0 1 0 1 1 0 0 1 0 0 1 0 - - - - - 0 1 0 0 0 0 1 1 0 1 2 0 1 0 0 0 0 0 0 1 1 0 1 0 0 0 0 0 1 0 0 0 0 0 0 0 0 0 0 0 0 1 1 0 0 0 1 1 0 1 1 0 0 0 0 ? 0 1 0 0 1 0 0 0 1 0 0 0 1 0 1 0 0 1 0 1 1 1 0 2 1 0 0 0 0 - - 0 0

144-Teillard de Chardin. Le phenomene humain, Tree #2 2 2 2 0 0 1 0 0 1 0 0 - - - - - - - - - - 0 1 1 0 1 0 0 1 0 1 1 0 1 0 0 1 0 1 1 0 0 1 0 0 1 0 - - - - - 0 1 0 0 0 0 1 1 0 1 2 0 1 0 0 0 0 0 0 1 1 0 1 0 0 0 0 0 1 0 0 0 0 0 0 0 1 0 0 0 0 1 0 0 0 0 1 1 0 1 1 0 0 0 0 ? 0 1 0 0 1 0 0 0 1 0 0 0 1 0 1 0 0 1 0 1 1 1 0 2 1 0 0 0 0 - - 0 0

145-Teillard de Chardin. Le phenomene humain, Tree #3 2 2 2 0 0 1 0 0 1 0 0 - - - - - - - - - - 0 1 1 0 1 0 0 1 0 1 1 0 1 0 0 1 0 1 1 0 0 1 0 0 1 0 - - - - - 0 1 0 0 0 0 1 1 0 1 2 0 1 0 0 0 0 0 0 1 1 0 1 0 0 0 0 0 1 0 0 0 0 0 0 0 1 0 0 0 0 1 0 0 0 0 1 1 0 1 1 0 0 0 0 ? 0 1 0 0 1 0 0 0 1 0 0 0 1 0 1 0 0 1 0 1 1 1 0 2 1 0 0 0 0 - - 0 0

146-Teillard de Chardin. Le groupe zoologique humain 2 0 2 1 0 1 0 1 1 0 0 0 0 0 0 0 1 0 1 1 1 0 1 1 0 1 0 0 1 0 1 1 0 1 0 0 1 0 1 1 0 0 1 0 0 1 0 - - - - - 0 1 0 0 0 0 1 1 0 1 0 0 0 0 0 0 0 0 0 1 1 0 1 0 1 0 0 0 1 0 0 0 0 0 0 0 0 0 0 0 0 1 0 0 0 0 1 1 0 1 0 0 0 ? 0 ? 0 0 0 0 1 0 0 0 1 0 0 0 0 0 0 0 0 1 1 1 1 1 0 2 1 0 0 0 0 - - 0 0

147-Simpson. Principles of animal taxonomy, Tree #1 0 2 1 0 0 0 1 ? 1 1 0 - - - - - - - - - - 1 0 1 ? 1 0 0 1 0 0 0 0 0 0 0 1 0 1 1 0 1 0 0 0 1 ? 0 1 0 0 2 1 1 0 0 1 0 1 1 0 1 0 0 0 0 0 0 0 0 0 1 0 ? ? ? 2 0 0 0 0 1 0 0 0 1 ? 1 1 0 0 1 0 1 2 1 ? 0 1 0 0 0 0 - 2 0 1 1 1 1 0 1 1 0 0 0 0 ? ? 0 0 0 1 0 0 0 0 1 1 1 0 2 1 1 1 0 0 - - 0 0

148-Simpson. Principles of animal taxonomy, Tree #2 0 2 1 0 0 0 1 ? 1 1 0 - - - - - - - - - - 1 0 1 ? 1 0 0 1 0 0 0 0 0 0 0 1 0 1 1 0 1 0 0 0 1 ? 0 1 0 0 2 1 1 0 0 1 0 1 1 0 1 0 0 0 0 0 0 0 0 0 1 0 ? ? ? 2 0 0 0 0 1 0 0 0 1 ? 1 1 0 0 1 0 1 2 1 ? 0 1 0 0 0 0 - 2 0 1 1 1 1 0 1 1 0 0 0 0 ? ? 0 0 0 1 0 0 0 0 1 1 1 0 2 1 1 1 0 0 - - 0 0

149-Simpson. Principles of animal taxonomy, Tree #3 0 2 1 0 0 0 0 ? 1 0 0 - - - - - - - - - - 1 0 1 1 1 0 0 1 0 0 1 1 0 0 0 1 ? 1 ? 0 0 0 0 0 1 ? - - - - - 1 2 1 0 1 1 1 1 0 1 0 0 0 0 0 0 0 0 0 0 0 ? ? ? 2 0 0 1 1 1 0 0 1 1 1 1 1 0 0 1 1 2 0 1 0 0 1 0 0 0 0 - 2 1 1 1 1 1 1 1 1 0 0 0 0 ? ? 0 0 0 1 0 0 0 0 1 1 1 1 2 1 1 1 0 0 - - 0 0

150-Simpson. Principles of animal taxonomy, Tree #4 0 2 1 0 0 0 0 ? 1 0 0 - - - - - - - - - - 1 0 1 1 1 0 0 1 0 0 1 0 0 0 0 1 ? 1 ? 0 0 0 0 0 1 ? - - - - - 1 1 0 0 1 0 1 1 0 1 0 0 0 0 0 0 0 0 0 1 0 ? ? ? 2 0 0 0 1 1 0 0 1 1 1 1 1 0 0 0 1 1 0 1 0 0 1 0 0 0 0 - 2 0 1 1 1 1 1 1 1 0 0 0 0 ? ? 0 0 0 1 0 0 0 0 1 1 1 0 2 1 1 1 0 0 - - 0 0

151-Simpson. Principles of animal taxonomy, Tree #5 0 2 1 0 0 0 0 ? 1 0 0 - - - - - - - - - - 1 0 1 1 1 0 0 1 0 0 1 0 0 0 0 1 ? 1 ? 0 0 0 0 0 1 ? 1 1 0 0 2 1 1 0 0 1 0 1 1 0 1 0 0 0 0 0 0 0 0 0 1 0 ? ? ? 2 0 0 0 1 1 0 0 1 1 1 1 1 0 0 0 1 1 0 1 0 0 1 0 0 0 0 - 2 0 1 1 1 1 1 1 1 0 0 0 0 ? ? 0 0 0 1 0 0 0 0 1 1 1 0 2 1 1 1 0 0 - - 0 0

152-Simpson. Principles of animal taxonomy, Tree #6 0 2 1 0 0 0 0 ? 1 0 0 - - - - - - - - - - 1 0 1 1 1 0 0 1 0 0 1 0 0 0 0 1 ? 1 ? 0 0 0 0 0 1 ? - - - - - 1 2 1 0 1 1 1 1 0 1 0 0 0 1 0 0 0 0 0 0 0 ? ? ? 2 0 0 1 1 1 0 0 1 1 1 1 1 0 0 0 1 2 2 1 0 0 1 0 0 0 0 - 2 0 1 1 1 1 1 1 1 0 0 0 0 ? ? 0 0 0 1 0 0 0 0 1 1 1 0 2 1 1 1 0 0 - - 0 0

153-Simpson. Principles of animal taxonomy, Tree #7 0 2 1 0 0 0 0 ? 1 0 0 - - - - - - - - - - 1 0 1 1 1 0 0 1 0 0 1 0 0 0 0 1 ? 1 ? 0 0 0 0 0 1 ? 1 1 0 0 2 1 1 0 0 1 0 1 1 0 1 0 0 0 0 0 0 0 0 0 1 0 ? ? ? 2 0 0 0 1 1 0 0 1 1 1 1 1 0 0 0 1 1 0 1 0 0 1 0 0 0 0 - 2 0 1 1 1 1 1 1 1 0 0 0 0 ? ? 0 0 0 1 0 0 0 0 1 1 1 0 2 1 1 1 0 0 - - 0 0

154-Simpson. Principles of animal taxonomy, Tree #8 0 2 1 0 0 0 0 ? 1 0 0 - - - - - - - - - - 1 0 1 1 1 0 0 1 0 0 1 0 0 0 0 1 ? 1 ? 0 0 0 0 0 1 ? - - - - - 1 2 1 0 1 1 1 1 0 1 0 0 0 0 0 0 0 0 0 0 0 ? ? ? 2 0 0 1 1 1 0 0 1 1 1 1 1 0 0 0 1 2 0 1 0 0 1 0 0 0 0 - 2 0 1 1 1 1 1 1 1 0 0 0 0 ? ? 0 0 0 1 0 0 0 0 1 1 1 0 2 1 1 1 0 0 - - 0 0

155-Simpson. Principles of animal taxonomy, Tree #9 0 2 1 0 0 0 0 ? 1 0 0 - - - - - - - - - - 1 0 1 1 1 0 0 1 0 0 1 0 0 0 0 1 ? 1 ? 0 0 0 0 0 1 ? 0 1 0 1 2 1 1 0 1 1 0 1 1 0 1 0 0 0 1 0 0 0 0 0 1 0 ? ? ? 2 0 0 0 1 1 0 0 1 1 1 1 1 0 0 0 1 1 0 1 0 0 1 0 0 0 0 - 2 0 1 1 1 1 1 1 1 0 0 0 0 ? ? 0 0 0 1 0 0 0 0 1 1 1 0 2 1 1 1 0 0 - - 0 0

156-Simpson. Principles of animal taxonomy, Tree #10 0 2 1 0 0 0 0 ? 1 0 0 - - - - - - - - - - 1 0 1 1 1 0 0 1 0 0 1 0 0 0 0 1 ? 1 ? 0 0 0 0 0 1 ? 0 1 0 1 2 1 1 0 1 1 0 1 1 0 1 0 0 0 1 0 0 0 0 0 1 0 ? ? ? 2 0 0 0 1 1 0 0 1 1 1 1 1 0 0 0 1 1 0 1 0 0 1 0 0 0 0 - 2 0 1 1 1 1 1 1 1 0 0 0 0 ? ? 0 0 0 1 0 0 0 0 1 1 1 0 2 1 1 1 0 0 - - 0 0

157-Simpson. Principles of animal taxonomy, Tree #11 0 2 2 0 0 0 0 ? 1 0 0 - - - - - - - - - - 0 0 1 0 1 0 0 1 0 0 0 0 0 0 0 1 0 ? 1 0 0 0 0 0 1 0 0 1 0 0 2 1 1 0 0 1 0 1 1 0 1 2 0 1 1 0 0 0 0 0 1 0 ? 1 ? - - 0 0 1 1 0 0 1 1 1 1 1 0 0 0 0 1 0 1 0 0 1 0 0 0 0 0 2 0 1 1 1 1 1 1 1 0 1 0 0 0 0 0 1 0 0 0 0 0 0 1 1 1 0 2 1 1 1 0 0 - - 0 0

158-Simpson. Principles of animal taxonomy, Tree #12 0 0 2 0 0 0 0 ? 1 0 0 - - - - - - - - - - 0 0 1 0 1 0 0 1 0 0 1 0 0 0 0 1 0 1 1 0 0 0 0 0 1 0 0 1 0 0 2 1 1 0 0 0 0 1 1 0 1 2 0 1 1 0 0 0 0 0 1 0 ? 1 ? 1 0 0 0 1 1 0 0 1 1 1 1 1 0 0 0 0 1 0 1 0 0 1 0 0 0 0 0 2 0 1 1 0 1 1 1 1 0 1 0 0 0 0 0 1 0 0 0 0 0 0 1 1 1 0 2 1 1 1 0 0 - - 0 0

159-Simpson. Principles of animal taxonomy, Tree #13 2 2 2 0 0 0 0 0 1 0 0 - - - - - - - - - - 0 0 1 0 1 0 1 1 0 0 1 0 0 0 0 1 0 1 1 0 0 0 0 1 1 0 0 1 0 0 0 1 1 0 0 1 0 1 1 0 1 0 0 1 1 0 0 0 0 0 1 1 0 1 0 1 0 0 0 1 0 0 0 1 1 1 1 0 1 0 0 0 1 2 1 0 0 0 0 0 0 0 0 2 0 1 1 0 1 0 0 1 0 0 0 1 0 0 0 1 0 0 1 0 0 0 1 1 1 0 2 1 1 1 0 0 - - 0 0

160-Simpson. Principles of animal taxonomy, Tree #14 0 2 1 0 0 0 0 ? 1 0 0 - - - - - - - - - - 1 0 1 0 1 0 0 1 0 0 1 1 0 0 0 1 0 1 1 0 0 0 0 0 1 0 - - - - - 1 1 0 0 1 0 1 1 0 1 2 0 0 1 0 0 0 0 0 1 0 ? ? ? - - 0 0 1 1 0 0 1 1 1 1 1 0 0 0 1 1 0 1 0 0 1 0 0 0 0 0 2 0 1 1 1 1 1 1 1 0 0 0 0 0 0 0 1 0 0 0 0 0 0 1 1 1 0 2 1 1 1 0 0 - - 0 0

161-Sokal & Sneath, p. H. A. 1963. Principles of numerical ta 0 2 0 0 0 0 0 - - - 0 - - - - - - - - - - 1 0 0 - - 0 0 1 1 0 0 1 0 0 1 0 1 - 0 1 1 0 0 0 0 1 - - - - - 1 2 1 0 1 1 0 1 1 0 0 0 0 0 0 0 0 0 0 - - - - - 2 0 1 1 0 1 1 0 1 1 1 1 1 0 0 1 1 2 0 1 0 1 0 1 1 1 0 1 2 1 1 1 1 1 1 1 0 1 0 0 0 0 1 1 1 0 1 0 0 0 0 0 0 0 0 0 - 1 1 0 0 1 - 1 1

162-Sokal & Sneath, p. H. A. 1963. Principles of numerical ta 0 2 0 0 0 0 0 - - - 1 - - - - - - - - - - 1 0 0 - - 0 0 1 1 0 0 1 0 0 1 0 1 - 0 1 1 0 0 0 0 1 - - - - - 1 2 1 0 1 1 0 1 1 0 0 0 0 0 0 0 0 1 0 - - - - - 2 0 1 1 0 1 1 0 1 1 1 1 1 0 0 1 1 2 0 1 0 1 0 1 1 1 0 1 2 1 1 1 1 1 1 1 0 1 0 0 0 0 1 1 1 0 1 0 0 0 0 0 0 0 0 0 1 1 1 ? 0 1 - 1 1

163-Hennig. Phylogenetic systematics, Tree #1 0 2 1 0 1 0 0 1 1 1 0 - - - - - - - - - - 1 0 0 1 1 0 0 0 0 0 1 1 0 1 0 1 1 1 2 1 1 0 0 0 2 1 - - - - - 1 2 1 0 1 1 0 0 0 1 0 0 0 0 0 0 0 0 0 0 0 0 0 2 2 0 0 1 1 1 1 0 1 1 1 1 1 0 0 1 1 2 0 1 0 0 0 1 1 0 0 1 2 1 1 1 1 1 1 1 1 1 0 0 0 0 1 1 0 0 1 0 0 0 0 1 0 1 1 2 1 2 2 0 1 0 - 1 1

164-Hennig. Phylogenetic systematics, Tree #2 0 2 1 0 1 0 0 1 1 1 0 - - - - - - - - - - 1 0 0 1 1 0 0 0 0 0 1 1 0 1 0 1 1 1 2 1 1 0 0 0 2 1 - - - - - 1 2 1 0 1 1 0 0 0 1 0 0 0 0 0 0 0 0 0 0 0 0 0 2 2 0 0 1 1 1 1 0 1 1 1 1 1 1 0 1 1 2 0 1 0 0 0 1 1 0 0 1 2 1 1 ? 1 1 1 1 1 1 0 0 0 0 1 1 0 0 1 0 0 0 0 1 0 1 1 2 1 2 2 0 1 0 - 1 1

165-Hennig. Phylogenetic systematics, Tree #3 0 2 1 0 1 0 0 1 1 1 0 - - - - - - - - - - 1 0 0 1 1 0 0 0 0 0 1 1 0 1 0 1 1 1 2 1 1 0 0 0 2 1 - - - - - 1 2 1 0 1 1 0 0 0 1 0 0 0 0 0 0 0 0 0 0 0 0 0 2 2 0 0 1 1 1 1 0 1 1 1 1 1 0 0 1 1 2 0 1 0 0 0 1 1 0 0 1 2 1 1 1 1 1 1 1 1 1 0 0 0 0 1 1 1 0 1 0 0 0 0 1 0 1 1 2 1 2 2 0 1 0 - 1 1

166-Hennig. Phylogenetic systematics, Tree #4 0 2 1 0 1 0 0 1 1 1 0 - - - - - - - - - - 1 0 0 1 1 0 0 0 0 0 1 1 0 1 0 1 1 1 2 1 1 0 0 0 2 1 - - - - - 1 2 1 0 1 1 0 0 0 1 0 0 0 0 0 0 0 0 0 0 0 0 0 2 2 0 0 1 1 1 1 0 1 1 1 1 1 0 0 1 1 2 0 1 0 0 0 1 1 0 0 1 2 1 1 ? 1 1 1 1 1 1 0 0 0 0 1 1 0 0 1 0 0 0 0 1 0 1 1 2 1 2 2 0 1 0 - 1 1

167-Romer. Vertebrate paleontology_2, Tree #1 2 0 2 0 0 0 0 0 0 0 0 0 1 0 0 0 1 0 1 0 ? 0 0 1 0 1 0 0 1 0 0 0 0 0 0 0 1 0 0 1 0 0 0 0 0 1 0 - - - - - 0 1 0 0 0 0 1 1 0 2 0 0 1 1 0 0 0 0 0 1 1 1 1 0 1 0 0 0 1 0 0 0 0 0 0 0 0 0 0 0 0 0 0 0 0 0 1 0 0 0 0 0 2 0 0 0 0 1 ? 0 1 0 0 0 0 0 0 0 1 0 0 1 0 1 0 1 1 1 0 2 1 1 1 0 0 0 - 0 0

168-Romer. Vertebrate paleontology_2, Tree #2 2 2 2 0 0 0 0 0 1 0 1 - - - - - - - - - - 0 0 1 0 1 0 1 1 0 0 1 0 0 0 0 1 0 0 1 0 0 0 0 1 1 0 1 1 0 1 0 0 1 0 0 0 0 1 1 0 1 2 0 1 1 0 0 0 0 0 1 1 1 1 0 1 0 0 0 1 0 0 0 0 1 0 0 0 0 0 0 0 1 0 0 0 0 1 0 0 0 0 0 2 0 0 0 0 1 ? 0 1 0 0 0 ? 0 0 0 1 0 0 1 0 0 0 1 1 1 0 2 1 1 1 0 0 0 - 0 0

169-Romer. Vertebrate paleontology_2, Tree #3 2 2 2 0 0 0 0 0 1 0 1 - - - - - - - - - - 0 0 1 0 1 0 0 1 0 0 1 0 0 0 0 1 0 0 1 0 0 0 0 1 1 0 1 1 0 0 0 0 1 0 0 0 0 1 1 0 1 2 0 0 1 0 0 0 0 0 1 1 1 1 0 2 0 0 0 1 0 0 0 0 0 0 0 0 0 0 0 0 1 0 0 0 0 1 0 0 0 0 0 2 0 0 0 0 1 ? 0 1 0 0 0 0 0 0 0 1 0 0 1 0 1 0 1 1 1 0 2 1 1 1 0 0 0 - 0 0

170-Romer. Vertebrate paleontology_2, Tree #4 2 2 2 0 0 1 0 0 1 0 0 - - - - - - - - - - 0 0 1 0 1 0 0 1 0 0 1 0 0 0 0 1 0 0 1 0 0 0 0 1 1 0 1 1 0 0 0 0 1 0 0 0 0 1 1 0 1 2 0 1 1 0 0 0 0 0 1 1 1 1 0 - - 0 0 1 0 0 0 0 1 0 0 0 0 0 0 0 1 0 0 0 0 1 0 0 0 0 0 2 0 0 0 0 1 0 0 1 0 0 0 0 0 0 0 1 0 0 1 0 1 0 1 1 1 0 2 1 1 1 0 0 0 - 0 0

171-Romer. Vertebrate paleontology_2, Tree #5 2 2 2 0 0 0 0 0 1 0 0 0 1 0 0 0 1 0 1 1 ? 0 0 1 0 1 0 0 1 0 0 0 0 0 0 0 1 0 0 1 0 0 0 0 0 1 0 - - - - - 0 1 0 0 0 0 1 1 0 2 0 0 1 1 0 0 0 0 0 1 1 1 1 0 - - 0 0 1 0 0 0 0 0 0 0 1 0 0 0 0 0 0 0 0 0 1 0 0 0 0 0 2 0 0 0 0 1 ? 0 1 0 0 0 0 0 0 0 1 0 0 1 0 1 0 1 1 1 0 2 1 1 1 0 0 0 - 0 0

172-Romer. Vertebrate paleontology_2, Tree #6 2 2 2 0 0 0 0 0 1 0 0 - - - - - - - - - - 0 0 1 0 1 0 0 1 0 0 1 0 0 0 0 1 0 0 1 0 0 0 0 1 1 0 1 1 0 0 0 0 1 0 0 0 0 1 1 0 1 2 0 1 1 0 0 0 0 0 1 1 1 1 0 2 0 0 0 1 0 0 0 0 1 0 0 0 0 0 0 0 1 0 0 0 0 1 0 0 0 0 0 2 0 0 0 0 1 ? 0 1 0 0 0 0 0 0 0 1 0 0 1 0 1 0 1 1 1 0 2 1 1 1 0 0 0 - 0 0

173-Romer. Vertebrate paleontology_2, Tree #7 2 0 2 0 0 0 0 0 1 0 0 - - - - - - - - - - 0 0 1 0 1 0 0 1 0 0 1 0 0 0 0 1 0 0 1 0 0 0 0 1 1 0 1 1 0 0 0 0 1 0 0 0 0 1 1 0 1 2 0 1 1 0 0 0 0 0 1 1 1 1 0 - - 0 0 1 0 0 0 0 1 0 0 0 0 0 0 0 1 0 0 0 0 1 0 0 0 0 0 2 0 0 0 0 1 ? 0 1 0 0 0 0 0 0 0 1 0 0 1 0 1 0 1 1 1 0 2 1 1 1 0 0 0 - 0 0

174-Romer. Vertebrate paleontology_2, Tree #8 2 2 2 0 0 1 0 0 1 0 1 - - - - - - - - - - 0 0 1 0 1 0 0 1 0 0 1 0 0 0 0 1 0 0 1 0 0 0 0 1 1 0 1 1 0 0 0 0 1 0 0 0 0 1 1 0 1 2 0 0 1 0 0 0 0 0 1 1 1 1 0 1 0 0 0 1 0 0 0 0 0 0 0 0 0 0 0 0 1 0 0 0 0 1 0 0 0 0 0 2 0 0 0 0 1 ? 0 1 0 0 0 0 0 0 0 1 0 0 1 0 1 0 1 1 1 0 2 1 1 1 0 0 0 - 0 0

175-Romer. Vertebrate paleontology_2, Tree #9 2 2 2 0 0 0 0 0 1 0 1 - - - - - - - - - - 0 0 1 0 1 0 0 1 0 0 1 0 0 0 0 1 0 0 1 0 0 0 0 1 1 0 1 1 0 0 0 0 1 0 0 0 0 1 1 0 1 2 0 0 1 0 0 0 0 0 1 1 1 1 0 2 0 0 0 1 0 0 0 0 0 0 0 0 0 0 0 0 1 0 0 0 0 1 0 0 0 0 0 2 0 0 0 0 1 ? 0 1 0 0 0 0 0 0 0 1 0 0 1 0 1 0 1 1 1 0 2 1 1 1 0 0 0 - 0 0

176-Romer. Vertebrate paleontology_2, Tree #10 2 2 2 0 0 0 0 0 1 0 1 - - - - - - - - - - 0 0 1 0 1 0 1 1 0 0 1 0 0 0 0 1 0 0 1 0 0 0 0 1 1 0 1 1 0 0 0 0 1 0 0 0 0 1 1 0 1 2 0 1 1 0 0 0 0 0 1 1 1 1 0 - - 0 0 1 0 0 0 0 1 0 0 0 0 0 0 0 1 0 0 0 0 1 0 0 0 0 0 2 0 0 0 0 1 ? 0 1 0 0 0 1 0 0 0 1 0 0 1 0 1 0 1 1 1 0 2 1 1 1 0 0 0 - 0 0

177-Romer. Vertebrate paleontology_2, Tree #11 2 2 2 0 0 1 0 0 1 0 0 - - - - - - - - - - 0 0 1 0 1 0 1 1 0 0 1 0 0 0 0 1 0 0 1 0 0 0 0 1 1 0 1 1 0 0 0 0 1 0 0 0 0 1 1 0 1 2 0 1 1 0 0 0 0 0 1 1 1 1 0 - - 0 0 1 0 0 0 0 1 0 0 0 0 0 0 0 1 0 0 0 0 1 0 0 0 0 0 2 0 0 0 0 1 ? 0 1 0 0 0 1 0 0 0 1 0 0 1 0 1 0 1 1 1 0 2 1 1 1 0 0 0 - 0 0

178-Romer. Vertebrate paleontology_2, Tree #12 2 2 2 0 0 1 0 0 1 0 0 - - - - - - - - - - 0 0 1 0 1 0 0 1 0 0 1 0 0 0 0 1 0 0 1 0 0 0 0 1 1 0 1 1 0 0 0 0 1 0 0 0 0 1 1 0 1 2 0 1 1 0 0 0 0 0 1 1 1 1 0 - - 0 0 1 0 0 0 0 1 0 0 0 0 0 0 0 1 0 0 0 0 1 0 0 0 0 0 2 0 0 0 0 1 ? 0 1 0 0 0 1 0 0 0 1 0 0 1 0 1 0 1 1 1 0 2 1 1 1 0 0 0 - 0 0

179-Romer. Vertebrate paleontology_2, Tree #13 2 2 2 0 0 1 0 0 1 0 0 - - - - - - - - - - 0 0 1 0 1 0 0 1 0 0 1 0 0 0 0 1 0 0 1 0 0 0 0 1 1 0 1 1 0 0 0 0 1 0 0 0 0 1 1 0 1 2 0 0 1 0 0 0 0 0 1 1 1 1 0 - - 0 0 1 0 0 0 0 1 0 0 0 0 0 0 0 1 0 0 0 0 1 0 0 0 0 0 2 0 0 0 0 1 ? 0 1 0 0 0 1 0 0 0 1 0 0 1 0 1 0 1 1 1 0 2 1 1 1 0 0 0 - 0 0

180-Romer. Vertebrate paleontology_2, Tree #14 2 2 2 0 0 0 0 0 1 0 1 - - - - - - - - - - 0 0 1 0 1 0 0 1 0 0 1 0 0 0 0 1 0 0 1 0 0 0 0 1 1 0 1 1 0 1 0 0 1 0 0 0 0 1 1 0 1 2 0 1 1 0 0 0 0 0 1 1 1 1 0 1 0 0 0 1 0 0 0 0 1 0 0 0 0 0 0 0 1 0 0 0 0 1 0 0 0 0 0 2 0 0 0 0 1 ? 0 1 0 0 0 ? 0 0 0 1 0 0 1 0 1 0 1 1 1 0 2 1 1 1 0 0 0 - 0 0

181-Romer. Vertebrate paleontology_3, Tree #1 2 0 2 0 0 0 0 0 0 0 1 - - - - - - - - - - 0 0 1 0 1 0 1 1 0 0 0 0 0 0 0 1 0 0 1 0 0 0 0 1 1 0 1 1 0 0 0 0 1 0 0 0 0 1 1 0 2 0 0 1 1 0 0 0 0 0 1 1 1 1 0 1 0 0 0 1 0 0 0 0 0 0 0 0 0 0 0 0 1 0 0 0 0 1 0 0 0 0 0 2 0 0 0 0 1 ? 0 1 0 0 0 0 0 0 0 1 0 0 1 0 1 0 1 1 1 0 2 1 1 1 0 0 0 - 0 0

182-Romer. Vertebrate paleontology_3, Tree #2 2 2 2 0 0 0 0 0 1 0 1 - - - - - - - - - - 0 0 1 0 1 0 1 1 0 0 1 0 0 0 0 1 0 0 1 0 0 0 0 1 1 0 1 1 0 1 0 0 1 0 0 0 0 1 1 0 1 2 0 1 1 0 0 0 0 0 1 1 1 1 0 1 0 0 0 1 0 0 0 0 1 0 0 0 0 0 0 0 1 0 0 0 0 1 0 0 0 0 0 2 0 0 0 0 1 ? 0 1 0 0 0 ? 0 0 0 1 0 0 1 0 0 0 1 1 1 0 2 1 1 1 0 0 0 - 0 0

183-Romer. Vertebrate paleontology_3, Tree #3 2 2 2 0 0 0 0 0 1 0 1 - - - - - - - - - - 0 0 1 0 1 0 0 1 0 0 1 0 0 0 0 1 0 0 1 0 0 0 0 1 1 0 1 1 0 0 0 0 1 0 0 0 0 1 1 0 1 2 0 0 1 0 0 0 0 0 1 1 1 1 0 2 0 0 0 1 0 0 0 0 0 0 0 0 0 0 0 0 1 0 0 0 0 1 0 0 0 0 0 2 0 0 0 0 1 ? 0 1 0 0 0 0 0 0 0 1 0 0 1 0 1 0 1 1 1 0 2 1 1 1 0 0 0 - 0 0

184-Romer. Vertebrate paleontology_3, Tree #4 2 2 2 0 0 1 0 0 1 0 0 - - - - - - - - - - 0 0 1 0 1 0 0 1 0 0 1 0 0 0 0 1 0 0 1 0 0 0 0 1 1 0 1 1 0 0 0 0 1 0 0 0 0 1 1 0 1 2 0 1 1 0 0 0 0 0 1 1 1 1 0 - - 0 0 1 0 0 0 0 1 0 0 0 0 0 0 0 1 0 0 0 0 1 0 0 0 0 0 2 0 0 0 0 1 0 0 1 0 0 0 0 0 0 0 1 0 0 1 0 1 0 1 1 1 0 2 1 1 1 0 0 0 - 0 0

185-Romer. Vertebrate paleontology_3, Tree #5 2 2 2 0 0 0 0 0 1 0 0 - - - - - - - - - - 0 0 1 0 1 0 0 1 0 0 0 0 0 0 0 1 0 0 1 0 0 0 0 0 1 0 - - - - - 0 1 0 0 0 0 1 1 0 2 0 0 1 1 0 0 0 0 0 1 1 1 1 0 - - 0 0 1 0 0 0 0 0 0 0 1 0 0 0 0 0 0 0 0 0 1 0 0 0 0 0 2 0 0 0 0 1 ? 0 1 0 0 0 0 0 0 0 1 0 0 1 0 1 0 1 1 1 0 2 1 1 1 0 0 0 - 0 0

186-Romer. Vertebrate paleontology_3, Tree #6 2 2 2 0 0 0 0 0 1 0 0 - - - - - - - - - - 0 0 1 0 1 0 0 1 0 0 1 0 0 0 0 1 0 0 1 0 0 0 0 1 1 0 1 1 0 0 0 0 1 0 0 0 0 1 1 0 1 2 0 1 1 0 0 0 0 0 1 1 1 1 0 2 0 0 0 1 0 0 0 0 1 0 0 0 0 0 0 0 1 0 0 0 0 1 0 0 0 0 0 2 0 0 0 0 1 ? 0 1 0 0 0 0 0 0 0 1 0 0 1 0 1 0 1 1 1 0 2 1 1 1 0 0 0 - 0 0

187-Romer. Vertebrate paleontology_3, Tree #7 2 0 2 0 0 0 0 0 1 0 0 - - - - - - - - - - 0 0 1 0 1 0 0 1 0 0 1 0 0 0 0 1 0 0 1 0 0 0 0 1 1 0 1 1 0 1 0 0 1 0 0 0 0 1 1 0 1 2 0 1 1 0 0 0 0 0 1 1 1 1 0 - - 0 0 1 0 0 0 0 1 0 0 0 0 0 0 0 1 0 0 0 0 1 0 0 0 0 0 2 0 0 0 0 1 ? 0 1 0 0 0 0 0 0 0 1 0 0 1 0 1 0 1 1 1 0 2 1 1 1 0 0 0 - 0 0

188-Romer. Vertebrate paleontology_3, Tree #8 2 2 2 0 0 0 0 0 1 0 1 - - - - - - - - - - 0 0 1 0 1 0 0 1 0 0 1 0 0 0 0 1 0 0 1 0 0 0 0 1 1 0 1 1 0 0 0 0 1 0 0 0 0 1 1 0 1 2 0 0 0 0 0 0 0 0 1 1 1 1 0 2 0 0 0 1 0 0 0 0 0 0 0 0 0 0 0 0 1 0 0 0 0 1 0 0 0 0 0 2 0 0 0 0 1 ? 0 1 0 0 0 0 0 0 0 1 0 0 1 0 1 0 1 1 1 0 2 1 1 1 0 0 0 - 0 0

189-Romer. Vertebrate paleontology_3, Tree #9 2 2 2 0 0 0 0 0 1 0 1 - - - - - - - - - - 0 0 1 0 1 0 1 1 0 0 1 0 0 0 0 1 0 0 1 0 0 0 0 1 1 0 1 1 0 0 0 0 1 0 0 0 0 1 1 0 1 2 0 1 1 0 0 0 0 0 1 1 1 1 0 - - 0 0 1 0 0 0 0 1 0 0 0 0 0 0 0 1 0 0 0 0 1 0 0 0 0 0 2 0 0 0 0 1 ? 0 1 0 0 0 1 0 0 0 1 0 0 1 0 1 0 1 1 1 0 2 1 1 1 0 0 0 - 0 0

190-Romer. Vertebrate paleontology_3, Tree #10 2 2 2 0 0 0 0 0 1 0 0 - - - - - - - - - - 0 0 1 0 1 0 1 1 0 0 1 0 0 0 0 1 0 0 1 0 0 0 0 1 1 0 1 1 0 0 0 0 1 0 0 0 0 1 1 0 1 2 0 1 1 0 0 0 0 0 1 1 1 1 0 - - 0 0 1 0 0 0 0 1 0 0 0 0 0 0 0 1 0 0 0 0 1 0 0 0 0 0 2 0 0 0 0 1 ? 0 1 0 0 0 1 0 0 0 1 0 0 1 0 1 0 1 1 1 0 2 1 1 1 0 0 0 - 0 0

191-Romer. Vertebrate paleontology_3, Tree #11 2 0 2 0 0 0 0 0 1 0 1 - - - - - - - - - - 0 0 1 0 1 0 0 1 0 0 1 0 0 0 0 1 0 0 1 0 0 0 0 1 1 0 1 1 0 1 0 0 1 0 0 0 0 1 1 0 1 2 0 1 1 0 0 0 0 0 1 1 1 1 0 1 0 0 0 1 0 0 0 0 1 0 0 0 0 0 0 0 1 0 0 0 0 1 0 0 0 0 0 2 0 0 0 0 1 ? 0 1 0 0 0 ? 0 0 0 1 0 0 1 0 1 0 1 1 1 0 2 1 1 1 0 0 0 - 0 0

192-Romer. Vertebrate paleontology_3, Tree #12 2 2 2 0 0 1 0 0 1 0 0 - - - - - - - - - - 0 0 1 0 1 0 0 1 0 0 1 0 0 0 0 1 0 0 1 0 0 0 0 1 1 0 1 1 0 0 0 0 1 0 0 0 0 1 1 0 1 2 0 0 1 0 0 0 0 0 1 1 1 1 0 - - 0 0 1 0 0 0 0 1 0 0 0 0 0 0 0 1 0 0 0 0 1 0 0 0 0 0 2 0 0 0 0 1 ? 0 1 0 0 0 1 0 0 0 1 0 0 1 0 1 0 1 1 1 0 2 1 1 1 0 0 0 - 0 0

193-Romer. Vertebrate paleontology_3, Tree #13 2 2 2 0 0 0 0 0 1 0 1 - - - - - - - - - - 0 0 1 0 1 0 0 1 0 0 1 0 0 0 0 1 0 0 1 0 0 0 0 1 1 0 1 1 0 1 0 0 1 0 0 0 0 1 1 0 1 2 0 1 1 0 0 0 0 0 1 1 1 1 0 1 0 0 0 1 0 0 0 0 1 0 0 0 0 0 0 0 1 0 0 0 0 1 0 0 0 0 0 2 0 0 0 0 1 ? 0 1 0 0 0 ? 0 0 0 1 0 0 1 0 1 0 1 1 1 0 2 1 1 1 0 0 0 - 0 0

194-Sokal. numerical taxonomy-article 0 2 0 0 0 0 0 - - - 0 - - - - - - - - - - 1 0 0 - - 0 0 1 1 0 0 1 0 0 1 0 1 - 0 1 1 0 0 0 0 1 - - - - - 1 2 1 0 1 1 0 1 1 0 0 0 0 0 0 0 0 0 0 - - - - - 2 0 1 1 0 1 1 0 1 1 1 1 1 0 0 1 1 2 0 1 0 1 0 1 1 1 0 1 2 1 1 1 1 1 1 1 0 1 0 0 0 0 1 1 0 0 0 0 0 0 0 0 0 0 0 0 1 1 1 0 0 1 - 1 1

195-Greenwood et al. Phyletic studies of teleostean fishes 2 0 2 0 0 0 0 0 1 0 1 - - - - - - - - - - 0 0 1 0 1 0 0 1 0 0 1 0 0 0 0 1 0 1 1 0 1 0 0 1 1 ? 0 1 0 0 2 0 1 0 0 0 0 1 1 0 2 0 0 0 0 0 0 0 0 0 1 1 0 1 0 - - 0 0 1 0 0 0 0 0 0 1 1 0 0 0 0 1 0 1 0 0 1 0 0 0 0 0 2 0 0 0 0 1 ? 0 1 0 0 0 0 0 0 0 1 0 0 1 0 0 0 1 1 1 0 2 1 1 1 0 0 0 - 0 0

196-Fitch. Construction of phylogenetic trees, Tree #1 0 2 0 0 0 0 0 - - - 0 - - - - - - - - - - 1 0 0 - - 0 0 1 1 0 0 1 0 0 1 0 1 - 0 1 1 0 0 0 0 1 - - - - - 1 2 1 0 1 1 0 1 1 0 0 0 0 0 0 0 0 0 0 0 0 - - - 2 0 1 1 1 1 1 0 1 1 1 1 1 0 0 1 1 2 0 1 0 0 0 1 1 0 0 1 2 1 1 1 1 1 1 1 0 1 0 0 0 1 1 1 1 0 0 0 0 0 0 0 0 0 0 2 ? 1 1 0 1 0 - 1 1

197-Fitch. Construction of phylogenetic trees, Tree #2 0 2 0 0 0 0 0 - - - 0 - - - - - - - - - - 1 0 0 - - 0 0 1 1 0 0 1 0 0 1 0 1 - 0 1 1 0 0 0 0 1 - - - - - 1 2 1 0 1 1 0 1 1 0 0 0 0 0 0 0 0 0 0 0 0 - - - 2 0 1 1 1 1 1 0 1 1 1 1 1 0 0 1 1 2 0 1 0 0 0 1 1 0 0 1 2 1 1 1 1 1 1 1 0 1 0 0 0 1 1 1 1 0 0 0 0 0 0 0 0 0 0 2 ? 1 1 0 1 0 - 1 1

198-Romer. Major steps in vertebrate evolution 2 0 2 0 0 0 0 0 1 0 0 - - - - - - - - - - 0 1 1 0 1 0 0 1 0 1 1 0 1 0 0 1 0 1 1 0 0 ? 0 0 1 0 - - - - - 0 1 0 0 0 0 1 1 0 1 0 0 0 0 0 0 0 0 0 1 1 1 1 0 0 0 0 0 1 0 0 0 0 0 0 0 0 0 0 0 0 1 0 1 0 0 1 0 0 1 0 0 1 0 0 0 0 1 0 0 1 0 0 0 1 0 0 0 1 0 1 1 0 0 0 1 1 1 0 2 1 1 1 0 0 0 - 0 0

199-Romer. L-origine des classes de vertebres, Tree #1 2 0 2 0 0 0 0 0 1 0 0 - - - - - - - - - - 0 1 1 0 1 0 1 1 0 0 1 0 0 0 0 1 0 0 1 0 0 0 0 1 1 0 1 1 0 0 0 0 1 0 0 0 0 1 1 0 1 2 0 1 1 0 0 0 0 0 1 1 0 1 0 - 0 0 0 1 0 0 0 0 0 0 0 0 0 0 0 0 1 0 1 0 0 1 0 0 0 0 0 2 0 0 1 0 1 0 0 1 0 0 0 0 0 0 0 1 0 0 1 0 1 0 1 1 1 0 2 1 1 1 0 0 0 - 0 0

200-Romer. L-origine des classes de vertebres, Tree #2 2 0 2 0 0 0 0 0 1 0 0 - - - - - - - - - - 0 0 1 0 1 0 1 1 0 0 1 0 0 0 0 1 0 0 1 0 0 0 0 1 1 0 1 1 0 0 0 0 1 0 0 0 0 1 1 0 1 2 0 1 1 0 0 0 0 0 1 1 0 1 0 1 0 0 0 1 0 0 0 0 0 0 0 0 0 0 0 0 1 0 1 0 0 1 0 0 0 0 0 2 0 0 1 0 1 0 0 1 0 0 0 0 0 0 0 1 0 0 1 0 1 0 1 1 1 0 2 1 1 1 0 0 0 - 0 0

201-Sokal & sneath. numerical taxonomy II, Tree #1 0 2 1 0 0 0 0 1 1 1 0 - - - - - - - - - - 1 0 0 1 1 0 0 0 0 0 1 1 0 1 0 1 1 1 2 1 1 0 0 0 2 1 - - - - - 1 2 1 0 1 1 0 0 0 1 0 0 0 0 0 0 0 0 0 0 0 0 0 2 2 0 0 1 1 1 1 0 1 1 1 1 1 0 0 1 1 2 0 1 0 0 0 1 1 0 0 1 2 1 1 1 1 1 1 1 1 1 0 0 0 1 1 1 1 0 0 0 0 0 0 1 0 1 1 2 ? 2 2 1 1 0 - 1 1

202-Sokal & sneath. numerical taxonomy II, Tree #2 0 2 0 0 0 0 0 - - - 0 - - - - - - - - - - 1 0 0 - - 0 0 1 1 0 0 1 0 0 1 0 1 - 0 1 1 0 0 0 0 1 - - - - - 1 2 1 0 1 1 0 1 1 0 0 0 0 0 0 0 0 0 0 - - - - - 2 0 1 1 0 1 1 0 1 1 1 1 1 0 0 1 1 2 0 1 0 1 0 1 1 1 0 1 2 1 1 1 1 1 1 1 0 1 0 0 0 0 1 1 1 0 0 0 0 0 0 0 0 0 0 0 ? 1 1 0 0 1 - 1 1

203-Mayr. Cladistic analysis or cladistic classification ?, Tree 2 2 2 0 1 0 0 0 1 1 0 - - - - - - - - - - 0 0 1 1 1 0 0 0 1 0 1 0 0 1 0 1 0 ? 1 ? 1 0 0 0 1 1 - - - - - 1 1 0 0 1 1 0 0 0 1 0 0 1 1 0 0 0 0 0 1 1 ? 0 0 0 0 0 0 1 0 1 0 0 0 1 1 1 0 0 0 0 1 0 1 1 0 0 1 1 0 0 1 2 0 0 1 0 1 1 0 1 1 0 0 0 ? ? 0 0 0 1 0 0 0 0 1 1 1 0 2 1 1 2 0 1 1 - 0 1

204-Mayr. Cladistic analysis or cladistic classification ?, Tree 2 2 2 0 1 0 0 0 1 1 0 - - - - - - - - - - 0 0 1 1 1 0 0 0 1 0 1 0 0 1 0 1 0 ? 1 ? 1 0 0 0 1 1 - - - - - 1 1 0 0 1 1 0 0 0 1 0 0 1 1 0 0 0 0 0 1 1 ? 0 0 0 0 0 0 1 0 1 0 0 0 1 1 1 0 0 0 1 1 2 1 1 0 0 1 1 0 0 1 2 0 0 1 0 1 0 0 1 1 0 0 0 ? ? 0 0 0 1 0 0 0 0 1 1 1 0 2 1 1 2 0 1 1 - 0 1

205-Mayr. Cladistic analysis or cladistic classification ?, Tree 2 2 2 0 0 0 0 ? 1 1 0 - - - - - - - - - - 0 0 1 1 1 0 0 0 1 0 1 0 0 1 0 1 0 ? 1 0 1 0 0 0 1 0 1 1 0 1 2 1 1 0 0 1 1 0 ? 0 1 0 0 1 1 0 0 0 0 0 1 1 ? 0 0 1 0 0 0 1 0 1 0 0 0 1 1 1 0 0 0 0 1 0 1 1 0 1 1 1 0 0 1 2 0 0 1 0 1 0 0 1 1 1 0 0 0 0 0 1 0 1 0 0 0 0 1 1 1 0 2 1 1 2 0 1 1 - 0 1

206-Mayr. Cladistic analysis or cladistic classification ?, Tree 2 2 2 0 0 0 0 ? 1 1 0 - - - - - - - - - - 0 0 1 1 1 0 0 0 1 0 1 0 0 1 0 1 0 ? 1 0 1 0 0 0 1 0 1 1 0 1 2 1 1 0 0 1 1 0 ? 0 1 0 0 1 1 0 0 0 1 0 1 1 ? 0 0 - 0 0 0 1 0 1 0 0 0 1 1 1 0 0 0 0 1 0 1 1 0 1 1 1 0 0 1 2 0 0 1 0 1 0 0 1 1 1 0 0 0 0 0 1 0 1 0 0 0 0 1 1 1 0 2 1 1 2 0 1 1 - 0 1

207-Hul Thol. Révision de quelques genres de Caesalpiniacea 0 2 0 0 0 0 1 - - - 0 - - - - - - - - - - 1 0 0 1 1 0 0 1 1 0 0 1 0 0 1 0 ? - 0 0 1 0 0 0 0 1 - - - - - 1 2 1 0 1 1 0 1 1 0 0 0 0 0 0 0 0 0 0 0 0 0 0 - - - 1 0 0 1 1 0 1 1 1 1 1 0 0 1 1 2 0 1 0 0 0 1 1 0 0 1 2 1 1 0 1 1 1 1 0 1 0 0 0 1 1 1 1 0 0 1 1 0 0 0 0 0 0 0 ? 1 2 0 0 1 1 1 1

208-Harper. A bayesian probability view of phylogenetic syste 0 2 1 0 1 0 0 1 1 1 0 - - - - - - - - - - 1 0 0 1 1 0 0 0 0 0 1 1 0 1 0 1 1 1 2 1 1 0 0 0 2 1 - - - - - 1 2 1 0 1 1 0 0 0 1 0 0 0 0 0 0 0 0 0 0 0 0 0 2 2 0 0 1 1 1 1 0 1 1 1 1 1 0 0 1 1 2 0 1 0 0 0 1 1 0 0 1 2 1 1 1 1 1 1 1 1 1 0 0 0 0 1 1 0 0 1 0 0 0 0 1 0 1 1 2 1 2 2 1 ? 0 0 1 1

209-Kemp. The reptiles that became mammals 2 0 2 0 0 0 0 0 1 0 0 - - - - - - - - - - 0 0 1 0 1 0 0 1 0 0 1 0 0 0 0 1 0 1 1 0 0 0 0 0 1 1 - - - - - 0 1 0 0 0 0 1 1 0 2 0 0 0 0 0 0 0 0 0 1 1 0 1 0 1 0 0 0 1 1 0 0 0 1 1 ? 1 0 0 0 0 1 0 0 0 0 1 0 0 0 0 0 1 0 1 0 0 1 0 0 1 0 0 0 0 1 0 1 1 0 0 1 0 0 0 1 1 1 0 2 1 1 1 0 0 0 1 0 0

210-Margulis & Chapman. Kingdoms & Domains, Tree #1 0 2 1 0 1 0 0 ? 1 1 0 - - - - - - - - - - 1 0 0 1 1 0 0 0 ? 0 1 1 0 1 ? 1 1 1 2 1 1 0 0 0 2 1 - - - - - 1 2 1 0 1 1 0 0 ? 1 0 0 0 0 0 0 0 0 0 0 0 0 0 C 2 0 0 1 1 1 1 0 1 1 1 1 1 0 0 1 1 2 0 0 0 0 0 0 0 0 1 1 2 ? 1 1 1 1 1 1 1 1 0 0 0 1 1 1 0 0 0 1 0 0 0 1 0 1 1 2 - C 2 0 1 ? ? 1 1

211-Margulis & Chapman. Kingdoms & Domains, Tree #2 0 2 1 0 ? 0 0 1 1 1 0 - - - - - - - - - - 1 0 0 1 1 0 0 ? ? 0 1 0 0 ? ? 1 1 1 2 0 1 0 0 0 2 1 - - - - - 1 2 1 1 1 1 0 ? ? 1 0 0 0 0 0 0 0 0 0 0 0 0 0 C - - 0 1 1 1 1 0 1 1 1 1 1 0 0 1 1 2 0 0 0 0 1 0 0 0 1 1 2 ? 1 1 1 1 1 1 1 1 1 0 0 1 0 1 1 0 0 1 0 1 0 1 0 1 1 2 - C 2 1 ? ? ? 1 1

212-Margulis & Chapman. Kingdoms & Domains, Tree #3 0 0 C 0 1 1 0 1 0 1 1 - - - - - - - - - - 1 0 0 1 1 1 1 0 0 0 1 1 0 1 0 1 1 1 2 0 1 0 1 1 2 1 - - - - - 1 2 1 0 1 1 0 0 0 1 2 0 0 0 0 0 0 0 0 0 0 0 0 2 2 0 0 1 1 1 1 0 1 1 1 1 1 1 0 1 1 2 0 0 0 0 0 0 0 0 1 0 2 1 1 ? 1 1 1 1 1 1 0 0 0 0 0 0 0 0 0 1 0 1 1 1 0 1 1 2 - 2 2 0 1 0 ? 1 1

213-Margulis & Chapman. Kingdoms & Domains, Tree #4 0 2 1 0 1 0 0 1 1 1 0 - - - - - - - - - - 1 0 0 1 1 0 0 ? ? 0 1 0 0 1 ? 1 1 1 2 0 1 0 0 0 2 1 - - - - - 1 2 1 0 1 1 0 0 0 1 0 0 0 0 0 0 0 0 0 0 0 0 0 2 2 0 0 0 1 1 1 0 1 1 1 1 1 0 0 1 1 0 2 0 0 0 1 0 0 0 0 1 2 1 1 1 1 1 0 1 1 1 0 0 0 1 0 1 0 0 0 1 0 1 0 1 0 1 1 2 1 C C 1 1 ? ? 1 1

214-Margulis & Chapman. Kingdoms & Domains, Tree #5 0 2 1 0 1 0 0 1 1 1 0 - - - - - - - - - - 1 0 0 1 1 0 0 0 0 0 1 1 0 1 0 1 1 1 2 1 1 0 0 0 2 1 - - - - - 1 2 1 0 1 1 0 0 0 1 0 1 0 0 0 0 0 0 0 0 0 0 0 2 - - 0 1 1 1 1 0 1 1 1 1 1 0 0 1 1 0 0 1 0 0 0 0 0 0 0 1 2 1 1 1 1 1 1 1 1 1 0 0 0 1 1 1 1 0 0 1 0 0 0 1 0 1 1 2 1 2 2 1 1 0 0 1 1

215-Margulis & Chapman. Kingdoms & Domains, Tree #6 0 0 2 0 1 0 0 1 1 1 0 - - - - - - - - - - 1 0 0 1 1 0 0 0 ? 0 1 1 0 1 ? 1 1 1 2 0 ? 0 0 0 2 1 - - - - - 1 2 1 0 1 1 0 0 ? 1 2 0 0 0 0 1 0 0 0 0 0 0 0 ? 2 0 0 1 1 1 1 0 1 1 1 1 1 0 0 1 1 0 0 0 0 0 1 0 0 0 0 1 2 1 1 1 1 1 1 1 1 1 0 0 0 0 0 1 1 0 0 1 0 1 0 1 0 1 1 2 1 C C 1 ? ? ? 1 1

216-Margulis & Chapman. Kingdoms & Domains, Tree #7 0 0 2 0 1 0 0 1 1 1 0 1 1 ? 1 1 1 0 1 0 1 1 0 0 1 1 0 0 0 ? 0 1 1 0 1 ? 1 1 1 2 0 ? 0 0 0 2 1 - - - - - 1 2 1 0 1 1 0 0 ? 1 2 0 0 0 0 1 0 0 0 0 0 0 0 ? 2 0 0 1 1 1 1 0 1 1 1 1 1 1 0 1 1 0 0 0 0 0 1 0 0 0 0 1 2 1 1 1 1 1 1 1 1 1 0 0 0 0 0 1 1 0 0 1 0 1 0 1 0 1 1 2 1 C C 1 ? ? ? 1 1

217-Margulis & Chapman. Kingdoms & Domains, Tree #8 0 0 2 0 1 0 0 1 1 1 0 1 1 ? 1 1 1 0 1 0 1 1 0 0 1 1 0 0 0 ? 0 1 1 0 1 ? 1 1 1 2 0 ? 0 0 0 2 1 0 0 1 0 2 1 2 1 0 1 1 0 0 ? 1 2 0 0 0 0 1 0 0 0 0 0 0 0 ? 2 0 0 1 1 1 1 0 1 1 1 1 1 0 0 1 1 0 0 0 0 0 1 0 0 0 0 1 2 1 1 1 1 1 1 1 1 1 0 0 0 0 0 1 1 0 0 1 0 1 0 1 0 1 1 2 1 C C 1 ? ? ? 1 1

218-Margulis & Schwartz. Five kingdoms, Tree #1 0 2 2 0 1 0 0 1 1 1 0 - - - - - - - - - - 1 0 0 1 1 0 0 ? ? 0 0 1 0 ? ? 1 ? 1 ? 0 1 0 0 1 C 0 0 1 0 0 1 1 1 0 0 0 0 0 0 0 2 0 0 0 0 0 0 0 0 0 1 0 0 0 ? 2 0 0 0 1 1 0 0 0 0 0 1 0 0 0 0 1 1 1 0 0 0 1 0 0 1 0 0 2 0 ? 1 0 1 0 0 1 0 0 0 0 0 0 0 0 0 0 1 0 1 1 1 1 1 0 2 1 1 1 0 0 0 1 0 0

219-Margulis & Schwartz. Five kingdoms, Tree #2 0 0 2 0 1 0 0 ? 0 1 0 1 1 0 1 1 1 0 1 0 1 1 0 0 1 1 0 0 0 0 0 1 1 0 1 0 1 1 1 2 0 1 0 0 0 2 1 - - - - - 1 2 1 1 1 1 0 0 0 1 0 0 0 1 0 0 0 0 0 0 0 0 0 ? - - 0 1 1 1 0 0 1 1 1 1 1 0 0 1 1 2 0 0 0 0 0 0 0 0 1 0 2 1 1 1 1 1 1 1 1 1 1 0 0 0 0 0 1 0 0 1 0 1 0 1 1 1 1 2 1 2 1 0 1 0 1 ? 1

220-Margulis & Schwartz. Five kingdoms, Tree #3 0 0 2 0 ? 0 0 ? 1 1 0 1 1 0 1 1 0 0 0 0 1 1 0 0 1 1 0 0 1 0 0 1 1 0 1 0 1 1 1 2 0 0 0 0 0 2 1 - - - - - 1 2 1 0 1 0 0 0 0 1 0 0 0 0 0 0 0 0 0 0 0 0 0 ? 2 0 0 1 1 1 0 0 1 1 1 1 1 0 0 1 1 2 0 0 0 0 0 0 0 0 1 0 2 1 1 1 1 1 1 1 1 0 0 0 0 0 0 1 1 0 0 1 0 1 1 1 1 1 0 2 1 ? 1 0 ? ? ? 0 1

221-Saitou. & Nei. The neighbor-joining method, Tree #1 0 2 0 0 0 0 0 - - - 0 - - - - - - - - - - 1 0 0 - - 0 0 1 1 0 0 1 0 0 1 0 1 - 0 1 1 0 0 0 0 1 - - - - - 1 2 1 0 1 1 0 1 1 0 0 0 0 0 0 0 0 0 0 0 0 - - - 2 0 1 1 0 1 1 0 1 1 1 1 1 0 0 1 1 2 0 1 0 0 0 1 1 1 0 1 2 1 1 0 1 1 1 1 ? 1 0 0 0 0 1 1 1 0 1 0 0 0 0 0 0 1 0 0 ? 1 1 0 0 1 1 1 1

222-Saitou. & Nei. The neighbor-joining method, Tree #2 0 2 0 0 0 0 0 - - - 0 - - - - - - - - - - 1 0 0 - - 0 0 1 1 0 0 1 0 0 1 0 1 - 0 1 1 0 0 0 0 1 - - - - - 1 2 1 0 1 1 0 1 1 0 0 0 0 0 0 0 0 0 0 0 0 - - - 2 0 1 1 0 1 1 0 1 1 1 1 1 0 0 1 1 2 0 1 0 0 0 1 1 1 0 1 2 1 1 1 1 1 1 1 ? 1 0 0 0 0 1 1 1 0 1 0 0 0 0 0 0 1 0 0 ? 1 1 0 0 1 1 1 1

223-Sober. Reconstructing the past 0 2 1 0 1 0 0 1 1 1 0 - - - - - - - - - - 1 0 0 1 1 0 0 0 0 0 1 1 0 1 0 ? 1 1 2 1 1 0 0 0 2 1 - - - - - 1 2 1 0 1 1 0 0 0 1 0 0 0 0 0 0 0 0 0 0 0 0 0 2 2 0 0 1 1 1 1 0 1 1 1 1 1 0 0 1 1 2 0 1 0 0 0 1 1 0 0 1 2 1 1 1 1 1 1 1 1 1 0 0 0 1 1 1 0 0 1 0 0 0 0 1 0 1 1 2 1 2 2 1 1 0 1 1 1

224-Nelson. & Platnick. Three-taxon statements 0 2 A 0 1 0 0 1 1 1 0 - - - - - - - - - - 1 0 0 1 1 0 0 0 0 0 1 1 0 1 0 1 1 1 2 1 1 0 0 0 2 1 - - - - - 1 2 1 0 1 1 0 0 0 1 0 0 0 0 0 0 0 0 0 0 0 0 0 2 2 0 0 1 1 1 1 0 1 1 1 1 1 0 0 1 1 2 0 1 0 0 0 1 0 0 0 1 2 1 1 1 1 1 1 1 0 1 1 0 0 0 1 1 0 0 1 0 0 0 0 1 0 1 1 2 1 2 2 1 1 0 1 1 1

225-Janvier. Early vertebrates 0 2 1 0 1 0 0 1 1 1 0 - - - - - - - - - - 1 0 0 1 1 0 0 0 0 0 1 1 0 1 0 1 1 1 2 0 1 0 0 0 2 1 - - - - - 1 2 1 0 1 1 0 0 0 1 0 0 0 0 0 0 0 0 0 0 0 0 0 2 - - 0 1 1 1 1 0 1 1 1 1 1 0 0 1 1 2 0 1 0 0 0 0 1 0 0 1 2 1 1 1 1 1 1 1 1 1 0 0 0 1 1 1 1 0 0 1 0 0 0 1 1 1 1 2 1 2 2 1 1 0 1 1 1

226-Smith et al. From bilateral symmetry to pentaradiality, Tre 0 2 1 0 1 0 0 1 1 1 0 - - - - - - - - - - 1 0 0 1 1 0 0 0 0 0 1 1 0 1 0 1 1 1 2 0 1 0 0 0 2 1 - - - - - 1 2 1 0 1 1 0 0 0 1 0 0 0 0 0 0 0 0 0 0 0 0 0 2 2 0 0 1 1 1 1 0 1 1 1 1 1 0 0 1 1 2 0 1 0 0 0 0 1 0 0 1 2 1 1 1 1 1 1 1 1 1 0 0 0 0 1 1 1 0 0 1 0 0 0 1 1 1 1 2 1 2 2 1 1 0 ? 1 1

227-Smith et al. From bilateral symmetry to pentaradiality, Tre 0 2 1 0 1 0 0 1 1 1 0 - - - - - - - - - - 1 0 0 1 1 0 0 0 0 0 1 1 0 1 0 0 1 1 2 0 1 0 0 0 2 1 - - - - - 1 2 1 0 1 1 0 0 0 1 0 0 0 0 0 0 0 0 0 0 0 0 0 2 2 0 0 1 ? 1 1 0 1 1 1 1 1 0 0 1 1 2 0 1 0 0 0 0 1 0 0 1 2 1 1 1 1 1 1 1 1 1 0 0 0 0 1 1 1 0 0 1 0 0 0 1 1 1 1 B 1 2 2 1 0 0 0 1 1

228-Letunic & bork. Itol – interactive tree of life 0 2 A 0 1 0 0 1 1 1 0 - - - - - - - - - - 1 0 0 1 1 0 0 ? ? 0 ? 1 0 ? ? ? 1 1 C 1 1 0 0 0 2 1 - - - - - 1 2 1 0 1 1 0 0 ? A 0 0 0 0 0 0 0 0 0 0 0 0 0 C 2 0 0 1 1 1 1 0 1 1 1 1 1 ? 0 1 1 2 0 1 ? ? 1 0 0 0 0 1 2 1 1 1 1 1 1 1 ? 1 ? ? ? ? ? 1 0 0 0 1 0 0 0 ? 0 ? ? B 1 C C ? ? ? ? 1 1

229-Spooner & Ritchie. An unusual phylogeography in the bus 0 2 0 0 1 0 0 - - - 0 - - - - - - - - - - 1 0 0 1 - 0 0 0 1 0 0 1 0 1 1 0 1 - 0 0 1 0 0 0 0 1 - - - - - 1 2 1 0 1 1 0 1 1 1 0 0 0 0 0 0 0 0 0 0 0 0 0 - - - 1 1 0 1 1 0 1 1 1 1 1 0 0 1 1 2 0 1 ? 0 1 0 0 0 0 1 2 1 1 1 1 1 1 1 0 1 0 0 0 1 1 1 1 0 1 0 0 0 0 0 0 1 0 0 ? 1 2 1 1 1 1 1 1

230-Blair hedges & kumar. Timetree of life 0 2 1 0 1 0 0 1 1 1 0 - - - - - - - - - - 1 0 0 1 1 0 0 0 0 0 1 1 0 1 0 1 1 1 2 0 1 0 0 0 2 1 - - - - - 1 2 1 0 1 1 0 0 0 1 2 0 0 0 0 0 0 0 0 0 0 0 0 2 2 0 0 1 1 1 1 0 1 1 1 1 1 0 0 1 1 2 0 1 0 0 0 0 0 0 0 1 2 1 1 1 1 1 1 1 1 1 0 0 0 0 1 1 0 0 0 1 0 1 0 1 1 ? 1 2 1 2 2 0 1 ? ? 1 1

231-Blair hedges & kumar. The timetree of life (book) 0 2 1 0 1 0 0 1 1 1 0 - - - - - - - - - - 1 0 0 1 1 0 0 1 0 0 1 1 0 1 0 1 1 1 2 0 1 0 0 0 2 1 - - - - - 1 2 1 0 1 1 0 0 0 1 2 0 0 0 0 0 0 0 0 0 0 0 0 2 2 0 0 1 1 1 1 0 1 1 1 1 1 0 0 1 1 2 0 1 0 0 0 0 0 0 0 1 2 1 1 1 1 1 1 1 1 1 0 0 0 1 1 1 1 0 0 1 0 1 0 1 1 ? 1 2 1 2 2 0 1 ? ? 1 1

232-Letunic & bork. Interactive tree of life v2 0 2 A 0 1 0 0 1 1 1 0 - - - - - - - - - - 1 0 0 1 1 0 0 ? ? 0 ? 1 0 ? ? ? 1 1 C 0 1 0 0 0 2 1 - - - - - 1 2 1 0 1 1 0 0 ? A 0 0 0 0 0 0 0 0 0 0 0 0 0 C 2 0 0 1 1 1 1 0 1 1 1 1 1 ? 0 1 1 2 0 1 ? ? 1 0 0 0 0 1 2 1 1 1 1 1 1 1 ? 1 ? ? ? ? ? 1 0 0 0 1 0 0 0 ? 0 ? ? B 1 C C ? ? ? ? 1 1

233-Rosindell et al. Onezoom, Tree #1 0 2 A 0 1 0 0 1 1 1 1 1 1 ? 1 1 0 1 0 0 1 1 0 0 1 1 1 1 ? ? ? ? 0 0 ? ? ? 1 1 C 1 1 0 1 1 2 1 - - - - - ? 2 1 0 1 1 0 0 1 A 0 0 ? 0 0 0 0 0 0 ? 0 0 0 C 2 0 0 1 1 1 1 0 1 1 1 1 0 0 0 1 1 2 0 1 0 ? 1 0 0 0 0 1 2 1 1 1 1 1 1 1 ? 1 ? ? ? ? ? 1 0 0 0 1 0 0 1 ? 0 ? ? B 1 C C ? ? ? ? 1 1

234-Rosindell et al. Onezoom, Tree #2 0 2 A 0 1 0 0 1 1 1 1 1 1 ? 1 1 0 0 0 0 1 1 0 0 1 1 1 1 ? ? ? ? 0 0 ? ? ? 1 1 C 1 1 0 1 1 2 1 - - - - - ? 2 1 0 1 1 0 0 1 A 0 0 ? 0 0 0 0 0 0 ? 0 0 0 C 2 0 0 1 1 1 1 0 1 1 1 1 0 0 0 1 1 2 0 1 0 ? 1 0 0 0 0 1 2 1 1 1 1 1 1 1 ? 1 ? ? ? ? ? 1 0 0 0 1 0 0 1 ? 0 ? ? B 1 C C ? ? ? ? 1 1

235-Rosindell et al. Onezoom, Tree #3 0 2 A 0 1 0 0 1 1 1 1 1 1 ? 1 1 0 0 0 0 1 1 0 0 1 1 1 1 ? ? ? ? 0 0 ? ? ? 1 1 C 1 1 0 1 1 2 1 - - - - - ? 2 1 0 1 1 0 0 1 A 0 0 ? 0 0 0 0 0 0 ? 0 0 0 C 2 0 0 1 1 1 1 0 1 1 1 1 0 0 0 1 1 2 0 1 0 ? 1 0 0 0 0 1 2 1 1 1 1 1 1 1 ? 1 ? ? ? ? ? 1 0 0 0 1 0 0 1 ? 0 ? ? B 1 C C ? ? ? ? 1 1
