# Supplementary figures and images for: The treeness of the tree of historical trees of life
Source: PLoS One. 2020 Jan 15;15(1):e0226567. doi: 10.1371/journal.pone.0226567 (PMC6961905; doi:10.1371/journal.pone.0226567)

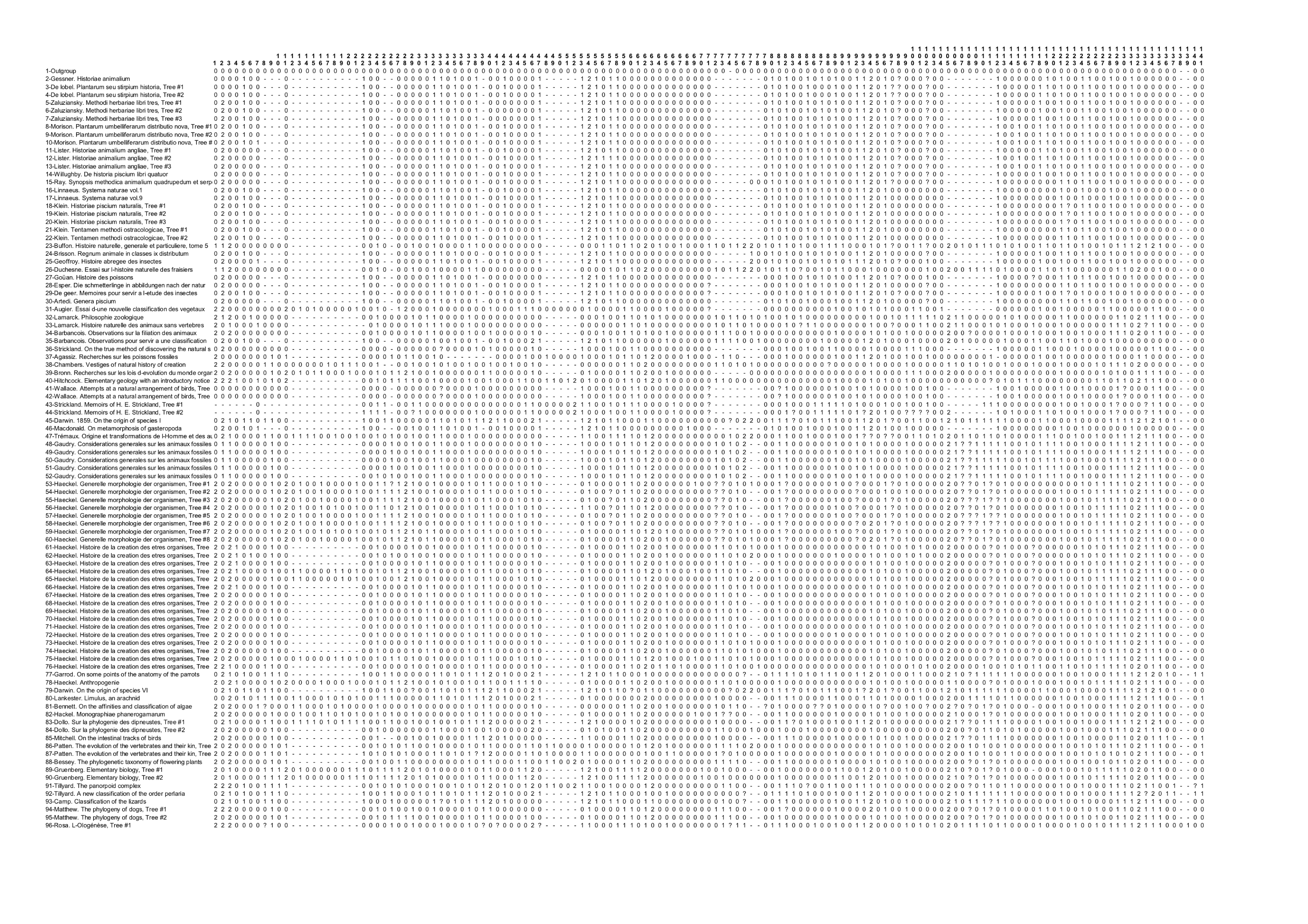

Supplement: S3 File — “?” means “unknown” ; “-” means “irrelevant”. See Annex I for description of trees that are coded and Annex II for description of each of the 141 characters. (PNG) [file pone.0226567.s003.png]
